# Solvent‐Free Synthesis of Core‐Functionalised Naphthalene Diimides by Using a Vibratory Ball Mill: Suzuki, Sonogashira and Buchwald–Hartwig Reactions

**DOI:** 10.1002/chem.202201444

**Published:** 2022-07-13

**Authors:** Lydia A. Panther, Daniel P. Guest, Andrew McGown, Hugo Emerit, Raysa Khan Tareque, Arathy Jose, Chris M. Dadswell, Simon J. Coles, Graham J. Tizzard, Ramón González‐Méndez, Charles A. I. Goodall, Mark C. Bagley, John Spencer, Barnaby W. Greenland

**Affiliations:** ^1^ Department of Chemistry School of Life Sciences University of Sussex Arundel Building 305 Falmer, Brighton BN1 9QJ UK; ^2^ UK National Crystallography Service Chemistry University of Southampton University Road Southampton SO17 1BJ UK; ^3^ Faculty of Engineering & Science FES Engineering & Science School Operations University of Greenwich Old Royal Naval College Park Row London SE10 9LS UK; ^4^ Sussex Drug Discovery Centre School of Life Sciences University of Sussex Falmer, Brighton BN1 9QG UK

**Keywords:** ball milling, core-functionalised naphthalene diimide, coupling reactions, green chemistry, solid-phase synthesis

## Abstract

Solvent‐free synthesis by using a vibratory ball mill (VBM) offers the chance to access new chemical reactivity, whilst reducing solvent waste and minimising reaction times. Herein, we report the core functionalisation of *N*,*N*’‐bis(2‐ethylhexyl)‐2,6‐dibromo‐1,4,5,8‐naphthalenetetracarboxylic acid (Br_2_‐NDI) by using Suzuki, Sonogashira and Buchwald–Hartwig coupling reactions. The products of these reactions are important building blocks in many areas of organic electronics including organic light‐emitting diodes (OLEDs), organic field‐effect transistors (OFETs) and organic photovoltaic cells (OPVCs). The reactions proceed in as little as 1 h, use commercially available palladium sources (frequently Pd(OAc)_2_) and are tolerant to air and atmospheric moisture. Furthermore, the real‐world potential of this green VBM protocol is demonstrated by the double Suzuki coupling of a monobromo(NDI) residue to a bis(thiophene) pinacol ester. The resulting dimeric NDI species has been demonstrated to behave as an electron acceptor in functioning OPVCs.

## Graphical abstract

**The solvent‐free synthesis** of core‐functionalised naphthalene diimide (c‐NDI) residues in a vibratory ball mill is reported. Twenty‐one naphthalene diimide‐based products were produced by using Suzuki, Sonogashira and Buchwald‐Hartwig palladium coupling reactions, including dimeric diimide products that are constituents in functioning organic photovoltaic solar cells. These reactions are rapid (60 to 90 minutes), do not require additional heating, and are tolerant of air and atmospheric moisture.

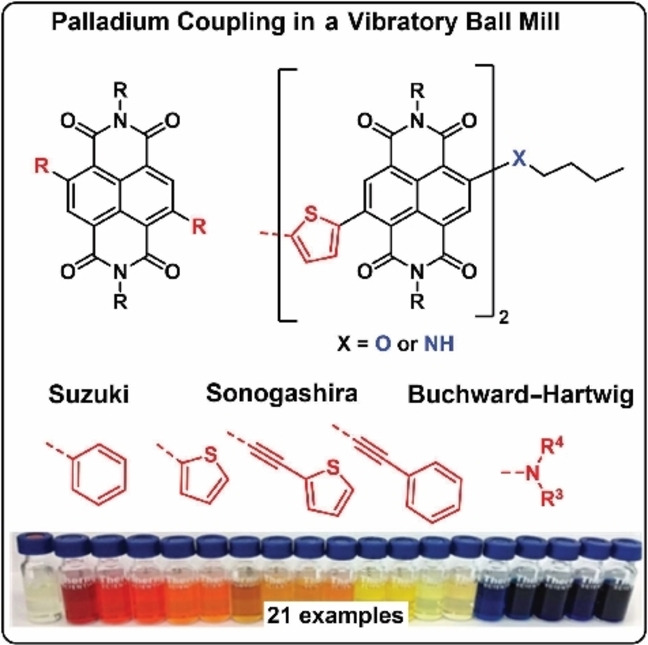

## Introduction

**Figure 1:**
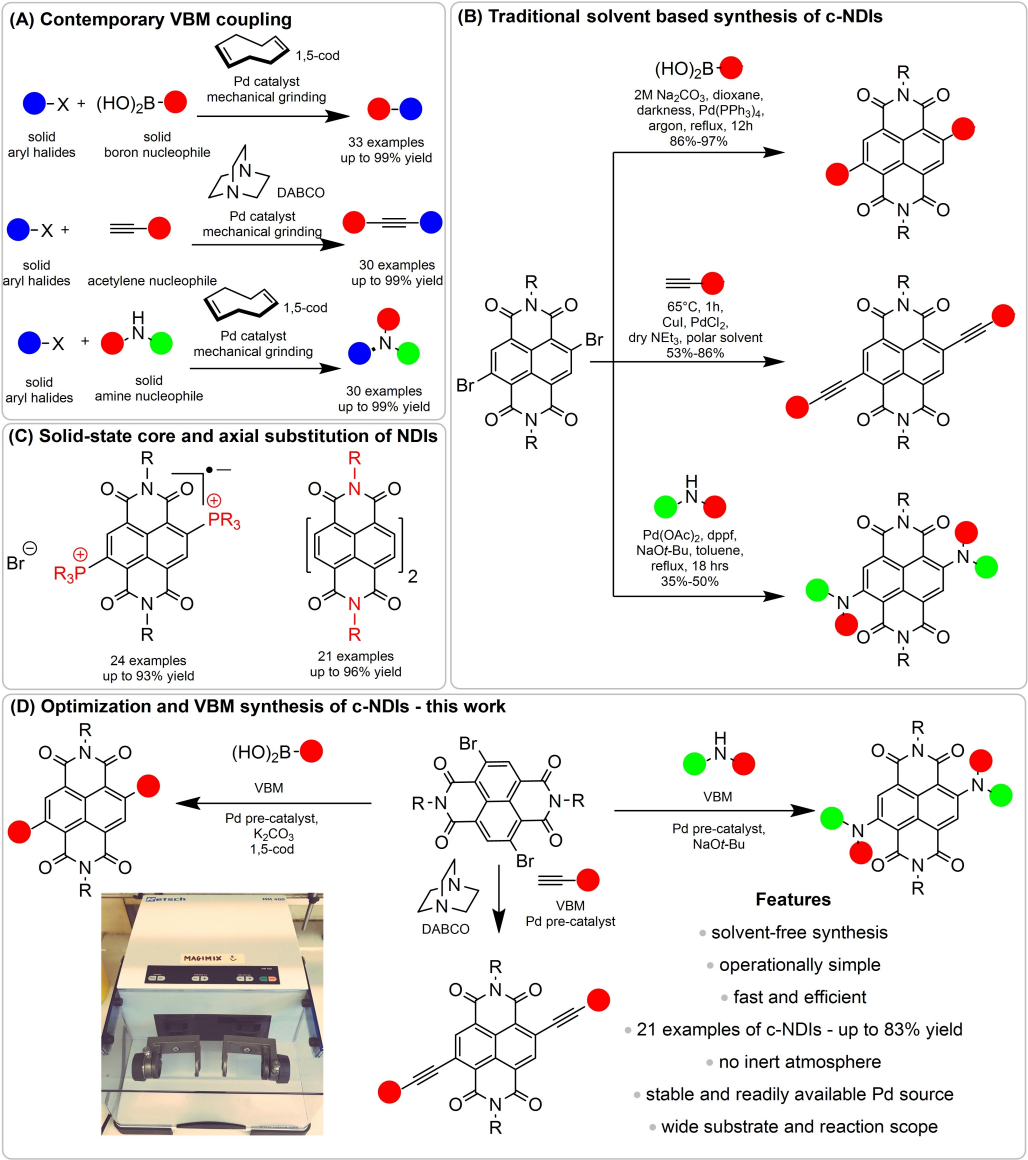
Coupling reactions using A) VBM and B)–D) syntheses of c‐NDIs. A) Contemporary VBM couplings on non‐NDI derivatives; B) Traditional solvent‐based synthesis of c‐NDIs. C) Solid‐state core and axial substitution of NDIs. D) Rapid optimisation and VBM synthesis of c‐NDIs. 1,5‐cod=1,5‐cyclooctodiene carried out in this work.

As the global population increases, and pressure builds on natural resources, the drive to discover greener chemical processes that reduce or eliminate hazardous waste is critical.^[1]^ The use of solvent in an organic reaction can account for over 80 % of the total mass and waste produced,^[2]^ which can be difficult and expensive to dispose of. Eliminating the use of solvents in reactions is one effective strategy towards greener synthesis.^[3]^ Mechanochemistry, which can be carried out using either a vibratory ball mill (VBM) or a planetary ball mill (PBM), is a powerful strategy for the rapid, clean, and environmentally friendly synthesis of compounds. Chemical extrusion, a processing technique based upon mechanochemical transformation, was identified by IUPAC as one of ten world‐changing technologies in 2019.^[4]^ Mechanochemistry is becoming an established methodology for carrying out a range of chemical reactions using little or no solvents; for example, in the synthesis of amino esters,^[5]^ hydrazones,^[6]^ peptides^[7]^ and nitrones.^[8]^ More recently, metal catalysed reactions such as click chemistry,^[9]^ activation of acyl azides^[10]^ and couplings which include Suzuki,^[11]^ Sonogashira (either containing copper^[12]^ or copper free^[13]^) and Buchwald‐Hartwig aminations^[14]^ have all been successfully carried out in a ball mill (Figure 1A). Ito and co‐workers[^11a^, ^11c^, ^14a^] found that addition of 1,5‐cyclooctodiene (1,5‐cod) led to increased yields in Pd‐catalysed cross coupling reactions on non‐NDI derivatives. Referred to in their work as a liquid‐assisted grinding (LAG) agent, it is suggested that the improved efficiency of the reaction is a consequence of reduced aggregation of the Pd catalyst, where the 1,5‐cod presumably acts as a ligand for the metal.

A substantial body of research using ball milling conditions has concentrated on medicinal chemistry applications,^[15]^ yet materials chemistry appears to have been relatively neglected, although this area has been reviewed recently.^[16]^ Selected examples are the VBM synthesis of poly(urethane)s by Wilson and co‐workers,^[17]^ in addition to cross‐coupling reactions leading to the production of poly(phenylene vinylene)^[18]^ and poly(*para*‐phenylene) polymers in a Suzuki reaction.^[19]^ This last synthesis is particularly laudable for its use of palladium milling balls to generate the catalytic species in situ, rather than the addition of a specific molecular precatalyst.

The relative lack of attention on solvent‐free synthesis for materials chemistry is more surprising as a consequence of the disparate scales of production between fine chemicals and materials such as polymers. European polymer demand has been estimated to be 61.8 million metric tonnes in 2018;^[20]^ compared to total imports of pharmaceutical products in 2019 of 5.5 million tonnes.^[21]^

Here, we have initiated a research programme to synthesise core‐functionalised naphthalene diimide species (c‐NDIs) which have well established applications in organic electronics,^[22]^ solar cell technology^[23]^ and artificial photosynthesis.^[24]^ In each of these applications, core functionalisation^[25]^ is the key synthetic step in order to tune the electronic properties of the resulting molecules, by the manipulation of the energy levels of the conjugated system. This is especially important because unsubstituted NDIs show relatively limited absorption in the visible region, which make them unattractive candidates for use in organic electronics. The synthesis of c‐NDIs is reported in solution‐based chemistry using Suzuki,^[26]^ copper facilitated Sonogashira^[27]^ and Buchwald‐Hartwig^[28]^ couplings (Figure 1B). These processes often necessitate long reaction times (e. g., 18 h), high temperatures (typically solvents at reflux, particularly toxic chlorinated or flammable aromatic solvents), strict anaerobic conditions, employing glove boxes and Schlenk line techniques. Sometimes, they also require light to be excluded.^[26]^

To date, only Kumar and Mukhopadhyay have produced c‐NDIs using mechanochemical means.^[29]^ However, their VBM synthesis generated C−P bonds to give di‐phosphonium substituted radical ions [NDI(PR_4_)_2_]^.+^Br^−^, rather than producing new C−C bonds at the core of the NDI species (Figure 1C). James and co‐workers, have examined the axial (not core) functionalisation of mono naphthalene anhydrides and perylene dianhydride by VBM and extrusion techniques (Figure 1C).^[30]^

Herein, we report the solventless synthesis using VBM of c‐NDIs by either Suzuki, Sonogashira without the addition of an external copper source (although we recognise that trace levels of Cu might be present in the reagents) or Buchwald‐Hartwig coupling reactions (Figure 1D). These reactions require no solvent and proceed rapidly (≤1.5 h) using commercially available palladium sources, (frequently Pd(OAc)_2_) and can be carried out under bench top conditions.

## Results and Discussion

### Optimisation of studies for Suzuki synthesis of c‐NDIs

Our initial studies into the synthesis of c‐NDI derivatives focused on the Suzuki coupling reaction,^[31]^ a well‐known solution‐state protocol used to produce this family of compounds.^[26]^ These optimisation reactions required the production of significant quantities of NDI‐Br_2_ (**1 b**), which was accessed using modified literature methods^[32]^ in batches of up to 15 g without column chromatography.

An initial screening program using VBM conditions (zirconium oxide jars and milling balls) was carried out by varying the nature of the base, precatalyst and LAG additive, for the Suzuki coupling between **1 b** and (4‐methoxyphenyl)boronic acid to yield **2 a** (Table 1).

**Table 1:** Optimisation of Suzuki coupling reactions and structures of precatalysts.

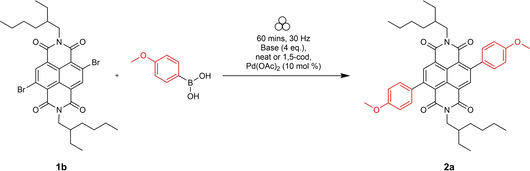			
Entry	Pd	Base (LAG)	Conversion to **2 a** by ^1^H NMR spectroscopy [%]
1	Pd(OAc)_2_	Hünig's base	25
2	Pd(OAc)_2_	NEt_3_	31
3	Pd(OAc)_2_	LiOH	44
4	Pd(OAc)_2_	NaOH	>98
5	Pd(OAc)_2_	KOH	83
6	Pd(OAc)_2_	Na_2_CO_3_	54
7	Pd(OAc)_2_	K_2_CO_3_	>98
8	Pd(OAc)_2_	Cs_2_CO_3_	84
9	Pd(OAc)_2_	K_3_PO_4_	86
10	Pd(OAc)_2_	NaOAc	24
11	Pd(OAc)_2_	K_3_PO_4_ [1,5‐cod]	>98
12	XPhos Pd G3 (**C1**)	K_3_PO_4_	>98
13	SPhos Pd G2 (**C2**)	K_3_PO_4_	86
14	CataCXium®A Pd G3 (**C3**)	K_3_PO_4_	90
15	A Phos Pd G3 (**C4**)	K_3_PO_4_	93
16	P(Cy_3_) Pd G3 (**C5**)	K_3_PO_4_	97
17	Pd‐PEPPSI™‐Ipent (**C6**)	K_3_PO_4_	>98
			
	**C1**	**C2**	**C3**
	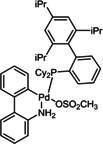	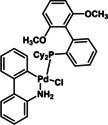	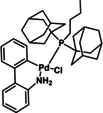
	**C4**	**C5**	**C6**
	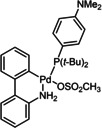	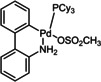	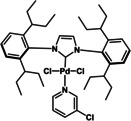

The ratio of the mass of reagents to the volume of grinding jars was ca. 20 mg cm^−3^. Reactions were carried out for 1 h at room temperature and a precatalyst loading of 10 mol%. Conversions were determined by ^1^H NMR spectroscopy in CDCl_3_ by comparing the loss of starting material vs. gain of product_._ When used, the LAG is ca. 10 wt% of the total reaction mass.

Initially, we selected Pd(OAc)_2_ as an inexpensive and widely available precatalyst and screened a range of bases (Table 1). Organic bases that are liquid at room temperature performed poorly in these reactions (<31 % conversion, entries 1 and 2) compared to solid, inorganic hydroxides and carbonates (44 % to >98 %, entries 3–8). Notably, the use of both NaOH and K_2_CO_3_ led to essentially full conversion (entries 4 and 7). Alternative inorganic bases, such as Cs_2_CO_3_ and K_3_PO_4_, afforded slightly lower conversions (entries 8 (84 %) and 9 (86 %)).

To be able to observe the impact of other possible variables, we selected the base K_3_PO_4_ that resulted in a moderate conversion (entry 9, 86 %), for additive and catalyst optimisation studies. As expected, ^[11a]^ addition of the LAG, 1,5‐cod, led to increased yields (entry 10 (84 %) vs. entry 11 (>98 %)). We then screened a range of commercially available precatalysts containing different phosphine and NHC ligands (**C1**–**C6**, entries 12–17), to compare against the results for Pd(OAc)_2_ (entries 1–11). These more active precatalysts led to higher yields than were observed for Pd(OAc)_2_ when combined with the moderately effective base, K_3_PO_4_. For example, XPhosPdG3 (**C1**) and Pd‐PEPPSI™‐Ipent (**C6**) with K_3_PO_3_ led to virtually complete conversion as determined by ^1^H NMR spectroscopy.

### Substrate scope for the synthesis of c‐NDIs by Suzuki coupling

**Scheme 1:**
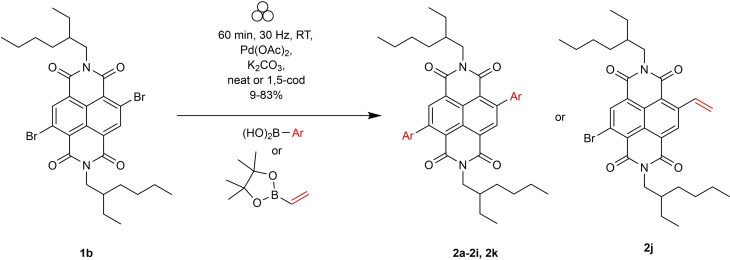
Synthesis of Suzuki‐coupled c‐NDIs.

**Figure 2:**
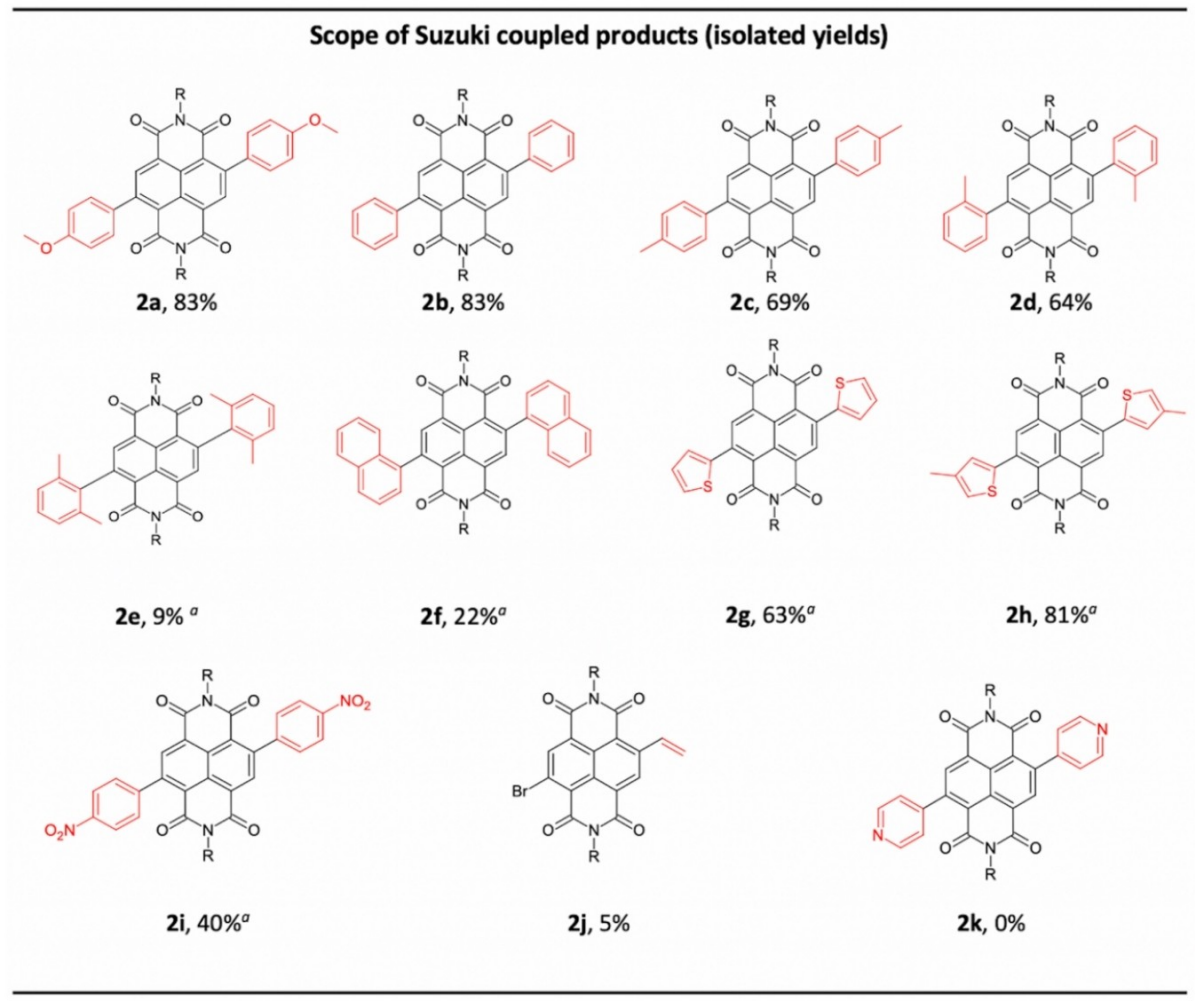
Scope of c‐NDI products **2 a**–**2 k** synthesised by Suzuki coupling using VBM methods and isolated by flash column chromatography. Isolated yields. [a] Cycloocta‐1,5‐diene (10 wt%) as LAG agent. R=2‐ethylhexyl chain.

The non‐hygroscopic nature of K_2_CO_3_ compared to NaOH made this base operationally simpler to use in a VBM. Next, the scope of the reaction was assessed (Scheme 1) using a selection of aryl and vinyl boronic acids to yield **2 a**–**2 k**, which were each isolated by flash column chromatography (Figure 2). These conditions gave an excellent isolated yield for the phenyl substituted c‐NDI after 60 min reaction time (**2 b**, 83 %). This may be compared to reported solution‐state synthetic routes to phenyl substituted c‐NDIs (10 %; Suzuki coupling, 14 h, 100 °C, under nitrogen)[^28c^, ^33^] and 80 % (CH arylation, 72 h, benzene under reflux).^[34]^

Further expanding the substrate scope of this solid‐state reaction revealed a small drop in yield when the coupling partner was substituted in either the *para* or *ortho* position, viz. **2 c** (69 %) and **2 d** (64 %). Couplings performed with the more sterically hindered 2,6‐dimethylaryl‐substituted boronic acid, furnished **2 e** in 9 % yield. Thiophene and substituted thiophene containing c‐NDIs were synthesised in yields over 80 % (**2 g**, **2 h**), comparable to the reported solution‐state synthesis of **2 g** in a Stille coupling^[35]^ (80 %, 5 h, 90 °C, N_2_) and CH arylation (96 %, 17 h,^[32a]^ and 80 %^[33]^ in toluene at reflux, respectively). Aryl boronic acids substituted with electron donating groups were readily tolerated (**2 a** (83 %)) although, as expected, a reduction in yield was observed for an electron deficient aryl boronic acid derivative (**2 i** (40 %)). Attempted di‐addition of vinyl pinacol ester only yielded the monosubstituted vinyl c‐NDI **2 j** in a low yield of 5 %. Disappointingly, the dipyridine c‐NDI **2 k** could not be isolated despite closely related c‐NDIs being reported by Bhosale and co‐workers from solution‐state methodologies.^[26]^

**Figure 3:**
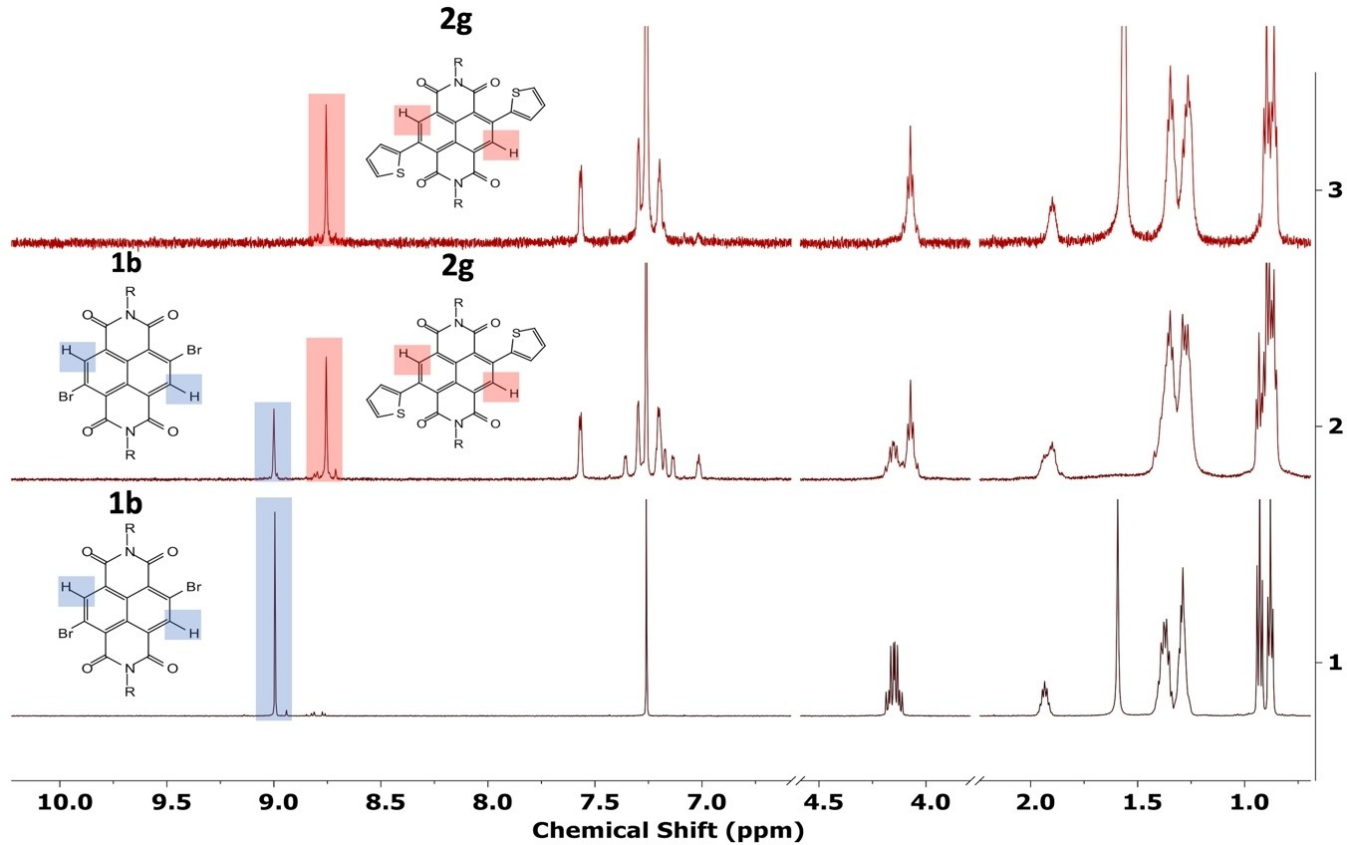
Stack of the spectra used to determine the conversion of **1 b** to **2 g** with and without the addition of LAG agent. ^1^H NMR spectra: 1) starting material **1 b**; 2) the crude reaction mix for **1 b** to **2 g** without LAG (conversion 64 %); 3) the crude reaction mix for **1 b** to **2 g** with LAG (conversion >98 %).

For several substituents, addition of 1,5‐cod as a LAG agent markedly increased the conversion of **1 b** to the desired c‐NDIs. This is exemplified in Figure 3 for the synthesis of **2 g**, which, shows the ^1^H NMR spectrum of the starting material (**1 b**, Figure 3, spectrum 1) together with the spectra of the crude reaction mixtures, straight after completion of ball milling. For the reaction that did not contain the LAG agent (Figure 3, spectrum 2) the conversion as measured by the ratio of the aromatic proton signals for **1 b** and **2 g** (δ8.99 and 8.75 ppm, respectively) was 64 %, whereas essentially complete conversion was observed in the reaction that contained the LAG agent (Figure 3, spectrum 3). For full conversion data, see Table S1.

### Optimisation studies and substrate scope for Sonogashira synthesis of c‐NDIs

Sonogashira reactions to form c‐NDIs are a less well studied derivatisation route compared to their Suzuki congeners.[^22^, ^26^] Notwithstanding, diacetylene‐substituted c‐NDIs have suitable electronic properties for use in OLEDs[^27c^, ^36^] and n‐type semiconductor materials^[37]^ amongst other applications.^[38]^ Typical Sonogashira reaction conditions include the use of either an organic base such as diazabicyclooctane (DABCO)^[13a]^ or an inorganic base such as K_3_PO_4_,^[39]^ and a palladium source.^[40]^ All reported solution‐state Sonogashira coupling reactions to form c‐NDIs require the addition of a copper co‐catalyst.[^27a^, ^27c^, ^41^] Using these precedents as guidance, a brief optimisation study was carried out in order to form the diphenylacetylene substituted c‐NDI **3 a** under VBM conditions (Table 2). Couplings using precatalyst **C2**, or Pd(OAc)_2_ and DABCO without the addition of an external copper source,^[42]^ resulted in essentially complete conversion to the disubstituted product **3 a** as determined by ^1^H NMR spectroscopy.

**Table 2:** Optimisation of the Sonogashira coupling between phenylacetylene and **1 b** in the solid state with cycloocta‐1,5‐diene (10 wt%) as LAG agent.

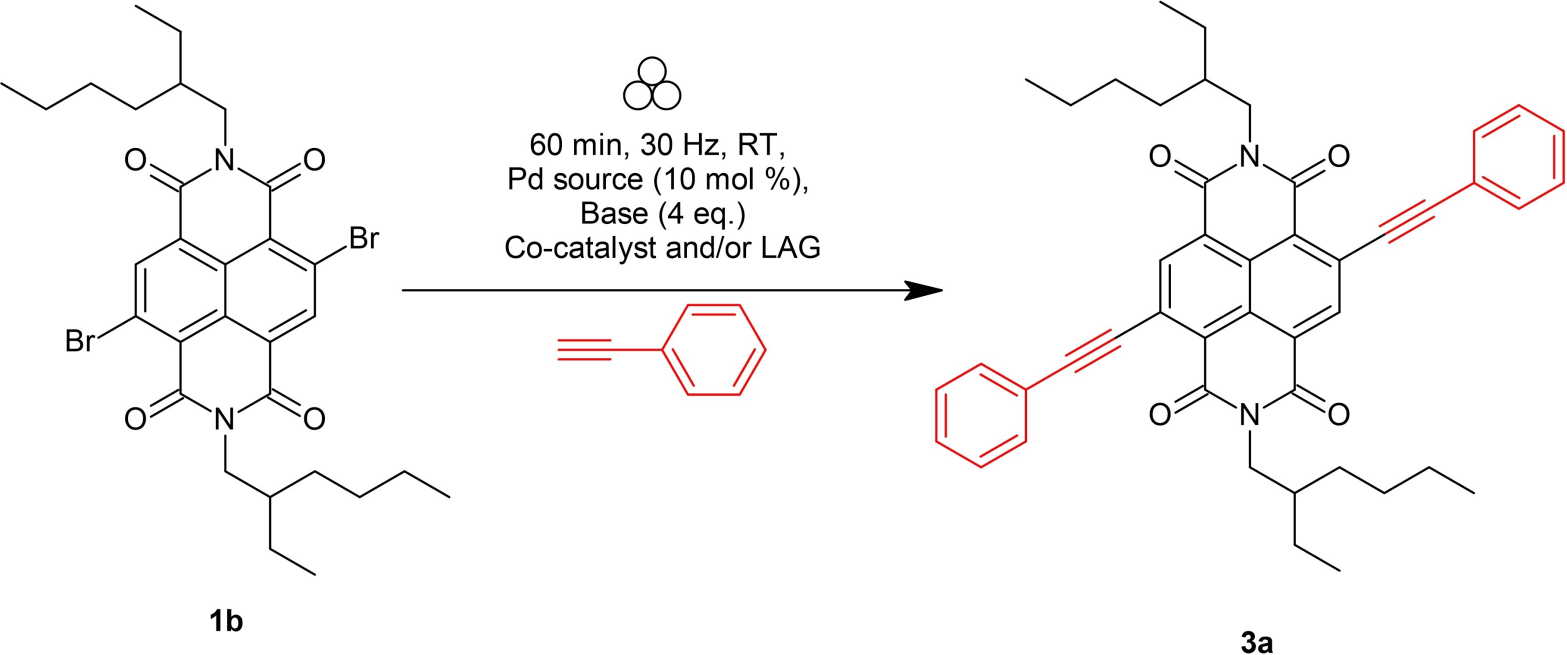						
Entry	Pd [10 mol%]	Base	Co‐catalyst	LAG	*t* [min]	Conversion to **3 a** by ^1^H NMR [%]
1	**C1**	K_3_PO_4_	CuI	none	60	32
2	**C1**	K_3_PO_4_	CuI	1,5‐cod	60	45
3	**C2**	NEt_3_	CuI	none	60	>98
4	**C2**	DABCO	none	none	60	>98
5	Pd(OAc)_2_	DABCO	none	none	60	>98

**Scheme 2:**
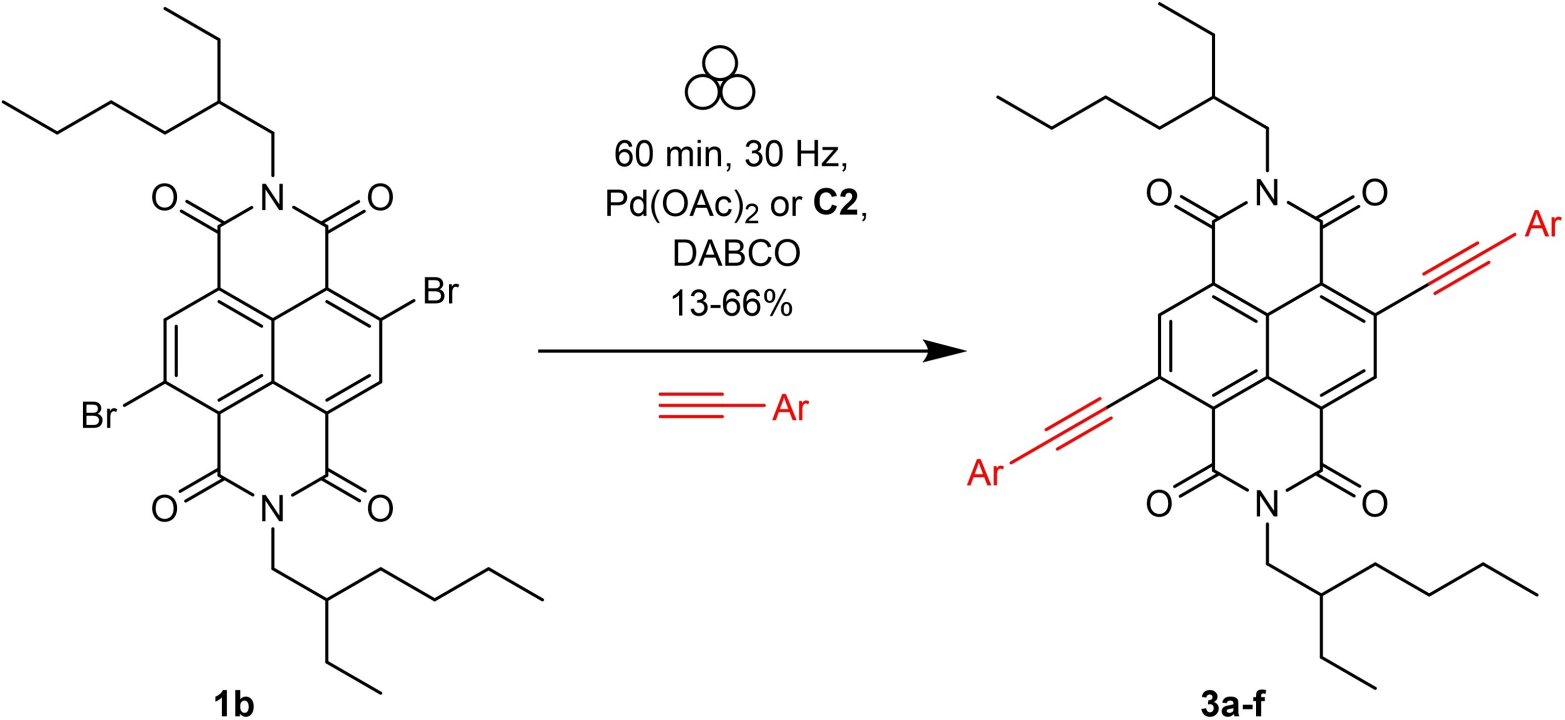
Synthesis of Sonogashira c‐NDIs.

**Figure 4:**
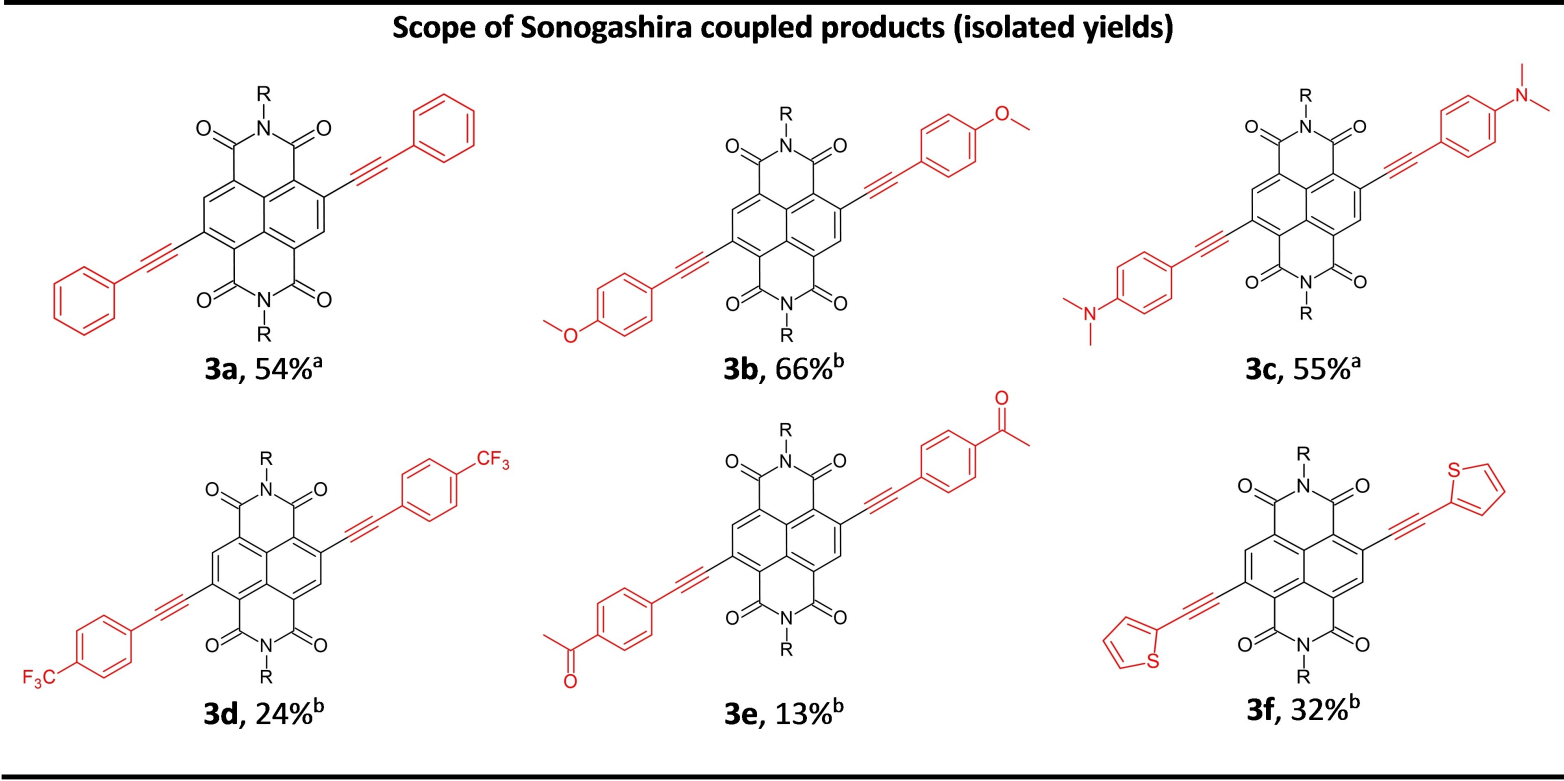
Scope of c‐NDI synthesised by Sonogashira coupling (**3 a**–**3 f**) using VBM and isolated by flash column chromatography. Isolated yields after flash column chromatography reported. [a] Using precatalyst Pd(OAc)_2_, [b] using precatalyst **C2**; R=2‐ethyl hexyl chain.

The scope of the reaction was then assessed (Scheme 2) using a selection of aryl acetylenes to yield **3 a**–**3 f** under conditions without the addition of a copper source where each product was isolated by flash column chromatography (Figure 4). Double substitution of **1 b** by phenylacetylene gave **3 a** (54 % isolated yield) after only 60 min reaction time. *Para*‐substituted electron donating aryl derivatives containing methoxy or *N*,*N*‐dimethyl groups proceeded in similar yields (**3 b** (66 %) and **3 c** (55 %)). Reactions with aromatic coupling partners containing electron withdrawing groups were considerably less efficient. For example, the trifluoromethyl and keto‐aryl c‐NDI derivatives were isolated in 24 % (**3 d**) and 13 % (**3 e**) yield, respectively. Alkyne‐thiophene substituted c‐NDIs have been studied for use in OLEDs and n‐type semiconducting materials^[37]^ and **3 f** could be accessed under these VBM conditions (32 %).

### Optimisation studies and substrate scope for Buchwald‐Hartwig amination of c‐NDIs

Aminated c‐NDIs have been demonstrated to behave as electron donor triads which exhibit spin‐orbit charge‐transfer intersystem crossing.^[28c]^ Compounds that have these properties are actively being studied for use in photo‐dynamic therapy (PDT),^[43]^ triplet‐triplet annihilation (TTA) upconversion,^[44]^ photocatalysis^[45]^ and photovoltaic applications.^[46]^

Currently, there are three solution‐state studies which furnish aminated c‐NDIs in modest yields (ca. 22–55 %).[^28a^, ^47^] Each reports their synthesis using similar Buchwald‐Hartwig type conditions: long reaction times (typically >12 h) with an inert solvent, a strong base such as sodium *tert*‐butoxide, a palladium source, and a diaryl secondary amine as the coupling partner.

Using the solution‐phase optimised conditions as a starting point for our solid‐state studies,^[12]^ we carried out an optimisation study concerning the addition of carbazole to **1 b** (Table 3). Using precatalyst **C3**, we observed that increasing the reaction time from 60 to 90 min, led to increased conversions (entries 1 and 2 (73 to 84 %, respectively)). Furthermore, addition of the of the LAG agent, 1,5‐cod, led to a further increase in conversion (entry 3 (>98 %)). The use of either precatalyst **C2** or Pd(OAc)_2_, each with sodium *tert*‐butoxide as base, also resulted in essentially complete conversion to the disubstituted product as determined by ^1^H NMR spectroscopy (entries 4 and 5).

**Table 3:** Optimisation of the Buchwald‐Hartwig amination between carbazole and **1 b** under VBM conditions.

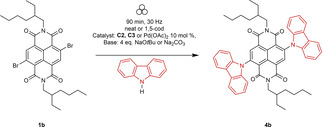					
Entry	Pd	Base	*t* [min]	LAG	Conversion to **4 b** by ^1^H NMR [%]
1	**C3**	NaO*t*Bu	60	none	73
2	**C3**	NaO*t*Bu	90	none	84
3	**C3**	NaO*t*Bu	90	1,5‐cod	>98
4	**C2**	NaO*t*Bu	90	none	>98
5	Pd(OAc)_2_	NaO*t*Bu	90	none	>98

**Scheme 3:**
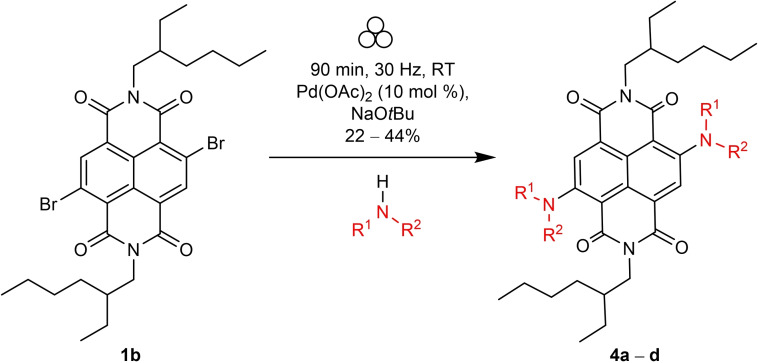
Synthesis pathway for Buchwald‐Hartwig aminated c‐NDIs.

**Figure 5:**
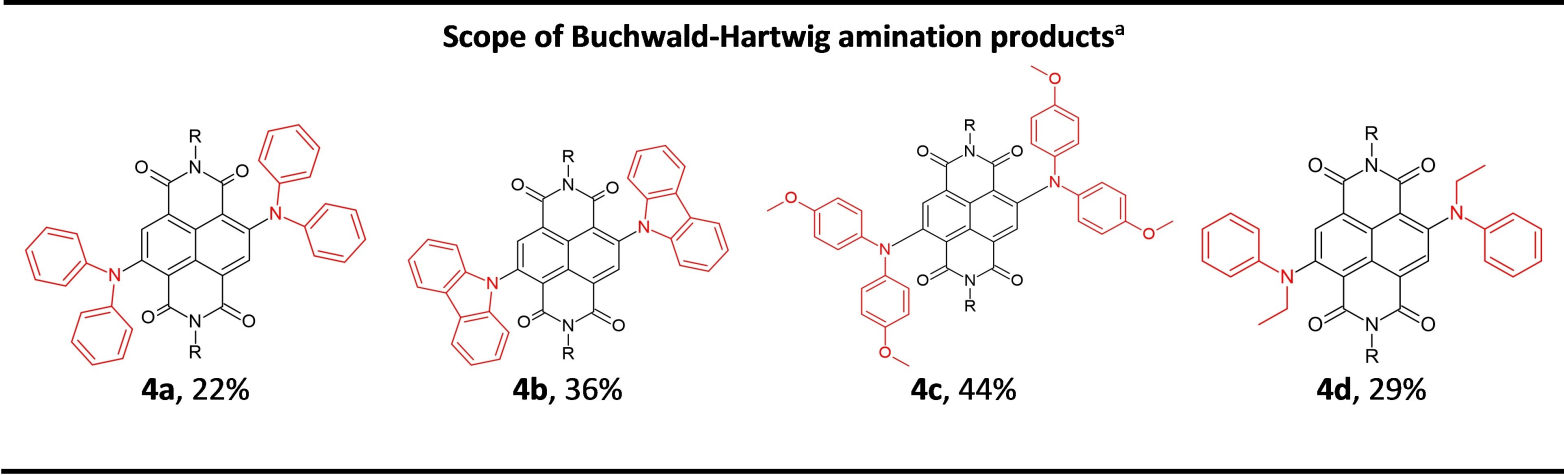
Scope of c‐NDI products **4 a**–**4 d** synthesised by Buchwald–Hartwig amination using VBM methods. [a] Isolated yields after flash column chromatography; R=2‐ethylhexyl chain.

The scope of the reaction was assessed (Scheme 3) using a range of diaryl and aryl/alkyl secondary amines to yield **4 a**–**4 d** in the solid state (Figure 5). These reactions proceeded in similar yields to the those reported for comparable solution‐state reactions (**4 a** (22 %), vs. 20–50 % in solution[^28a^, ^28c^]) and carbazole (**4 b** (36 %) vs. 42–43 % in solution[^28a^, ^28c^]). Electron rich groups were readily tolerated, for example *para*‐methoxy substituted diphenyl amine gave **4 c** in 44 % yield (cf. 42 %^[28a]^ reported in the solution phase). The use of *N*‐ethylaniline as a coupling partner led to the production of mono‐aryl‐mono‐alkyl cNDI, **4 d** (29 %), which represents the first time, to the best of our knowledge, that alkyl substituted amines (rather than diaryl substituted amines) have been coupled to NDI cores.

### Impurity analysis after VBM synthesis

A problem encountered when synthesising species using cross reactions is the difficulty in removing relatively high levels of heavy metals in the products.^[48]^ This could be exacerbated when using VBM synthesis by the possibility of the surface of the reaction vessel being eroded and furthering contaminating the products. Therefore, inductively coupled plasma ‐ mass spectrometry (ICP‐MS) was carried out on selected samples to see if residual Zr and Pd were present from the synthesis. **2 a** and **3 a** had Zr levels below the instrumental limit of detection and had low residual levels of Pd (0.002 (**2 a**) and 0.003 (**3 a**) wt. %). Compound **4 c** was found to have low, but measurable, levels of both of Zr (0.046 wt. %) and Pd (0.009 wt. %).

### UV/vis absorption properties of c‐NDIs

**Figure 6:**
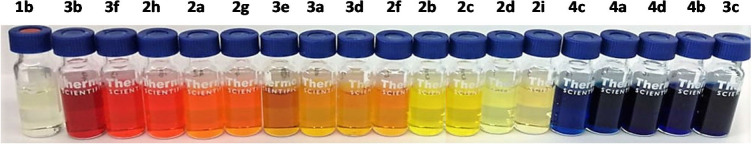
Photograph of 1 mM solutions of the starting material **1 b** (left) and cross‐coupled products **2 a**–**4 d** in chloroform under white light.

A key design element of materials intended for electronic applications is an accessible and tuneable HOMO‐LUMO energy gap, which is also responsible for the highly coloured nature of this class of molecule.[^41^, ^49^] The panchromatic nature of c‐NDIs produced in this work can be seen in their solution‐state absorption properties, where a broad range of colours is evident (Figure 6).

**Figure 7:**
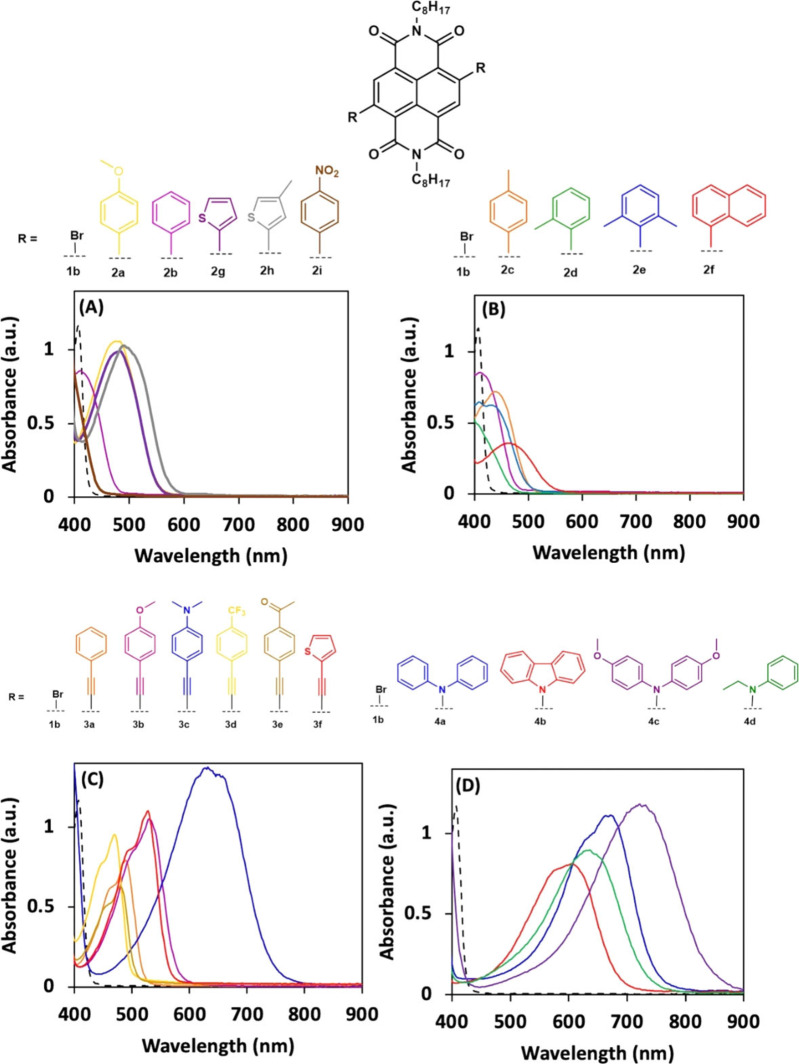
Photophysical data of c‐NDIs as observed by UV/vis absorption spectroscopy (0.2 mM, CHCl_3_) for A), B) Suzuki, C) Sonogashira and D) Buchwald–Hartwig coupled products.

The UV‐Vis spectroscopic data for all the c‐NDI products are presented in Figure 7. The introduction of substituents on the naphthalene core led to significant changes in the absorption spectra for the c‐NDIs compared to the dibromo‐NDI **1 b** (dashed black line in Figure 7). All compounds, including **1 b**, exhibit a high energy absorption in the *λ*_max_ 310–380 nm (Table 4) region, which has been attributed to the NDI π‐π* transition.[^27a^, ^27b^] The c‐NDIs (except dinitro species, **2 i**) also exhibited a lower energy band with *λ*_max_ between 430–700 nm, which corresponds to the intramolecular charge transfer (ICT) transition.^[50]^

**Table 4:** UV‐Vis absorbance values of **1 b** and c‐NDIs **2 a**–**4 d** recorded at 0.2 mM in chloroform.

c‐NDI	*λ*_abs_ [nm]		c‐NDI	*λ*_abs_ [nm]	
	π‐π* transition	ICT transition		π‐π* transition	ICT transition
**1 b**	357, 364	404	**3 a**	321, 370, 381	493
**2 a**	303, 359, 379	472	**3 b**	340, 374	526
**2 b**	357, 375	432	**3 c**	364, 396	646
**2 c**	359, 377	447	**3 d**	318, 374, 383	476
**2 d**	357, 375	434	**3 e**	329, 372, 381	485
**2 e**	358, 374	435	**3 f**	340, 360, 385	532
**2 f**	303, 359, 375	484	**4 a**	303, 346, 373	660
**2 g**	305, 364, 379	491	**4 b**	333, 353, 379	596
**2 h**	310, 362, 379	495	**4 c**	301, 360, 392	718
**2 i**	303, 362, 377	NS	**4 d**	336, 374	626

NS: not seen.

Comparison of the ICT transition value of the starting material **1 b** to those measured for the c‐NDIs **2 a**–**2 k** shows that the addition of a phenyl ring through a carbon–carbon σ bond to the naphthalene core induces a bathochromic shift (from 404 nm (**1 b**) to 432 nm (**2 b**)). This is as a consequence of the extension of the π‐conjugation region to the phenyl groups from the NDI core. Increasing π‐conjugation further, for example by introduction of a naphthalene substituent onto the core (**2 f**), shows an even greater bathochromic shift in the ICT transition, to 484 nm.

The *para*‐tolyl analogue **2 c** displayed a measurable additional bathochromic shift in the low energy transition when compared to the unsubstituted analogues (447 nm (**2 c**) compared to 432 nm (**2 b**)). However, *ortho*‐mono‐ or dimethyl substitution of the aryl group had a negligible effect on the energy of the ICT transition (less than 3 nm difference compared to **2 b**). The presence of electron‐rich substituents such as 3‐methoxyaryl (**2 a**) or thiophene (**2 g**) shifts the ICT transition to significantly lower energies (472 and 491 nm respectively). The methyl substituted more electron rich thiophene **2 h** exhibited the lowest energy absorption in the Suzuki c‐NDI series, at 495 nm. Conversely, no significant electronic transitions were observed below 400 nm when a nitro substituent was on the aromatic ring attached to the NDI (**2 i**). This may be as a consequence of either a lack of an ICT transition in this species, or a hypsochromic shift in the ICT transition (to 377 nm) when compared to unsubstituted **2 b** (432 nm).

When studying the absorption characteristics of the c‐NDIs produced by Sonogashira coupling reactions, the products generally exhibit a lower energy ICT transition (476–646 nm **3 a**–**3 f**) than those produced by the Suzuki reaction (432 to 495, **2 a**–**2 h**). This is consistent with the triple bond extending the region of conjugation compared to **2 a**–**2 h**. It also has been postulated that the steric clash between the *ortho* protons of the phenyl and the NDI core is reduced by the inclusion of the alkyne residue which, in turn allows more efficient orbital overlap in the π‐conjugated system.^[49]^ This narrowing of the HOMO‐LUMO gap is evident as a red‐shift of the ICT transition by 61 nm when comparing **3 a** (493 nm) to **2 b** (432 nm). The presence of electron‐rich *para*‐substituents such as a methoxy or NMe_2_‐aryl, or indeed a thiophene residue on the c‐NDI (**3 b**, **3 c** and **3 f**) led to a decrease in the energy of the ICT transition (526, 646, 532 nm, respectively) compared to the unsubstituted phenyl c‐NDI (**3 a**, 493 nm). In contrast, electron withdrawing *para*‐CF_3_ (**3 d**, 476 nm) or acetyl substituted aryl analogues (**3 e**, 485 nm) show hypsochromic shifts compared to **3 a** (493 nm).

Finally, c‐NDIs produced from the Buchwald‐Hartwig reaction exhibited the largest stabilisation of the HOMO‐LUMO gap for a single series, with ICT transition values between 596–718 nm. Solutions of carbazole substituted NDI **4 b** were deep blue as a consequence of an ICT transition at 596 nm. This is significantly greater in wavelength than that observed for c‐NDIs containing either phenyl/ethyl (**4 d**, 626 nm) or diphenyl (**4 a**, 660 nm) substituents. Therefore, it appears that the rigidity of the carbazole as a consequence of the fused rings reduces the efficiency of the internal charge transfer process compared to the conformationally less constrained molecules in this series (e. g., **4 a** or **4 d**). The lowest‐energy ICT transition for the molecules studied in this work was observed for the methoxy‐substituted diphenyl amine c‐NDI (**4 d**) which exhibited an ICT transition of 718 nm compared to 404 nm for **1 b**.

### Single‐crystal X‐ray analysis

**Figure 8:**
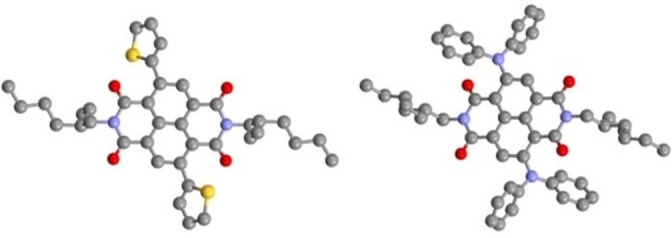
X‐ray crystal structures of c‐NDI **2 g** (left) and **4 a** (right). Minor disorder components and hydrogen atoms are omitted for clarity. Only one independent molecule is shown of **4 a**.

Single crystals for X‐ray structure determination were grown by slow diffusion of heptane into a pyridine solution of **2 g** (Figure 2) and **4 a** (Figure 5). The resulting solid‐state structures revealed the correct regiochemistry of the product, and, therefore, by inference, the substitution pattern of **1 b**. Crystal packing for **2 g** is a monoclinic crystal system with P2_1_/*n* space group whereas **4 a** displays a triclinic crystal system with *P* $\bar{1}$ comprising two independent molecules with similar conformations (Figure 8). The branched alkane moieties **2 g** and **4 a** and the thiophene moiety of **2 g** are disordered in the crystal structures (see the Supporting Information for details). There are no dominant, overarching structural features displayed in either structure. The major disorder component of **2 g** has a very weak thiophene C−H⋅⋅⋅O short contact (C⋅⋅⋅O distance 3.205(10) Å). The structure of **4 a** contains four very weak aromatic C−H⋅⋅⋅O short contacts (C⋅⋅⋅O distance 3.236(7)–3.264(6) Å).

### Synthesis of c‐NDI dimeric systems suitable for OPVC applications

**Scheme 4:**
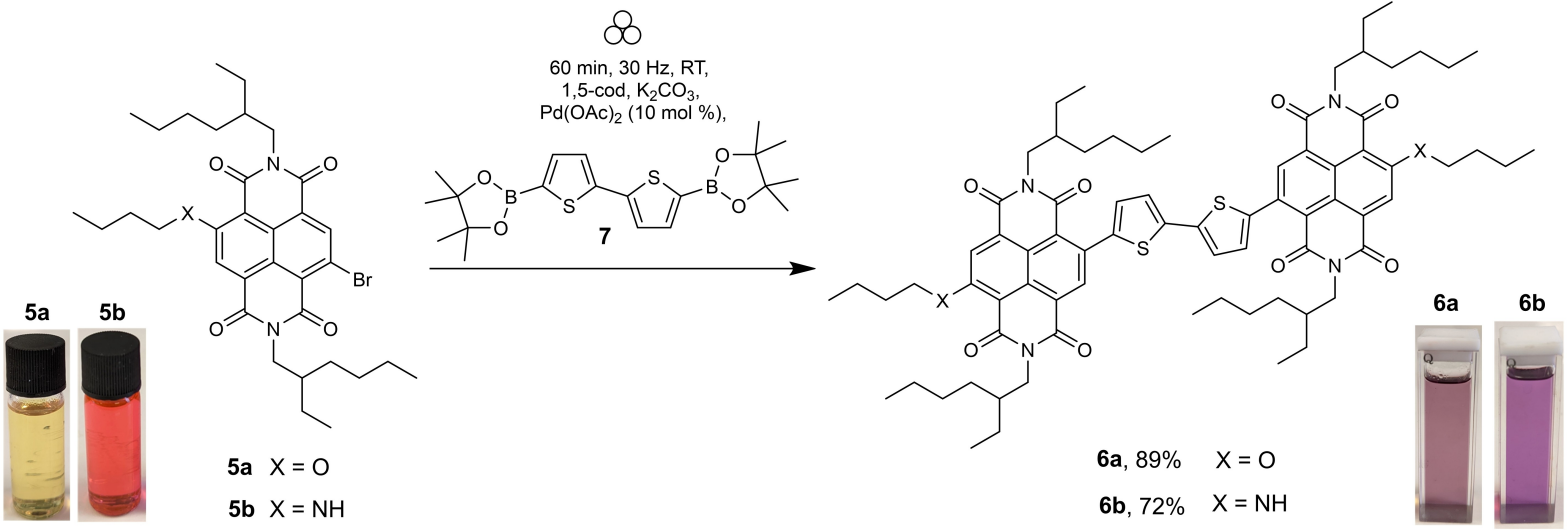
Synthesis of the molecular electron acceptor species **6 a** and **6 b**.

Small‐molecule^[51]^ and polymeric^[52]^ structures containing c‐NDIs have been shown to perform well as the electron accepting component in OPVCs. As a final test of this VBM methodology, we have carried out Suzuki coupling on mono(brominated) NDIs **5 a** and **5 b** to produce two dimeric c‐NDIs (**6 a** and **6 b**; Scheme 4). Each of these have been demonstrated to work as electron acceptor components in functioning OPVCs.^[51b]^ Using the conditions established above (Figure 2), double addition of either **5 a** or **5 b** to the bis(pinacol ester) **7** gave the target species in just 1 h in air and without any exclusion of moisture (89 and 72 % yield, respectively), comparable to the reported conditions for their solution‐state synthesis: 24 h, degassed toluene, at reflux (85 % (**6 a**) and 80 % (**6 b**)).^[51b]^

## Conclusions

In conclusion, we have highlighted the synthesis of over 20 c‐NDI structures by using Suzuki, Sonogashira without the addition of an external copper source and Buchwald–Hartwig reactions under VBM conditions. Although the purification of the products requires halogenated solvents, the reactions proceed without solvent, in less than 90 min, and are tolerant of air and moisture. The purified products contained low residual levels of Pd and Zr. These VBM conditions are in contrast to those reported for the typical solution‐state synthesis of this class of molecule, where dry and degassed solvents, strict anaerobic conditions and longer reaction times (often 18–24 h) are generally reported. In addition, the VBM conditions for the Sonogashira synthesis of c‐NDIs are the first reported that do not require the addition of copper. Except for the *para*‐dinitrophenyl‐substituted c‐NDI (**2 i**), the c‐NDIs exhibited ICT transitions with *λ*_max_ in the visible region, between 432 and 718 nm. The VBM synthesis of dimeric c‐NDIs (**6 a** and **6 b**), which are known to behave as electron acceptors in functioning OPVCs, paves the way for a more environmentally friendly, solvent‐free method for producing all‐polymer, high‐value end products.

## Associated Content

### Author Contributions

B.W.G. conceived and supervised the work assisted by J.S. and M.C.B. The synthesis and photophysical studies were carried out by L.A.P. for all the reported compounds. D.P.G. carried out initial synthetic studies, assisted with purification and grew the single crystals for X‐ray analysis. A.M., H.E., R.K.T. and A.J. conducted preliminary synthetic studies on the cross‐coupling reactions of NDIs. R.G.‐M. and C.A.I.G. conducted MS analysis of the samples. C.M.D. carried out ICP‐MS. S.J.C and G.J.T. conducted X‐ray analysis. B.W.G., L.A.P. and J.S. drafted the manuscript through discussion with all the authors who approved the final version of the manuscript.

## Conflict of interest

The authors declare no conflict of interest.

1

## Supporting information

As a service to our authors and readers, this journal provides supporting information supplied by the authors. Such materials are peer reviewed and may be re‐organized for online delivery, but are not copy‐edited or typeset. Technical support issues arising from supporting information (other than missing files) should be addressed to the authors.

Supporting Information

## Data Availability

The data that support the findings of this study are available in the supplementary material of this article at the following link: https://chemistry‐europe.onlinelibrary.wiley.com/doi/abs/10.1002/chem.202201444.
