# Supplementary material for: Solvent‐Free Synthesis of Core‐Functionalised Naphthalene Diimides by Using a Vibratory Ball Mill: Suzuki, Sonogashira and Buchwald–Hartwig Reactions
Source: Chemistry. 2022 Jul 13;28(49):e202201444. doi: 10.1002/chem.202201444 (PMC9544761; doi:10.1002/chem.202201444)
Supplement: Supplementary file 1 — Supporting Information [file CHEM-28-0-s001.pdf]

Chemistry–A European Journal

Supporting Information

Solvent-Free Synthesis of Core-Functionalised Naphthalene Diimides by Using a Vibratory Ball Mill: Suzuki, Sonogashira and Buchwald–Hartwig Reactions

Lydia A. Panther, Daniel P. Guest, Andrew McGown, Hugo Emerit, Raysa Khan Tareque, Arathy Jose, Chris M. Dadswell, Simon J. Coles, Graham J. Tizzard, Ramón González-Méndez, Charles A. I. Goodall, Mark C. Bagley, John Spencer, and Barnaby W. Greenland*

Table of Contents

S1.0 Solid State Optimization studies S3

S1.1 Suzuki Library Optimizations S3

S2.0 Experimental Details S4

S2.1 General Procedures S4

S2.2 Synthetic Procedures S5

S3.0 Characterization Data S24

S3.1 UV-Vis-NIR Spectra S24

S3.2 ^{1}H and ^{13}C NMR Spectra S27

S3.3 Mass Spectra S50

S3.4 Crystal Data S65

S4.0 References S86

S1.0 Solid State Optimization Studies

S1.1 Suzuki Library Optimizations

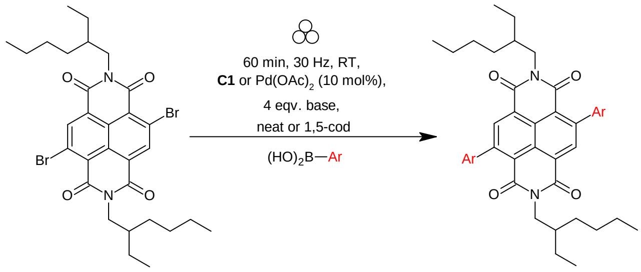

Scheme S1: Suzuki coupling of **1b** with aryl boronic acids to produce c-NDIs

Entry	[Pd] (10 mol %)	Boronic acid	Base	LAG	^{1}H NMR conversion (%) of c-NDI
1	$Pd(OAc)_2$	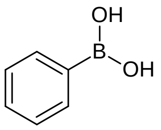	K_2CO_3	-	>98
2	$Pd(OAc)_2$		K_3PO_4	-	>98
3	$Pd(OAc)_2$	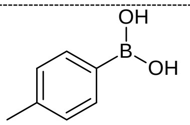	K_2CO_3	-	>98
4	$Pd(OAc)_2$		K_3PO_4	-	>98
5	$Pd(OAc)_2$	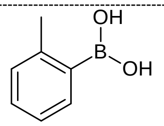	K_2CO_3	-	>98
6	$Pd(OAc)_2$		K_3PO_4	-	>98
7	$Pd(OAc)_2$	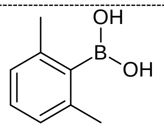	K_2CO_3	-	0
8	$Pd(OAc)_2$		K_3PO_4	-	0
9	$Pd(OAc)_2$		K_2CO_3	1,5-cod	23
10	$Pd(OAc)_2$	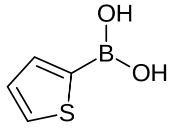	K_2CO_3	-	64
11	$Pd(OAc)_2$		K_3PO_4	-	24
12	X Phos Pd G3 (**C1**)		K_3PO_4	-	21
13	X Phos Pd G3 (**C1**)		K_2CO_3	-	42
14	$Pd(OAc)_2$		K_2CO_3	1,5-cod	>98
15	$Pd(OAc)_2$	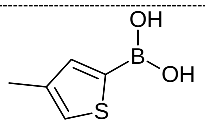	K_2CO_3	-	26
16	$Pd(OAc)_2$		K_2CO_3	1,5-cod	>98
17	$Pd(OAc)_2$		K_3PO_4	-	33
18	$Pd(OAc)_2$	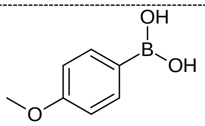	K_2CO_3	-	>98
19	$Pd(OAc)_2$		K_3PO_4	-	86
20	$Pd(OAc)_2$	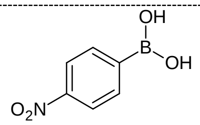	K_2CO_3	-	29
21	$Pd(OAc)_2$		K_2CO_3	1,5-cod	73
22	$Pd(OAc)_2$		K_3PO_4	-	42
23	$Pd(OAc)_2$		K_2CO_3	-	30

24	$Pd(OAc)_2$	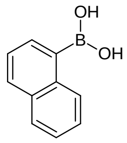	K_2CO_3	1,5-cod	66

Table S1: Optimization of Suzuki coupling; screening choice of catalyst, boronic acids, base and addition of LAG.

S2.0 Experimental Details

S2.1 General Procedures

Reagents were purchased from Merck, Fisher Scientific UK Ltd, Tokyo Chemical Industry UK Ltd or Fluorochem Ltd, and used without further purification. The ball milling reactions were carried out in a Retsch MM400 vibratory ball mill (VBM) operating at 30 Hz. Milling load is defined as the sum of the mass of the reactants per free volume in the jar.

Unless stated otherwise, purifications were performed *via* flash column chromatography on silica gel (RediSep® Rf Silica Gel Disposable Flash Columns, 40–60 micron) on Teledyne ISCO CombiFlash Lumen apparatus. Analytical thin layer chromatography (TLC) was performed on silica gel 60 F254 (Merck).

UV-Vis sample details: 1 mM stock solution of samples dissolved in $CHCl_3$ (Figure S3) and diluted to 0.2 mM for UV-vis measurements. UV-Vis measurements (300-900nm) were recorded at room temperature on a Perkin Elmer Lambda 25 double beam spectrometer. ^{1}H (600 MHz) and ^{13}C NMR (150 MHz) spectra were recorded on a Varian VNMRS 600 MHz spectrometer, at room temperature, using the residual protic solvent signal in the deuterated solvent for calibration (chloroform-*d* at 7.26 ppm or DMSO-d_6 at 2.50 ppm). Chemical shifts are reported in ppm. Spin multiplicities are reported as a singlet (s), doublet (d), triplet (t) or multiplet (m). with coupling constants (*J*) given in Hz, where applicable.

ESI mass spectra for compounds **2e, 2i** and **2j** were obtained by Dr. Ramon Gonzalez-Mendez using a Waters Xevo G2 Q-ToF HRMS(Wilmslow, UK) equipped with analytical flow ESI source. ESI experimental parameters were: capillary voltage 3.0 kV, sampling cone 35, extraction cone 4, source temperature 120 ºC and desolation gas 450ºC with a desolvation gas flow of 650L/h and no cone gas. MS conditions were MS1 in resolution mode between 100-1500 Da. Accurate mass data was obtained using MassLynx software. All accurate mass data was within ±5ppm from its theoretical value. ICP-MS analysis for metals was done by Dr. Christopher Dadswell using an Agilent 7500ce ICP-MS instrument with collision cell (He as collision gas) for Fe and standard mode (no collision gas) for Zr and Pd, using ^{72}Ge as internal standard. The following experimental parameters were used: a) plasma: RF power 1500 W, sampling depth 8.5mm, carrier gas 0.8L/min, make-up gas 0.11 L/min; b) quadrupole: mass range 1-250 amu, dwell time 100 msec, replicates 3, integration time 0.1sec/point. Calibration solutions were prepared for each element between 0 and 200ng/mL using certified reference standards (Fisher Scientific, UK)

High Resolution Mass Spectrometry (HRMS) data for all other samples were obtained by Dr Iain Goodall of the University of Greenwich Mass Spectrometry Service using a Waters (Wilmslow, UK) Synapt G2 Q-ToF HRMS. Chromatographic characterisation was provided by a Waters H-class UPLC pumping module with heated column and auto-sampler, running a reversed-phase gradient.

Solid State Structure determination: Crystals of compound **2g** were grown from pyridine and heptane using the slow diffusion method at room temperature. A single red rod-shaped crystal 0.10×0.03×0.03 mm^3 was selected and mounted on a MITIGEN holder in perfluoroether oil on a Rigaku 007HF diffractometer equipped with Arc)Sec VHF Varimax confocal mirrors and a UG2 goniometer and HyPix 6000HE detector. The crystal was kept at a steady T = 100(2) K during data collection. The structure was solved with the **ShelXT** 2018/2[1] structure solution program using the using dual methods solution method and by using **Olex2**[2] 1.5-alpha (Dolomanov et al., 2009) as the graphical interface. The model was refined with **ShelXL** 2018/3[3] using full matrix least squares minimisation on $\boldsymbol{F^2}$ minimisation.

Crystals of **4a** were grown from pyridine and heptane using the slow diffusion method at room temperature. A suitable single blue lath-shaped crystal of **4a** with dimensions 0.20 × 0.04 × 0.01 mm^3 was selected and mounted on a MITIGEN holder in perfluoroether oil on a Rigaku 007HF diffractometer with HF Varimax confocal mirrors, an AFC11 goniometer and HyPix 6000HE detector diffractometer. The crystal was kept at a steady T = 100.00(11) K during data collection. The structure was solved with the **ShelXT** 2018/2[1] solution program using dual methods and by using **Olex2**[2] 1.5-α as the graphical interface. The model was refined with **ShelXL** 2018/3[3] using full matrix least squares minimisation on $\boldsymbol{F}^2$.

S2.2 Synthetic Procedures

Synthesis of N,N'-bis(2-ethylhexyl)-2,6-dibromo-1,4,5,8-naphthalenetetracarboxylic acid, **1b** (known). [4,5]

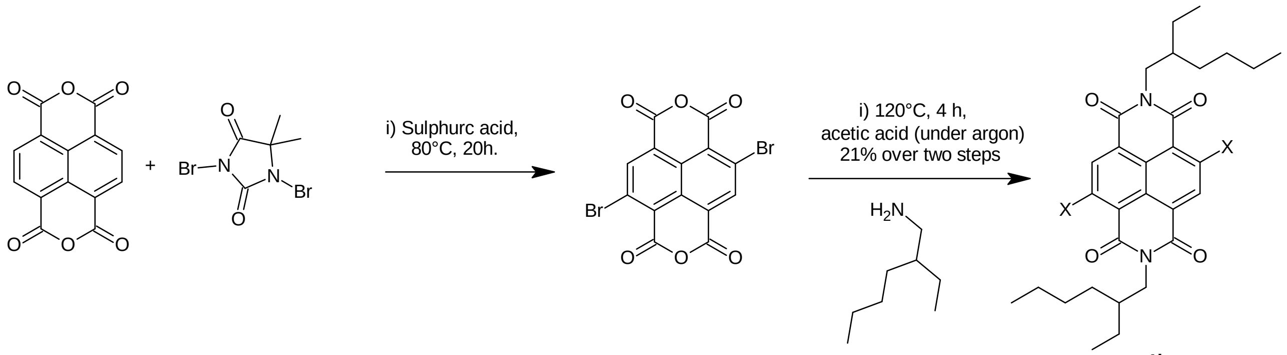

1

1a

1b = Br
1c = H

Scheme S2: Synthesis of **1b.**

Modified literature procedure by T. Govindaraju and co-workers was followed, details outlined below:

Synthesis of 2,6-dibromo-1,4,5,8-naphthalene tetracarboxylic acid **1a:**

Into a 500 mL round bottomed flask, napththalenetetracarboxylic acid **1** (30.02 g, 0.11 mol) was added to stirring conc. sulphuric acid (18 M, 300 mL) at room temperature (CAUTION, strong acid). 1.25 equivalents of 5,5-dimethyl-1,3-dibromohydantoin (40.02 g, 0.14 mol) were added in four portions over 1 hr. The reaction was stoppered and heated to 80°C for 20 h, where the mixture turned from beige to bright yellow with a red gas produced. The hot mixture was then poured directly onto 3L of crushed ice which melted to give a volume of approximately 1.5L of water whereupon the resulting yellow precipitate was collected *in-vacuo* on a low porosity sinter. The solid was washed with MeOH (300 mL) followed by water (300 mL) and dried in an oven at 40 °C under vacuum for 72 h to yield a mixture (47.4 g) of 2,6-dibromo-1,4,5,8-naphthalenetetracarboxylic acid (NDA) **1a** and NDA starting material **1**. 32:68 ratio of dibrominated NDA:NDA starting material was calculated from ^{1}H NMR data by integration of the signals at δ = 8.79 ppm : 8.71 ppm. The crude product was used in the next step of synthesis without purification due to its poor solubility.

Imidization of **1a** to N,N'-bis(2-ethylhexyl)-2,6-dibromo-1,4,5,8-naphthalenetetracarboxylic acid, **1b**:

Crude product **1a** (44.2 g) was added to acetic acid (300 mL) and stirred. 2-Ethylhexylamine (47.0 g, 0.41 mol) was added in four portions over 30 min at room temperature. The reaction was then heated to 120°C for 4 hr, where a colour change in solution from yellow to dark red was observed. Once the reaction was complete, confirmed by TLC (DCM:hexane v:v 70:30 R_f^{SM} = 0.00, R_f^{Prod} = 0.45), it was cooled and poured onto ice (2 L). The resulting bright orange precipitate was collected *in vacuo* on a low porosity sinter and washed with water (300 mL), MeOH (500 mL) where red washings were observed, and finally hexane (1 L) where orange washings were observed. The crude orange solid (44.32 g, 33:67 ratio of **1b**:**1c** by ^{1}H NMR) was then dissolved in a minimum amount of boiling $CHCl_3$ (*ca*. 500 mL) and was poured, while hot, into rapidly stirring cold hexane (2 L) to remove non-brominated diimide impurities and the pale orange crude solid was collected *in vacuo*. The precipitation procedure was repeated to yield yellow solid (25 g, 90:10 ratio of **1b**:**1c** by ^{1}H NMR). **1b** was isolated by a recrystallization over two weeks in DCM, where it was transferred to the

fridge after one week, to yield analytically pure product as a yellow fluffy needle-like crystalline solid (15.2 g, 21% over two steps). ^{1}H NMR (600 MHz, chloroform-*d*) δ 8.99 (s, 2H), 4.15 (m, 4H), 1.93 (m, 2H), 1.43 – 1.33 (m, 8H), 1.22-1.32 (m, 8H), 0.93 (m, 6H), 0.88 (m, 6H). ^{13}C NMR (151 MHz, chloroform-*d*) δ 161.6, 161.4, 139.5, 128.7, 128.1, 125.6, 124.4, 45.5, 38.1, 30.9, 28.9, 24.3, 23.4, 14.5, 10.9. HRMS-ESI: calcd for $C_{30}H_{37}Br_2N_2O_4$ [M+H]$^+$ 647.1042 found 647.1120. Characterization data in agreement with the literature.[6]

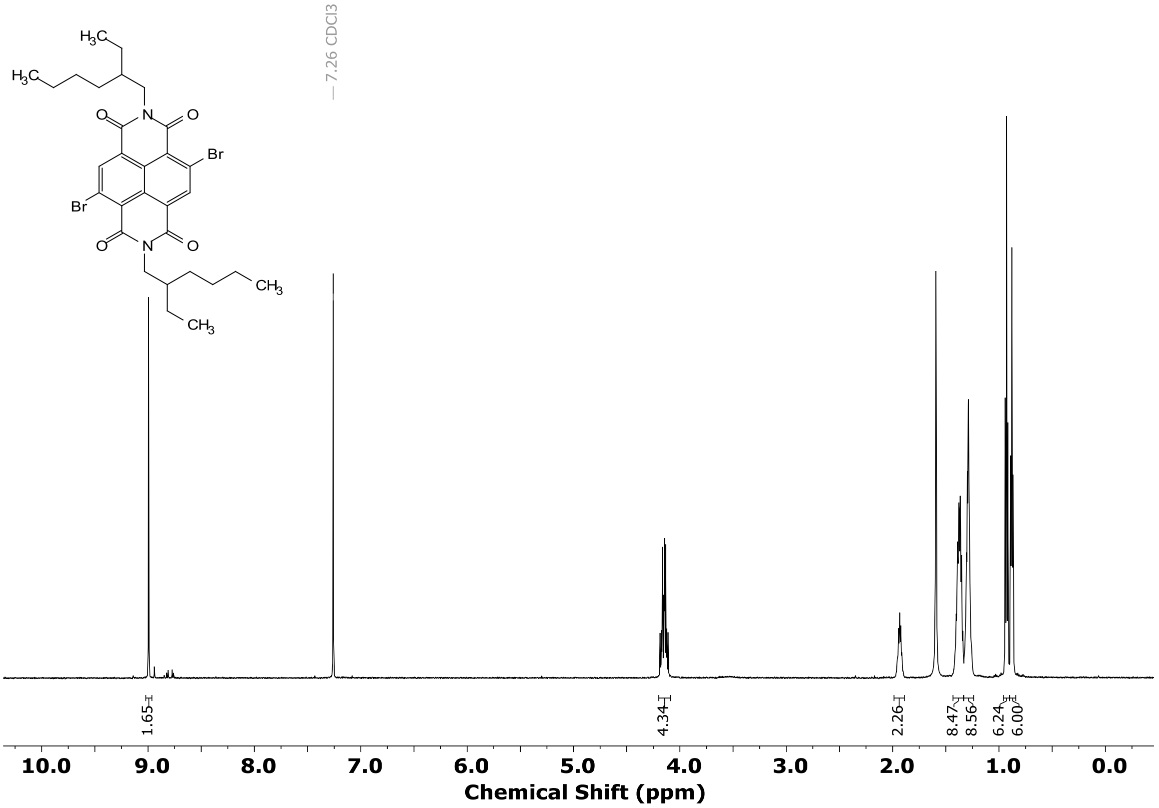

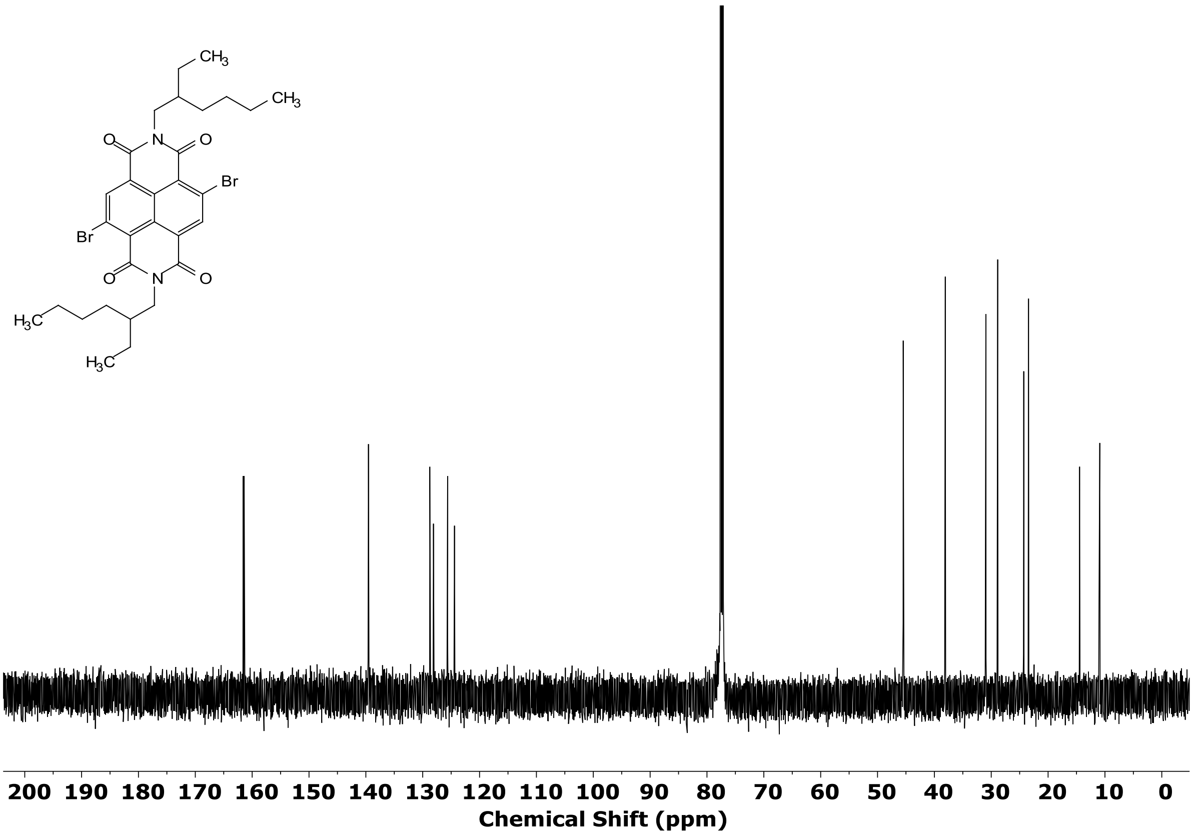

Figure S2: H (600 MHz) and ^{13}C (150 MHz) NMR spectra of **1b** ($CDCl_3$ at 298K).

General synthesis 1: Suzuki-Miyaura coupling

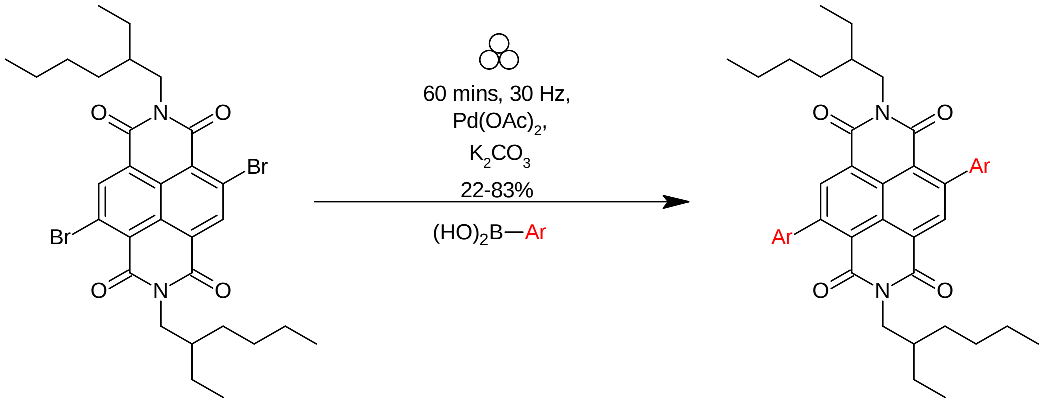

1b **2a-k**

General protocol 1. Into a 25mL ZrO_2 grinding jar were placed N,N'-bis(2-ethylhexyl)-2,6-dibromo-1,4,5,8-naphthalenetetracarboxylic acid **1b** (100 mg, 0.15 mmol), 3 equivalents of the requisite aryl boronic acid (0.46 mmol), 10 mol% palladium acetate (3.5 mg, 1.54 $x10^{-2}$ mmol), K_2CO_3 (85 mg, 0.62 mmol) and two 15 mm ZrO_2 balls. The total mass of the reagents was calculated so that milling load equals *ca*. 20 $mg.mL^{-1}$. The jar was then closed and subjected to grinding for 60 minutes in the VBM operated at 30 Hz. The jars were left to cool to room temperature and the resulting solid was dissolved in dichloromethane (25 mL) and passed through Celite, which was then washed with dichloromethane until the washings ran clear. Unless stated otherwise, the combined organic phases were washed with water (3 x 50 mL), dried over $MgSO_4$, filtered and the solvent was removed *in vacuo*. The crude material was then subjected to flash column chromatography (DCM:hexane) using a solvent gradient starting from 40% DCM up to 100% to obtain the c-NDI products**.**

Synthesis of (2,7-bis(2-ethylhexyl)-4,9-bis(4-methoxyphenyl)benzo[lmn][3,8]phenanthroline-1,3,6,8(2H,7H)-tetraone) **2a**

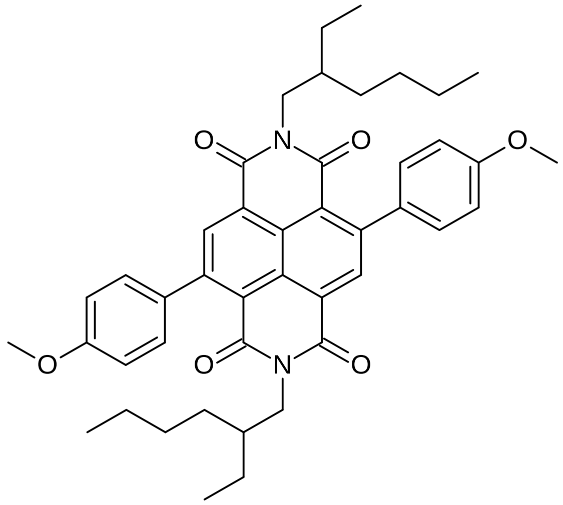

2a

The title compound was synthesized by following general protocol 1 using *p*-methoxybenzene boronic acid (70 mg, 0.46 mmol), **1b** (100 mg, 0.15 mmol), K_2CO_3 (85 mg, 0.62 mmol) and $Pd(OAc)_2$ (3.5 mg, 1.54 $x10^{-2}$ mmol). **2a** was isolated as a bright red solid after column chromatography (59 mg, 83%). (DCM:hexane2:1 (v/v) R_f = 0.45). ^{1}H NMR (600 MHz, chloroform-*d*) δ 8.79 (s, 2H), 7.67 (d, *J* = 8.4 Hz, 4H), 6.95 (d, *J* = 8.4 Hz, 4H), 4.13 - 4.18 (m, 4H), 3.87 (s, 6H), 1.98 - 1.95 (m, 2H), 1.36 (m, 16H), 1.03 - 0.85 (m, 12H). ^{13}C NMR (151 MHz, chloroform-*d*) δ 163.0, 162.9, 159.8, 147.3, 136.2, 132.5, 129.8, 127.2, 125.4, 122.6, 113.9, 55.3, 44.4,

37.6, 30.5, 28.5, 23.8, 23.1, 14.1, 10.6. IR(ATR); ν_{max} (cm^{-1}) 2933, 2859, 1704, 1664, 1438, 1246, 1035, 797. HRMS-ESI: calcd for $C_{44}H_{51}N_2O_6$ $[M+H]^+$ 703.3747; found 703.3740.

Synthesis of (2,7-bis(2-ethylhexyl)-4,9-diphenylbenzo[lmn][3,8]phenanthroline-1,3,6,8(2H,7H)-tetraone) **2b** (known)[7,8]

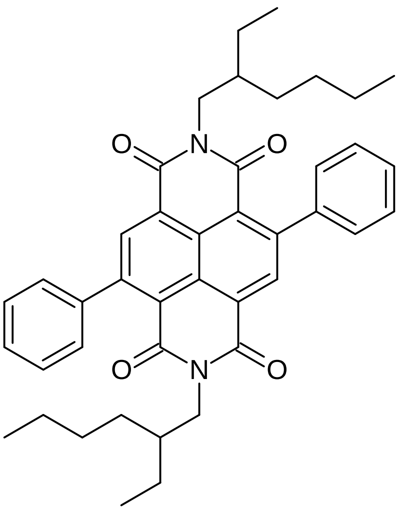

2b

The title compound was synthesized by following general protocol 1 using phenylboronic acid (56 mg, 0.46 mmol), **1b** (100 mg, 0.15 mmol), K_2CO_3 (85 mg, 0.62 mmol) and $Pd(OAc)_2$ (3.5 mg, 1.54 x10^{-2} mmol). **2b** was isolated as a bright yellow solid by column chromatography (85 mg, 83%). (DCM: hexane 60:40 (v/v) R_f = 0.4). ^{1}H NMR (600 MHz, chloroform-*d*) δ 8.66 (s, 2H), 7.51 (d, *J* = 7.0 Hz, 6H), 7.41 (d, *J* = 7.0 Hz, 4H), 4.11 – 3.96 (m, 4H), 1.87 (h, *J* = 6.6 Hz, 2H), 1.29 (m, 16H), 0.87 (m, 12H). ^{13}C NMR (151 MHz, chloroform-*d*) δ 163.3, 163.0, 148.0, 140.9, 136.2, 128.8, 128.6, 128.4, 127.6, 125.9, 123.3, 44.7, 38.0, 30.9, 28.8, 24.2, 23.4, 14.4, 10.9. IR(ATR); ν_{max} (cm^{-1}) 2957, 2854, 1706, 1667, 1573 1436, 1305, 1198, 696. HRMS-ESI: calcd for $C_{42}H_{47}N_2O_4$ $[M+H]^+$ 643.3536; found 643.3538.

Synthesis of (2,7-bis(2-ethylhexyl)-4,9-di-p-tolylbenzo[lmn][3,8]phenanthroline-1,3,6,8(2H,7H)-tetraone) **2c**

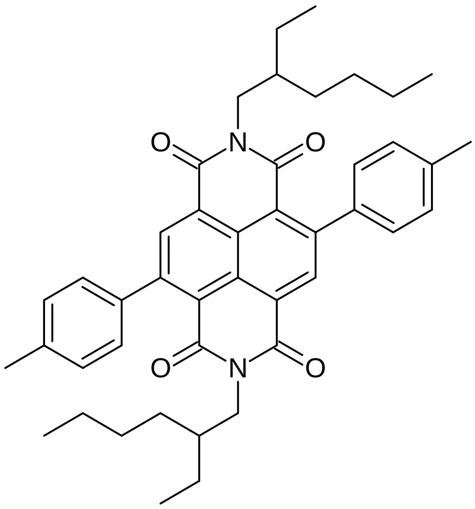

2c

The title compound was synthesized by following general protocol 1 using *p*-tolylboronic acid (64 mg, 0.46 mmol), **1b** (100 mg, 0.15 mmol), K_2CO_3 (85 mg, 0.62 mmol) and $Pd(OAc)_2$ (3.5 mg, 1.54 x10^{-2} mmol). **2c** isolated as a bright yellow solid by column chromatography (73 mg, 69%). (DCM: hexane 70:30 (v/v) R_f = 0.5). ^{1}H NMR (600 MHz, chloroform-*d*) δ 8.64 (s, 2H), 7.32 (s, 8H), 4.09 – 3.98 (m, 4H), 2.47 (s, 6H), 1.92 –

1.84 (m, 2H), 1.29 (m, 16H), 0.87 (m, 12H). ^{13}C NMR (151 MHz, chloroform-*d*) δ 163.0, 162.8, 147.7, 138.2, 137.5, 136.0, 129.2, 128.1, 127.2, 125.4, 122.8, 44.4, 37.6, 30.5, 28.5, 23.8, 23.1, 21.5, 14.1, 10.6. IR(ATR); ν_{max} (cm^{-1}) 2958, 2858, 1704, 1667, 1439, 1305, 1201, 796. HRMS-ESI: calcd for $C_{44}H_{51}N_2O_4$ [M+H]$^+$ 671.3849; found 671.3859.

Synthesis of (2,7-bis(2-ethylhexyl)-4,9-di-o-tolylbenzo[lmn][3,8]phenanthroline-1,3,6,8(2H,7H)-tetraone) **2d**

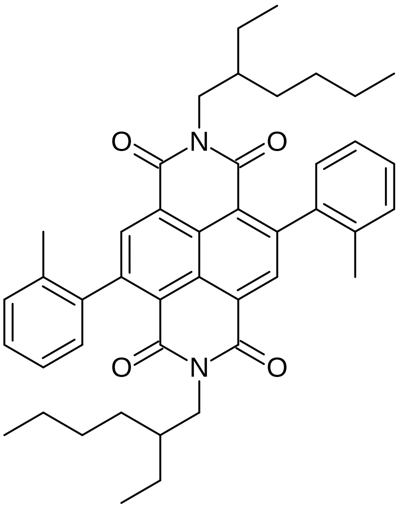

2d

The title compound was synthesized by following general protocol 1 using *o*-tolylboronic acid (63 mg, 0.46 mmol), **1b** (100 mg, 0.15 mmol), K_2CO_3 (85 mg, 0.62 mmol) and $Pd(OAc)_2$ (3.5 mg, 1.54 x10^{-2} mmol). **2d** was isolated as a pale-yellow solid by column chromatography (67 mg, 64%). (DCM:hexane70:30 (v/v) R_f = 0.5). ^{1}H NMR (600 MHz, chloroform-*d*) δ 8.58 (s, 2H), 7.47 – 7.29 (m, 6H), 7.13 (dt, J = 12.7, 6.5 Hz, 2H), 4.03 (s, 4H), 2.12 (s, 3H), 2.09 (s, 3H), 1.91- 1.83 (m, 2H), 1.35 – 1.16 (m, 16H), 0.90 – 0.80 (m, 12H). ^{13}C NMR (151 MHz, chloroform-*d*) δ 162.9, 162.4, 147.2, 140.5, 135.5, 134.2, 130.0, 128.2, 127.1, 127.0, 126.0, 125.7, 123.5, 44.4, 37.5, 30.5, 28.5, 23.8, 23.1, 20.0, 14.1, 10.6. IR(ATR); ν_{max} (cm^{-1}) 2956, 1705, 1665, 1435, 1308, 1203, 798. HRMS-ESI: $C_{44}H_{51}N_2O_4$ [M+H]$^+$ 671.3849; found 671.3853.

Synthesis of (4,9-bis(2,6-dimethylphenyl)-2,7-bis(2-ethylhexyl)benzo[lmn][3,8]phenanthroline-1,3,6,8(2H,7H)-tetraone) **2e**

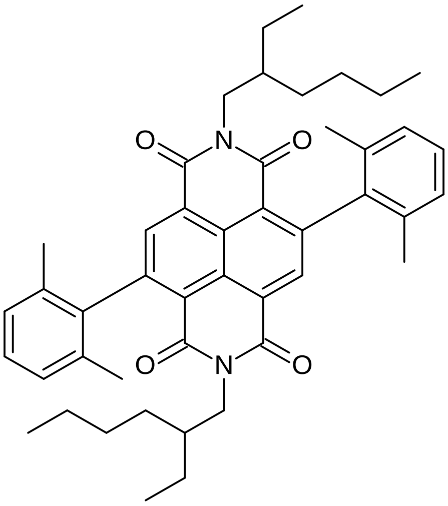

2e

The title compound was synthesized by following general protocol 1 using 2,6-dimethylphenylboronic acid (69 mg, 0.46 mmol), **1b** (100 mg, 0.15 mmol), K_2CO_3 (85 mg, 0.62 mmol) and $Pd(OAc)_2$ (3.5 mg, 1.54 x10^{-2} mmol). **2e** was isolated as a yellow solid by column chromatography (8 mg, 9%). (DCM:hexane70:30 (v/v) R_f

= 0.6). ^{1}H NMR (600 MHz, chloroform-*d*) δ 8.50 (s, 2H), 7.30 (t, *J* = 8.7, 6.3 Hz, 2H), 7.21 (d, *J* = 7.6 Hz, 4H), 4.03 (m, 4H), 1.98 (s, 12H), 1.85 (m, 2H), 1.35 – 1.13 (m, 16H), 0.92 – 0.76 (m, 12H). ^{13}C NMR (151 MHz, chloroform-*d*) δ 162.8, 162.3, 146.9, 140.1, 135.2, 133.5, 133.5, 127.8, 127.7, 127.1, 126.4, 123.6, 44.3, 37.5, 30.4, 28.4, 23.8, 23.1, 20.8, 20.7, 14.0, 10.6. IR(ATR); ν_{max} (cm^{-1}) HRMS-ESI: $C_{46}H_{55}N_2O_4$ $[M+H]^+$ 699.4162; found 699.4177.

Synthesis of (2,7-bis(2-ethylhexyl)-4,9-di(naphthalen-1-yl)benzo[lmn][3,8]phenanthroline-1,3,6,8(2H,7H)-tetraone) **2f**

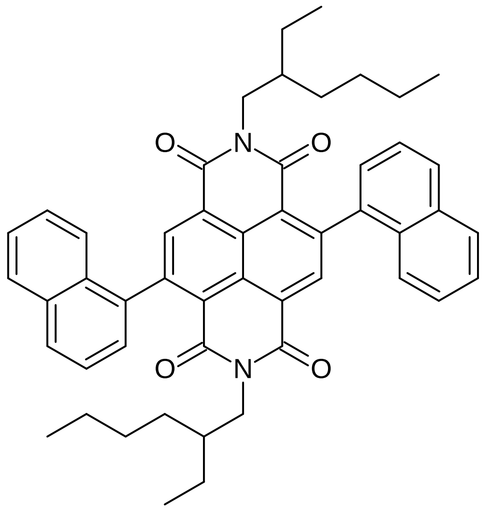

2f

The title compound was synthesized by following general protocol 1 using naphthalene-1-boronic acid pinacol ester (117 mg, 0.46 mmol), **1b** (100 mg, 0.15 mmol), K_2CO_3 (85 mg, 0.62 mmol) and $Pd(OAc)_2$ (3.5 mg, 1.54 x10^{-2} mmol). **2f** was isolated as an orangey yellow solid by column chromatography (25 mg, 22%). (EtOAc:hexane 60:40 (v/v) Rf = 0.45). ^{1}H NMR (600 MHz, chloroform-*d*) δ 8.75 (s, 2H), 8.00 (dd, J = 15.4, 8.2 Hz, 4H), 7.65 (dd, J = 7.2 Hz, 2H), 7.52 (dd, J = 7.2 Hz, 2H), 7.48 – 7.30 (m, 6H), 4.04 – 3.78 (m, 4H), 1.74 (tt, J = 13.1, 6.3 Hz, 2H), 1.47 – 1.01 (m, 16H), 0.79 (m, 12H).^{13}C NMR (151 MHz, chloroform-*d*) δ 162.8, 162.1, 146.1, 146.0, 138.7, 136.3, 133.4, 130.9, 128.8, 128.6, 127.3, 126.4, 126.0, 125.9, 125.5, 125.0, 124.6, 44.2, 44.2, 37.5, 37.4, 30.4, 30.3, 28.4, 28.3, 23.8, 23.7, 22.9, 22.9, 14.1, 14.0, 10.6. IR(ATR); ν_{max} (cm^{-1}) 2958, 2859, 1703, 1662, 1439, 1307, 772. HRMS-ESI: calcd for $C_{50}H_{51}N_2O_4$ $[M+H]^+$ 743.3849; found 743.3852.

Synthesis of (2,7-bis(2-ethylhexyl)-4,9-di(thiophen-2-yl)benzo[lmn][3,8]phenanthroline-1,3,6,8(2H,7H)-tetraone) **2g** (known)[9]

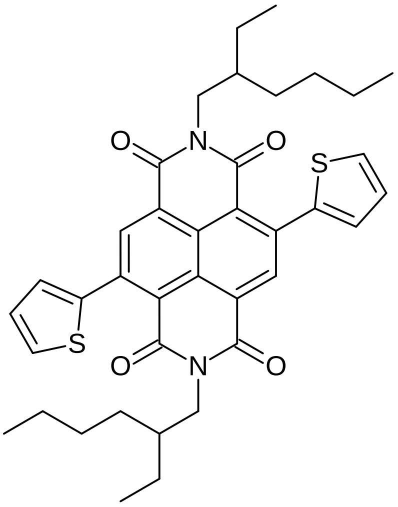

2g

The title compound was synthesized by following general protocol 1 using 2-Thienylboronic acid (64 mg, 0.46 mmol), **1b** (100 mg, 0.15 mmol), K_2CO_3 (85 mg, 0.62 mmol) and $Pd(OAc)_2$ (3.5 mg, 1.54 x10^{-2} mmol). **2g** was isolated as a bright red solid by column chromatography (68 mg, 67%). (DCM:hexane 80:20 (v/v) R_f = 0.6).^{1}H NMR (600 MHz, chloroform-*d*) δ 8.75 (s, 2H), 7.57 (d, *J* = 5.1 Hz, 2H), 7.30 (d, *J* = 3.6 Hz, 2H), 7.20 (dd, *J* = 5.1, 3.6 Hz, 2H), 4.14 – 4.02 (m, 4H), 1.88-1.92 (m, 2H), 1.41 – 1.19 (m, 16H), 0.84-0.92 (m, 12H). ^{13}C NMR (151 MHz, chloroform-*d*) δ 162.6, 162.4, 140.8, 140.2, 136.7, 128.3, 128.1, 127.5, 127.4, 125.4, 123.4, 44.6, 37.7, 30.6, 28.6, 23.9, 23.1, 14.1, 10.6. IR(ATR); $ν_{max}$ (cm^{-1}) 2959, 2859, 1706, 1660, 1574, 1441, 1307, 1195, 696. HRMS-ESI: calcd for $C_{38}H_{43}N_2O_4S_2$ $[M+H]^+$ 655.2664; found 655.2654.

Synthesis of (2,7-bis(2-ethylhexyl)-4,9-bis(4-methylthiophen-2-yl)benzo[lmn][3,8]phenanthroline-1,3,6,8(2H,7H)-tetraone) **2h**

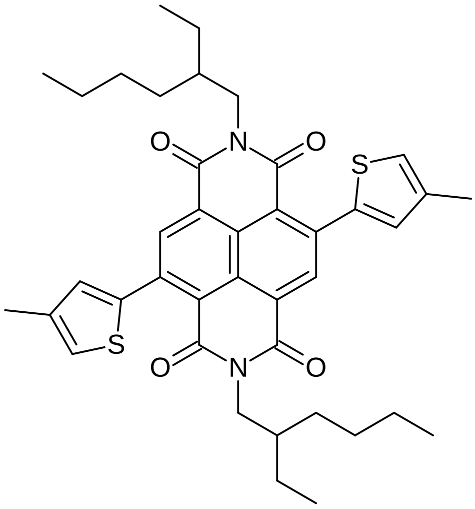

2h

The title compound was synthesized by following general protocol 1 using 3-methylthiophene-2-boronic acid (66 mg, 0.46 mmol), **1b** (100 mg, 0.15 mmol), K_2CO_3 (85 mg, 0.62 mmol) and $Pd(OAc)_2$ (3.5 mg, 1.54 x10^{-2} mmol). **2h** was isolated as a dark red solid by column chromatography (87 mg, 83%). (DCM:hexane 80:20 (v/v) R_f = 0.65). ^{1}H NMR (600 MHz, chloroform-*d*) δ 8.73 (s, 2H), 7.13 (d, *J* = 15.8 Hz, 4H), 4.09 (q, *J* = 9.7, 7.2 Hz, 4H), 2.37 (s, 6H), 1.91 (d, *J* = 10.3 Hz, 2H), 1.47 – 1.20 (m, 16H), 1.02 – 0.74 (m, 12H). ^{13}C NMR (151 MHz, chloroform-*d*) δ 163.0, 162.8, 141.0, 140.7, 138.4, 136.9, 130.9, 127.7, 125.7, 124.1, 123.4, 44.9, 38.0, 30.9, 28.9, 24.2, 23.4, 16.2, 14.4, 10.9. IR(ATR); $ν_{max}$ (cm^{-1}) 2958, 2859, 1671, 1662, 1445, 1307, 1193, 795. HRMS-ESI: calcd for $C_{40}H_{47}N_2O_4S_2$ $[M+H]^+$ 683.2977; found 683.2972.

Synthesis of (2,7-bis(2-ethylhexyl)-4,9-bis(4-nitrophenyl)benzo[lmn][3,8]phenanthroline-1,3,6,8(2H,7H)-tetraone) **2i**

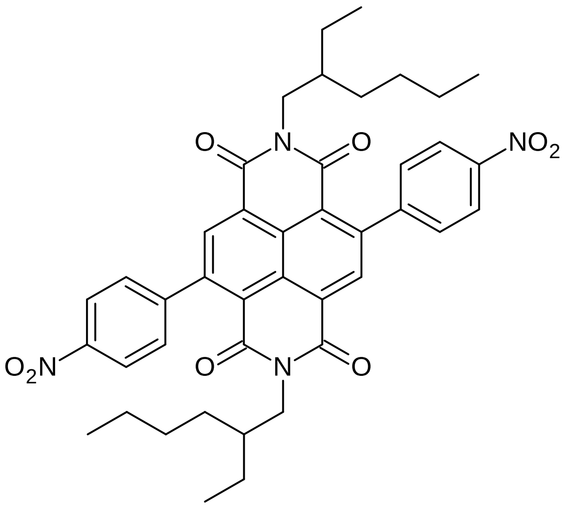

2i

The title compound was synthesized by following general protocol 1 using *p*-nitrobenzene boronic acid (77 mg, 0.46 mmol), **1b** (100 mg, 0.15 mmol), K_2CO_3 (85 mg, 0.62 mmol) and $Pd(OAc)_2$ (3.5 mg, 1.54 x10^{-2} mmol). **2i** was isolated as a sandy yellow solid by column chromatography (49 mg, 43%).(DCM:hexane2:1 (v/v) R_f = 0.35). ^{1}H NMR (600 MHz, chloroform-*d*) δ 8.62 (s, 2H), 8.39 (d, J = 8.5 Hz, 4H), 7.55 (d, J = 8.5 Hz, 4H), 4.08 – 3.94 (m, 4H), 1.83 (hept, *J* = 6.4 Hz, 2H), 1.40 – 1.14 (m, 16H), 1.03-0.75 (m, 12H). ^{13}C NMR (151 MHz, chloroform-*d*) δ 162.3, 162.3, 147.7, 147.0, 145.5, 134.9, 129.0, 127.4, 126.0, 123.8, 123.3, 44.7, 37.7, 30.5, 28.4, 23.8, 23.1, 14.1, 10.6. IR(ATR); ν_{max} (cm^{-1}) 2958, 2859, 1708, 1662, 1511, 1340, 1198, 848. HRMS-ESI: calcd for $C_{42}H_{45}N_4O_8$ $[M+H]^+$ 733.3237; found 733.3243.

Synthesis of (4-bromo-2,7-bis(2-ethylhexyl)-9-vinylbenzo[lmn][3,8]phenanthroline-1,3,6,8(2H,7H)-tetraone) **2j**

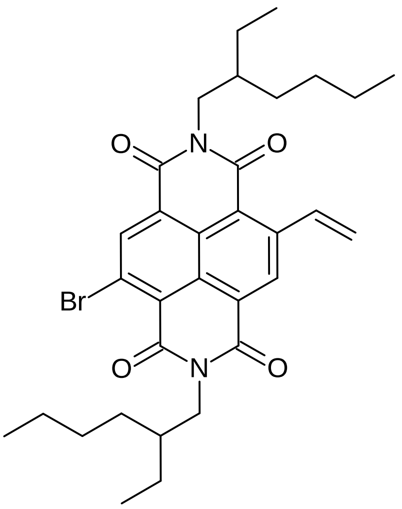

2j

The title compound was synthesized by following general protocol 1 using vinyl boronic pinacol ester (71 mg, 0.46 mmol), **1b** (100 mg, 0.15 mmol), K_2CO_3 (85 mg, 0.62 mmol) and $Pd(OAc)_2$ (3.5 mg, 1.54 x10^{-2} mmol). **2j** was isolated as a red solid product (16 mg, 5.2%) and isolated using the procedure below: Upon completion of ball milling, the vessels were cooled to room temperature before the contents were dissolved

in a mixture of dichloromethane and methanol (18 ml, 2:1). This suspension was filtered through a celite plug and the resulting filtrate was concentrated to yield a deep red, viscous semi solid. The red solid was dissolved in ethyl acetate (40 ml) and stirred for 10 minutes leading to the formation of an insoluble brown precipitate which was removed by filtration. The filtrate was concentrated and purified by column chromatography (16 mg, 5.2 %.) ^{1}H NMR (600 MHz, chloroform-*d*) δ 8.84 (s, 1H), 8.32 (s, 1H), 8.08 (dd, J = 17.5, 11.0 Hz, 1H), 5.98 (d, J = 17.5 Hz, 1H), 5.72 (d, J = 11.0 Hz, 1H), 4.19 – 4.01 (m, 4H), 1.92 (m, 2H), 1.48 – 1.13 (m, 16H), 0.95 – 0.84 (m, 12H). ^{13}C NMR (151 MHz, chloroform-*d*) δ 163.7, 163.7, 162.9, 162.4, 141.1, 135.9, 132.7, 129.2, 127.1, 124.7, 123.9, 122.0, 121.8, 120.6, 105.4, 44.7, 44.1, 37.8, 37.8, 30.7, 30.6, 28.6, 28.5, 23.99, 23.97, 23.07, 23.02, 14.07, 10.59, 10.56. IR(ATR); ν_{max} (cm^{-1}) 2925, 2858, 1700, 1643, 1279, 799, 762, 729, 529. HRMS-ESI: calcd for $C_{32}H_{39}BrN_2O_4$ [M+Na]$^+$, 617.1991 [^{79}Br] and 619.1970 [^{81}Br]; found, 617.2279 and 619.2303.

General synthesis 2: Sonogashira coupling

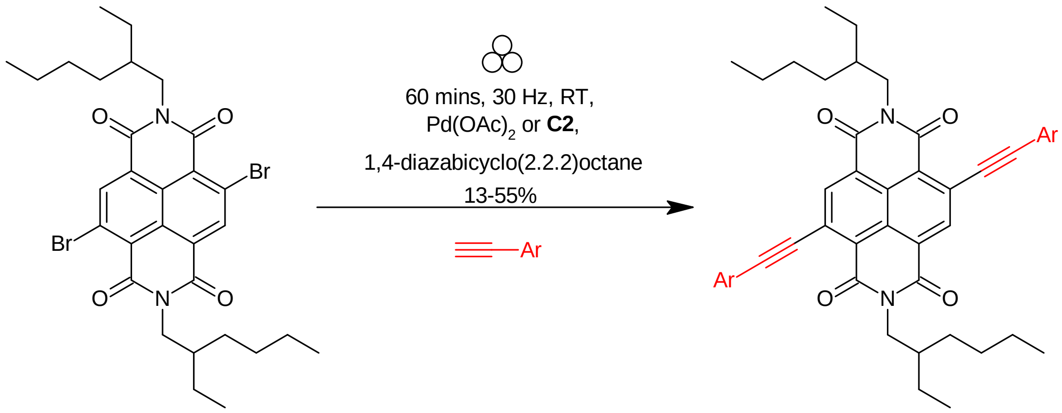

1b **3a-f**

General Protocol 2. Into a 25mL ZrO_2 grinding jar were placed N,N'-bis(2-ethylhexyl)-2,6-dibromo-1,4,5,8-naphthalenetetracarboxylic acid **1b** (100 mg, 0.15 mmol), 3 equivalents of the requisite aryl acetylene (0.46 mmol), 10 mol% palladium source (1.54 $x10^{-2}$ mmol), 4 equivalents of fresh 1,4-diazabicyclo[2.2.2]octane (69 mg, 0.62 mmol) and two 15 mm ZrO_2 balls. The total mass of the reagents was calculated so that milling load equals *ca*. 20 $mg.mL^{-1}$. The jar was then closed and subjected to grinding for 60 minutes in the VBM operated at 30 Hz. The jars were left to cool to room temperature and the resulting solid was dissolved in dichloromethane (25 mL) and passed through Celite, which was then washed with dichloromethane until the washings ran colourless. The combined organic layers were dried *in vacuo* and the crude material was then subjected to flash column chromatography (DCM:hexane) using a solvent gradient starting from 30% DCM up to 100% to obtain the c-NDI products**.**

Synthesis of (2,7-bis(2-ethylhexyl)-4,9-bis(phenylethynyl)benzo[lmn][3,8]phenanthroline-1,3,6,8(2H,7H)-tetraone) **3a**

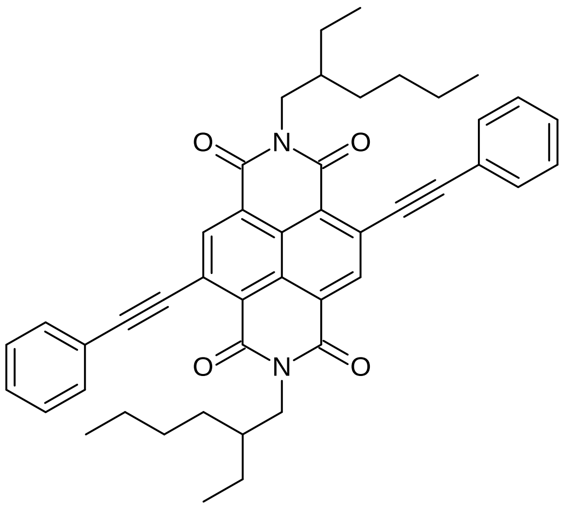

3a

The title compound was synthesized by following general protocol 2 using phenyl acetylene (47 mg, 0.46 mmol), **1b** (100 mg, 0.15 mmol), 1,4-diazabicyclo[2.2.2]octane (85 mg, 0.62 mmol) and $Pd(OAc)_2$ (3.5 mg, 1.54 $x10^{-2}$ mmol). **3a** was isolated as a bright orange solid by column chromatography (51 mg, 48%). (DCM:hexane2:1 (v/v) R_f = 0.5).^{1}H NMR (600 MHz, chloroform-*d*) δ 8.88 (s, 2H), 7.76 (dd, *J* = 6.7, 2.9 Hz, 4H), 7.44-7.46 (m, 6H), 4.18 (qd, *J* = 13.0, 7.3 Hz, 4H), 1.97-2.03 (m, 2H), 1.45 – 1.30 (m, 16H), 0.84-0.96 (m, 12H). ^{13}C NMR (151 MHz, chloroform-*d*) δ 162.5, 161.8, 137.3, 132.6, 129.9, 128.6, 127.2, 126.5, 125.3, 125.1,

122.5, 102.9, 89.6, 44.6, 37.7, 30.6, 28.5, 24.0, 23.1, 14.2, 10.6. IR(ATR); v_{max} (cm^{-1}) 2928, 2859, 2198 (C≡C), 1702, 1659, 1443, 1220, 768. HRMS-ESI: calcd for $C_{46}H_{47}N_2O_4$ $[M+H]^+$ 691.3536; found 691.3542.

Synthesis of (2,7-bis(2-ethylhexyl)-4,9-bis((4-methoxyphenyl)ethynyl)benzo[lmn][3,8]phenanthroline-1,3,6,8(2H,7H)-tetraone) **3b**

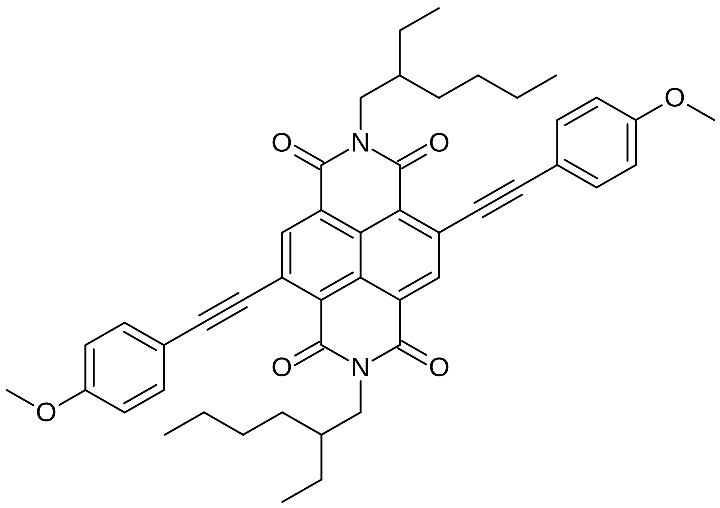

3b

The title compound was synthesized by following general protocol 2 using 1-ethynyl-4-methoxybenzene (70 mg, 0.46 mmol), **1b** (100 mg, 0.15 mmol), 1,4-diazabicyclo[2.2.2]octane (85 mg, 0.62 mmol) and **C2** (11 mg, 1.54 x10^{-2} mmol). **3b** was isolated as a rich purple solid by column chromatography (76 mg, 66%). (DCM: hexane 80:20 (v/v) R_f = 0.4). ^{1}H NMR (600 MHz, chloroform-*d*) δ 8.79 (s, 2H), 7.67 (d, *J* = 8.4 Hz, 4H), 6.95 (d, *J* = 8.4 Hz, 4H), 4.16 (qd, *J* = 13.0, 7.4 Hz, 4H), 3.87 (s, 6H), 1.98 (s, 2H), 1.29-1.45 (m, 16H), 1.03 – 0.85 (m, 12H). ^{13}C NMR (151 MHz, chloroform-*d*) δ 162.5, 161.9, 161.0, 137.1, 134.44 , 127.2, 126.3, 124.8, 124.6, 114.6, 114.3, 103.6, 89.4, 55.4, 44.5, 37.7, 30.6, 28.5, 24.0, 23.1, 14.2, 10.6. IR(ATR); v_{max} (cm^{-1}) 2958, 2859, 2188 (C≡C), 1702, 1660, 1510, 1248, 831. HRMS-ESI: calcd for $C_{48}H_{51}N_2O_6$ $[M+H]^+$ 751.3747; found 751.3740.

Synthesis of (4,9-bis((4-(dimethylamino)phenyl)ethynyl)-2,7-bis(2-ethylhexyl)benzo[lmn][3,8] phenanthroline-1,3,6,8(2H,7H)-tetraone) **3c**

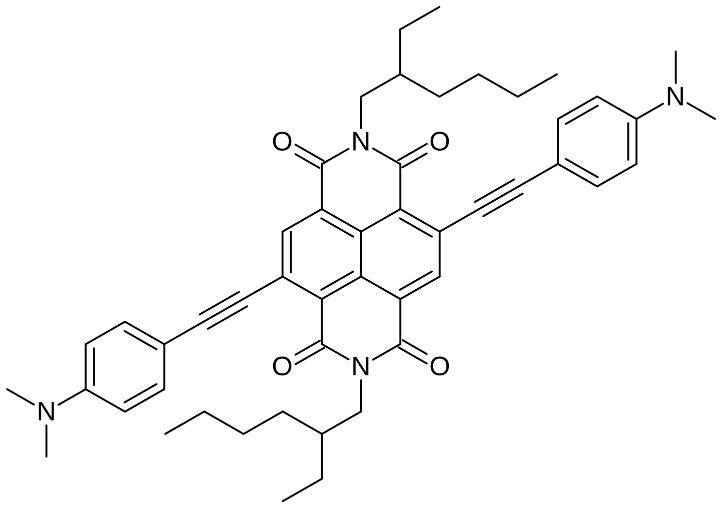

3c

The title compound was synthesized by following general protocol 2 using 4-ethynyl-N,N-dimethylaniline (71 mg, 0.46 mmol), **1b** (100 mg, 0.15 mmol), 1,4-diazabicyclo[2.2.2]octane (85 mg, 0.62 mmol) and $Pd(OAc)_2$ (3.5 mg, 1.54 x10^{-2} mmol). **3c** was isolated as a dark blue solid by column chromatography (68 mg, 55%). (DCM:hexane70:30 (v/v) R_f = 0.2). ^{1}H NMR (600 MHz, chloroform-*d*) δ 8.75 (s, 2H), 7.56 (d, *J* = 9.0 Hz, 4H),

6.66 (d, *J* = 9.0 Hz, 4H), 4.17 (qd, *J* = 13.0, 7.3 Hz, 4H), 3.05 (s, 12H), 2.01 (dq, *J* = 13.2, 6.7, 5.4 Hz, 2H), 1.49 – 1.21 (m, 16H), 0.86 – 0.97 (m, 12H). ^{13}C NMR (151 MHz, chloroform-*d*) δ 162.8, 162.0, 150.8, 136.9, 134.0, 127.0, 126.1, 124.4, 123.5, 111.4, 108.8, 105.5, 90.6, 44.31, 40.0, 37.7, 30.7, 29.7, 28.6, 24.0, 23.1, 14.2, 10.6. IR(ATR); ν_{max} (cm^{-1}) 2934, 2853, 2173 (C≡C), 1694, 1656, 1568, 1445, 1367, 1187, 813. HRMS-ESI: calcd for $C_{50}H_{57}N_4O_4$ [M+H]$^+$ 777.4380; found 777.4385.

Synthesis of (2,7-bis(2-ethylhexyl)-4,9-bis((4-(trifluoromethyl)phenyl)ethynyl)benzo[lmn][3,8] phenanthroline-1,3,6,8(2H,7H)-tetraone) **3d**

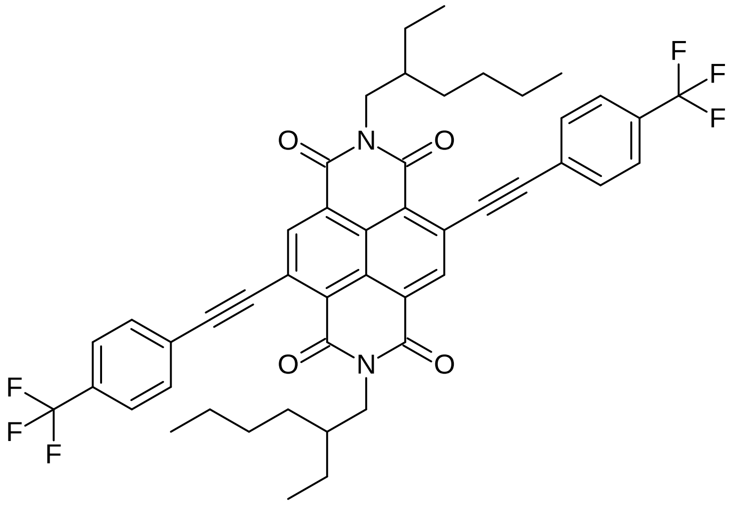

3d

The title compound was synthesized by following general protocol 2 using 4-ethynyl-α,α,α-trifluorotoluene (78 mg, 0.46 mmol), **1b** (100 mg, 0.15 mmol), 1,4-diazabicyclo[2.2.2]octane (85 mg, 0.62 mmol) and **C2** (11 mg, 1.54 x10^{-2} mmol). **3d** was isolated as a sandy yellow solid by column chromatography (30 mg, 24%). (DCM:hexane50:50 (v/v) R_f = 0.6).^{1}H NMR (600 MHz, chloroform-*d*) δ 8.79 (s, 2H), 7.83 (d, *J* = 8.0 Hz, 4H), 7.69 (d, *J* = 8.0 Hz, 4H), 4.14 (qd, *J* = 13.1, 7.3 Hz, 4H), 1.96 (hept, *J* = 6.9 Hz, 2H), 1.25 – 1.44 (m, 16H), 0.74 – 0.96 (m, 12H). ^{13}C NMR (151 MHz, chloroform-*d*) δ 162.1, 161.6, 137.2, 132.8, 132.5, 126.6, 125.2, 91.0, 44.6, 37.7, 30.6, 28.5, 24.0, 23.1, 14.1, 10.6. IR(ATR); ν_{max} (cm^{-1}) 2932, 2853, 2204 (C≡C), 1704, 1661, 1447, 1315, 1122, 845. HRMS-ESI: calcd for $C_{48}H_{45}F_6N_2O_4$ [M+H]$^+$ 827.3284; found 827.3256. Low solubility and C-F coupling meant not all signals in the ^{13}C NMR spectrum could be resolved.

Synthesis of (4,9-bis((4-acetylphenyl)ethynyl)-2,7-bis(2-ethylhexyl)benzo[lmn][3,8]phenanthroline-1,3,6,8(2H,7H)-tetraone) **3e**

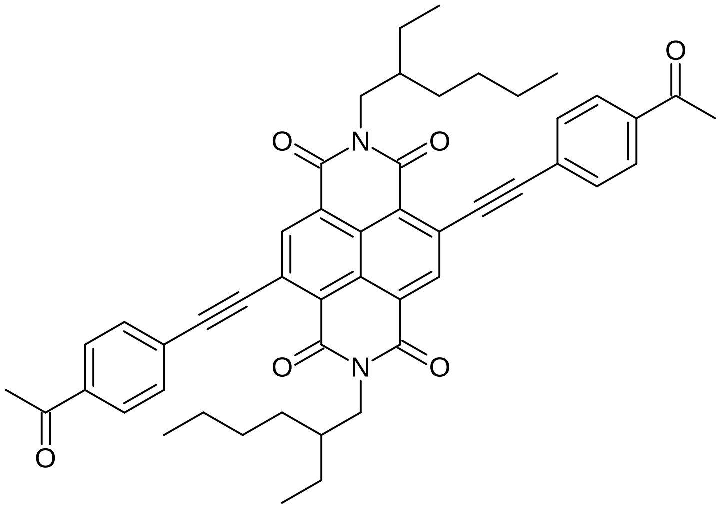

3e

The title compound was synthesized by following general protocol 2 using 4'-ethynylacetophenone (70 mg, 0.46 mmol), **1b** (100 mg, 0.15 mmol), 1,4-diazabicyclo[2.2.2]octane (85 mg, 0.62 mmol) and $Pd(OAc)_2$ (3.5 mg, 1.54 x10^{-2} mmol). **3e** was isolated as a dark orange solid by column chromatography (21 mg, 13%). (DCM 100% (v/v) R_f = 0.1).^{1}H NMR (600 MHz, chloroform-*d*) δ 8.79 (s, 2H), 7.99 (d, J = 8.3Hz, 4H), 7.78 (d, J = 8.3Hz, 4H), 4.15 (qd, *J* = 13.1, 7.4 Hz, 4H), 2.63 (s, 6H), 1.97 (h, *J* = 6.5 Hz, 2H), 1.22-1.44 (m, 16H), 0.84 – 0.96 (m, 12H). ^{13}C NMR (151 MHz, chloroform-*d*) δ 197.2, 162.1, 161.5, 137.3, 137.1, 132.7, 128.4, 127.0, 126.6, 126.54 , 125.7, 125.2, 101.3, 91.9, 44.6, 37.7, 30.6, 28.5, 26.7, 24.0, 23.1, 14.1, 10.6. IR(ATR); ν_{max} (cm^{-1}) 2930, 2858, 2202 (C≡C), 1686, 1665, 1573, 1261, 791. HRMS-ESI: calcd for $C_{50}H_{51}N_2O_6$ $[M+H]^+$ 775.3747; found 775.3732.

Synthesis of (2,7-bis(2-ethylhexyl)-4,9-bis(thiophen-2-ylethynyl)benzo[lmn][3,8]phenanthroline-1,3,6,8(2H,7H)-tetraone) **3f**

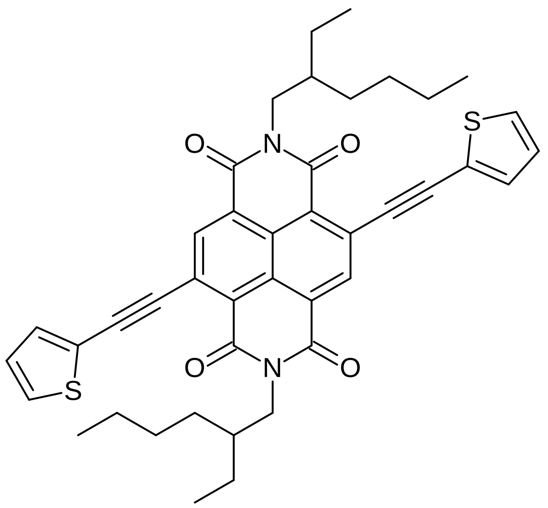

3f

The title compound was synthesized by following general protocol 2 using 2-ethynylthiophene (50 mg, 0.46 mmol), **1b** (100 mg, 0.15 mmol), 1,4-diazabicyclo[2.2.2]octane (85 mg, 0.62 mmol) and **C2** (11 mg, 1.54 x10^{-2} mmol). **3f** was isolated as a bright red solid by column chromatography (70 mg, 32%).(DCM:hexane70:30 (v/v) R_f = 0.3). ^{1}H NMR (600 MHz, chloroform-*d*) δ 8.80 (s, 2H), 7.56 (d, *J* = 5.1 Hz, 2H), 7.50 (d, *J* = 5.1 Hz, 2H), 7.12 (t, *J* = 4.4 Hz, 2H), 4.16 (m, 4H), 1.98 (m, 2H), 1.46 – 1.27 (m, 16H), 0.89 – 0.96 (m, 12H).^{13}C NMR (151 MHz, chloroform-*d*) δ 162.4, 161.8, 136.8, 134.8, 130.5, 127.7, 126.7, 126.4, 125.0, 124.7, 122.5, 96.5, 94.1, 44.6, 37.7, 30.6, 28.5 24.0, 23.1, 14.2, 10.6. IR(ATR); ν_{max} (cm^{-1}) 2957, 2852, 2186 (C≡C), 1701, 1652, 1445, 1195, 724. HRMS-ESI: calcd for $C_{42}H_{43}N_2O_4S_2$ $[M+H]^+$ 703.2664; found 703.2667.

General synthesis 3: Buchwald-Hartwig Aminations

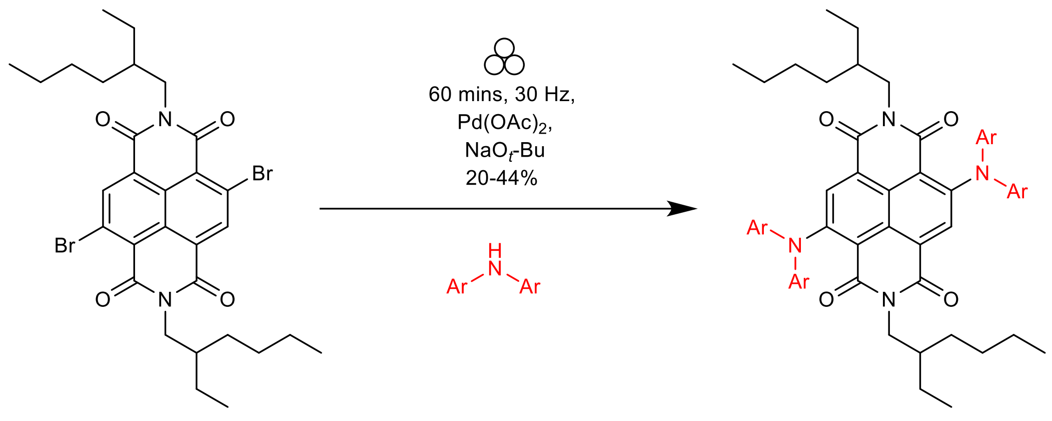

1b **4a-d**

General Protocol 3. Into a 25mL ZrO_2 grinding jar were placed N,N'-bis(2-ethylhexyl)-2,6-dibromo-1,4,5,8-naphthalenetetracarboxylic acid **1b** (100 mg, 0.15 mmol), 3 equivalents of the requisite aryl amine (0.46 mmol), 10 mol% palladium acetate (3.5 mg, 1.54 x10^{-2} mmol), 4 equivalents of fresh sodium *tert*-butoxide (60 mg, 0.62 mmol) and two 15 mm ZrO_2 balls. The total mass of the reagents was calculated so that milling load equals *ca*. 20 mg.mL^{-1}. The jar was then closed and subjected to grinding for 60 minutes in the VBM operated at 30 Hz. The jars were left to cool to room temperature and the resulting solid was dissolved in dichloromethane (25 mL) and passed through Celite, which was then washed with dichloromethane until the washings ran colourless. The combined organic layers were dried *in vacuo* and the crude material was then subjected to flash column chromatography (DCM:hexane) using a solvent gradient starting from 40% DCM up to 100% to obtain the c-NDI products**.**

Synthesis of (4,9-bis(diphenylamino)-2,7-bis(2-ethylhexyl)benzo[lmn][3,8]phenanthroline-1,3,6,8(2H,7H)-tetraone) **4a** (known)[8]

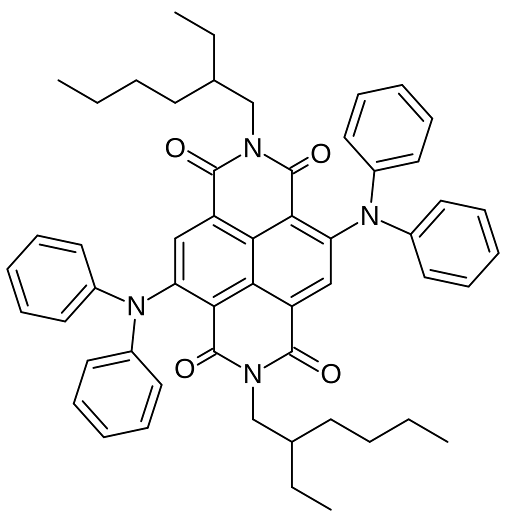

4a

The title compound was synthesized by following general protocol 3 using diphenylamine (79 mg, 0.46 mmol), **1b** (100 mg, 0.15 mmol), sodium tert-butoxide (59 mg, 0.62 mmol) and $Pd(OAc)_2$ (3.5 mg, 1.54 x10^{-2} mmol). **4a** was isolated as a dark turquoise solid by column chromatography (39 mg, 22%). (DCM:hexane 55:45 (v/v) R_f = 0.3). ^{1}H NMR (600 MHz chloroform-*d*) δ 8.43 (s, 2H), 7.24 (d, *J* = 7.6 Hz, 8H), 7.07 (d, 7.6z Hz, 12H), 3.77-3.90 (m, 4H), 1.15-1.18 (m 12H), 0.99-1.01 (m, 2H), 0.93 – 0.63 (m, 16H).^{13}C NMR (151 MHz, chloroform-*d*) δ 162.9, 160.3, 148.9, 147.1, 133.8, 129.4, 126.3, 126.1, 124.4, 123.9, 116.7, 43.9, 37.3, 30.4, 28.6, 23.5, 23.0, 14.1, 10.4. IR(ATR); ν_{max} (cm^{-1}) 2958, 2853, 1695, 1662, 1491, 1442, 1196, 691. HRMS-ESI:

calcd for $C_{54}H_{57}N_4O_4$ $[M+H]^+$ 825.4380; found 825.4390.

$C_{54}H_{56}N_4O_4$, M_r = 825.02, triclinic, *P*-1 (No. 2), a = 11.5685(9) Å, b = 14.3736(9) Å, c = 15.5702(10) Å, *a* = 115.595(6)°, *b* = 99.722(7)°, *g* = 97.130(6)°, *V* = 2244.2(3) Å^3, *T* = 100.00(11) K, *Z* = 2, *Z'* = 1, *m*(Cu K_a) = 0.606, 28811 reflections measured, 4997 unique (R_{int} = 0.0926) which were used in all calculations. The final wR_2 was 0.2433 (all data) and R_1 was 0.0775 (I≥2 *s*(I)).

Synthesis of (4,9-di(9H-carbazol-9-yl)-2,7-bis(2-ethylhexyl)benzo[lmn][3,8]phenanthroline-1,3,6,8(2H,7H)-tetraone) **4b** (known)[8]

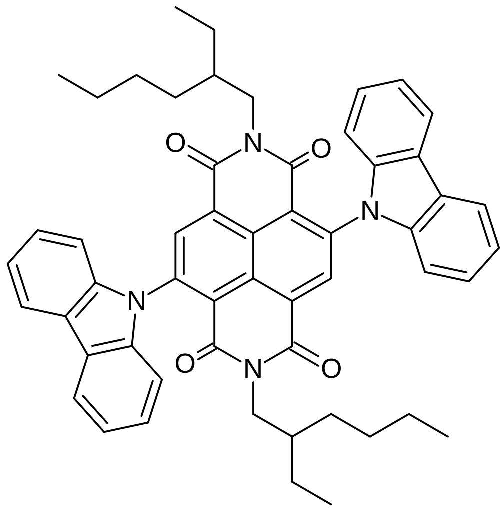

4b

The title compound was synthesized by following general protocol 3 using carbazole (78 mg, 0.46 mmol), **1b** (100 mg, 0.15 mmol), sodium tert-butoxide (59 mg, 0.62 mmol) and $Pd(OAc)_2$ (3.5 mg, 1.54 x10^{-2} mmol). **4b** was isolated as a royal blue solid by column chromatography (44 mg, 36%). (DCM:hexane2:1 (v/v) R_f = 0.6). ^{1}H NMR (600 MHz, chloroform-*d*) δ 9.03 (s, 2H), 8.20 (d, *J* = 7.5 Hz, 4H), 7.46 – 7.34 (m, 8H), 7.19 (d, *J* = 7.5, 4H), 4.05 – 3.87 (m, 4H), 1.82 – 1.69 (m, 2H), 1.36 – 1.11 (m, 16H), 0.96 – 0.73 (m, 12H). ^{13}C NMR (151 MHz, chloroform-*d*) δ 162.0, 160.1, 140.5, 139.9, 134.3, 127.9, 127.8, 126.4, 126.3, 124.7, 121.5, 120.9, 109.6, 44.5 37.5, 30.4, 28.5, 23.7, 23.0, 14.1, 10.5. IR(ATR); $ν_{max}$ (cm^{-1}) 2928, 2853, 1706, 1659, 1445, 1301, 743. HRMS-ESI: calcd for $C_{54}H_{53}N_4O_4$ $[M+H]^+$ 821.4067; found 821.4063.

Synthesis of (4,9-bis(bis(4-methoxyphenyl)amino)-2,7-bis(2-ethylhexyl)benzo[lmn][3,8]phenanthroline-1,3,6,8(2H,7H)-tetraone) **4c**

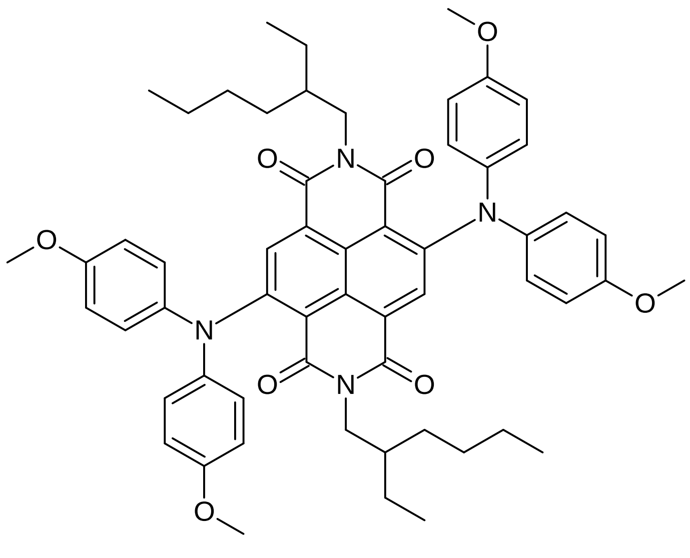

4c

The title compound was synthesized by following general protocol 3 using 4,4′-dimethoxydiphenylamine (106 mg, 0.46 mmol), **1b** (100 mg, 0.15 mmol), sodium tert-butoxide (59 mg, 0.62 mmol) and $Pd(OAc)_2$ (3.5 mg, 1.54 x10^{-2} mmol). **4c** was isolated as a dark blue solid by column chromatography (129 mg, 44%). (DCM:hexane 60:40 (v/v) R_f = 0.25). ^{1}H NMR (600 MHz, chloroform-*d*) δ 8.35 (s, 2H), 6.98 (d, *J* = 8.4 Hz, 8H), 6.78 (d, *J* = 8.4 Hz, 8H), 3.90-3.78 (m, 4H), 3.78 (s, 12H), 1.72 – 1.56 (m, 2H), 1.25 – 1.20 (m, 16H), 0.96 – 0.71 (m, 12H). ^{13}C NMR (151 MHz, chloroform-*d*) δ 163.1, 160.2, 156.5, 148.7, 140.8, 132.7, 125.8, 125.5, 125.2, 114.6, 55.3, 43.7, 37.3, 30.4, 28.5, 23.4, 22.9, 14.0, 10.1. IR(ATR); $ν_{max}$ (cm^{-1}) 2925, 2839, 1689, 1654, 1502, 1241, 833. HRMS-ESI: calcd for $C_{58}H_{65}N_4O_8$ $[M+H]^+$ 945.4802; found 945.4802.

Synthesis of (4,9-bis(ethyl(phenyl)amino)-2,7-bis(2-ethylhexyl)benzo[lmn][3,8]phenanthroline-1,3,6,8(2H,7H)-tetraone) **4d**

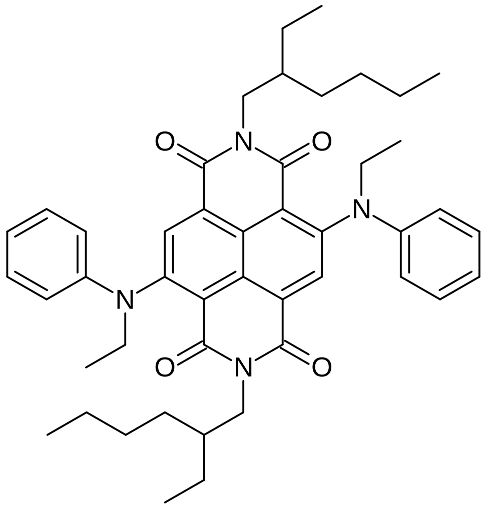

4d

The title compound was synthesized by following general protocol 3 using N-ethylaniline (60 mg, 0.46 mmol), **1b** (100 mg, 0.15 mmol), sodium tert-butoxide (59 mg, 0.62 mmol) and $Pd(OAc)_2$ (3.5 mg, 1.54 x10^{-2} mmol). **4d** was isolated as a dark blue solid by column chromatography (67 mg, 29%). (DCM:hexane 60:40 (v/v) R_f = 0.3).^{1}H NMR (600 MHz, chloroform-*d*) δ 8.55 (s, 2H), 7.21 (t, *J* = 7.8 Hz, 4H), 6.93 (d, *J* = 7.8 Hz, 6H), 4.04 (q, *J* = 7.3 Hz, 4H), 3.95 (m, 4H), 1.57 (s, 6H), 1.52 (s, 2H), 1.34 – 1.12 (m, 16H), 0.84 (m, 12H). ^{13}C NMR (151 MHz, chloroform-*d*) δ 163.1, 161.0, 149.1, 147.5, 131.9, 129.4, 126.1, 126.0, 122.0, 119.2, 48.2, 44.1, 37.4, 30.5, 28.6, 23.7, 23.1, 14.1, 13.8, 10.5. IR(ATR); $ν_{max}$ (cm^{-1}) 2958, 2857, 1692, 1652, 1445, 1255, 691. HRMS-ESI: calcd for $C_{46}H_{57}N_4O_4$ $[M+H]^+$ 729.4380; found 729.4368.

Synthesis of 4-bromo-9-butoxy-2,7-bis(2-ethylhexyl)benzo[lmn][3,8]phenanthroline-1,3,6,8(2H,7H)-tetraone **5a** (known)[10]

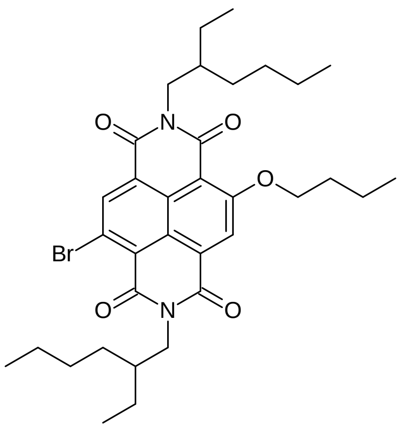

5a

Into a 250 mL round bottomed flask, **1b** (0.50 g, 7.71 mmol) was dissolved in dry DMF (125 mL). Pestle and mortar ground K_2CO_3 (1.07 g, 0.771 mmol) followed by *n*-butanol (0.29 mL, 0.385 mmol) were added to the vessel which was then heated to 80°C for 28 h. The mixture was cooled to room temperature and DCM (100 mL) was added to the reaction mixture. The organic phase was washed with water (3 x 75 mL) and the combined aqueous phases were washed with DCM (3 x 20 mL) washings were colourless. The organic phases were combined and dried with $MgSO_4$ before the solvent was removed *invacuo*. **5a** was isolated as a deep yellow solid by flash column chromatography (253 mg, 51%). (DCM:hexane 70:30 (v/v) R_f = 0.25). ^{1}H NMR (600 MHz, chloroform-*d*) δ 8.94 (d, *J* = 3.2 Hz, 1H), 8.51 (d, *J* = 3.2 Hz, 1H), 4.47 (q, *J* = 5.0, 3.8 Hz, 2H), 4.13 (m, 5H), 2.02 (t, *J* = 7.6 Hz, 2H), 1.94 (s, 2H), 1.62 (q, *J* = 7.7 Hz, 2H), 1.45 – 1.21 (m, 16H), 1.08 – 0.99 (m, 3H), 0.97 – 0.84 (m, 12H). ^{13}C NMR (151 MHz, chloroform-*d*) δ 162.32, 162.21, 161.96, 161.63, 161.13, 139.14, 128.45, 127.56, 125.07, 123.56, 123.47, 119.99, 111.23, 70.79, 53.58, 45.19, 44.51, 37.95, 37.78, 31.18, 30.79, 30.72, 28.71, 28.68, 24.12, 24.07, 23.23, 19.21, 14.25, 13.93, 10.75, 10.73. . HRMS-ESI: calcd for $C_{34}H_{46}BrN_2O_5[M+H]^+$ 642.2590; found 642.2595.

Synthesis of (4-bromo-9-(butylamino)-2,7-bis(2-ethylhexyl)benzo[lmn][3,8]phenanthroline-1,3,6,8(2H,7H)-tetraone **5b** (known)[10]

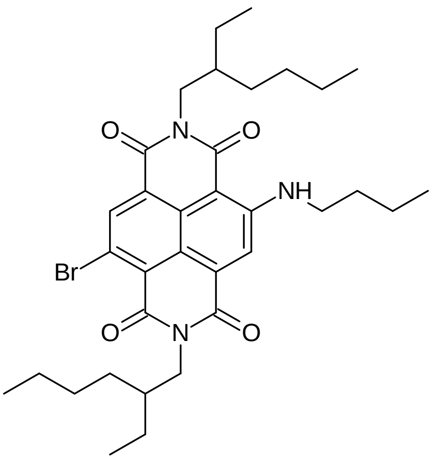

5b

Into a 500 mL round bottomed flask, **1b** (310 mg, 0.48 mmol) was dissolved $CHCl_3$ (240 mL) at room temperature. Butylamine (24 mL, excess) was added to the reaction mixture and an instantaneous colour change from yellow to bright pink was observed. The reaction was complete after 5 minutes, confirmed by TLC (DCM:hexane v:v 70:30 R_f^{SM} = 0.45, R_f^{Prod} = 0.60). DCM (200 mL) was added and the mixture was then washed with water (3 x 150 mL) and the organic layer was dried using $MgSO_4$. The solvent was removed *invacuo* and **5b** was isolated as a pink solid by flash column chromatography (293 mg, 96%). (DCM:hexane 90:10 (v/v) R_f = 0.65).^{1}H NMR (600 MHz, chloroform-*d*) δ 10.10 (s, 1H), 8.86 (d, *J* = 2.6 Hz, 1H), 8.28 (d, *J* = 2.9 Hz, 1H), 4.12 (tq, *J* = 13.5, 7.2 Hz, 3H), 3.58 (q, *J* = 6.9 Hz, 2H), 1.93 (s, 2H), 1.81 (q, *J* = 7.7 Hz, 2H), 1.54 (dd, *J* = 14.4, 7.0 Hz, 2H), 1.45 – 1.23 (m, 12H), 1.02 (t, *J* = 7.5 Hz, 2H), 0.91 (dt, *J* = 31.7, 7.4 Hz, 9H).^{13}C NMR (151 MHz, chloroform-*d*) δ 166.29, 162.54, 162.31, 161.80, 151.86, 138.39, 128.75, 123.44, 123.37, 121.51, 120.64, 120.28, 99.90, 44.95, 44.13, 43.06, 37.78, 37.75, 31.46, 30.64, 28.58, 23.98, 23.09, 20.22, 14.10, 13.78, 10.65, 10.60. HRMS-ESI: calcd for $C_{34}H_{47}BrN_3O_4$ $[M+H]^+$ 640.2750; found 640.2747

Synthesis of 9,9'-([2,2'-bithiophene]-5,5'-diyl)bis(4-butoxy-2,7-bis(2-ethylhexyl)benzo[lmn][3,8]phenanthroline-1,3,6,8(2H,7H)-tetraone) **6a**. (known)[10]

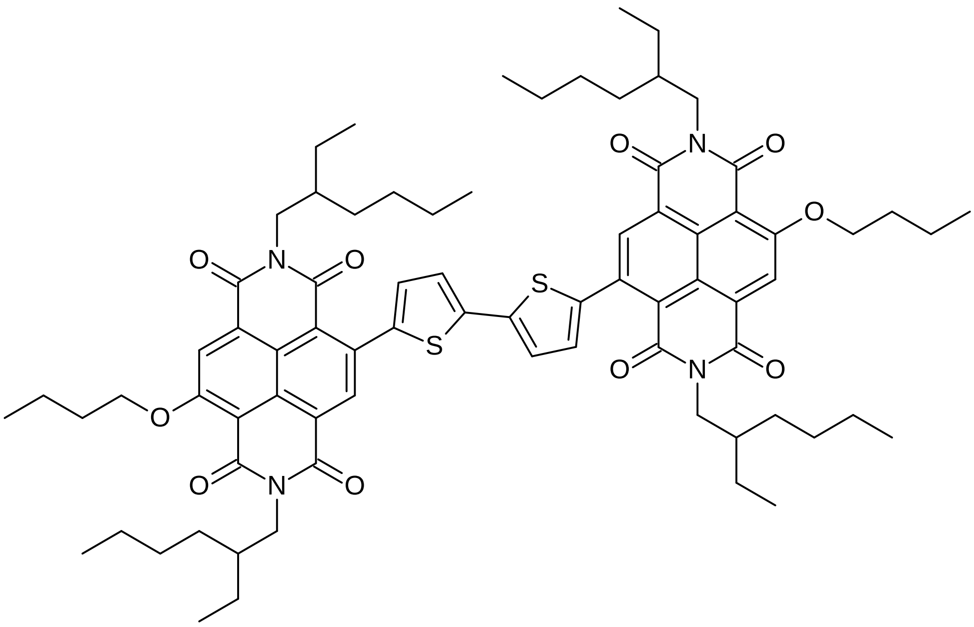

6a

Into a 25mL ZrO_2 grinding jar were placed 4-bromo-9-butoxy-2,7-bis(2-ethylhexyl)benzo[lmn][3,8]phenanthroline-1,3,6,8(2H,7H)-tetraone **5a** (59.61 mg, 0.093 mmol), 5,5'-bis(4,4,5,5-tetramethyl-1,3,2-dioxaborolan-2-yl)-2,2'-bithiophene **7** (19.61 mg, 0.047 mmol), 10 mol% palladium acetate (2.4 mg, 0.93 x10^{-2} mmol), K_2CO_3 (63 mg, 0.37 mmol), 1,5-cod (27 µL) and two 15 mm ZrO_2 balls. The jar was then closed and subjected to grinding for 60 minutes in the VBM operated at 30 Hz. The jar was left to cool to room temperature and the resulting solid was dissolved in dichloromethane (25 mL) and passed through Celite, which was then washed with dichloromethane until the washings ran clear. The combined organic phases were washed with water (3 x 50 mL), dried over $MgSO_4$, filtered and the solvent was removed *in vacuo*. **6a** was isolated as a green brown solid by flash column chromatography (53 mg, 89%). (DCM 100% R_f = 0.20). ^{1}H NMR (600 MHz, chloroform-*d*) δ 8.74 (s, 2H), 8.51 (s, 2H), 7.28 (s, 2H), 7.22 (d, *J* = 3.1 Hz, 2H), 4.49 (t, *J* = 6.9 Hz, 5H), 4.11 (tdd, *J* = 21.4, 13.1, 7.2 Hz, 10H), 2.04 (p, *J* = 7.3 Hz, 5H), 1.94 (dp, *J* = 19.6, 6.8 Hz, 6H), 1.64 (h, *J* = 7.6 Hz, 6H), 1.44 – 1.23 (m, 42H), 1.05 (t, *J* = 7.4 Hz, 8H), 0.90 (dt, *J* = 32.5, 7.1 Hz, 31H).^{13}C NMR (151 MHz, chloroform-*d*) δ 162.82, 162.71, 162.51, 161.90, 161.27, 140.37, 139.41, 136.73, 129.17, 128.78, 128.20, 124.48, 124.38, 122.96, 122.56, 119.62, 110.85, 70.59, 44.67, 44.27, 37.80, 37.66, 31.08, 30.65, 30.61, 28.58, 23.95, 23.92, 23.12, 23.10, 19.08, 14.12, 13.82, 10.64, 10.62. HRMS-ESI: calcd for $C_{76}H_{94}N_4O_{10}S_2$ $[M+H]^+$ 1287.6490; found 1287.6521

Characterization data in agreement with the literature.

Synthesis of 9,9'-([2,2'-bithiophene]-5,5'-diyl)bis(4-(butylamino)-2,7-bis(2-ethylhexyl)benzo[lmn][3,8]phenanthroline-1,3,6,8(2H,7H)-tetraone) **6b**. (known)[10]

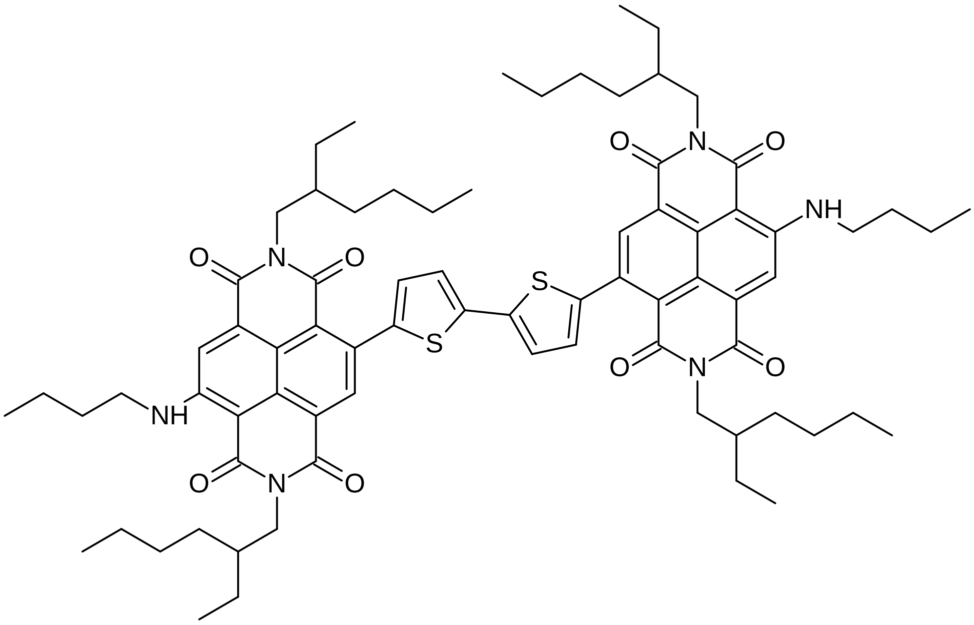

6b

Into a 25mL ZrO_2 grinding jar were placed (4-bromo-9-(butylamino)-2,7-bis(2-ethylhexyl)benzo[lmn][3,8]phenanthroline-1,3,6,8(2H,7H)-tetraone **5b** (100 mg, 0.15 mmol), 5,5'-bis(4,4,5,5-tetramethyl-1,3,2-dioxaborolan-2-yl)-2,2'-bithiophene **7** (32.45 mg, 0.075 mmol), 10 mol% palladium acetate (3.5 mg, 1.54 x10^{-2} mmol), K_2CO_3 (85 mg, 0.62 mmol), 1,5-cod (27 µL) and two 15 mm ZrO_2 balls. The total mass of the reagents was calculated so that milling load equals *ca*. 20 mg.mL^{-1}. The jar was then closed and subjected to grinding for 60 minutes in the VBM operated at 30 Hz. The jars were left to cool to room temperature and the resulting solid was dissolved in dichloromethane (25 mL) and passed through Celite and the solvent removed. The crude material and **5a** was isolated as a deep purple solid by flash column chromatography (73 mg, 72%). (DCM:hexane 95:5 (v/v) R_f = 0.40). ^{1}H NMR (600 MHz, chloroform-*d*) δ 10.10 (d, *J* = 5.9 Hz, 2H), 8.61 (s, 2H), 8.27 (s, 2H), 7.22 (s, 2H), 7.11 (s, 2H), 4.10 (ddd, *J* = 32.9, 16.1, 9.1 Hz, 9H), 3.61 (q, *J* = 6.8 Hz, 4H), 2.00 – 1.88 (m, 5H), 1.84 (p, *J* = 7.6 Hz, 5H), 1.68 – 1.49 (m, 9H), 1.46 – 1.20 (m, 38H), 1.04 (t, *J* = 7.4 Hz, 7H), 0.98 – 0.70 (m, 29H).^{13}C NMR (151 MHz, chloroform-*d*) δ 166.46, 163.08, 163.03, 162.76, 151.99, 140.85, 138.83, 136.24, 133.13, 129.24, 128.24, 128.12, 123.93, 123.54, 122.32, 120.47, 120.31, 99.79, 44.55, 44.00, 43.06, 37.77, 31.53, 30.68, 30.65, 28.61, 24.00, 23.92, 23.14, 23.11, 20.26, 14.12, 13.81, 10.69, 10.64. HRMS-ESI: calcd for $C_{76}H_{96}N_6O_8S_2$[M+H]$^+$ 1285.6809; found 1285.6862

Characterization data in agreement with the literature.

S3.0 Characterization Data

S3.1 UV-Vis Spectra

1b 3b 3f 2h 2a 2g 3e 3a 3d 2f 2b 2c 2d 2i 4c 4a 4d 4b 3c 6b 6a

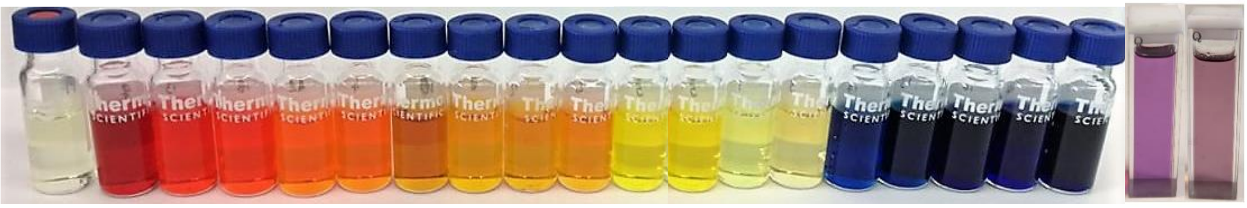

Figure S3: Photograph of 1mM solution of starting material **1b** (left) and cross coupled products **2a**-**6b** in chloroform.

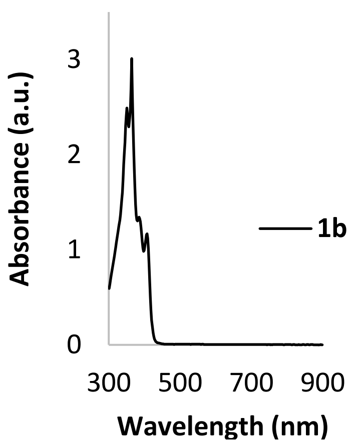

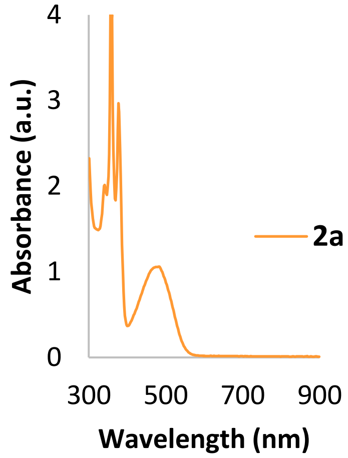

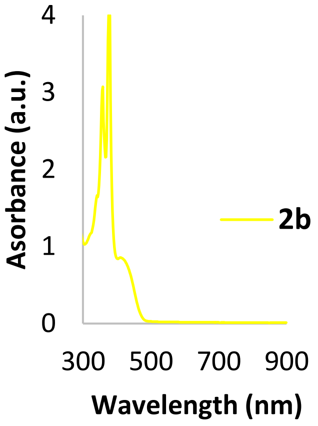

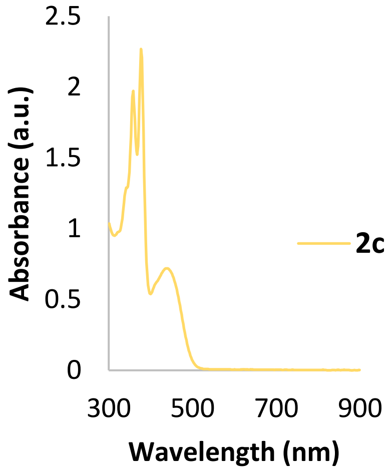

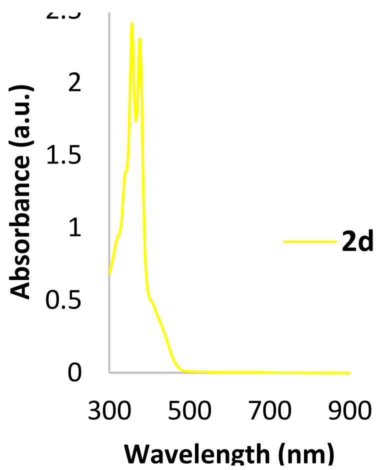

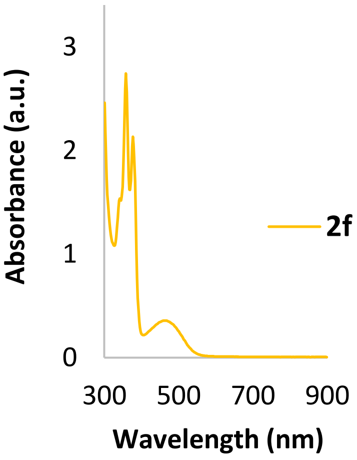

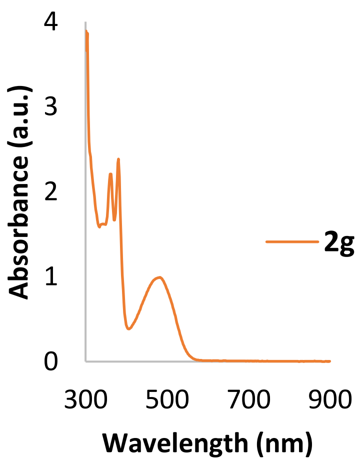

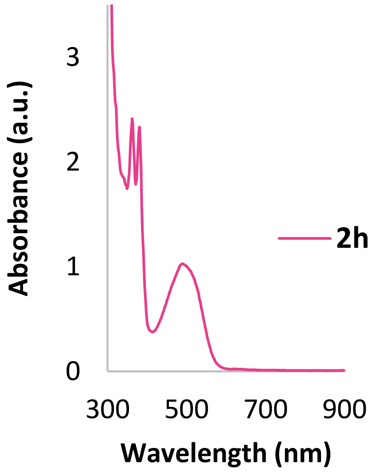

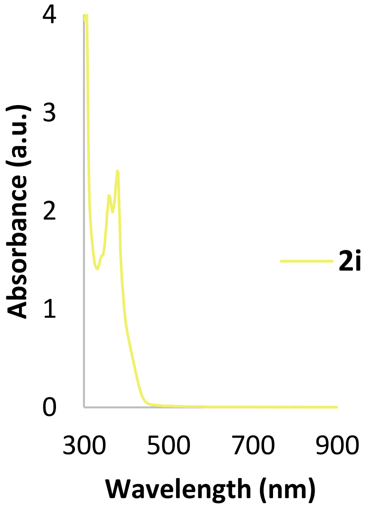

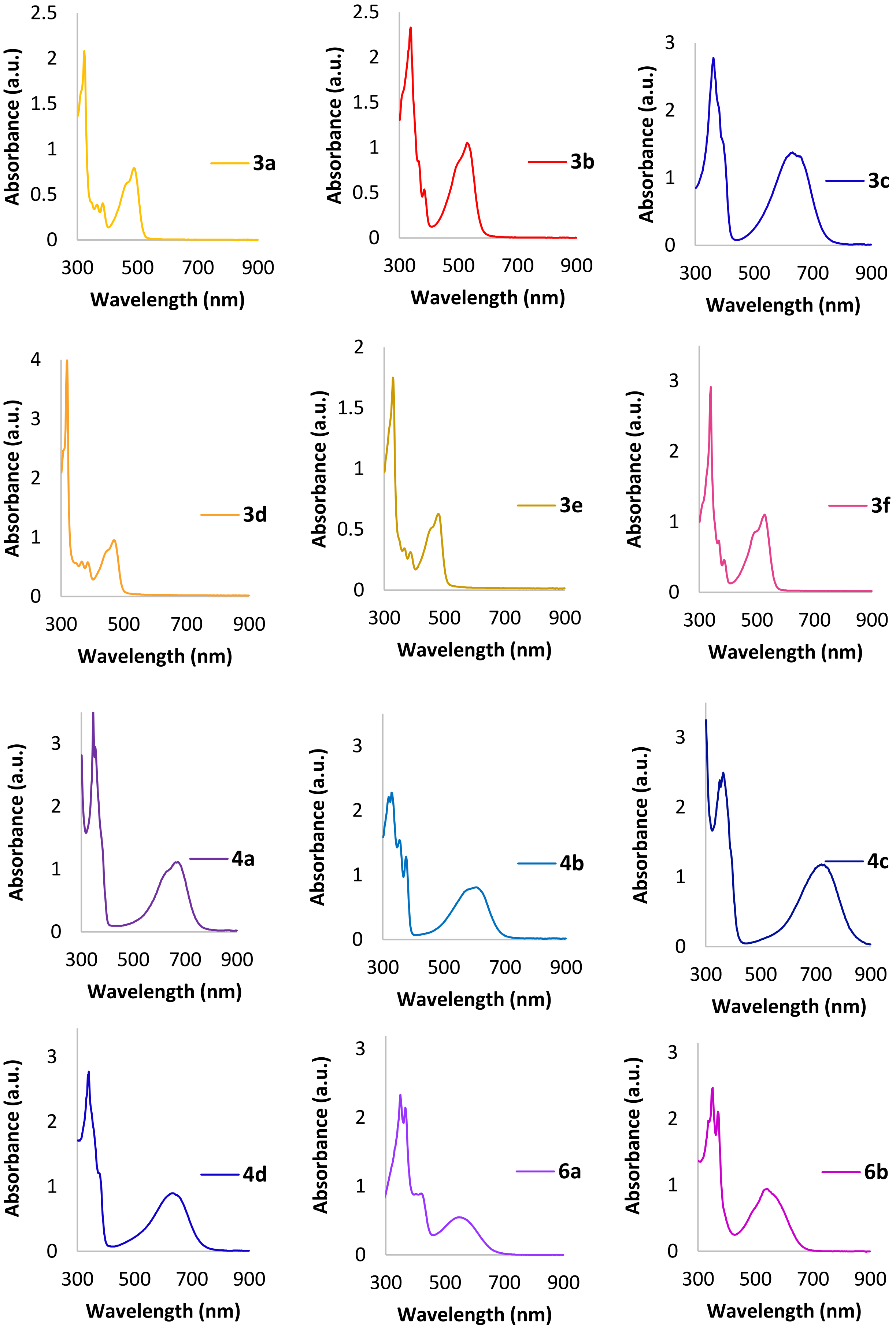

Figure S4: UV-vis spectra for synthesized c-NDI products **1b-6b** in chloroform at 2 x 10^{-5} M

Table S2: UV-Vis absorption characteristics of compounds 1d-4d

Compound	λ_{abs} /nm
**1b**	357, 364, 404
**2a**	303, 359, 379, 472
**2b**	357, 375, 432
**2c**	359, 377, 447
**2d**	357, 375, 434
**2e**	358, 414, 435
**2f**	303, 359, 375, 484
**2g**	305, 364, 379, 491
**2h**	310, 362, 379, 495
**2i**	303, 362, 377
**3a**	321, 370, 381, 493
**3b**	340, 374, 526
**3c**	364, 624, 646
**3d**	318, 374, 383, 476
**3e**	329, 372, 381, 485
**3f**	340, 360, 385, 532
**4a**	303, 346, 660
**4b**	333, 353, 379, 596
**4c**	301, 360, 718
**4d**	336, 374, 626
**6a**	351, 368, 424, 559
**6b**	349, 355, 542

S3.2 ^{1}H and ^{13}C NMR spectra of all c-NDIs

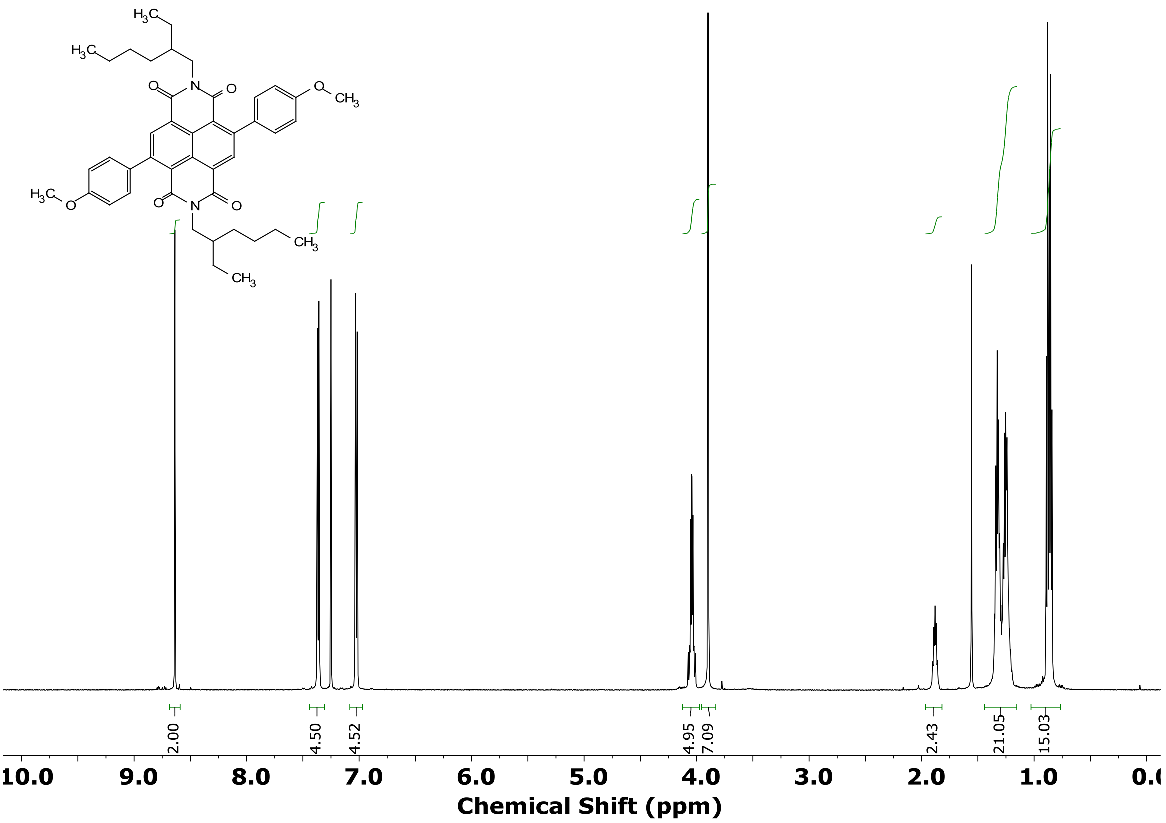

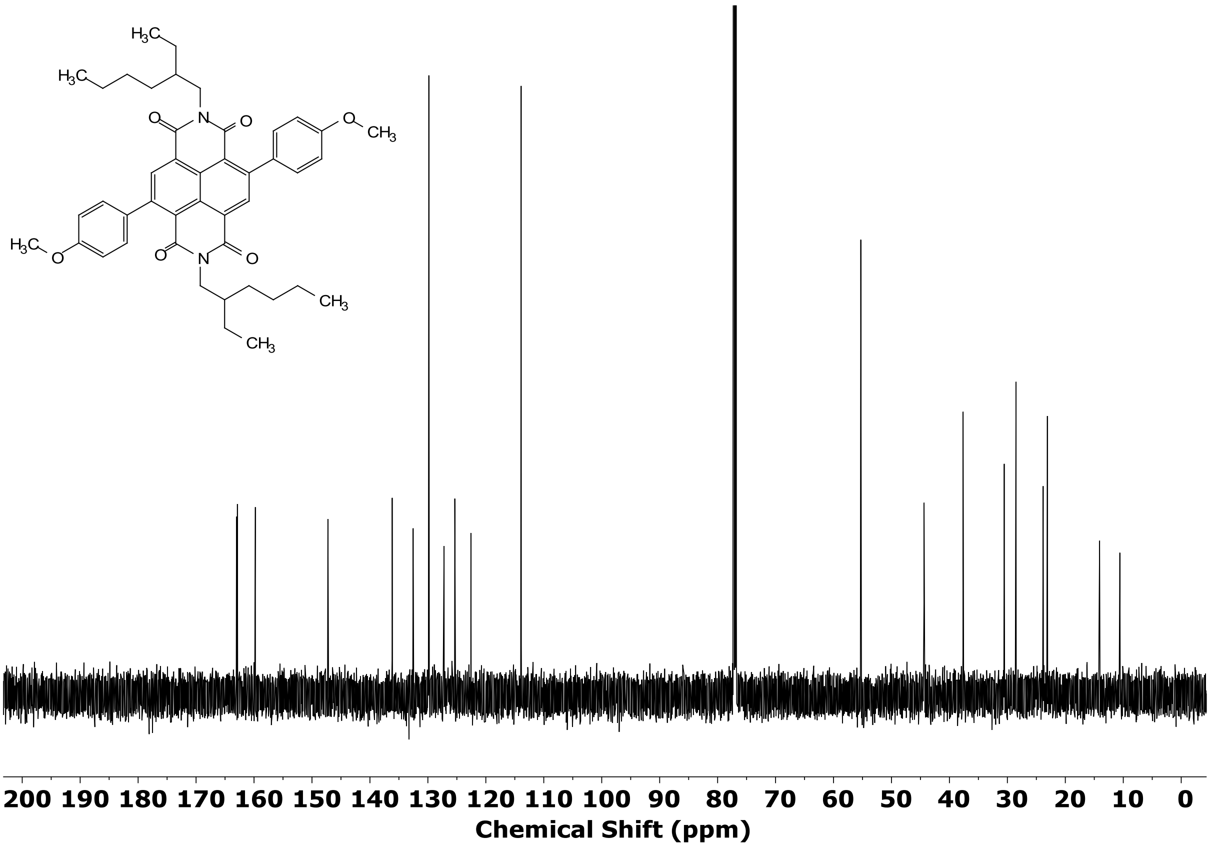

Figure S5: ^{1}H (600 MHz) and ^{13}C (150 MHz) NMR spectra of **2a** (chloroform-*d* at 298K)

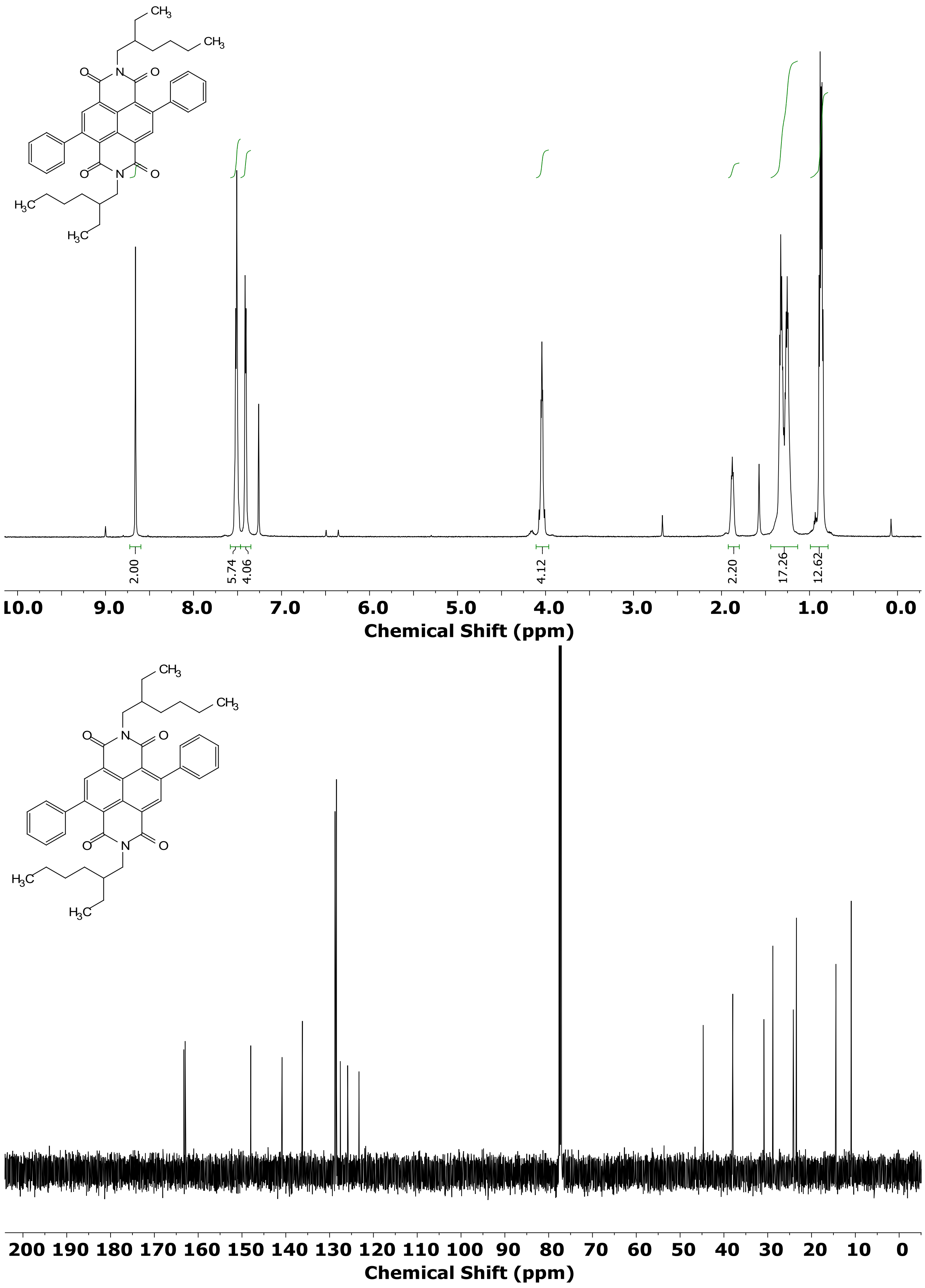

Figure S6: ^{1}H (600 MHz) and ^{13}C (150 MHz) NMR spectra of **2b** (chloroform-*d* at 298K)

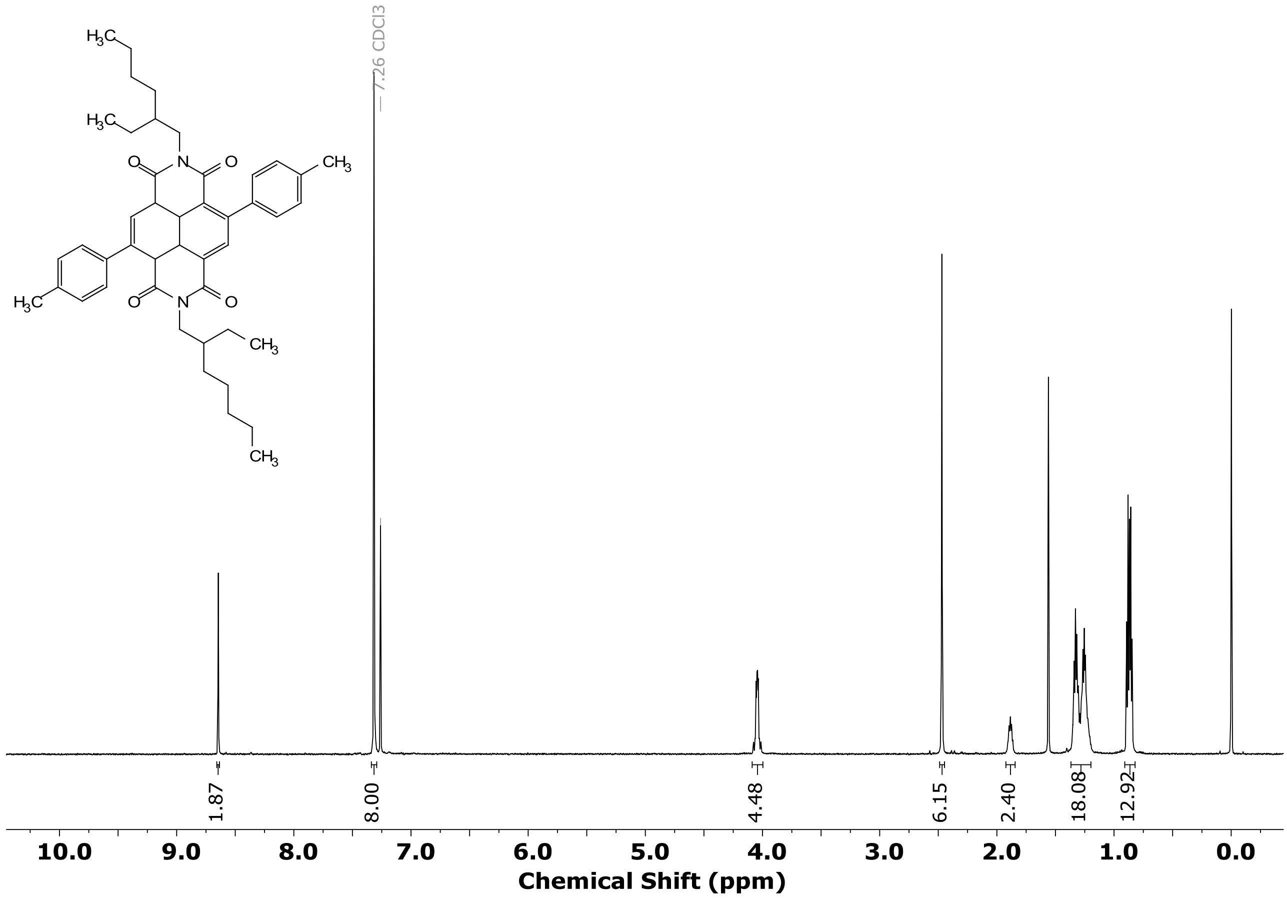

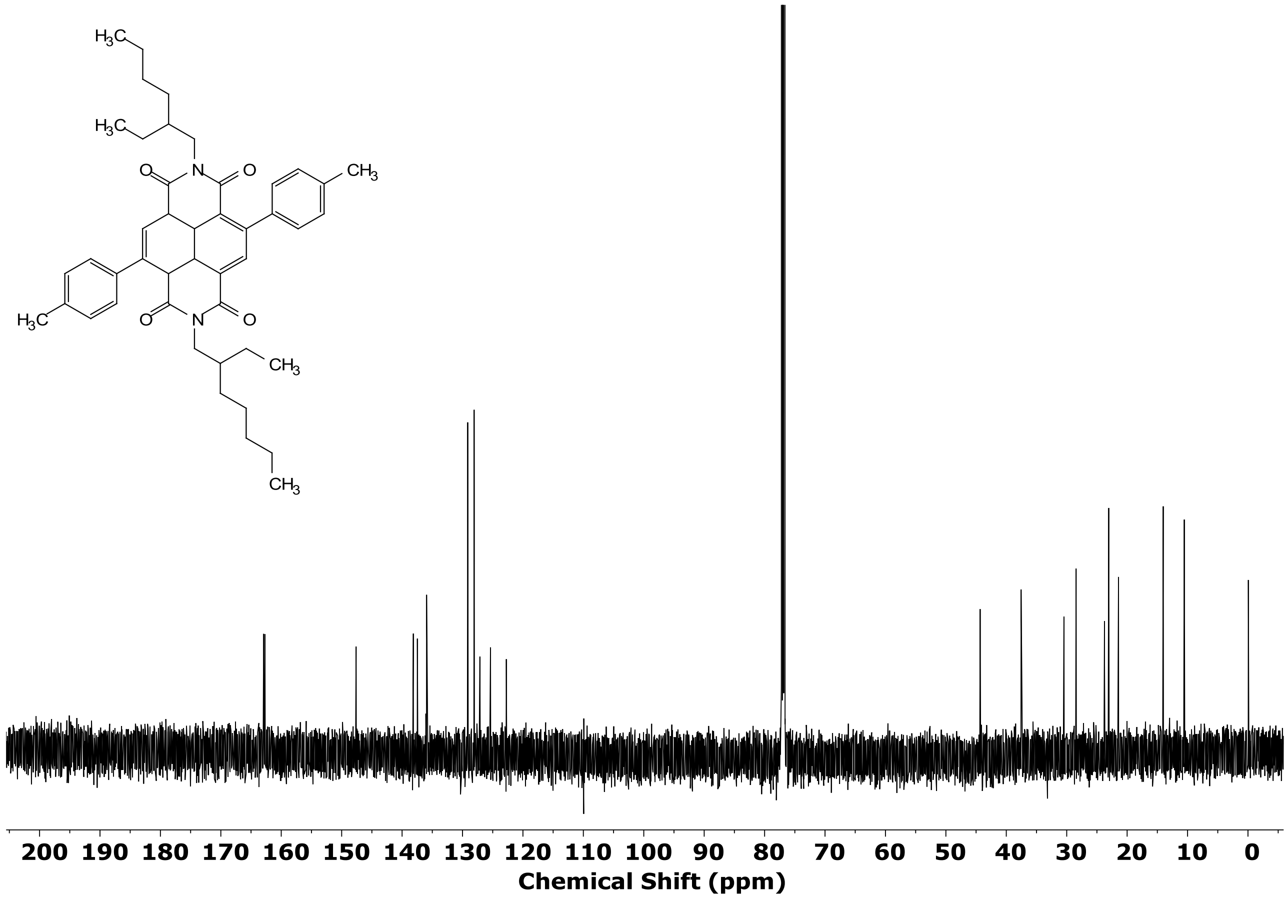

Figure S7: ^{1}H (600 MHz) and ^{13}C (150 MHz) NMR spectra of **2c** (chloroform-*d* at 298K)

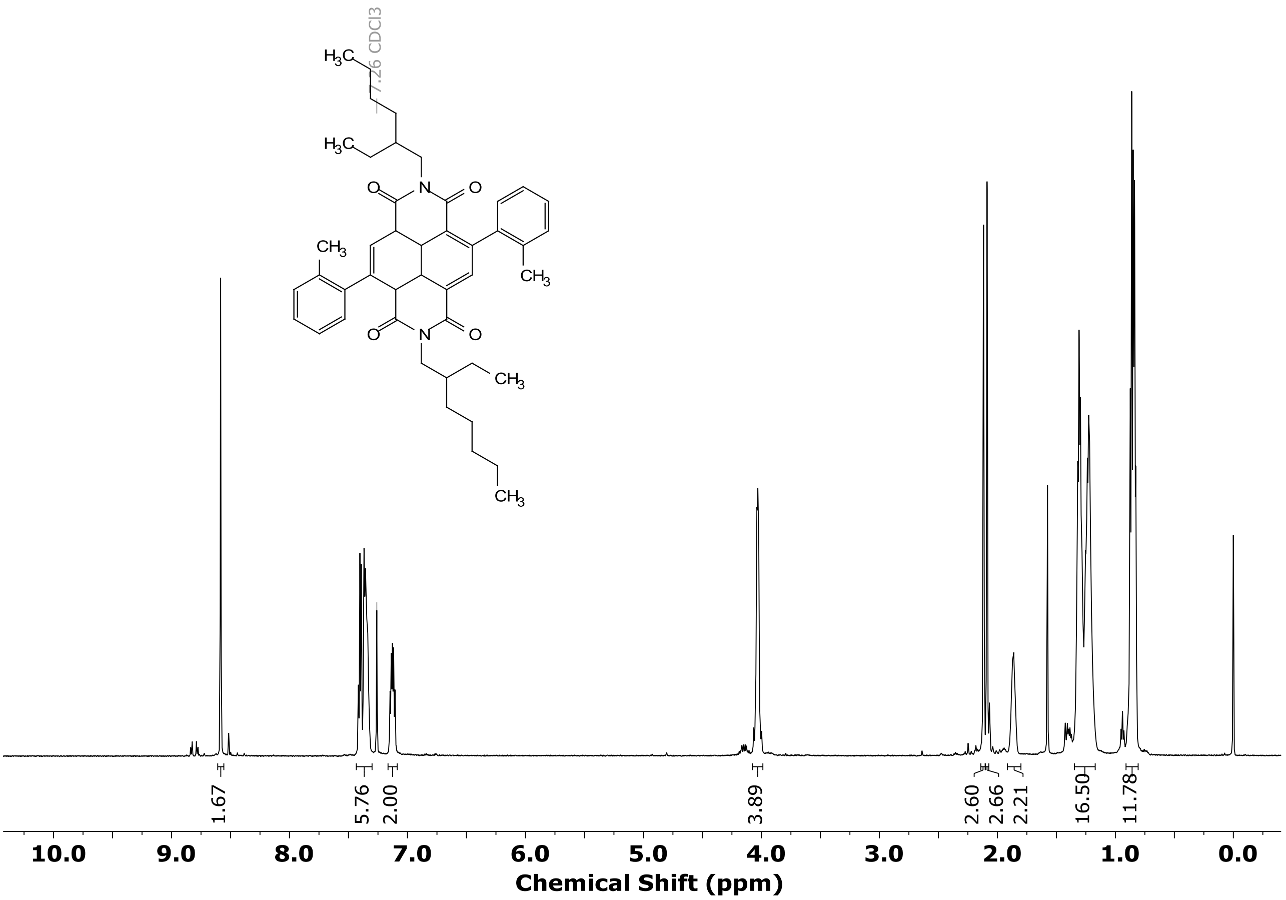

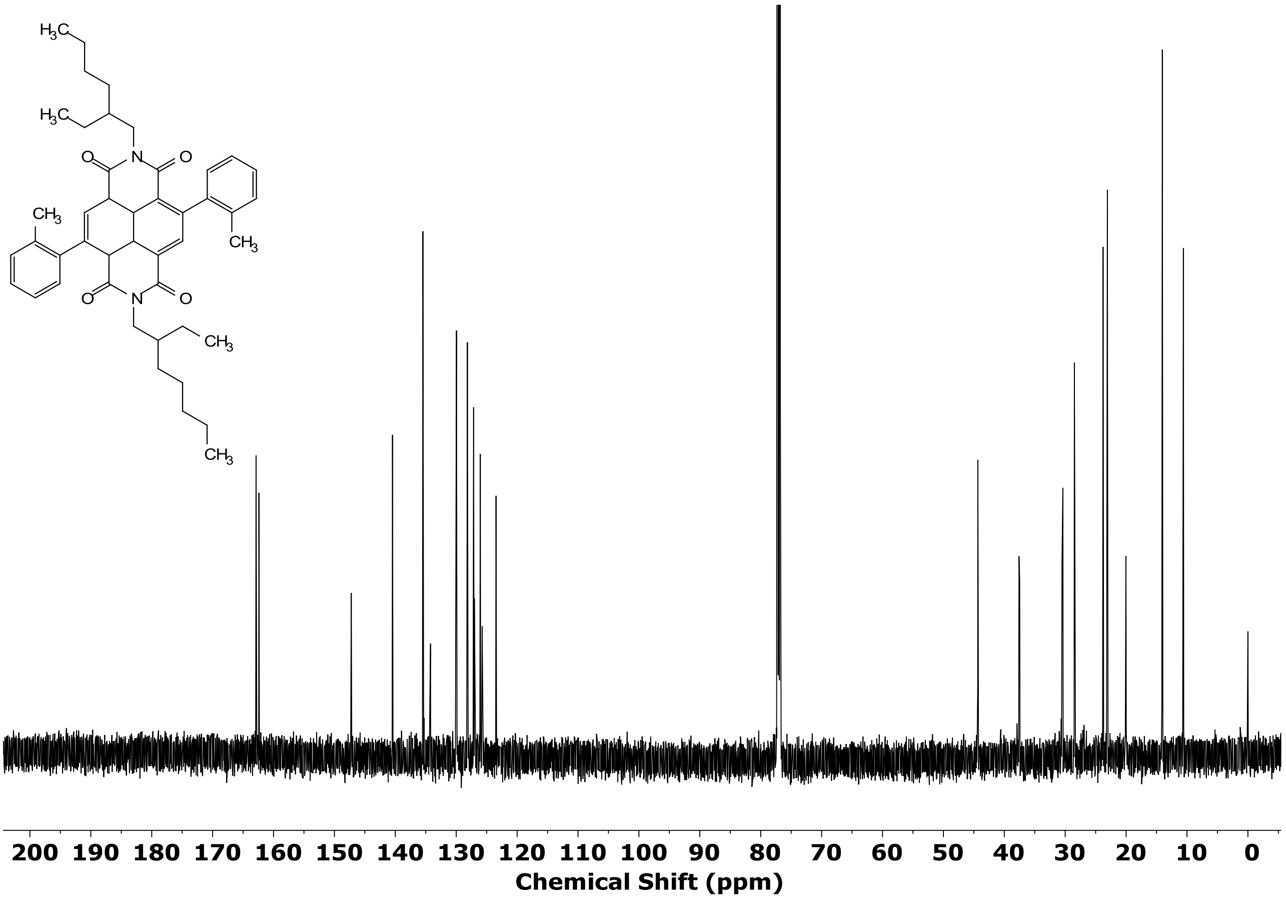

Figure S8: ^{1}H (600 MHz) and ^{13}C (150 MHz) NMR spectra of **2d** (chloroform-*d* at 298K)

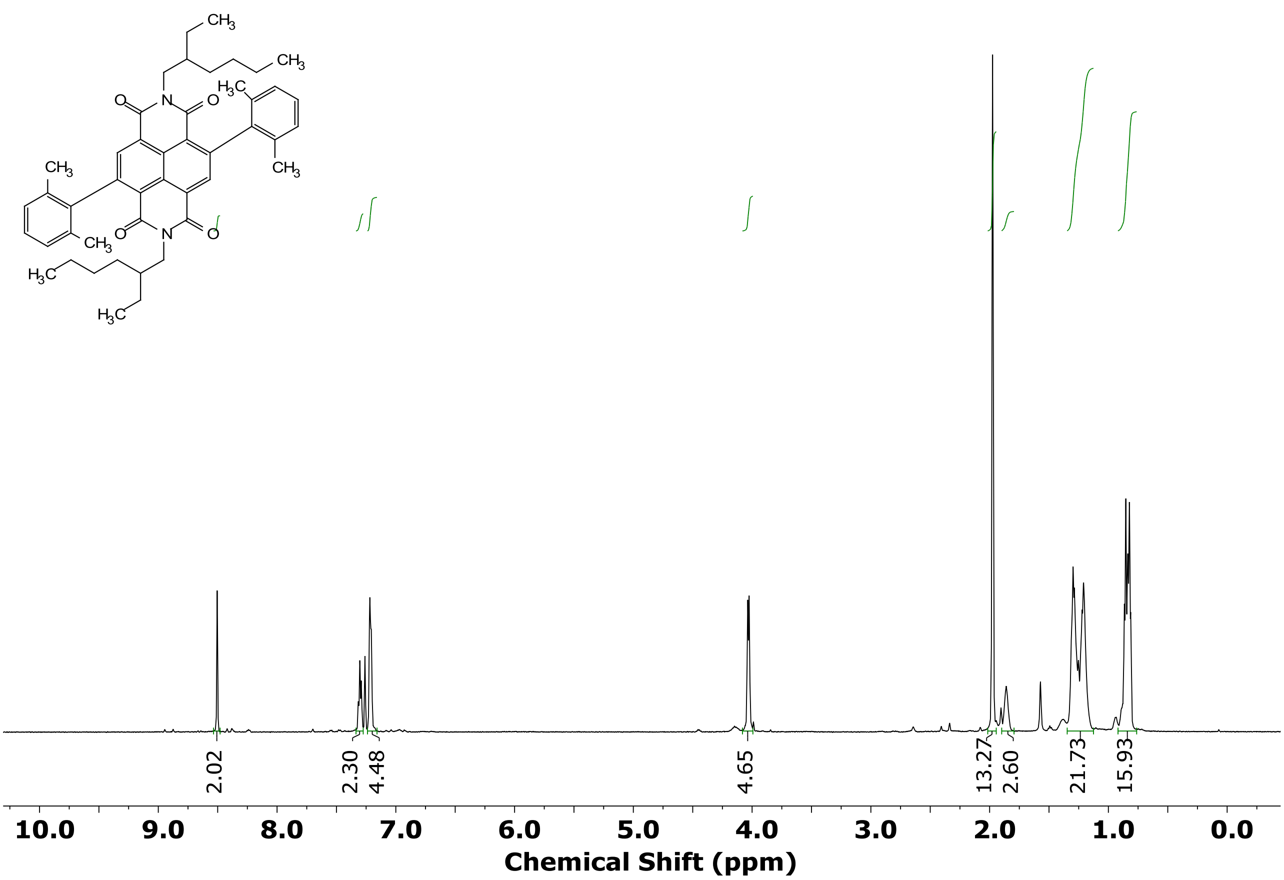

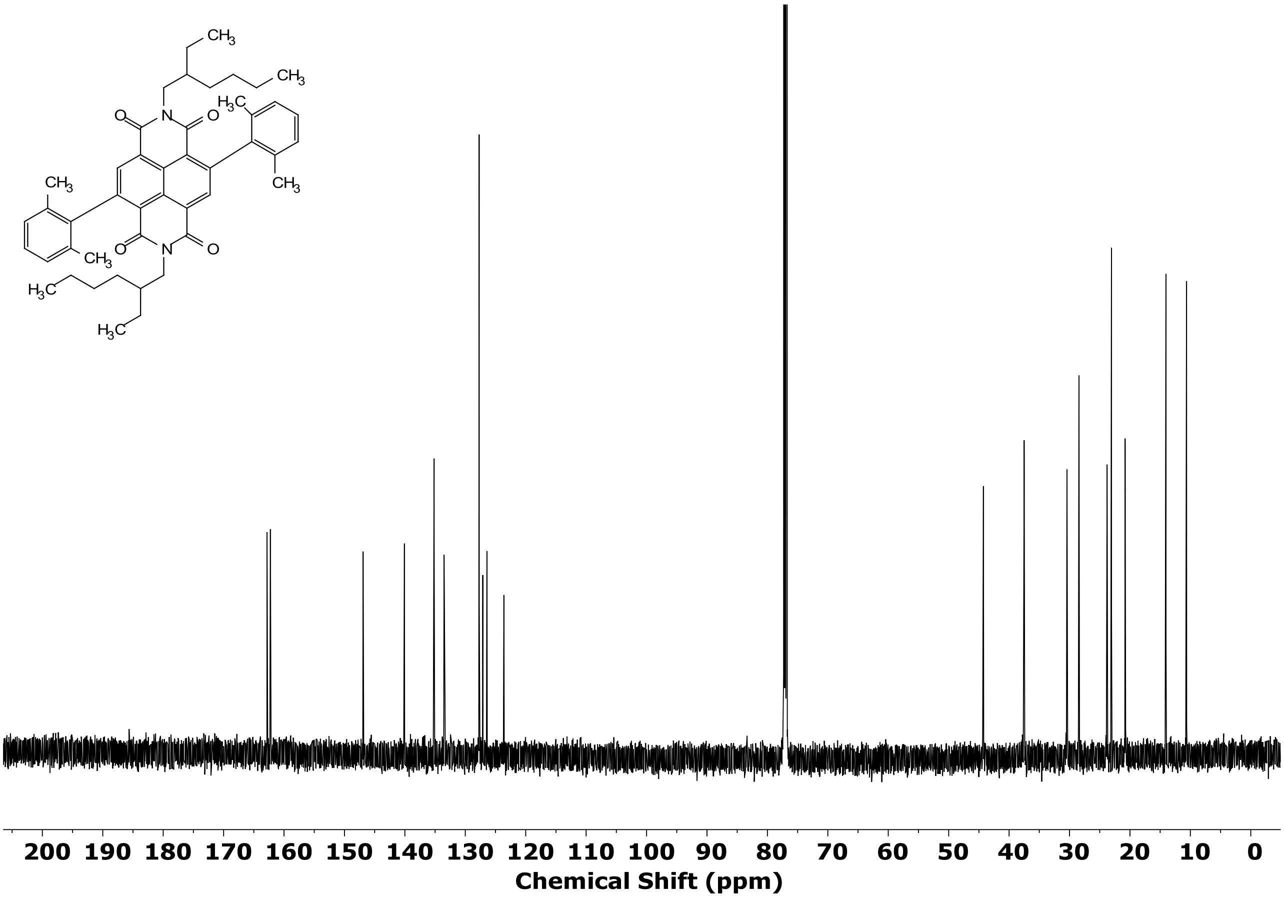

Figure S9: ^{1}H (600 MHz) and ^{13}C (150 MHz) NMR spectra of **2e** (chloroform-*d* at 298K)

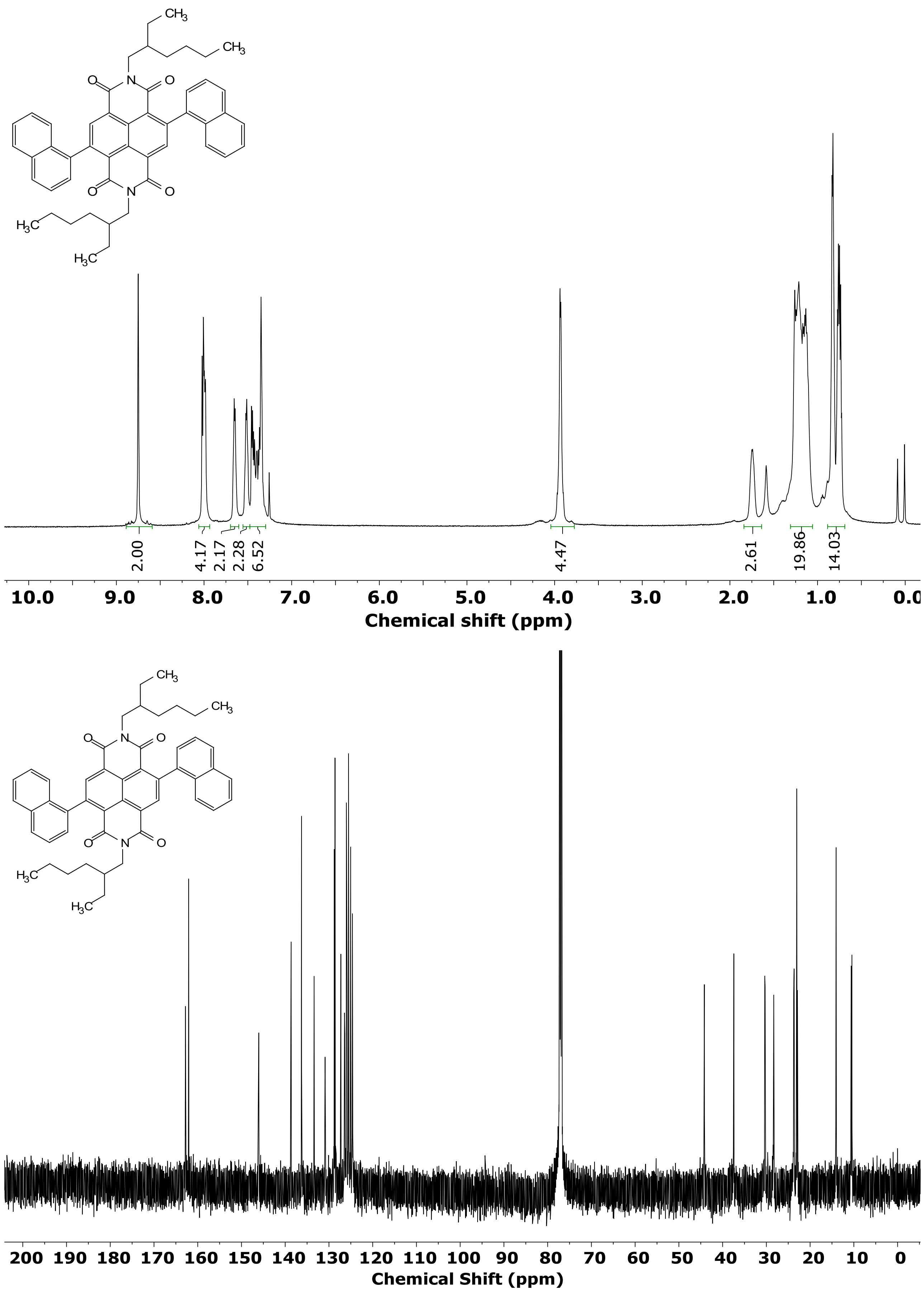

Figure S10: ^{1}H (600 MHz) and ^{13}C (150 MHz) NMR spectra of **2f** (chloroform-*d* at 298K)

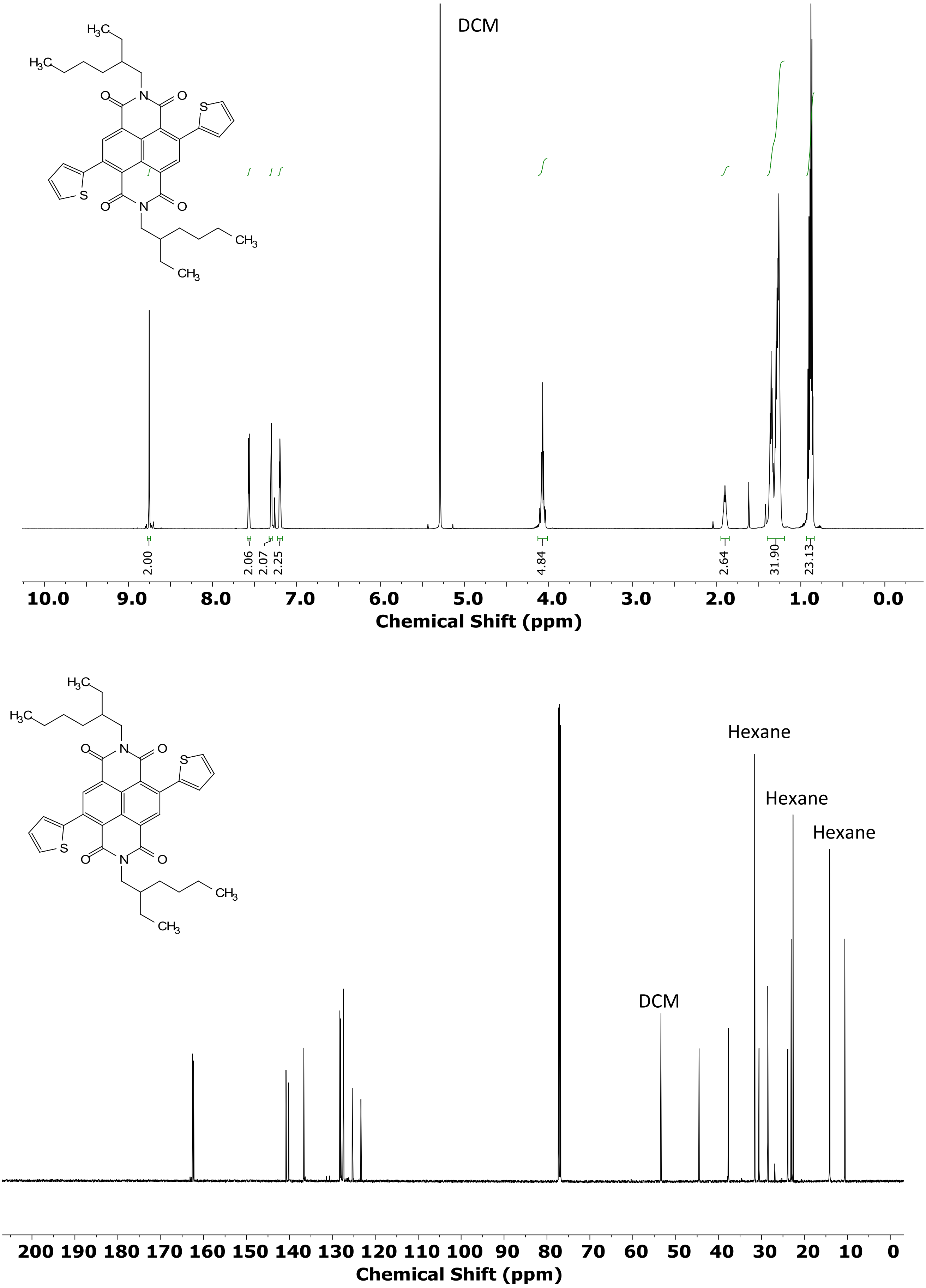

Figure S11: ^{1}H (600 MHz) and ^{13}C (150 MHz) NMR spectra of **2g** (chloroform-*d* at 298K)

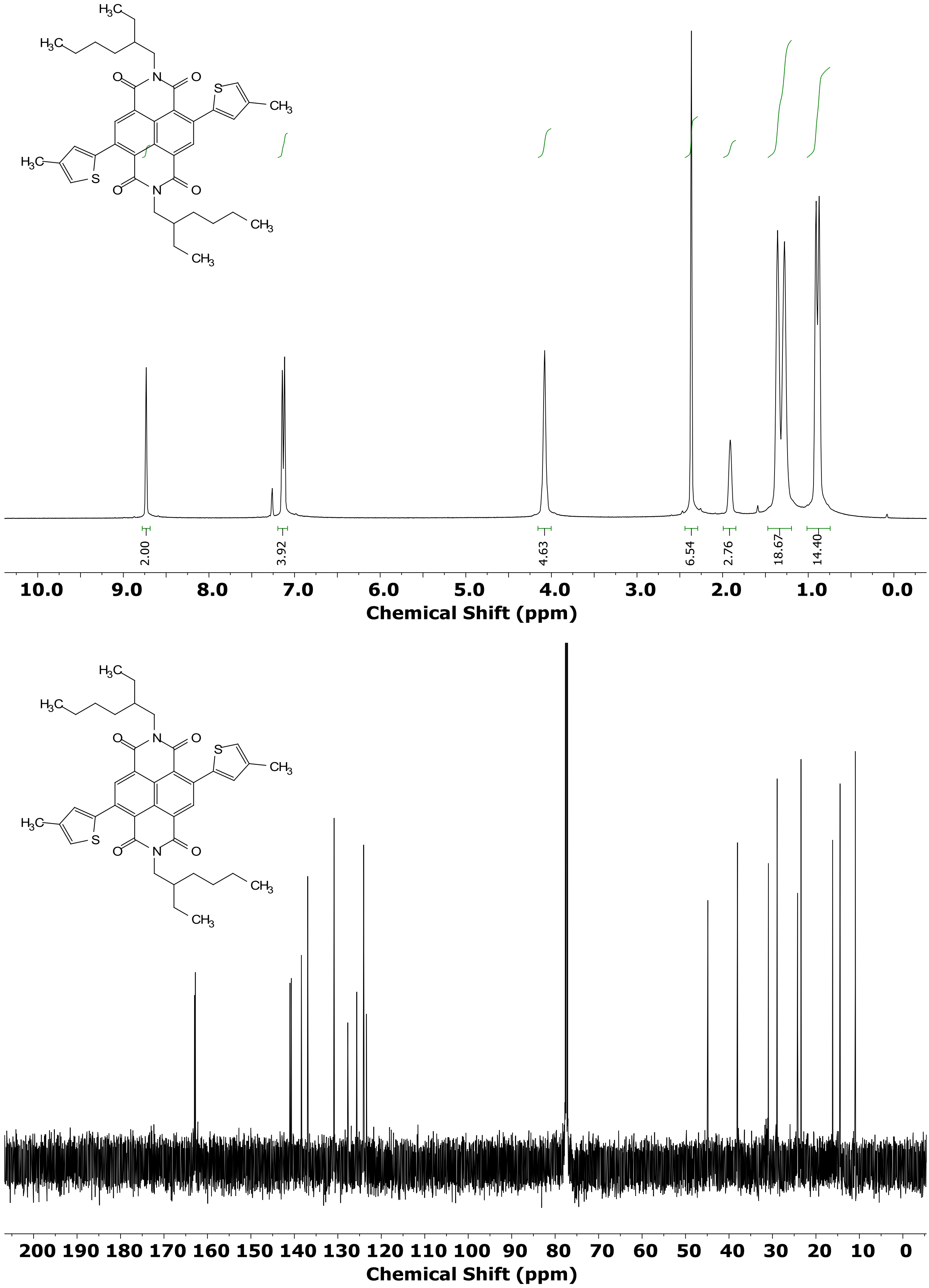

Figure S12: ^{1}H (600 MHz) and ^{13}C (150 MHz) NMR spectra of **2h** (chloroform-*d* at 298K)

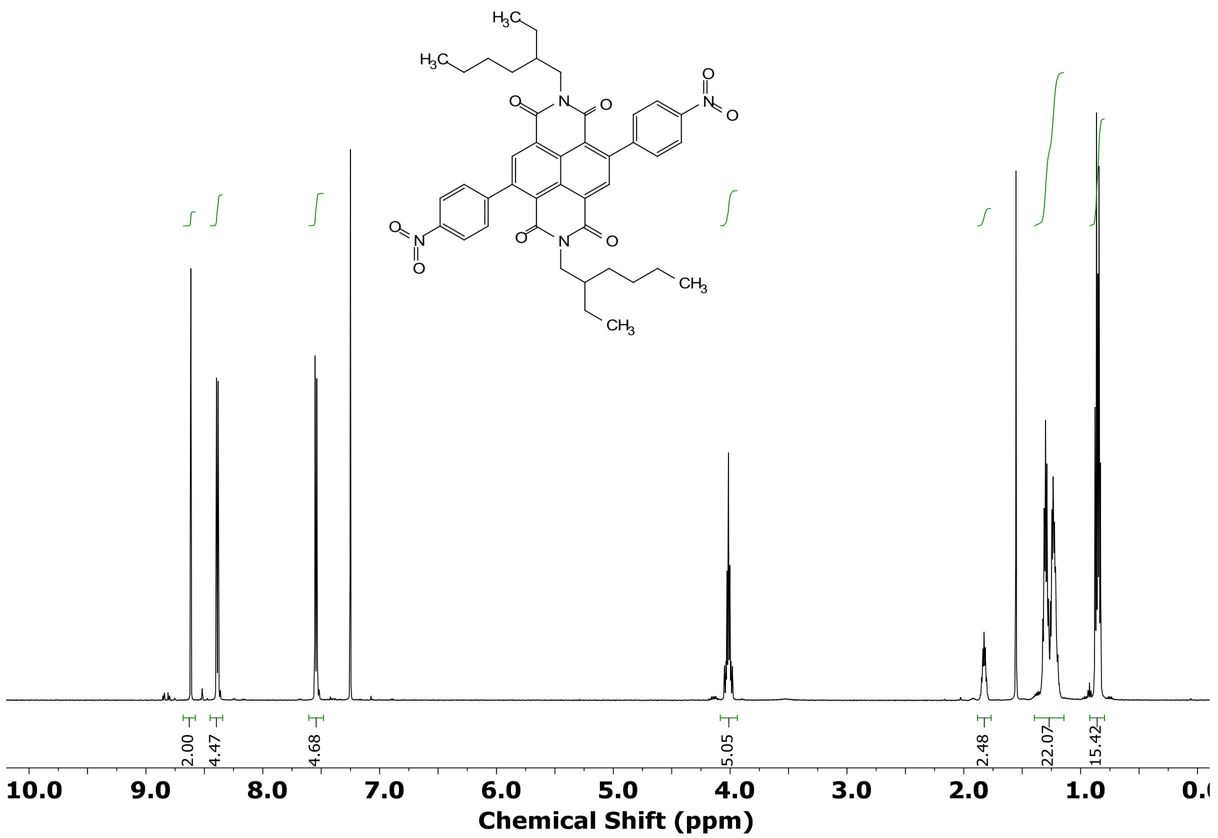

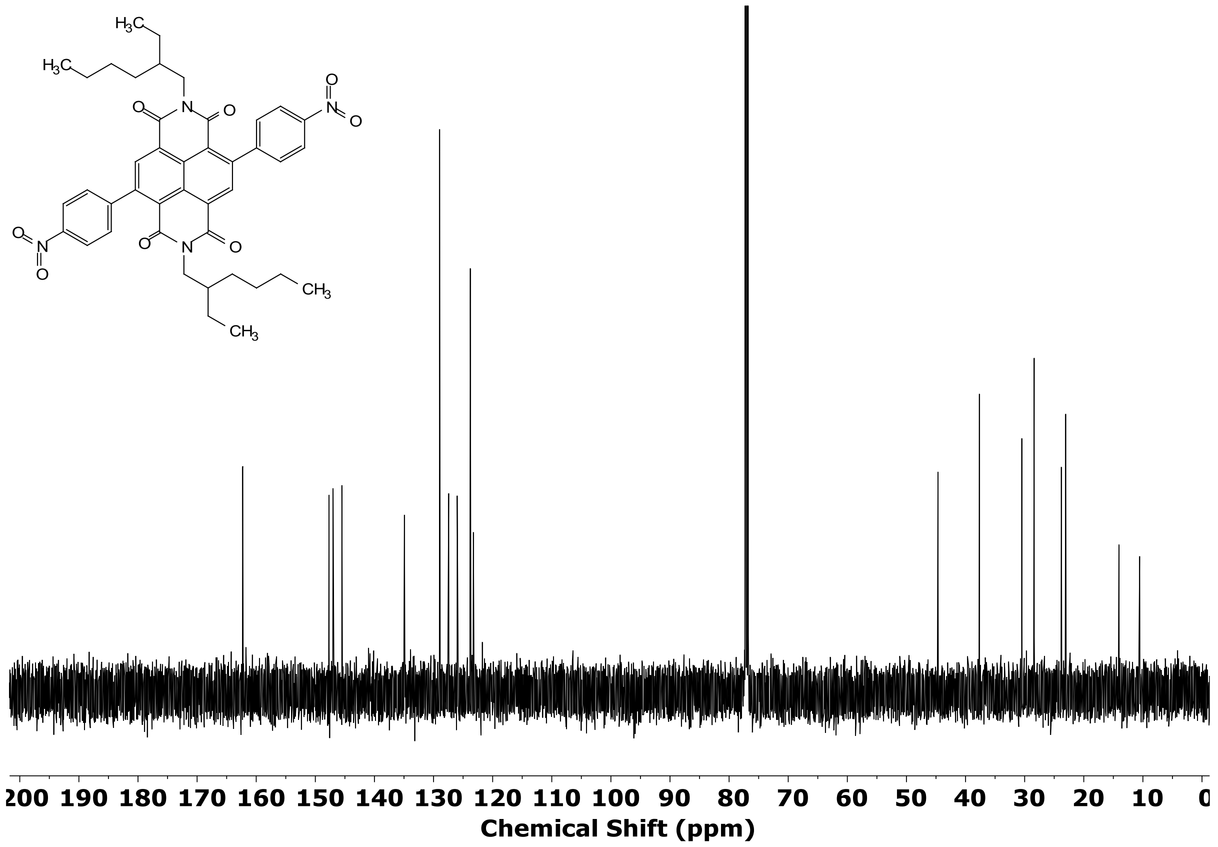

Figure S13: ^{1}H (600 MHz) and ^{13}C (150 MHz) NMR spectra of **2i** (chloroform-*d* at 298K)

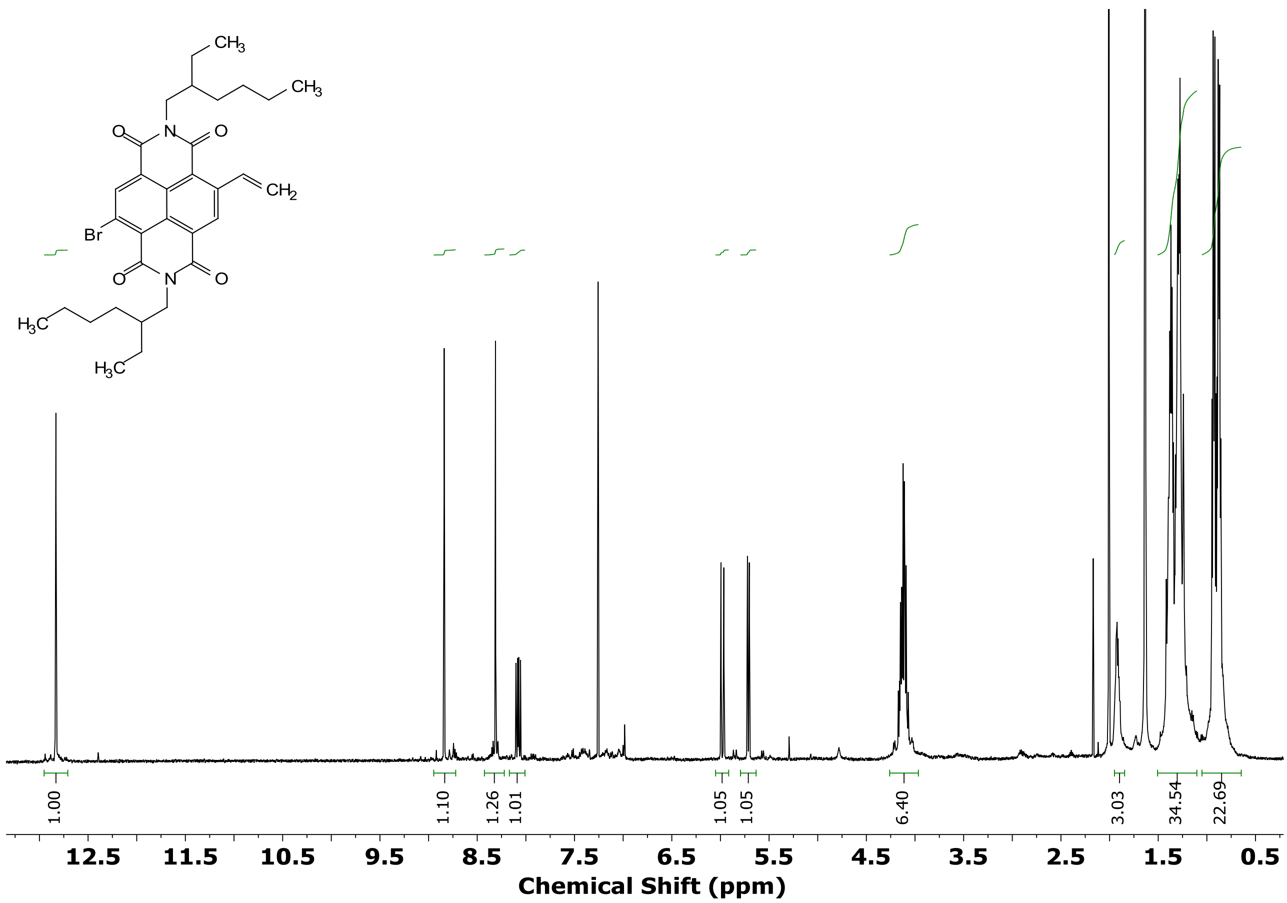

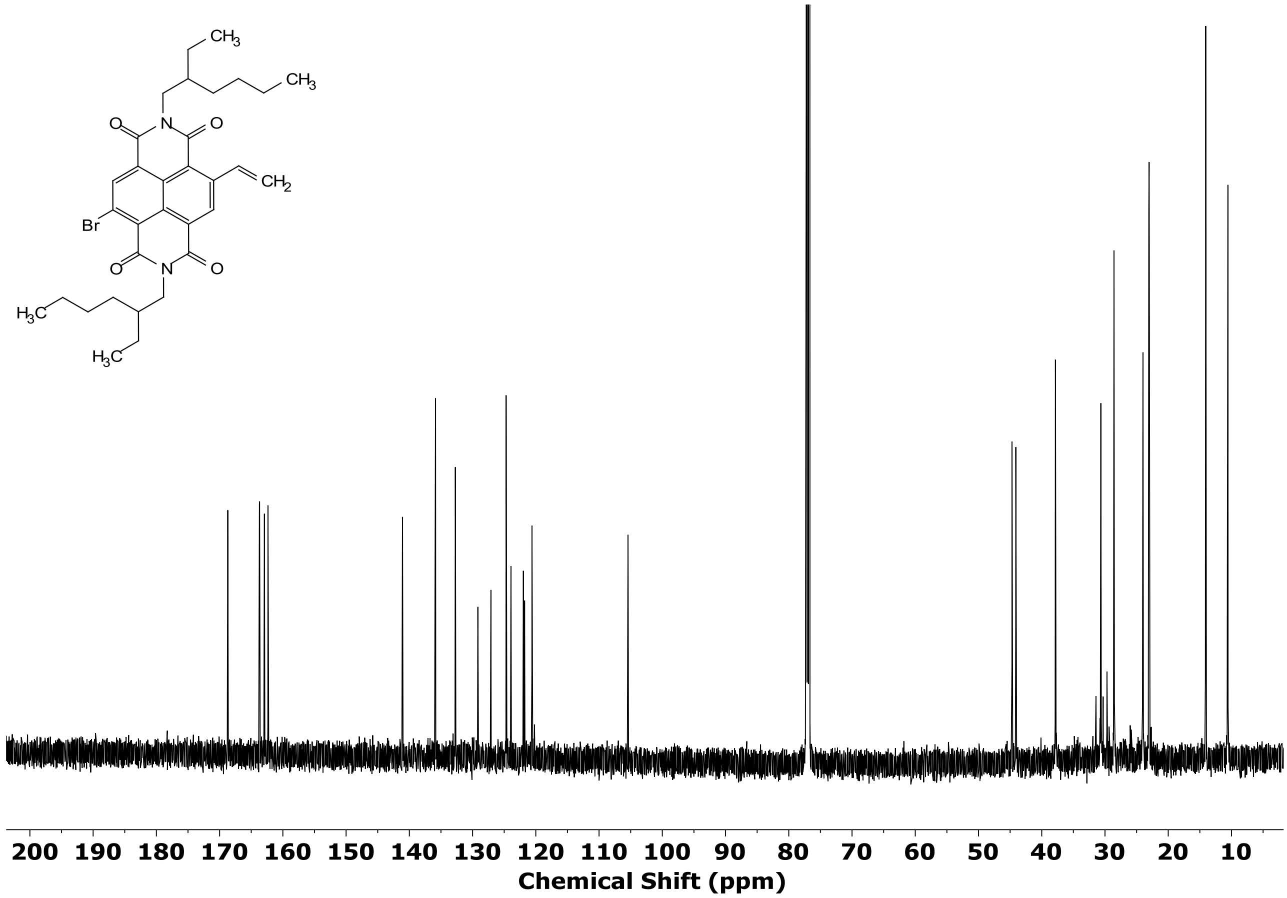

Figure S14: ^{1}H (600 MHz) and ^{13}C (150 MHz) NMR spectra of **2k** (chloroform-*d* at 298K)

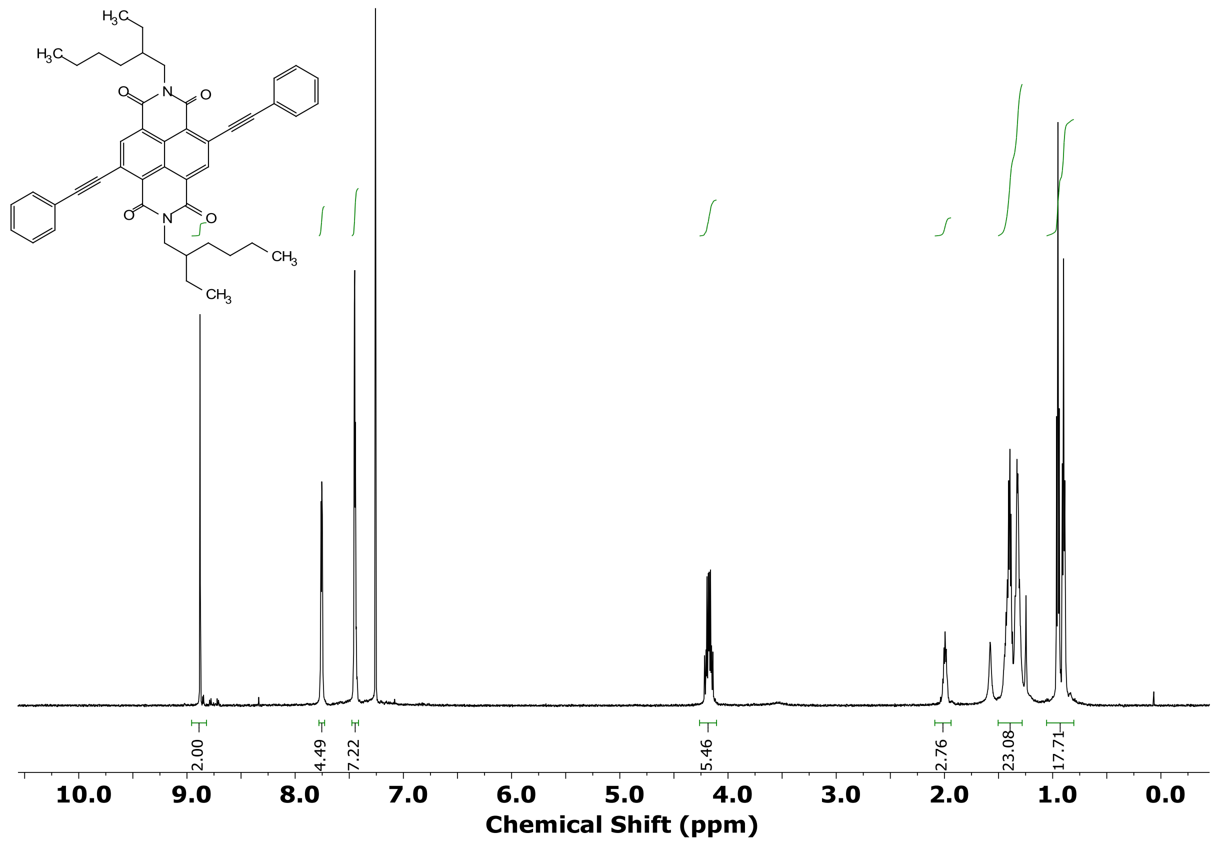

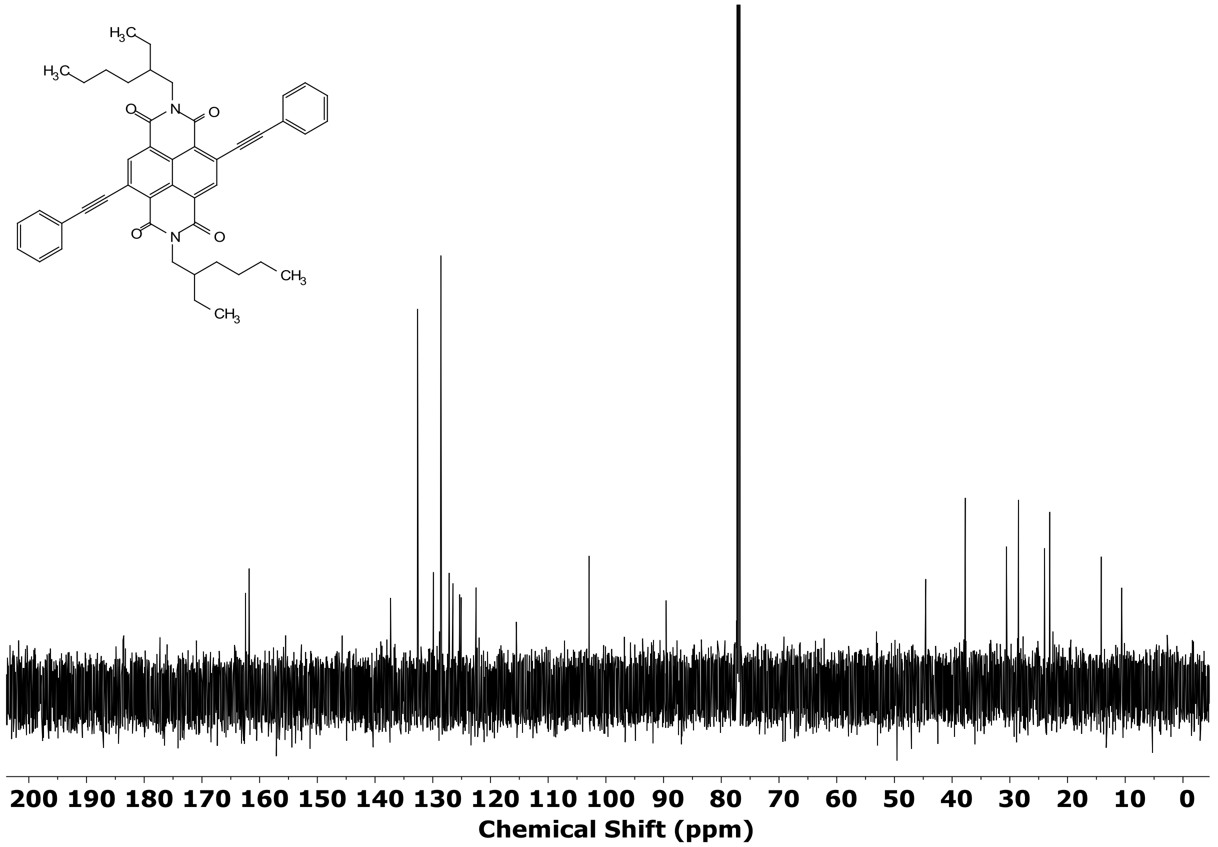

Figure S15: ^{1}H (600 MHz) and ^{13}C (150 MHz) NMR spectra of **3a** (chloroform-*d* at 298K)

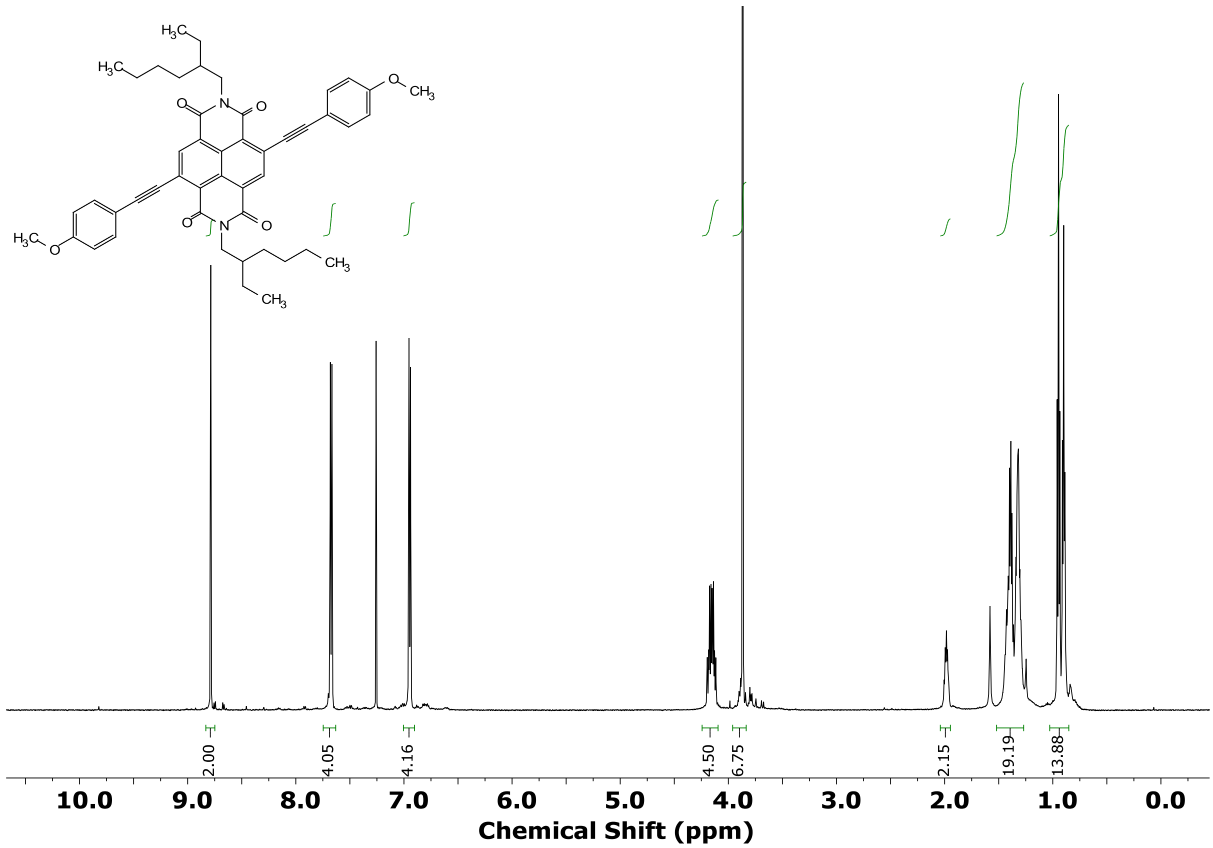

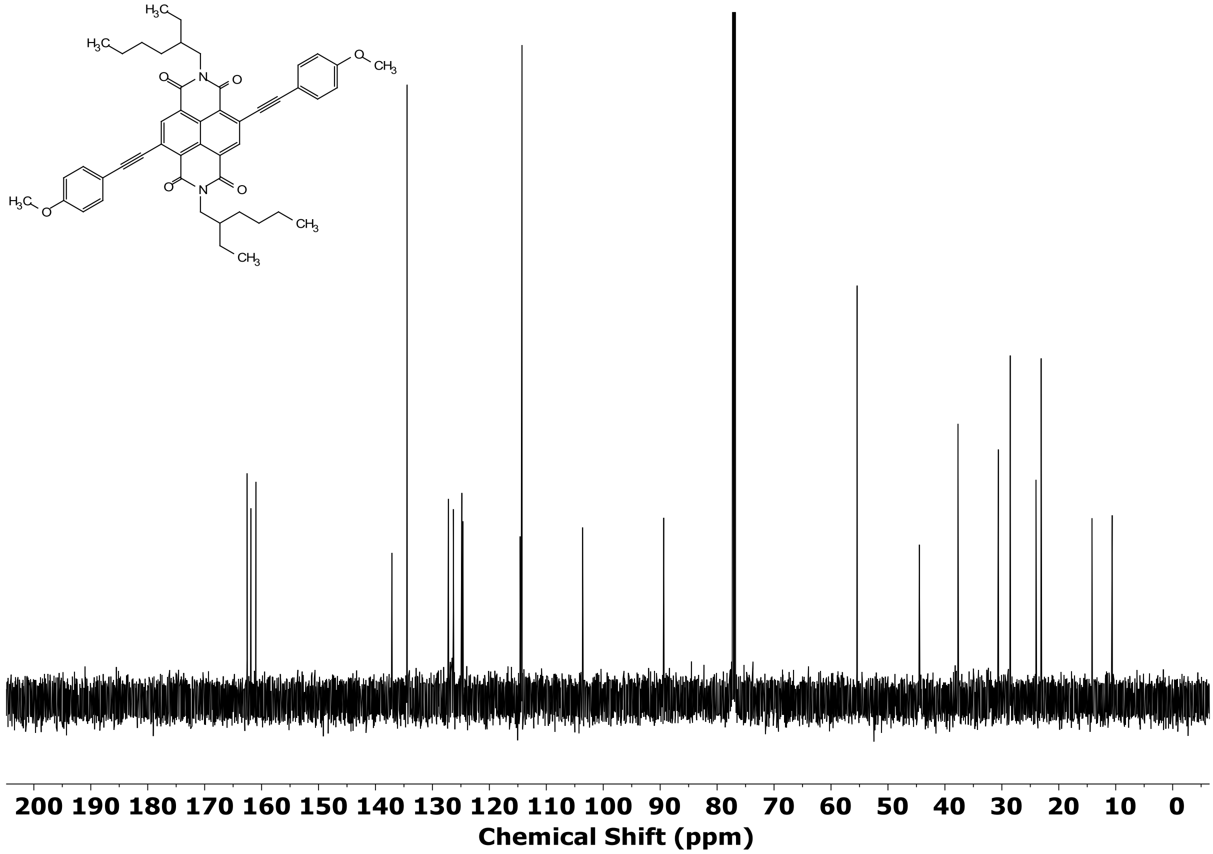

Figure S16: ^{1}H (600 MHz) and ^{13}C (150 MHz) NMR spectra of **3b** (chloroform-*d* at 298K)

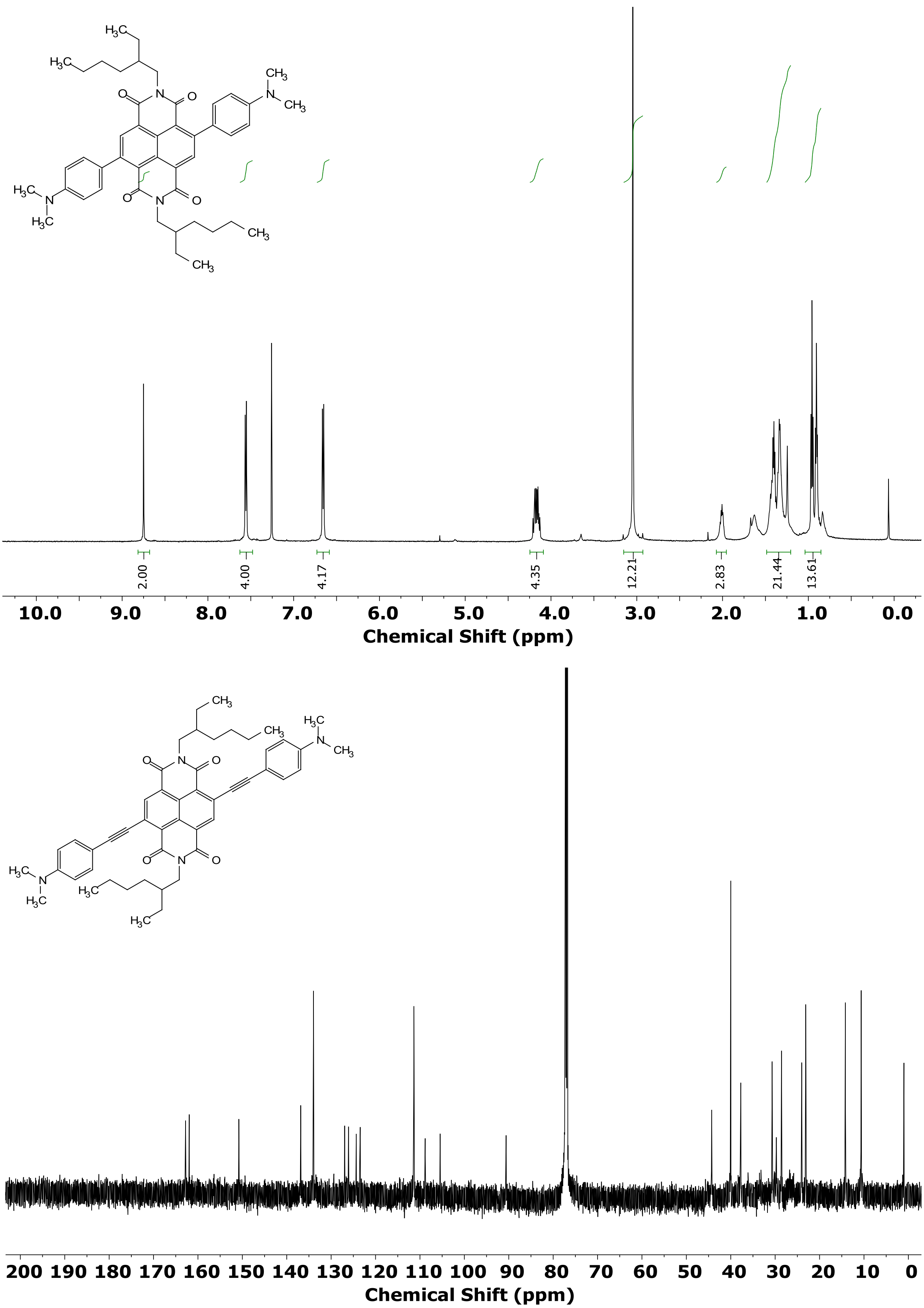

Figure S17: ^{1}H (600 MHz) and ^{13}C (150 MHz) NMR spectra of **3c** (chloroform-*d* at 298K)

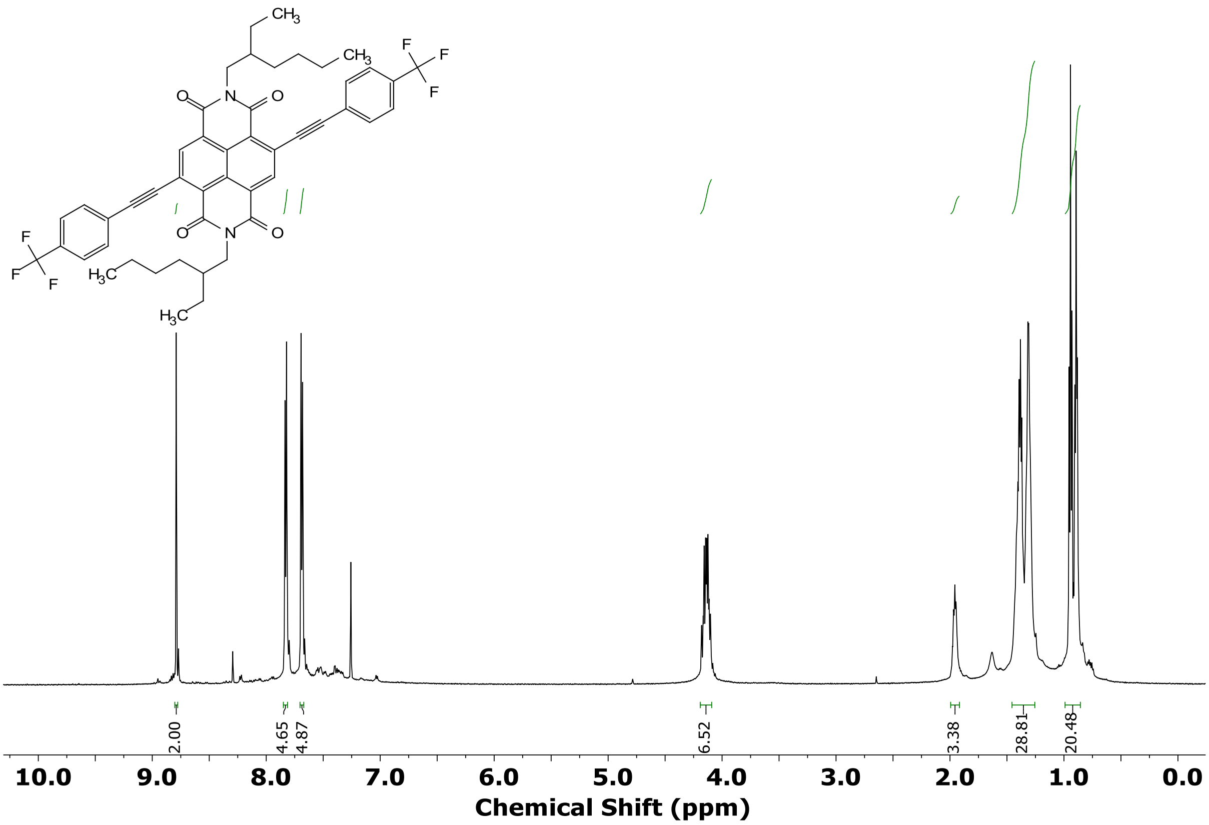

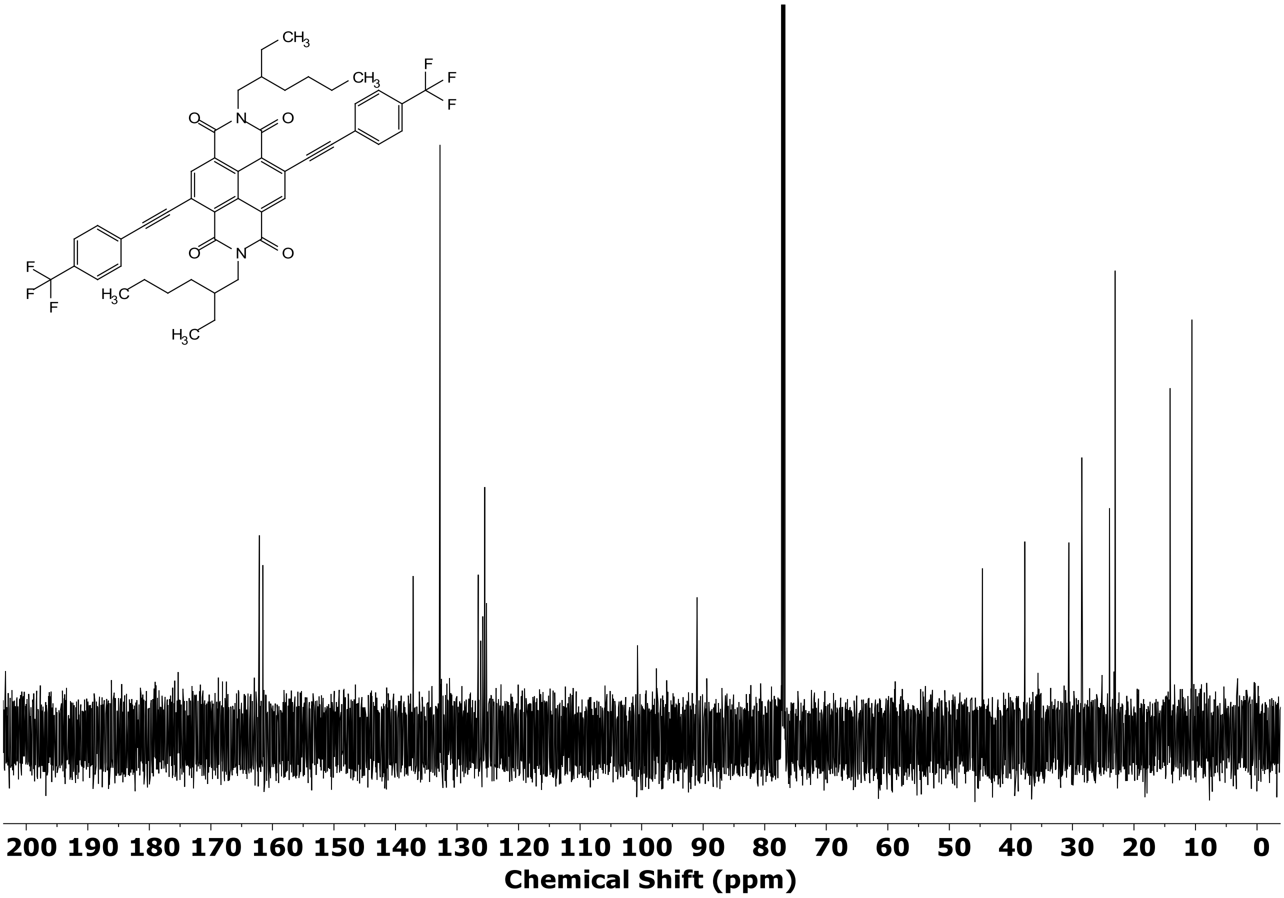

Figure S18: ^{1}H (600 MHz) and ^{13}C (150 MHz) NMR spectra of **3d** (chloroform-*d* at 298K)

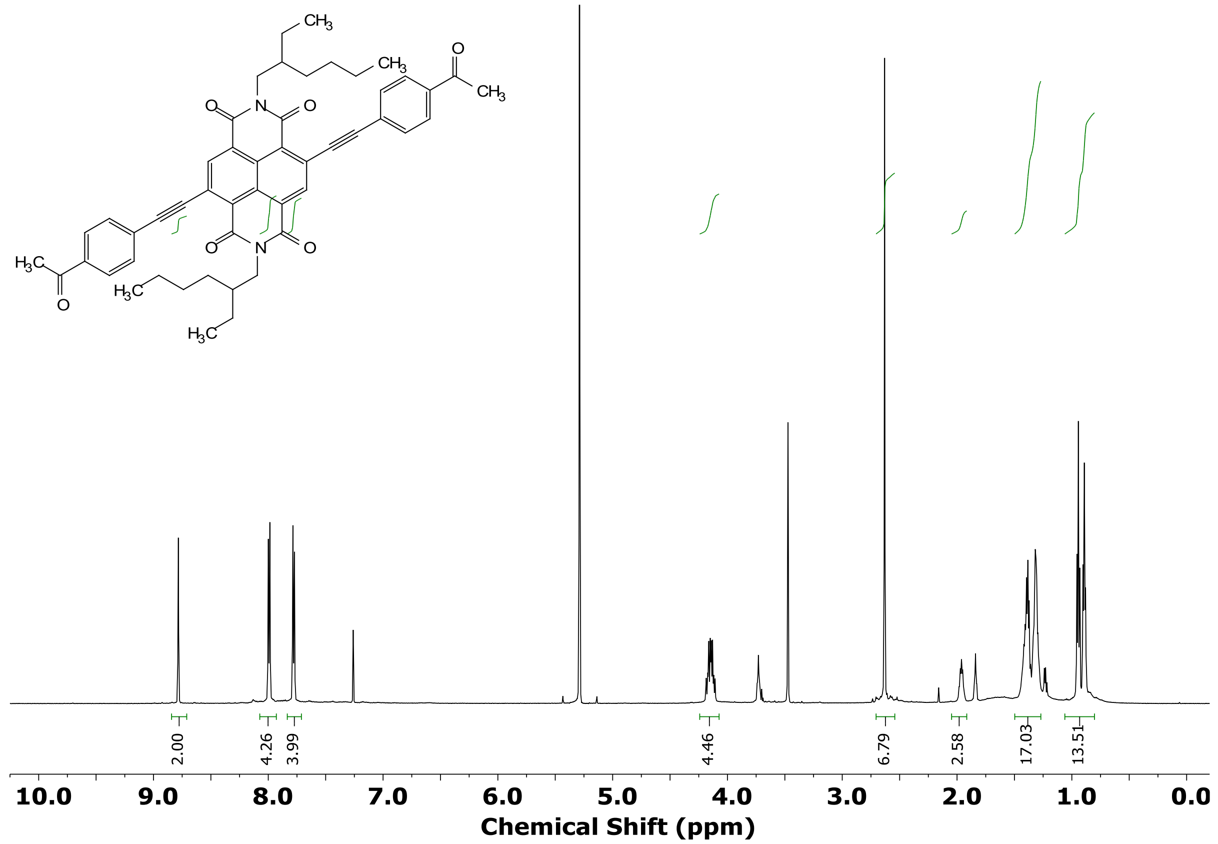

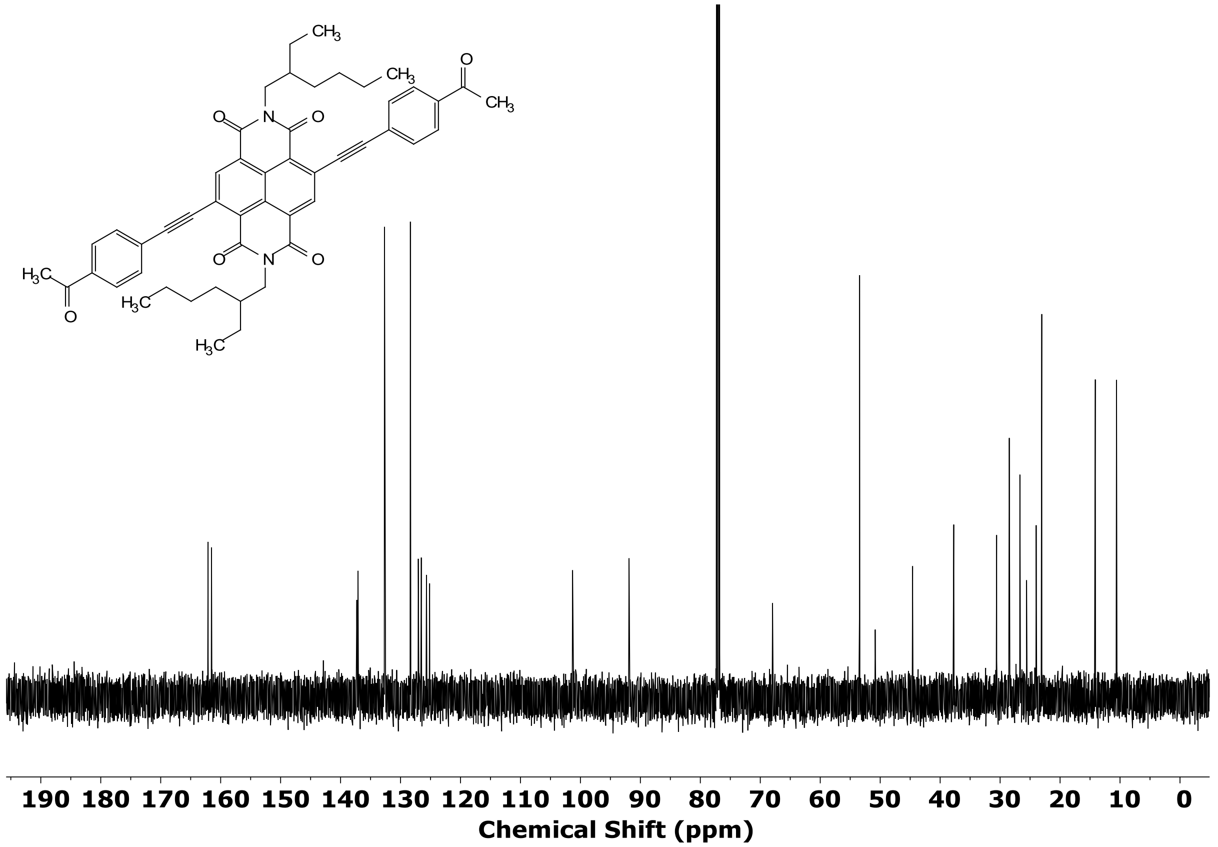

Figure S19: ^{1}H (600 MHz) and ^{13}C (150 MHz) NMR spectra of **3e** (chloroform-*d* at 298K)

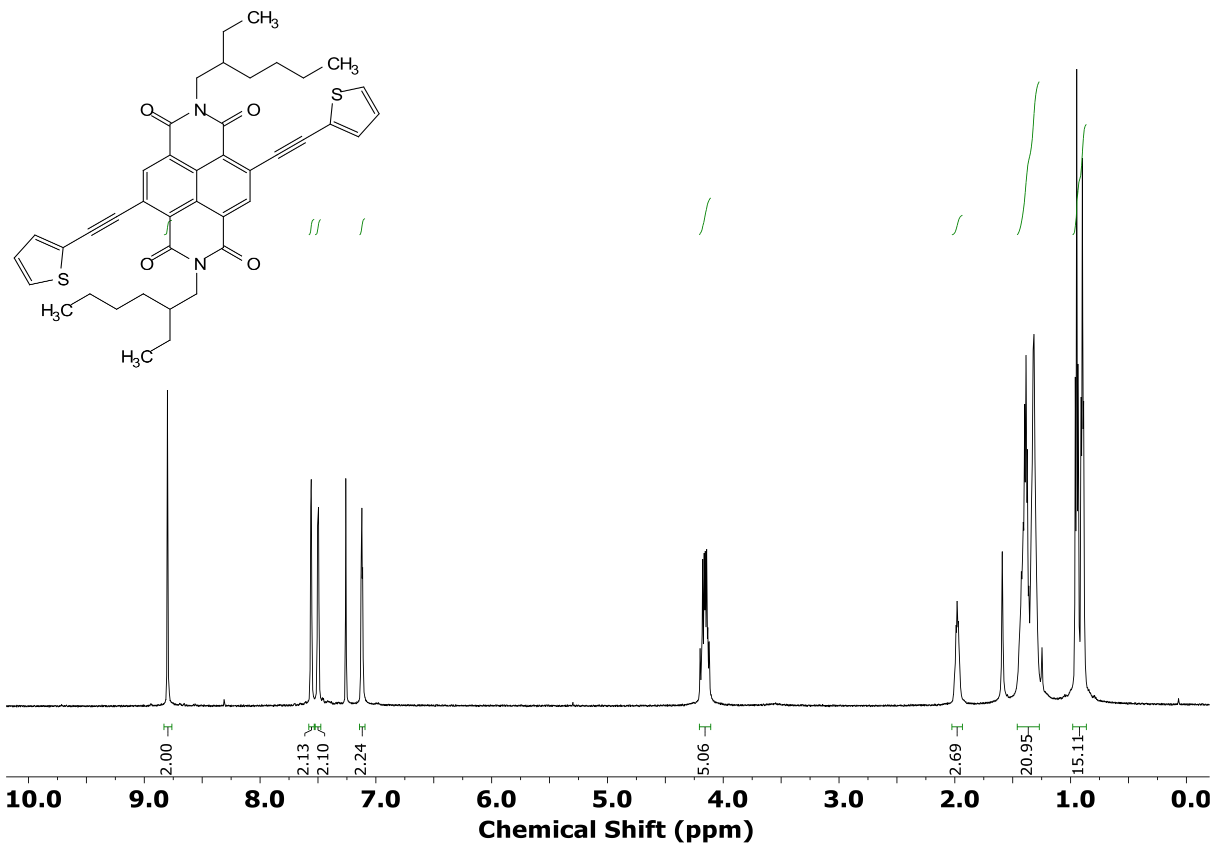

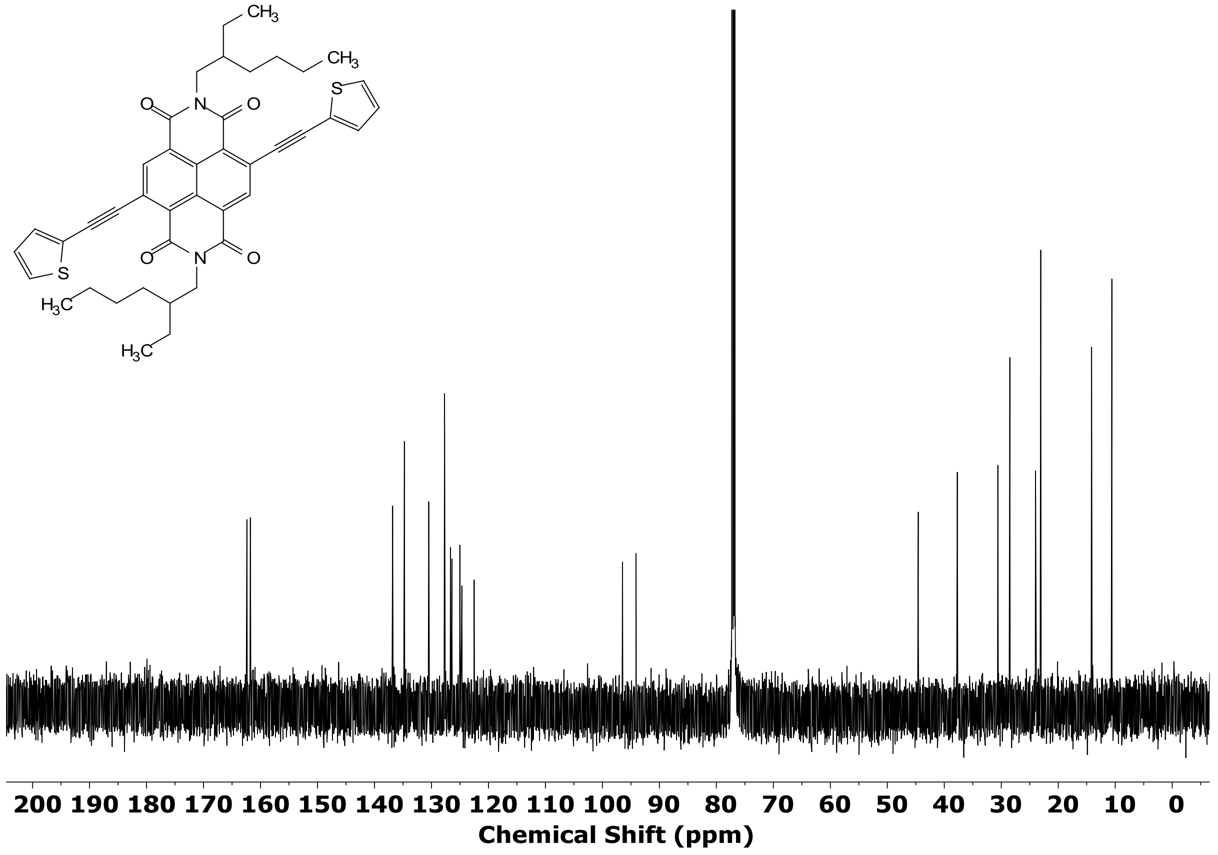

Figure S20: ^{1}H (600 MHz) and ^{13}C (150 MHz) NMR spectra of **3f** (chloroform-*d* at 298K)

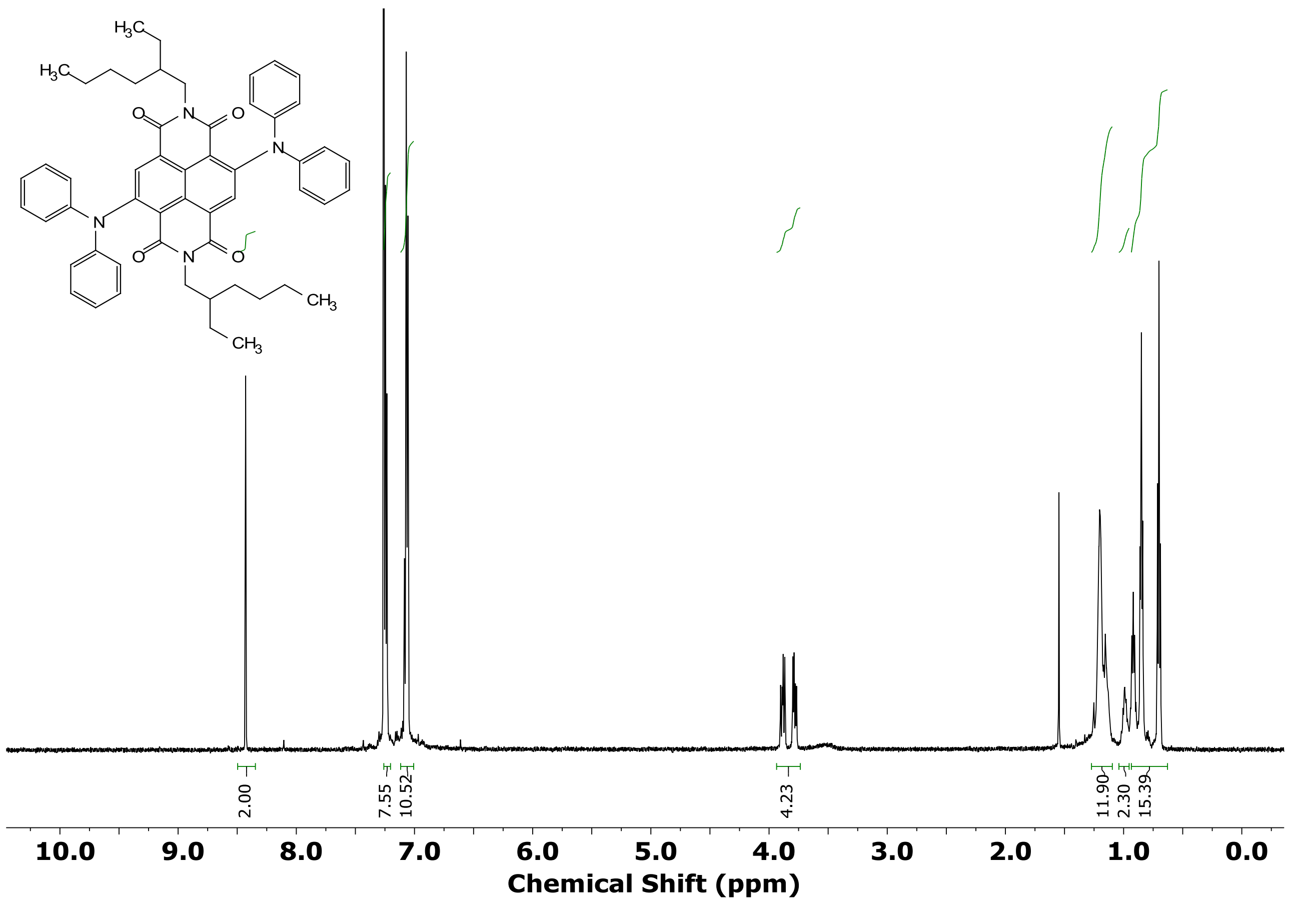

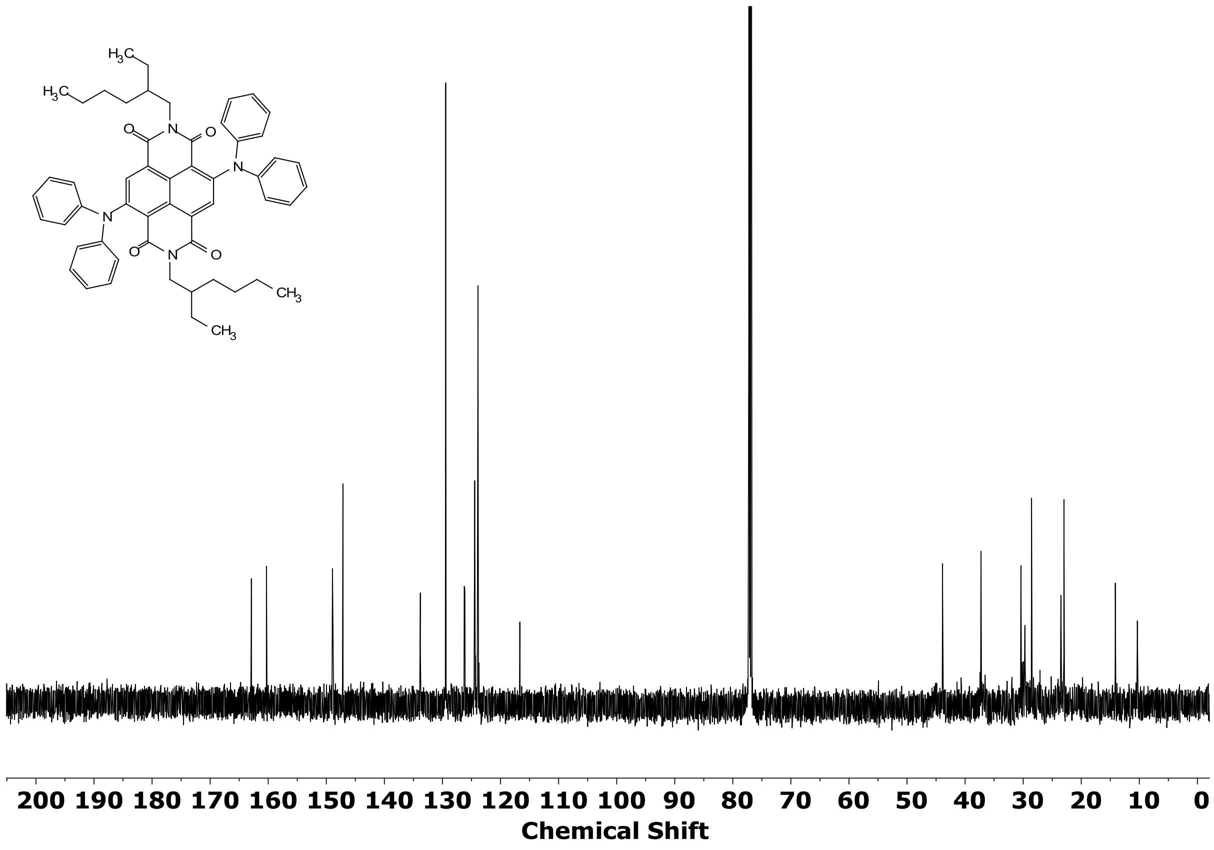

Figure S21: ^{1}H (600 MHz) and ^{13}C (150 MHz) NMR spectra of **4a** (chloroform-*d* at 298K)

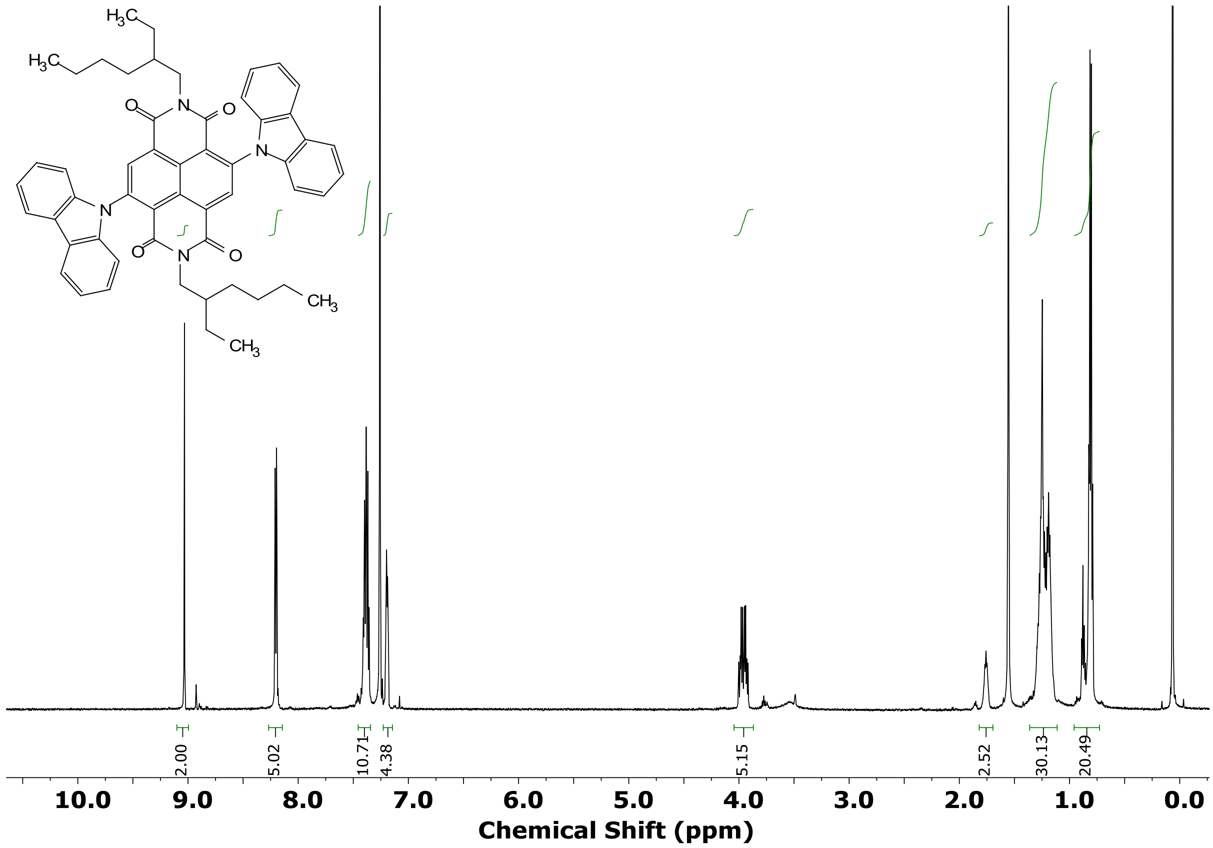

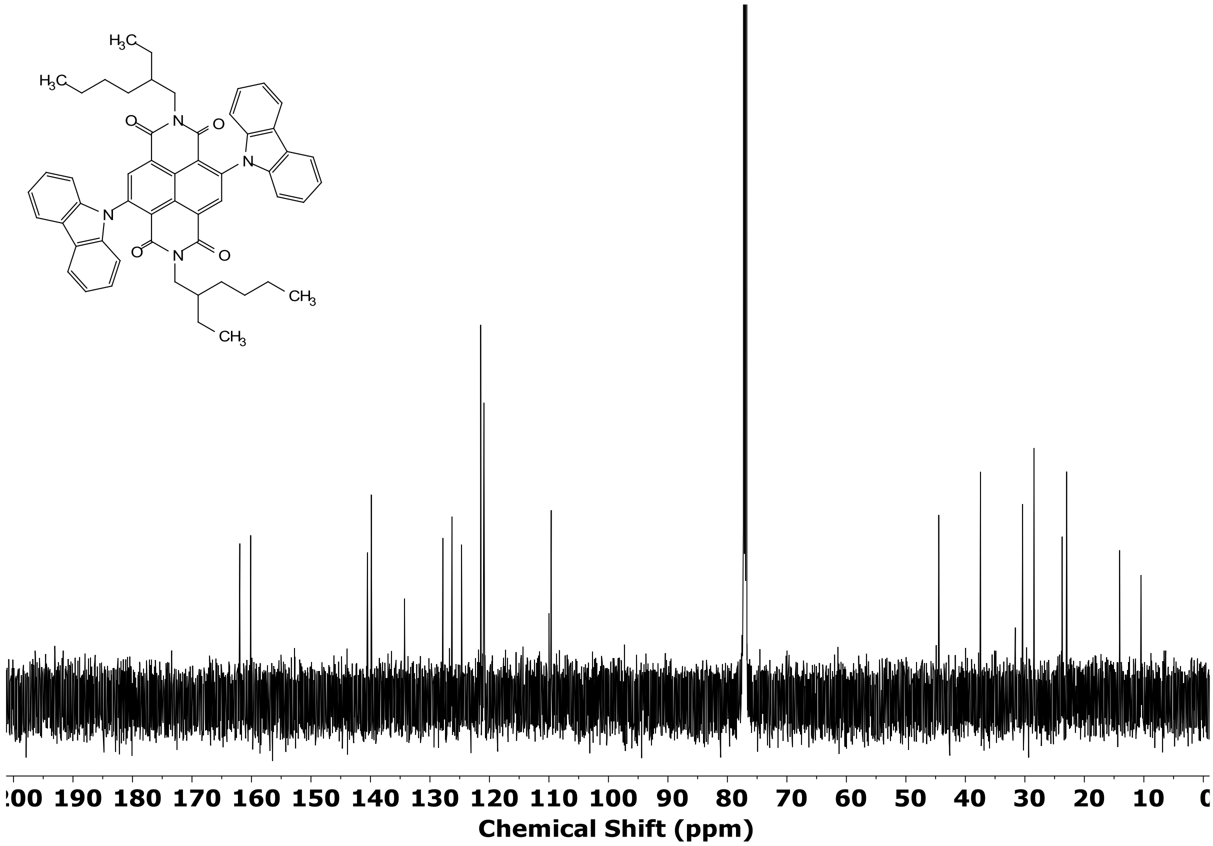

Figure S22: ^{1}H (600 MHz) and ^{13}C (150 MHz) NMR spectra of **4b** (chloroform-*d* at 298K)

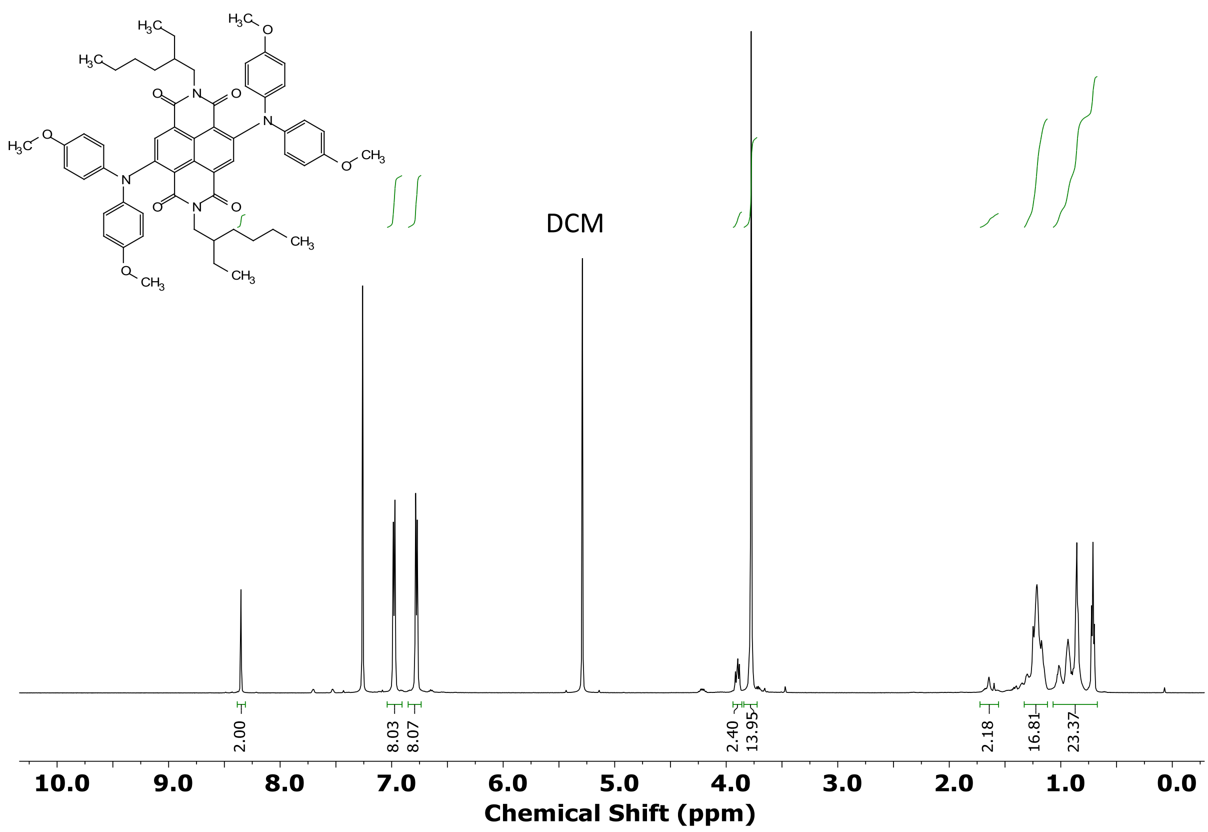

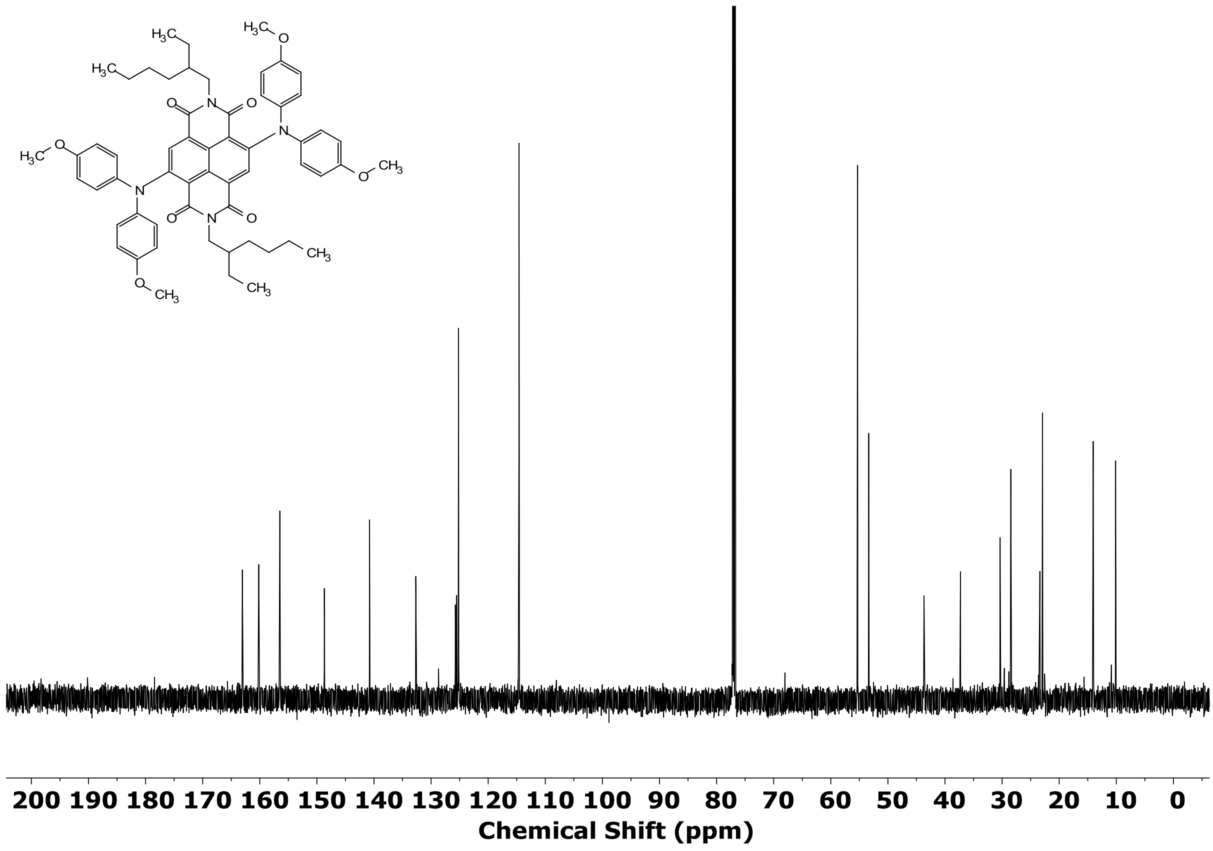

Figure S23: ^{1}H (600 MHz) and ^{13}C (150 MHz) NMR spectra of **4c** (chloroform-*d* at 298K)

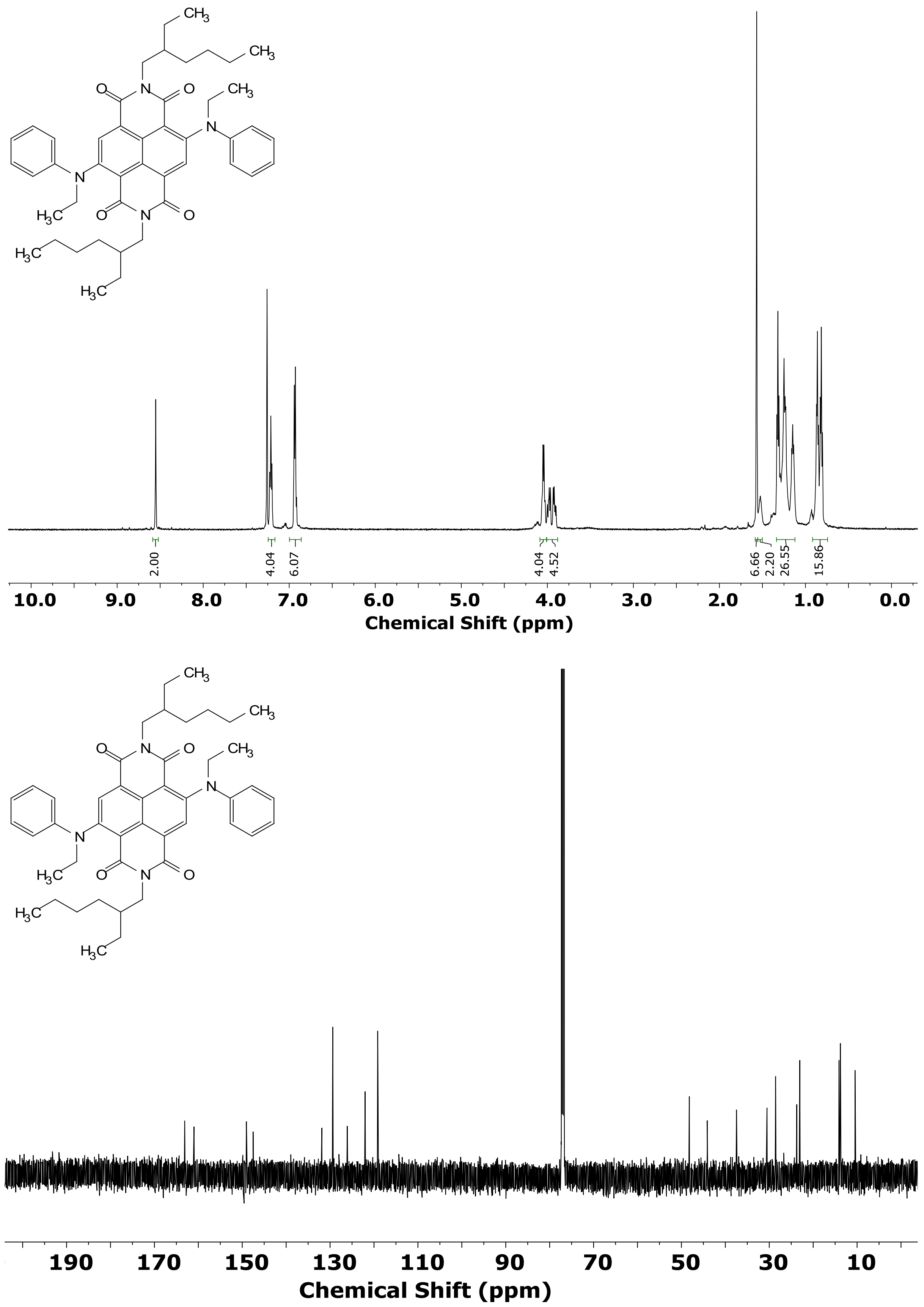

Figure S23: ^{1}H (600 MHz) and ^{13}C (150 MHz) NMR spectra of **4d** (chloroform-*d* at 298K)

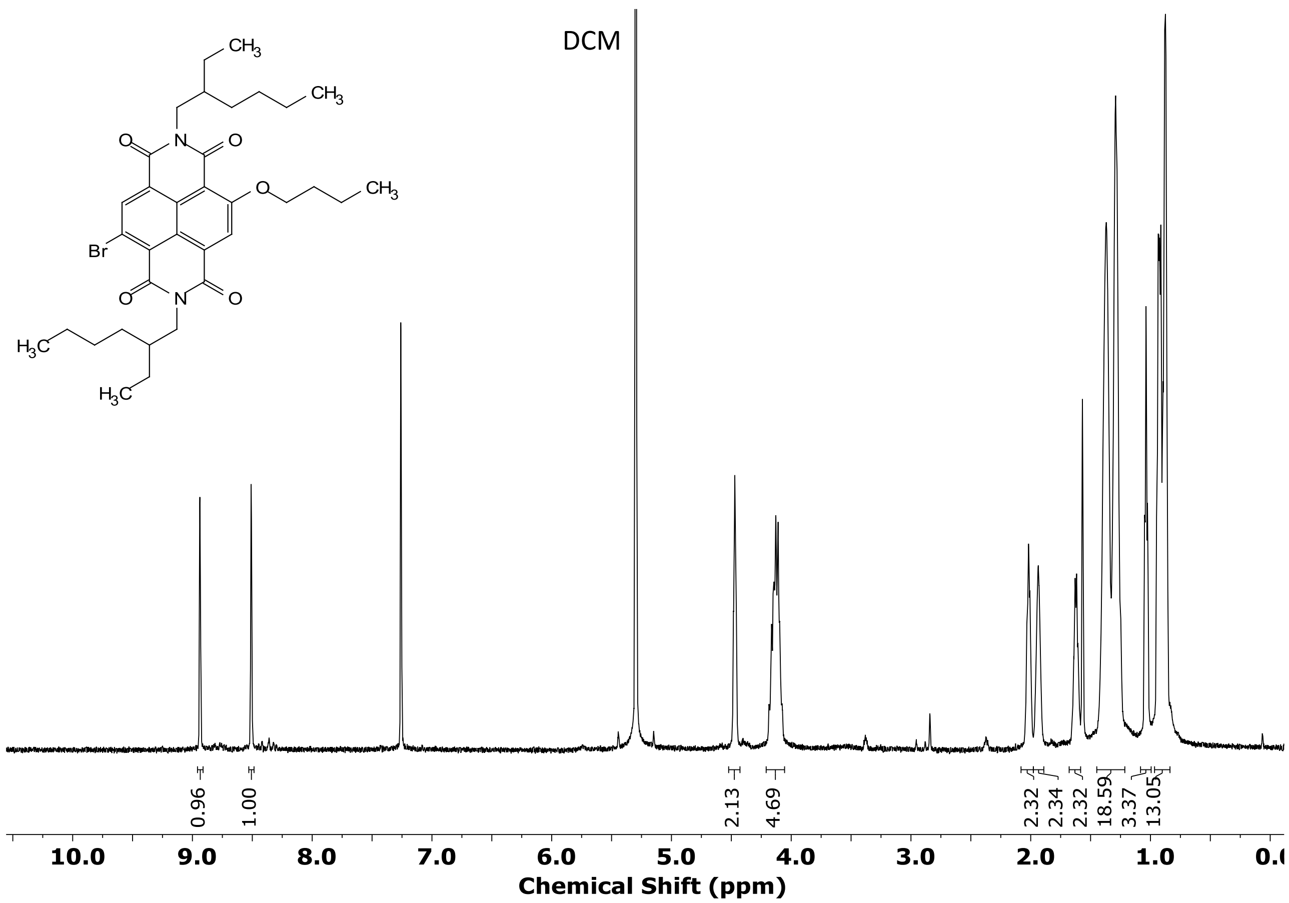

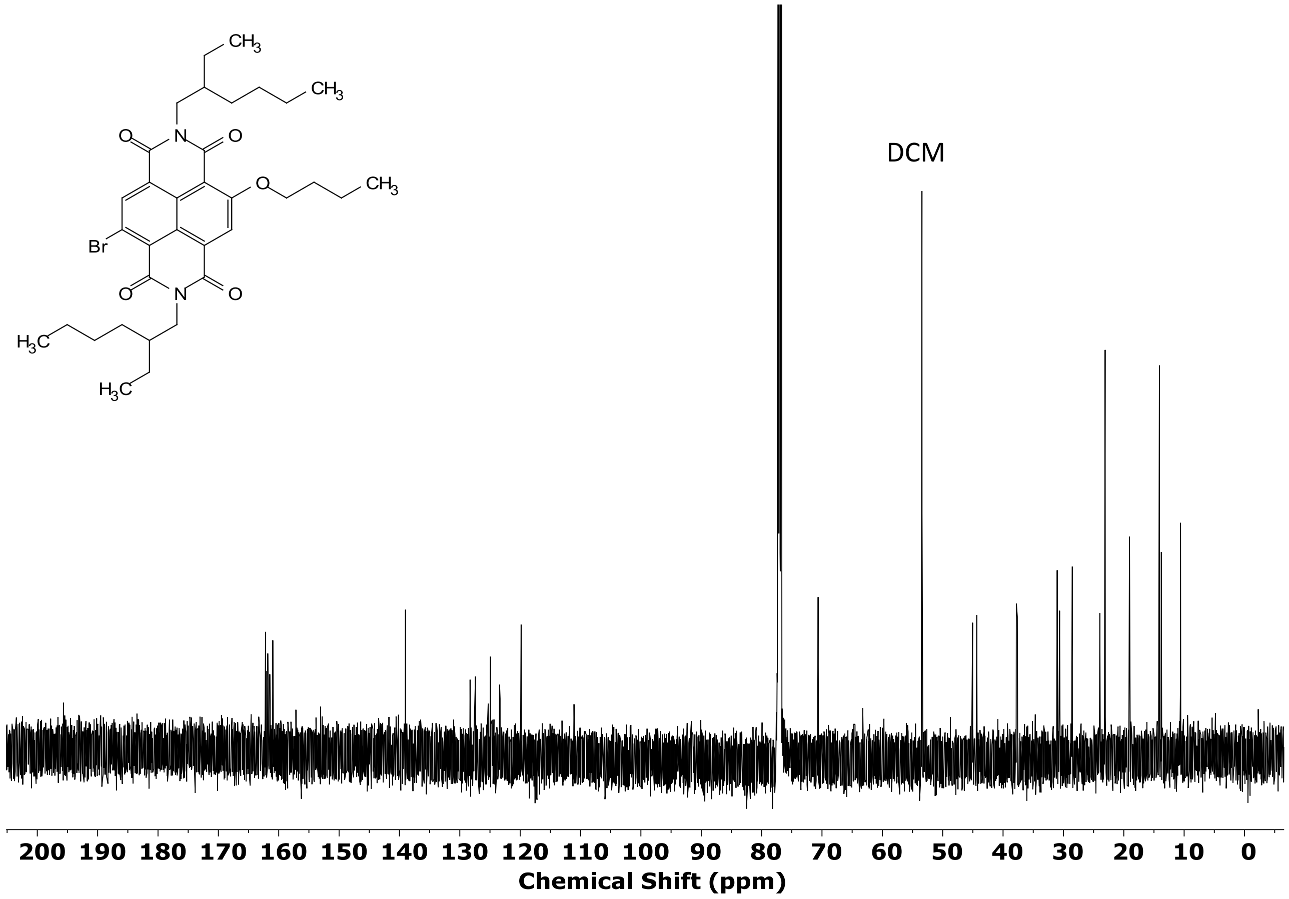

Figure S24: ^{1}H (600 MHz) and ^{13}C (150 MHz) NMR spectra of **5a** (chloroform-*d* at 298K)

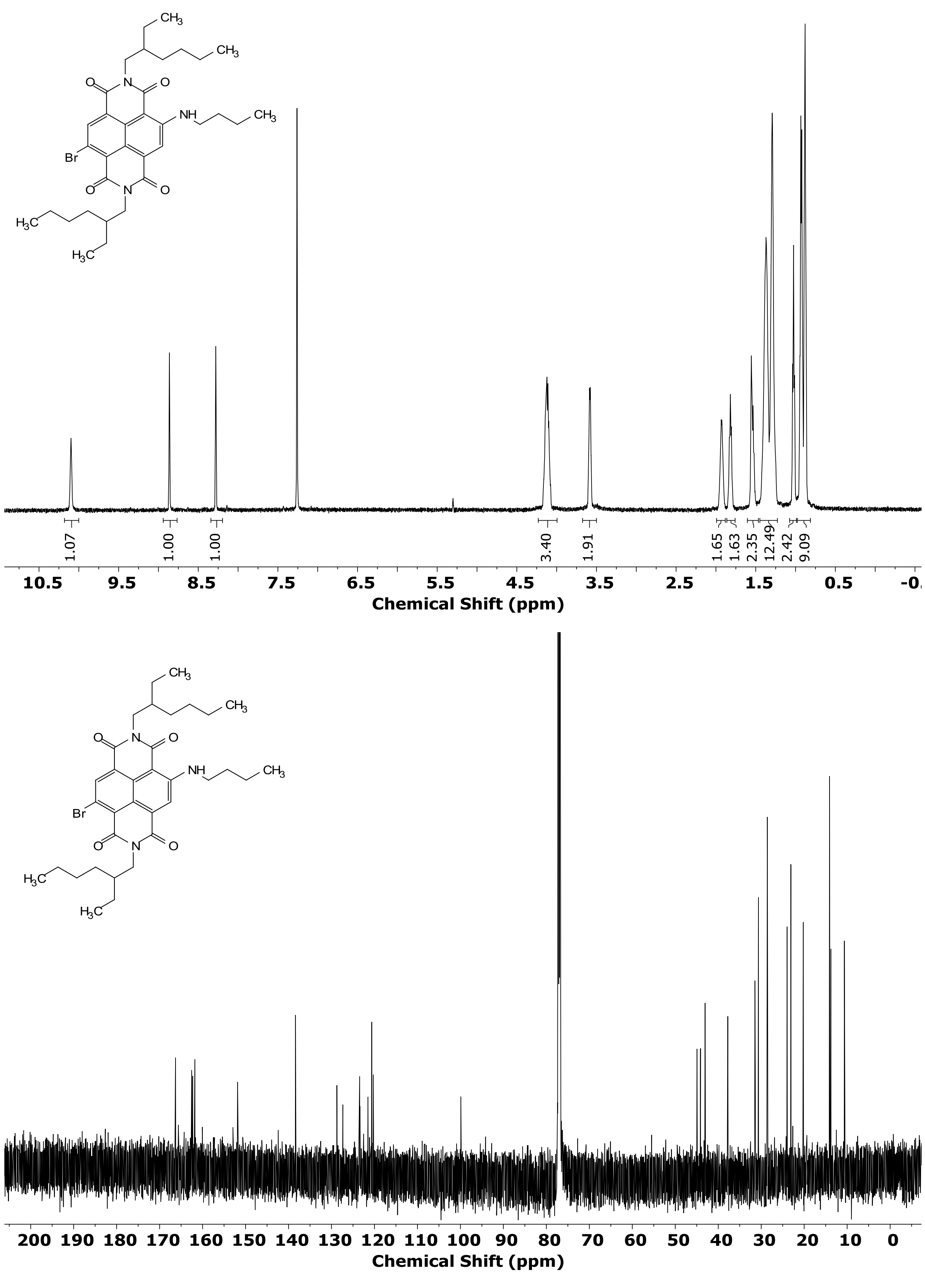

Figure S25: ^{1}H (600 MHz) and ^{13}C (150 MHz) NMR spectra of **5b** (chloroform-*d* at 298K)

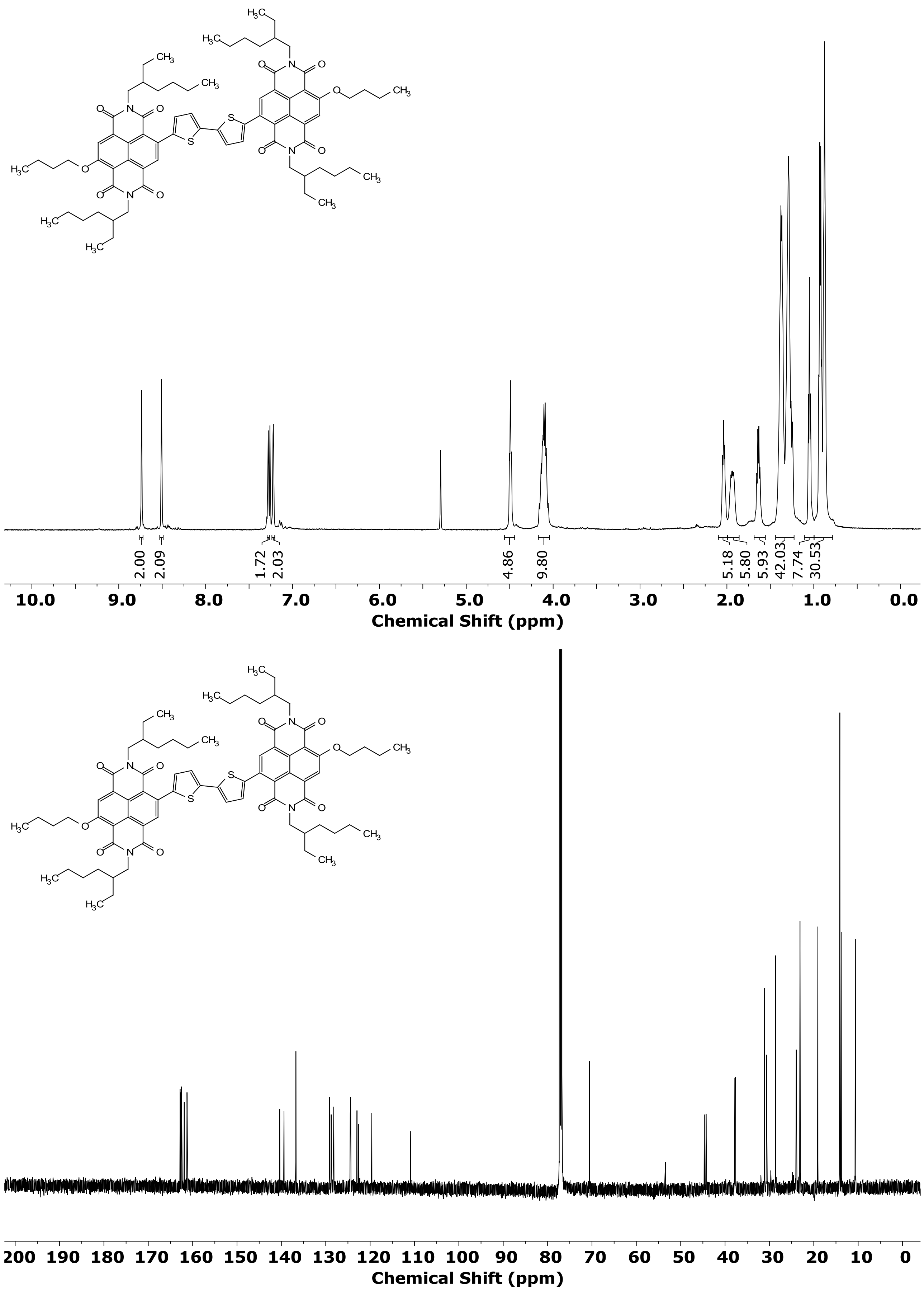

Figure S26: ^{1}H (600 MHz) and ^{13}C (150 MHz) NMR spectra of **6a** (chloroform-*d* at 298K)

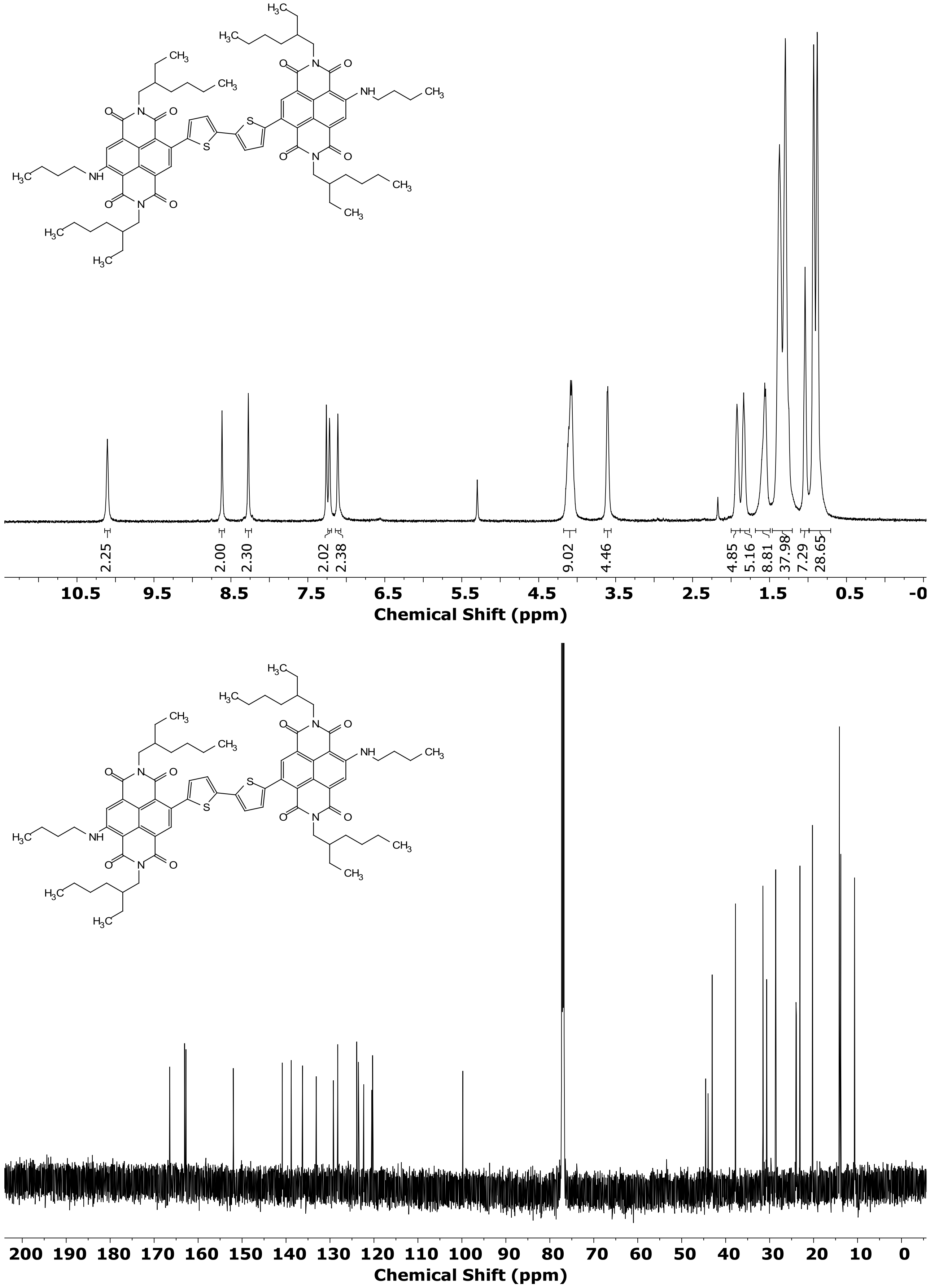

Figure S27: ^{1}H (600 MHz) and ^{13}C (150 MHz) NMR spectra of **6b** (chloroform-*d* at 298K)

S3.3 Mass Spectra

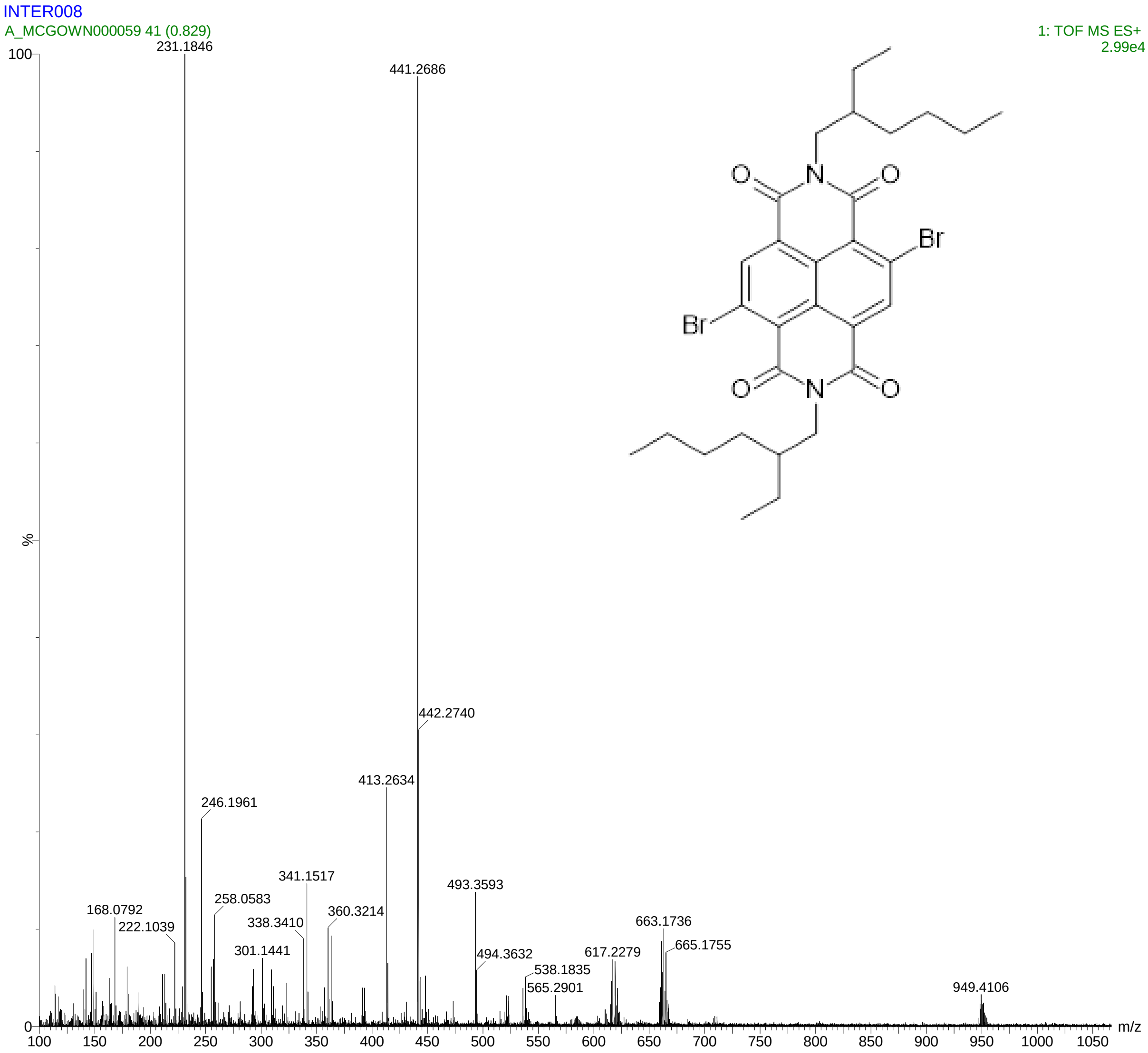

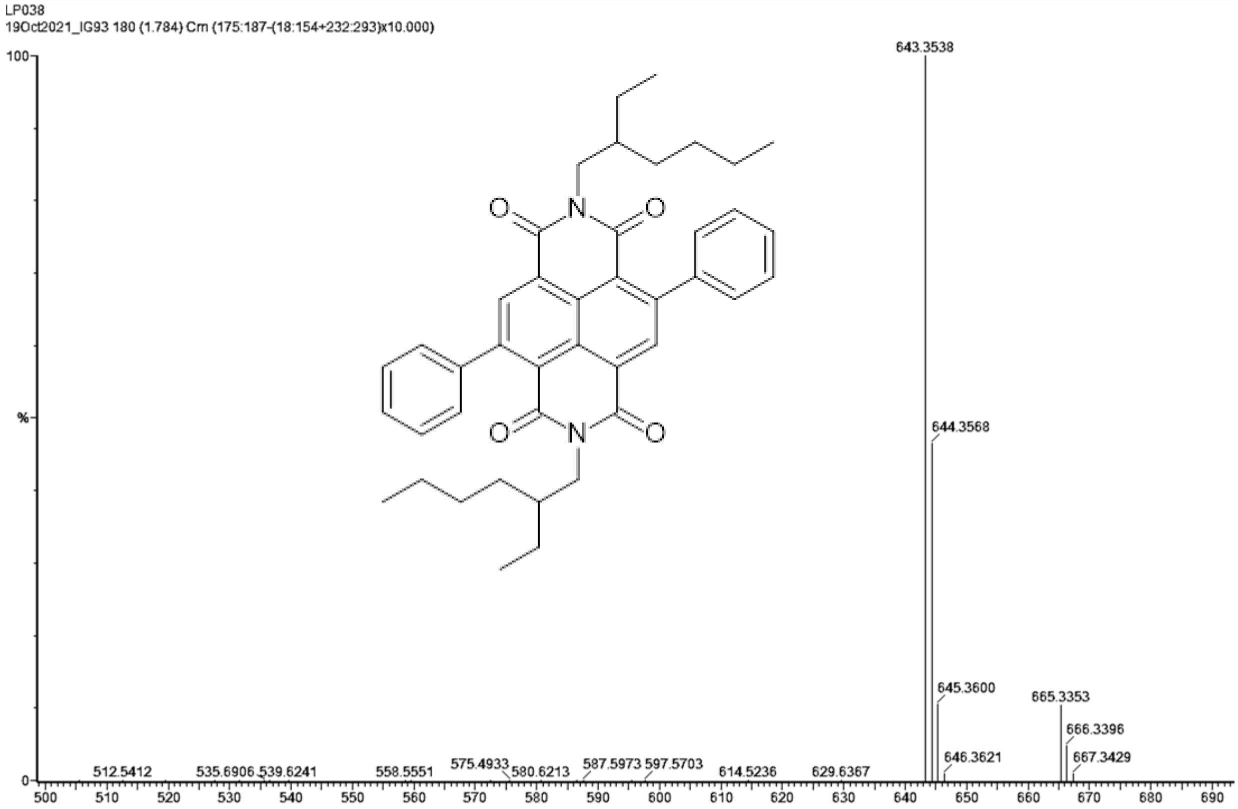
LP038
19Oct2021_IG93 180 (1.784) Cm (175:187-(18:154+232:293)x10.000)
643.3538
644.3568
645.3600
646.3621
665.3353
666.3396
667.3429
512.5412
535.6906
539.6241
558.5551
575.4933
580.6213
587.5973
597.5703
614.5236
629.6367
100
%
0

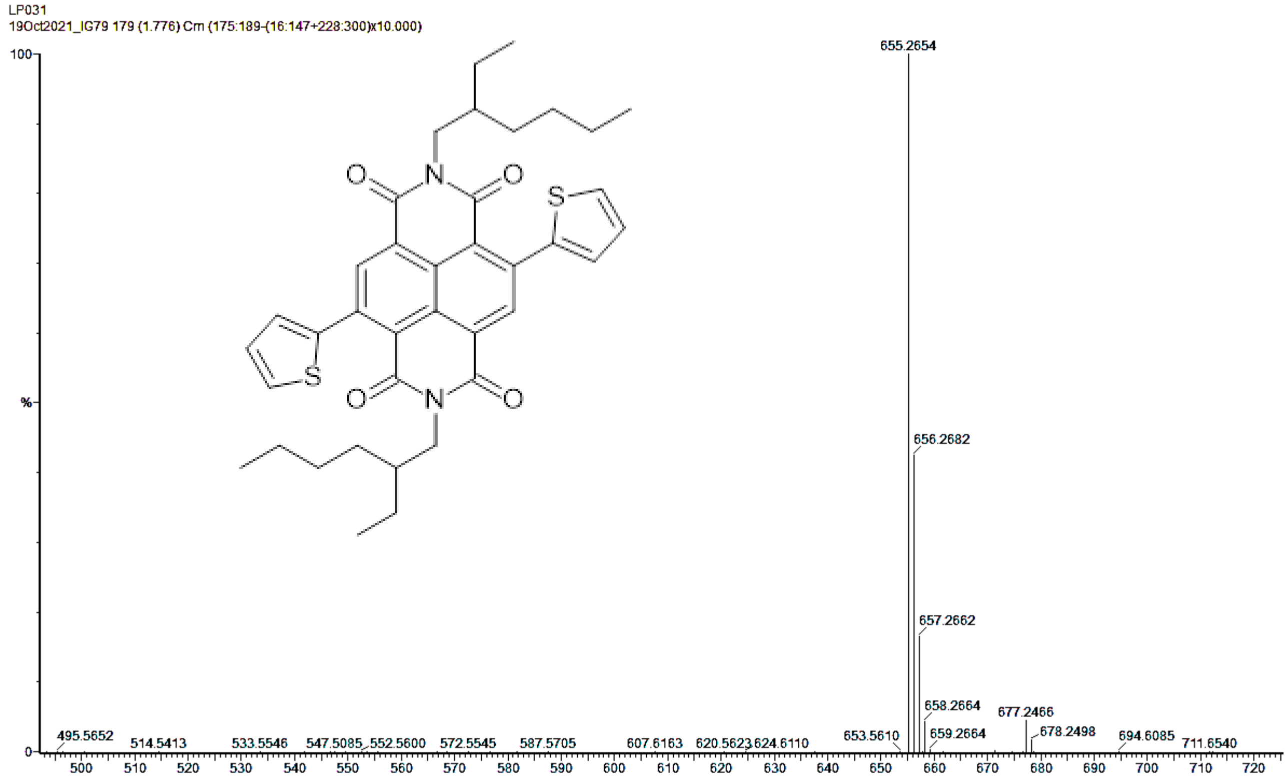
LP031
19Oct2021_IG79 179 (1.776) Cm (175:189-(16:147+228:300)x10.000)
655.2654
656.2682
657.2662
658.2664
659.2664
677.2466
678.2498
694.6085
711.6540
495.5652
514.5413
533.5546
547.5085
552.5600
572.5545
587.5705
607.6163
620.5623
624.6110
653.5610
100
%
0

Mass	Calc. Mass	mDa	PPM	DBE	Formula	i-FIT	i-FIT Norm	Fit Conf %	C	H	N	O
671.3859	671.3849	1.0	1.5	20.5	C44 H51 N2 O4	24.7	n/a	n/a	44	51	2	4

LP030
19Oct2021_IG77 184 (1.839) Cm (179:190-(38:166+217:302)x10.000)

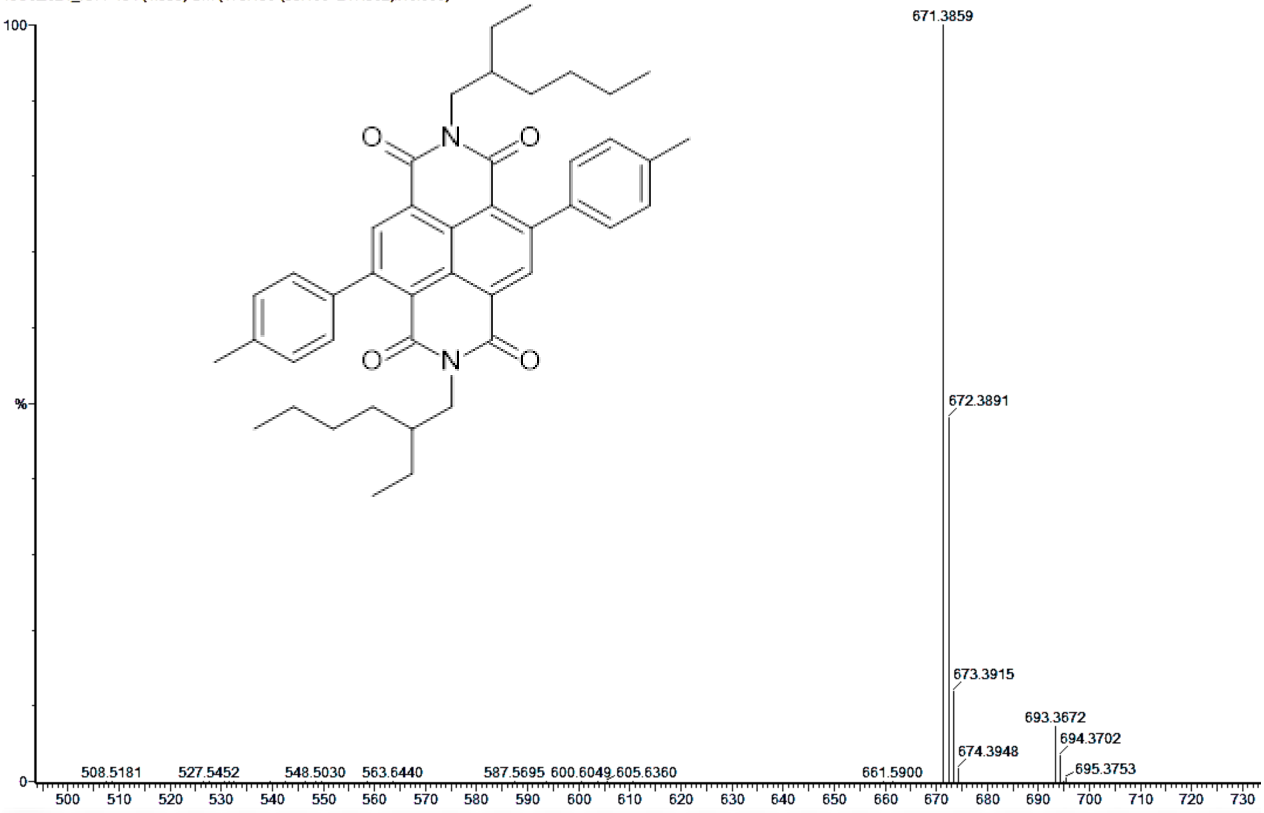

Mass	Calc. Mass	mDa	PPM	DBE	Formula	i-FIT	i-FIT Norm	Fit Conf %	C	H	N	O
671.3853	671.3849	0.4	0.6	20.5	C44 H51 N2 O4	28.9	n/a	n/a	44	51	2	4

LP029
19Oct2021_IG75 185 (1.848) Cm (183:194-16:162x10.000)

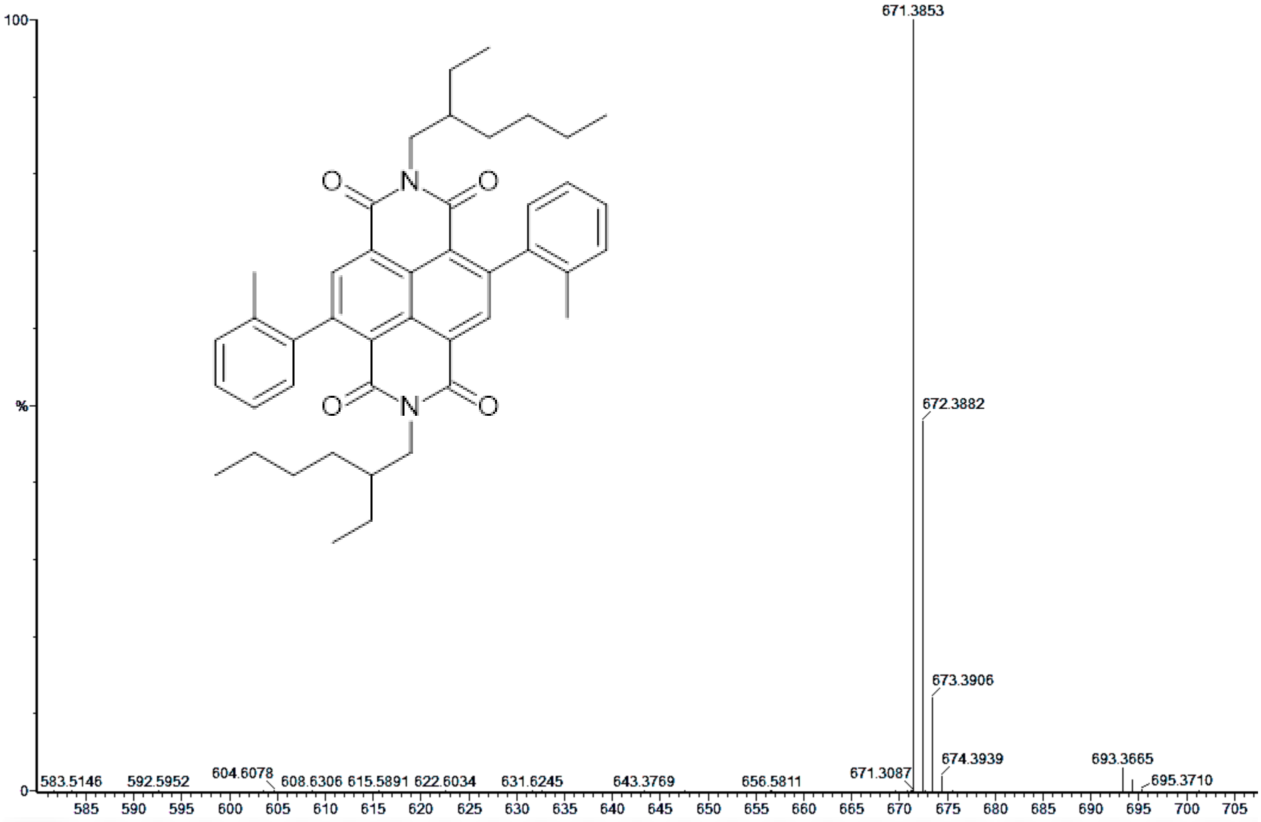

.P029
19Oct2021_IG75 185 (1.848) Cm (183:194-16:162x10.000)

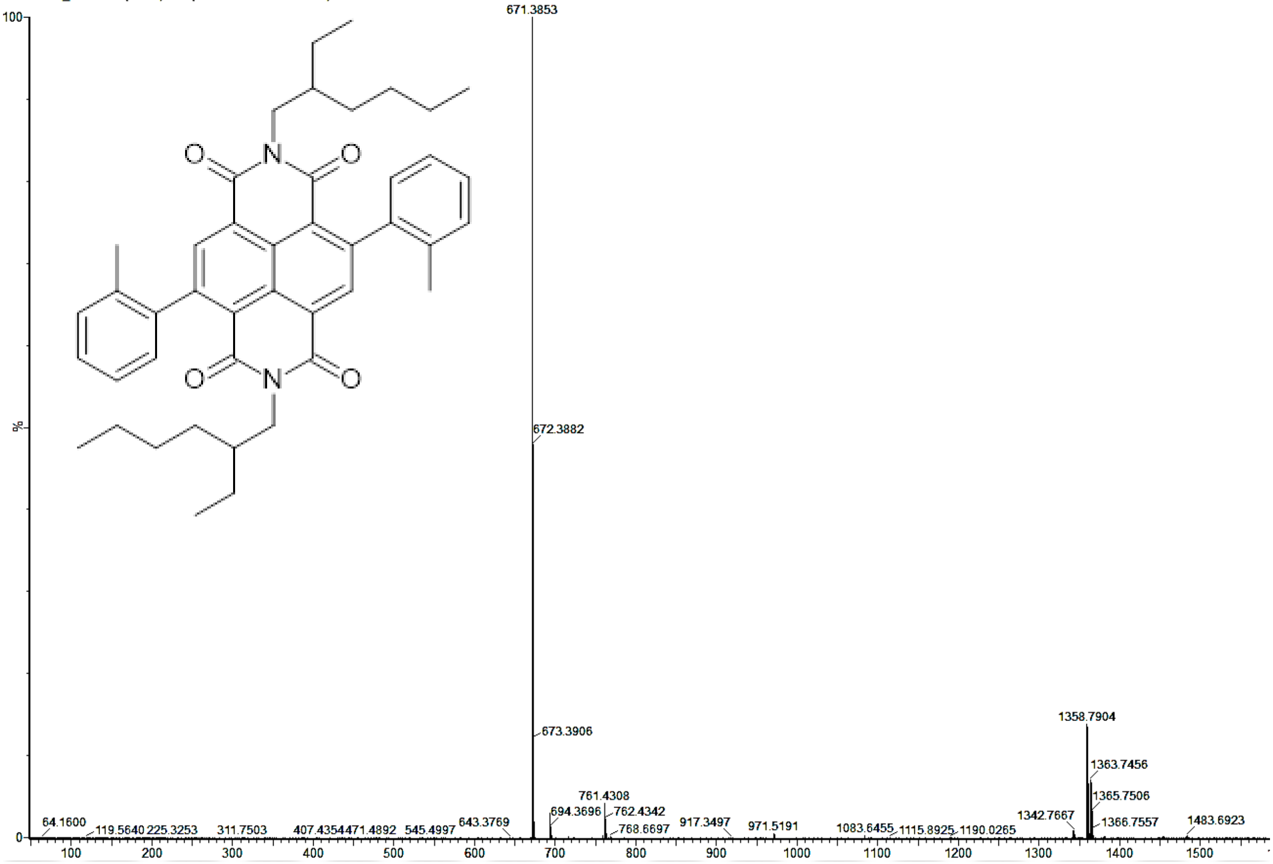

Mass	Calc. Mass	mDa	PPM	DBE	Formula	i-FIT	i-FIT Norm	Fit Conf %	C	H	N	O	S
683.2972	683.2977	-0.5	-0.7	18.5	C40 H47 N2 O4 S2	35.4	n/a	n/a	40	47	2	4	2

LP032
19Oct2021_IG81 186 (1.856) Cm (183:195-(19:159+236:290)x10.000)

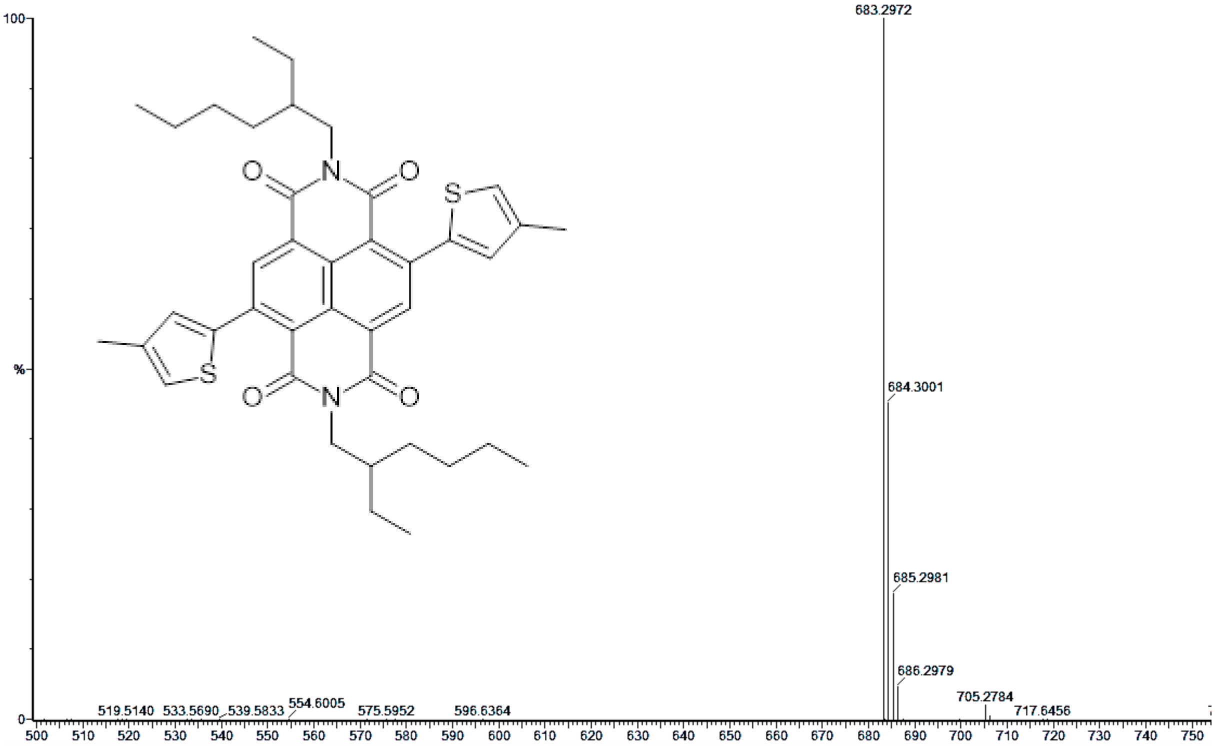

Mass	Calc. Mass	mDa	PPM	DBE	Formula	i-FIT	i-FIT Norm	Fit Conf %	C	H	N	O
703.3740	703.3747	-0.7	-1.0	20.5	C44 H51 N2 O6	29.1	n/a	n/a	44	51	2	6

LP034
19Oct2021_IG85 169 (1.688) Cm (168:180-(22:149+217:288)x10.000)

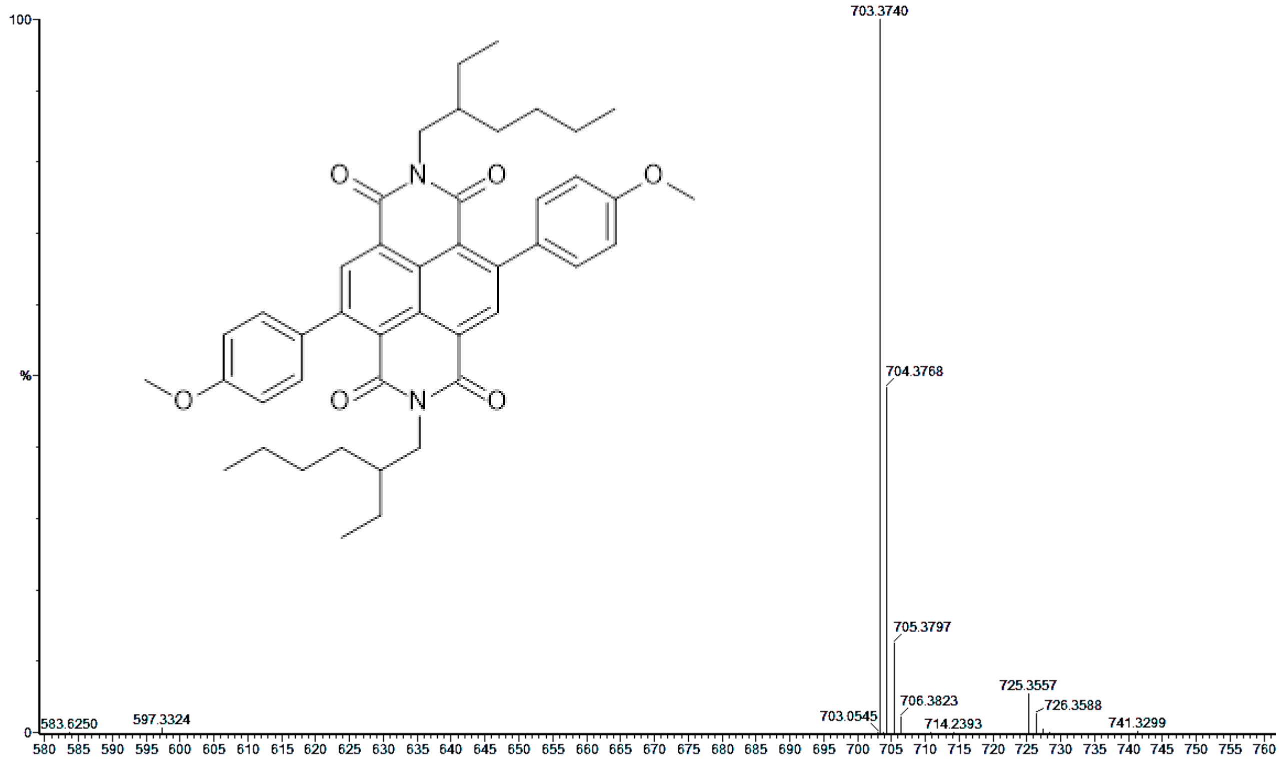

Mass	Calc. Mass	mDa	PPM	DBE	Formula	i-FIT	i-FIT Norm	Fit Conf %	C	H	N	O
743.3852	743.3849	0.3	0.4	26.5	C50 H51 N2 O4	23.6	n/a	n/a	50	51	2	4

_P036
19Oct2021_IG89 187 (1.865) Cm (181:192-(19:163+234:291)x10.000)

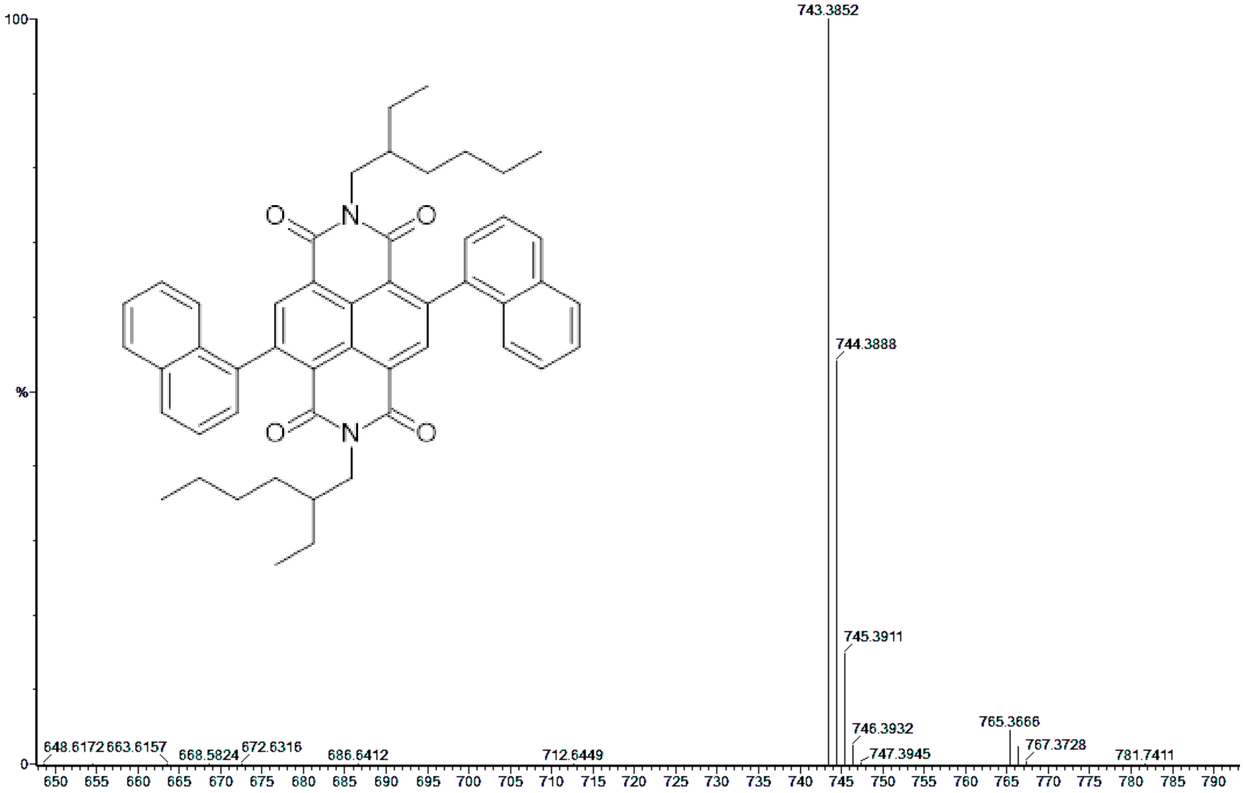

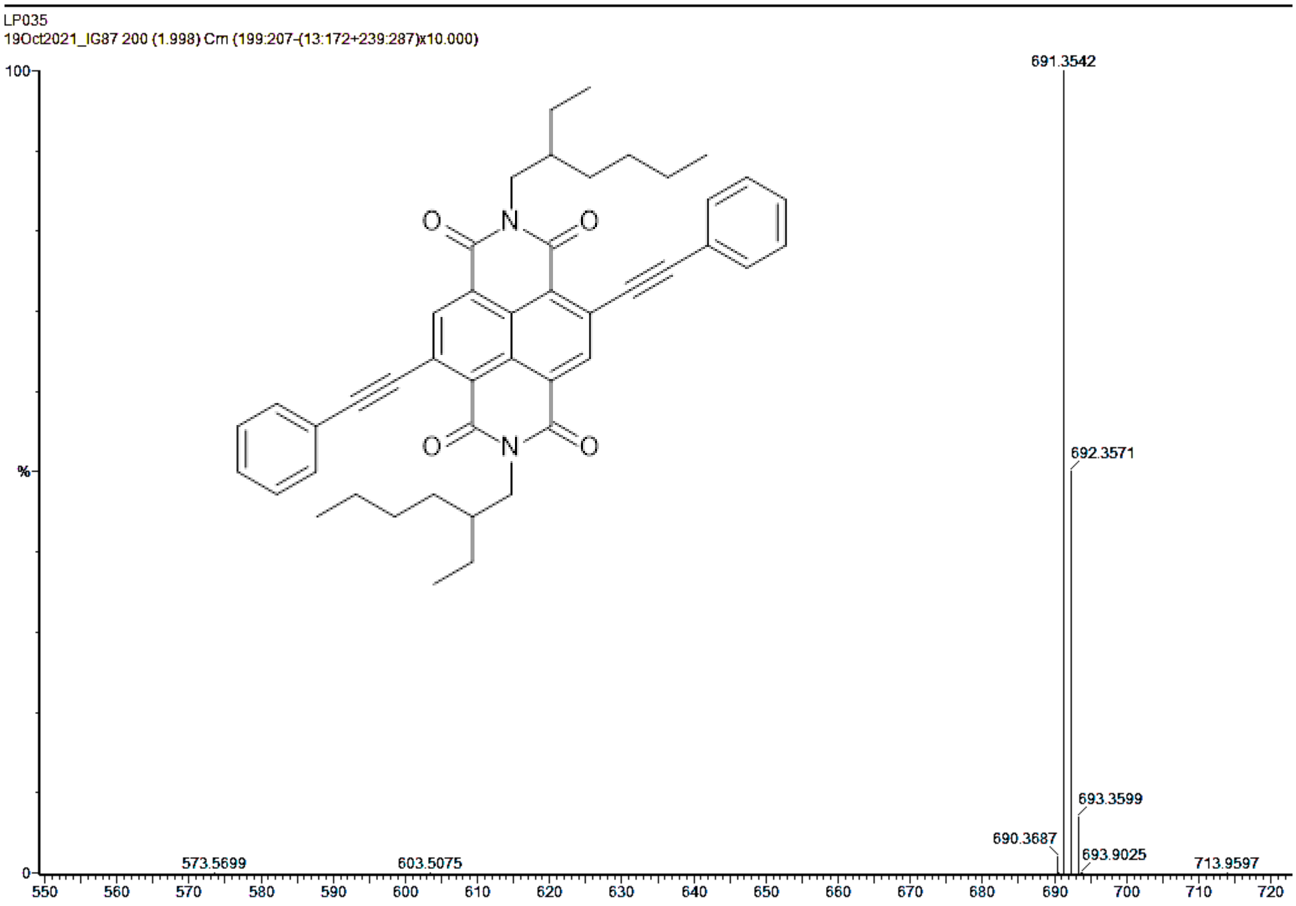
LP035
19Oct2021_IG87 200 (1.998) Cm (199:207-(13:172+239:287)x10.000)
100
%
0
691.3542
692.3571
693.3599
690.3687
693.9025
713.9597
573.5699
603.5075
O
N
550 560 570 580 590 600 610 620 630 640 650 660 670 680 690 700 710 720

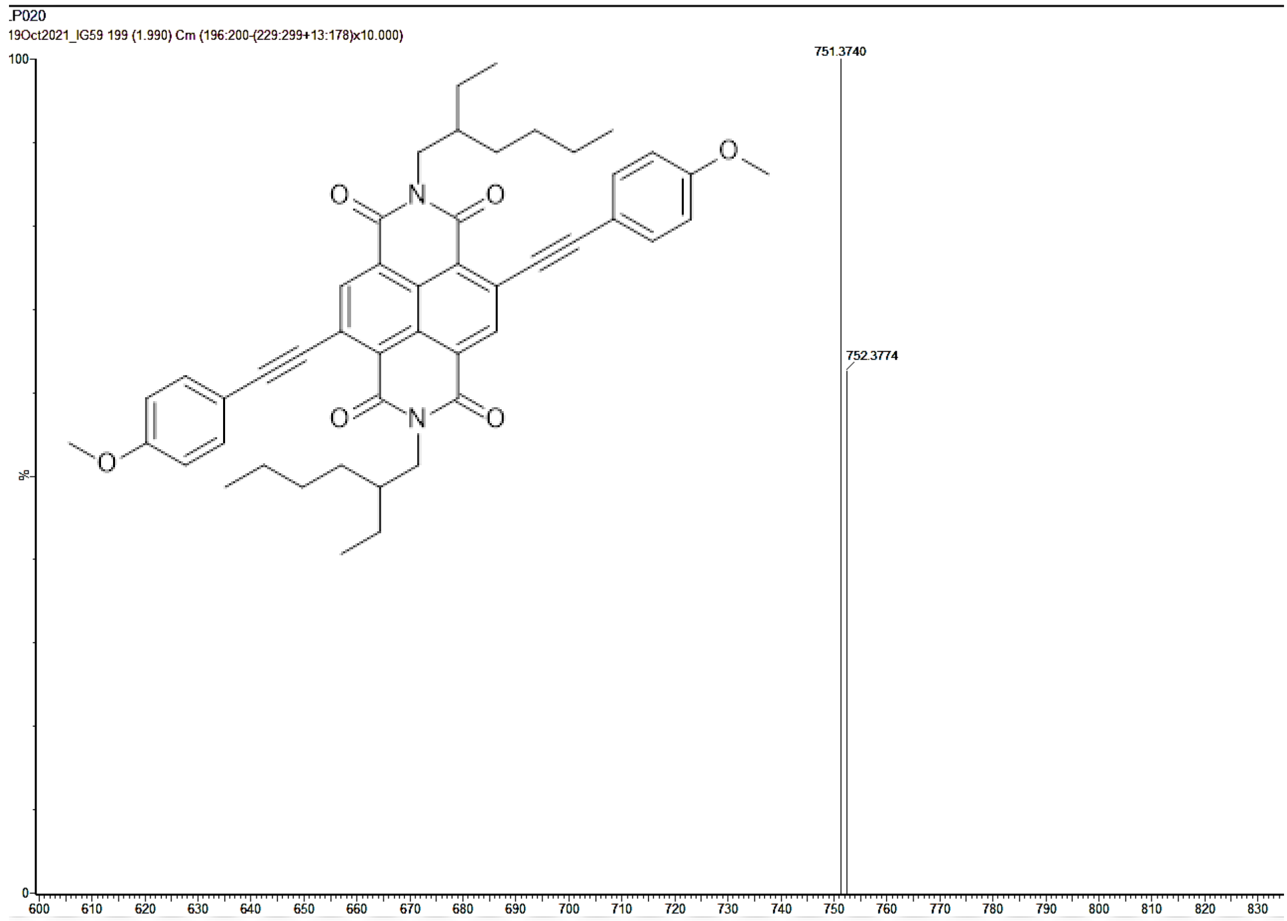
.P020
19Oct2021_IG59 199 (1.990) Cm (196:200-(229:299+13:178)x10.000)
100
%
0
751.3740
752.3774
O
N
600 610 620 630 640 650 660 670 680 690 700 710 720 730 740 750 760 770 780 790 800 810 820 830

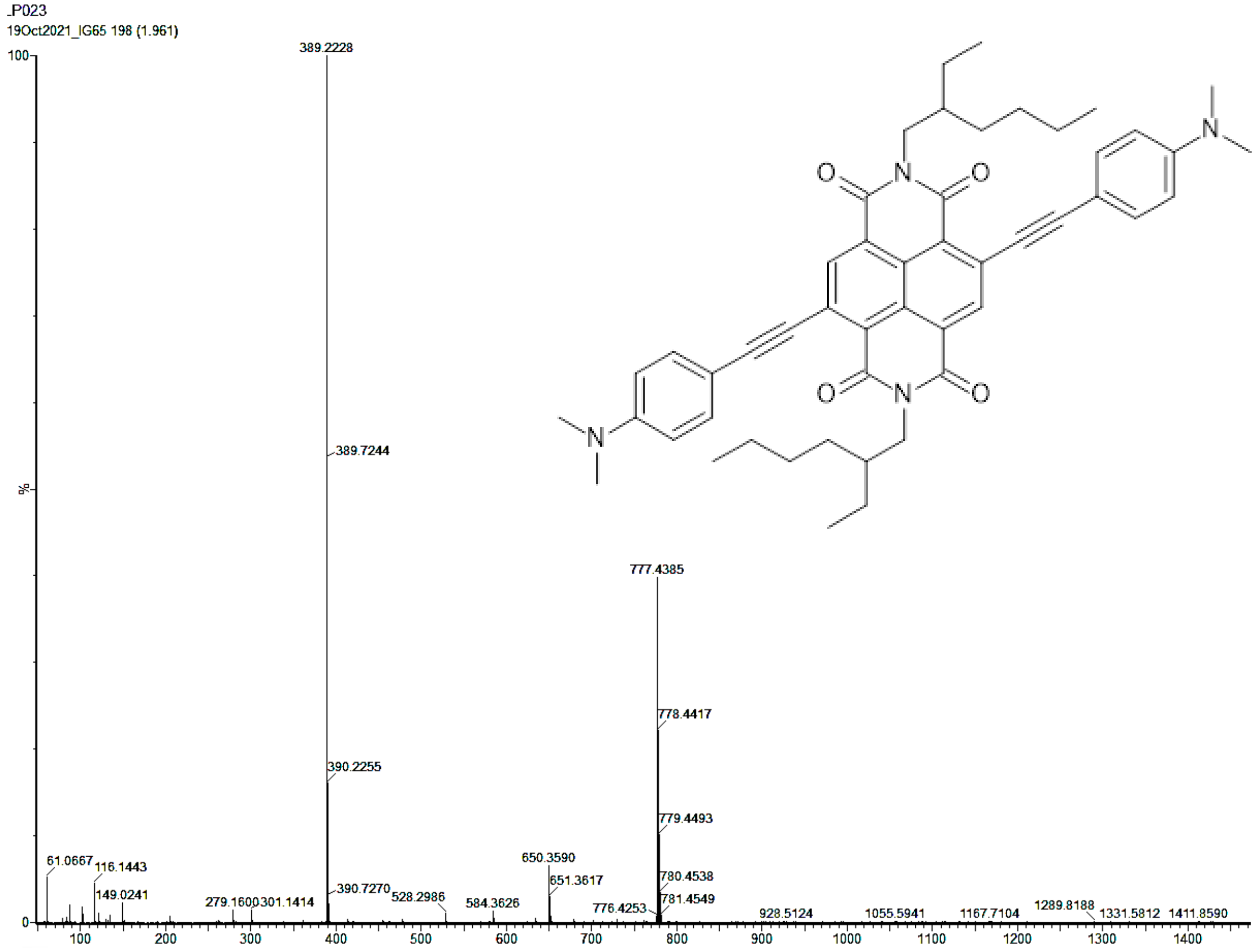
_P023
19Oct2021_IG65 198 (1.961)
100
%
0
389.2228
389.7244
390.2255
390.7270
777.4385
778.4417
779.4493
780.4538
781.4549
776.4253
650.3590
651.3617
61.0667
116.1443
149.0241
279.1600
301.1414
528.2986
584.3626
928.5124
1055.5941
1167.7104
1289.8188
1331.5812
1411.8590
100 200 300 400 500 600 700 800 900 1000 1100 1200 1300 1400

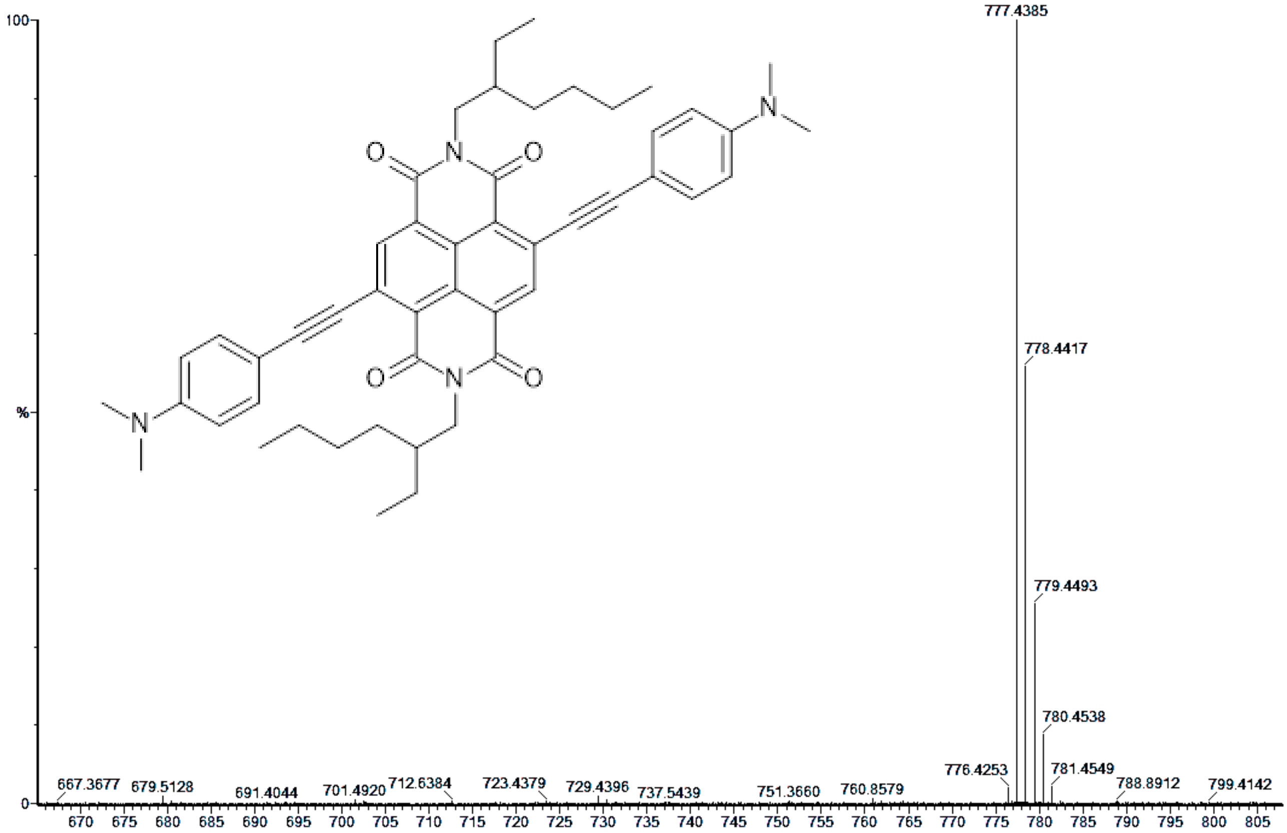
100
%
0
777.4385
778.4417
779.4493
780.4538
781.4549
776.4253
667.3677
679.5128
691.4044
701.4920
712.6384
723.4379
729.4396
737.5439
751.3660
760.8579
788.8912
799.4142
670 675 680 685 690 695 700 705 710 715 720 725 730 735 740 745 750 755 760 765 770 775 780 785 790 795 800 805

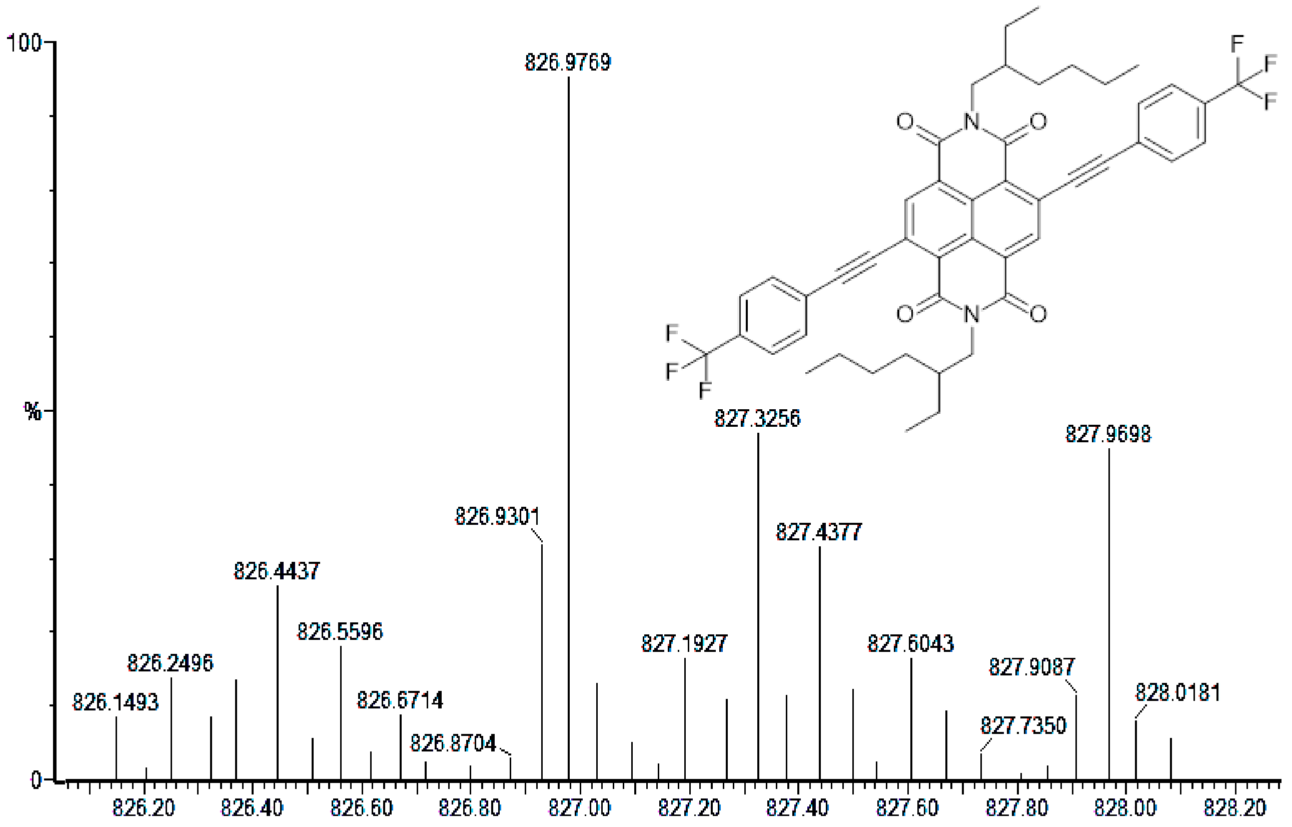

100
%
0
826.9769
827.3256
827.9698
826.9301
827.4377
826.4437
826.5596
827.1927
827.6043
826.2496
827.9087
828.0181
826.1493
826.6714
827.7350
826.8704
826.20
826.40
826.60
826.80
827.00
827.20
827.40
827.60
827.80
828.00
828.20
F
F
F
O
N
O
O
N
O
F
F
F

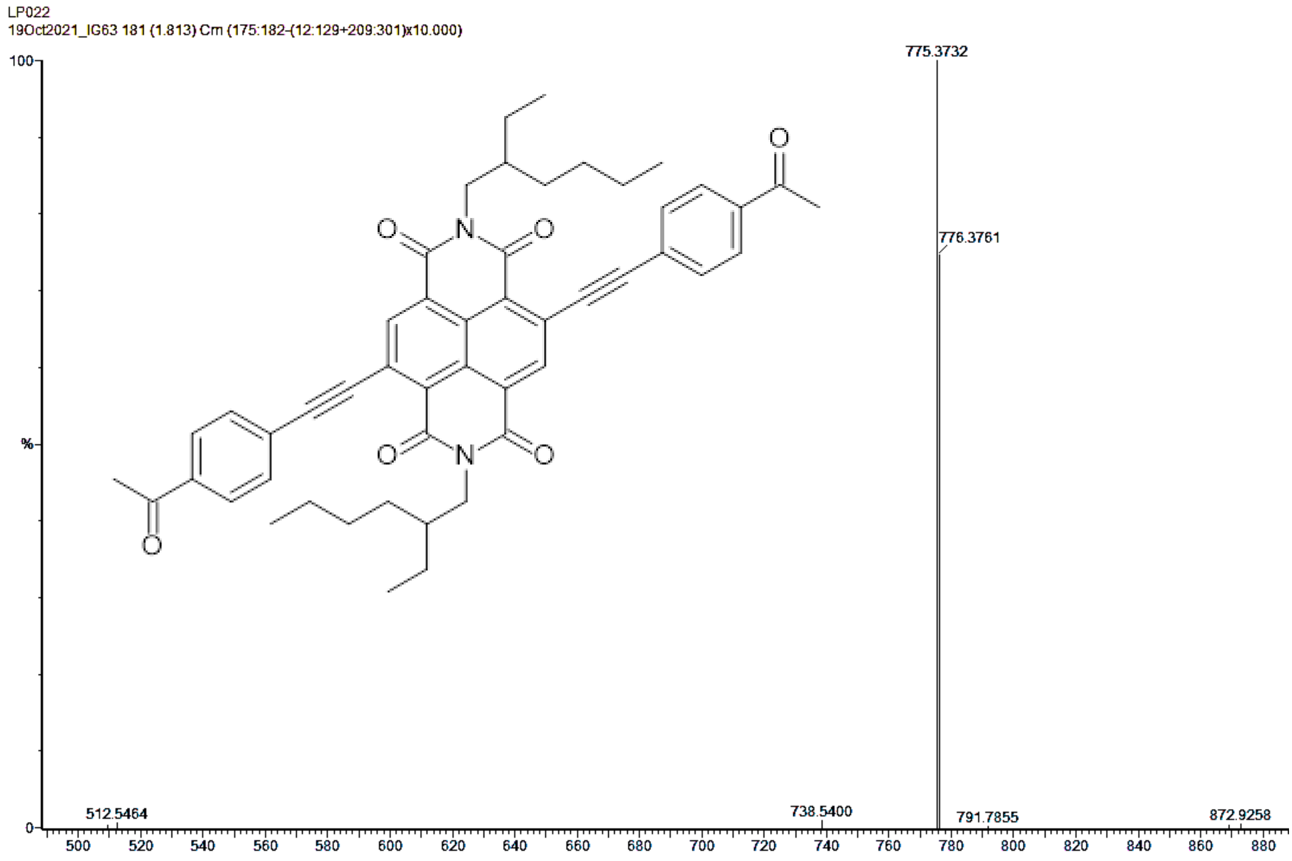

LP022
19Oct2021_IG63 181 (1.813) Cm (175:182-(12:129+209:301)x10.000)
100
%
0
775.3732
776.3761
512.5464
738.5400
791.7855
872.9258
500
520
540
560
580
600
620
640
660
680
700
720
740
760
780
800
820
840
860
880
O
O
N
O
O
N
O
O

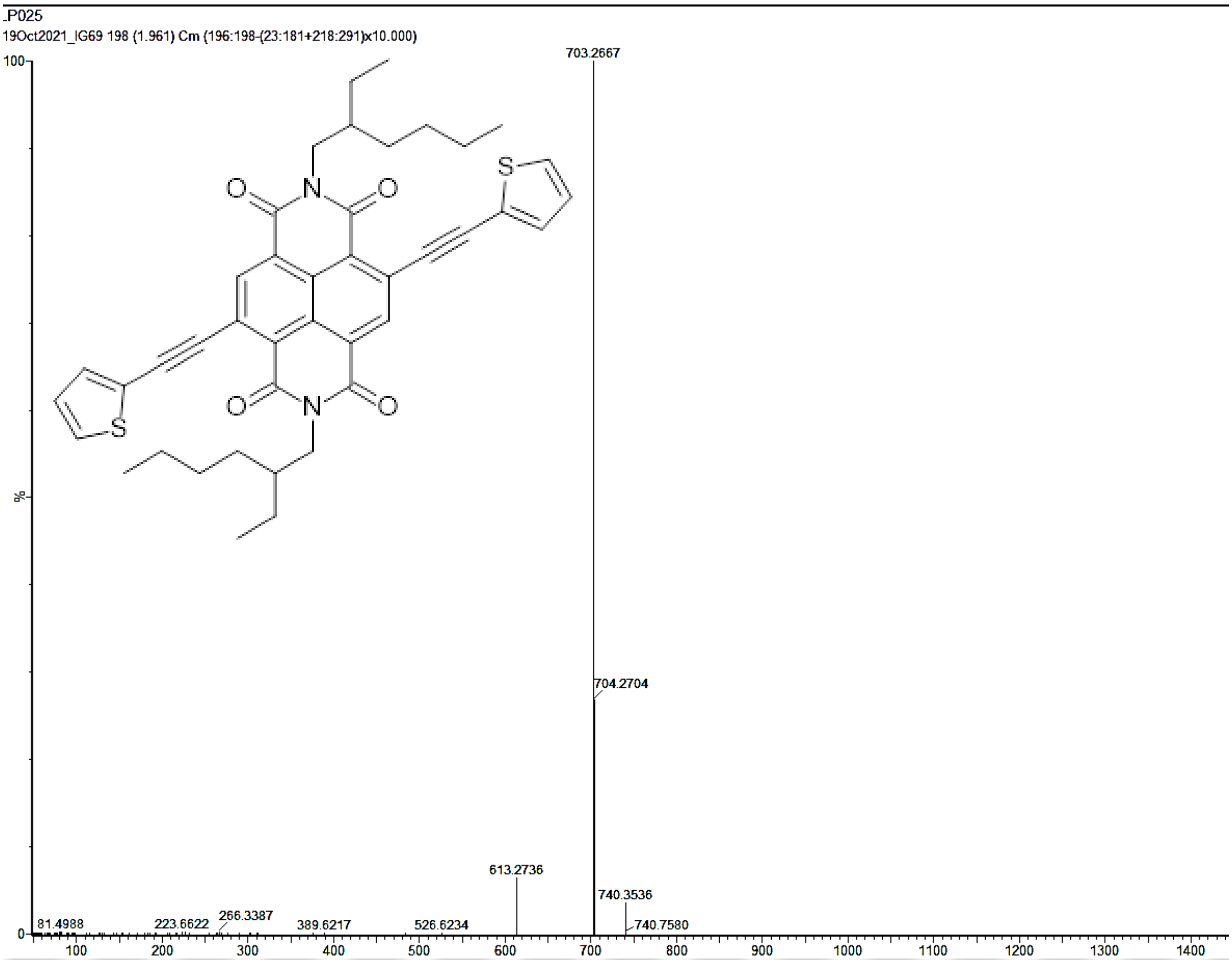
_P025
19Oct2021_IG69 198 (1.961) Cm (196:198-(23:181+218:291)x10.000)
703.2667
704.2704
613.2736
740.3536
740.7580
81.4988
223.6622
266.3387
389.6217
526.6234
%

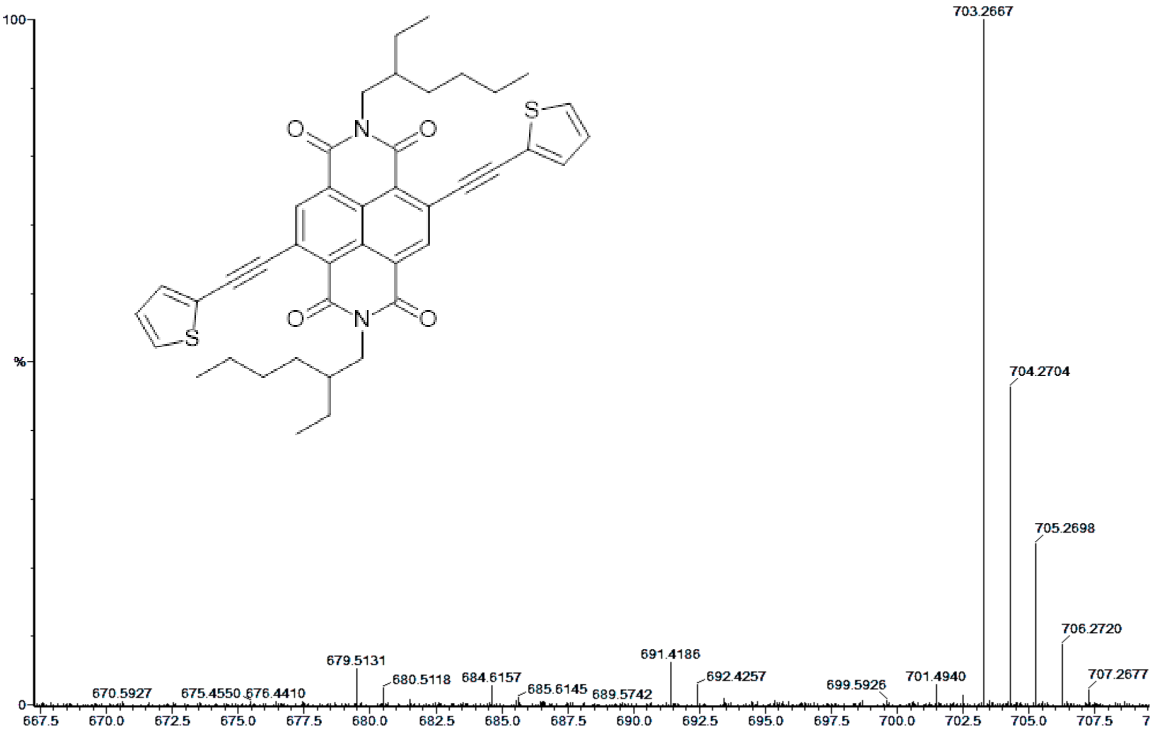
703.2667
704.2704
705.2698
706.2720
707.2677
679.5131
680.5118
684.6157
685.6145
689.5742
691.4186
692.4257
699.5926
701.4940
670.5927
675.4550
676.4410
%

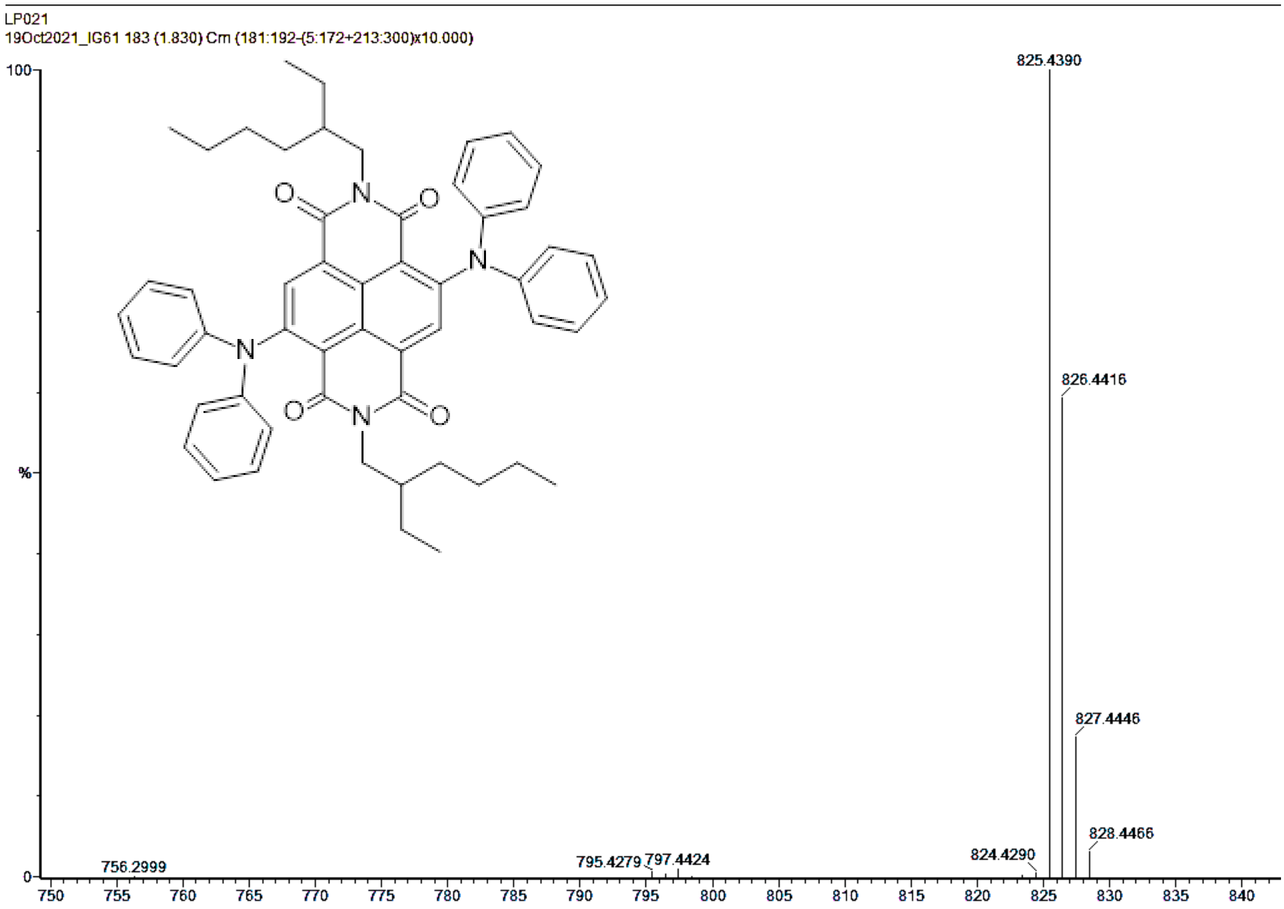
LP021
19Oct2021_IG61 183 (1.830) Cm (181:192-(5:172+213:300)x10.000)
825.4390
826.4416
827.4446
828.4466
824.4290
795.4279
797.4424
756.2999
100
%
0
750
755
760
765
770
775
780
785
790
795
800
805
810
815
820
825
830
835
840

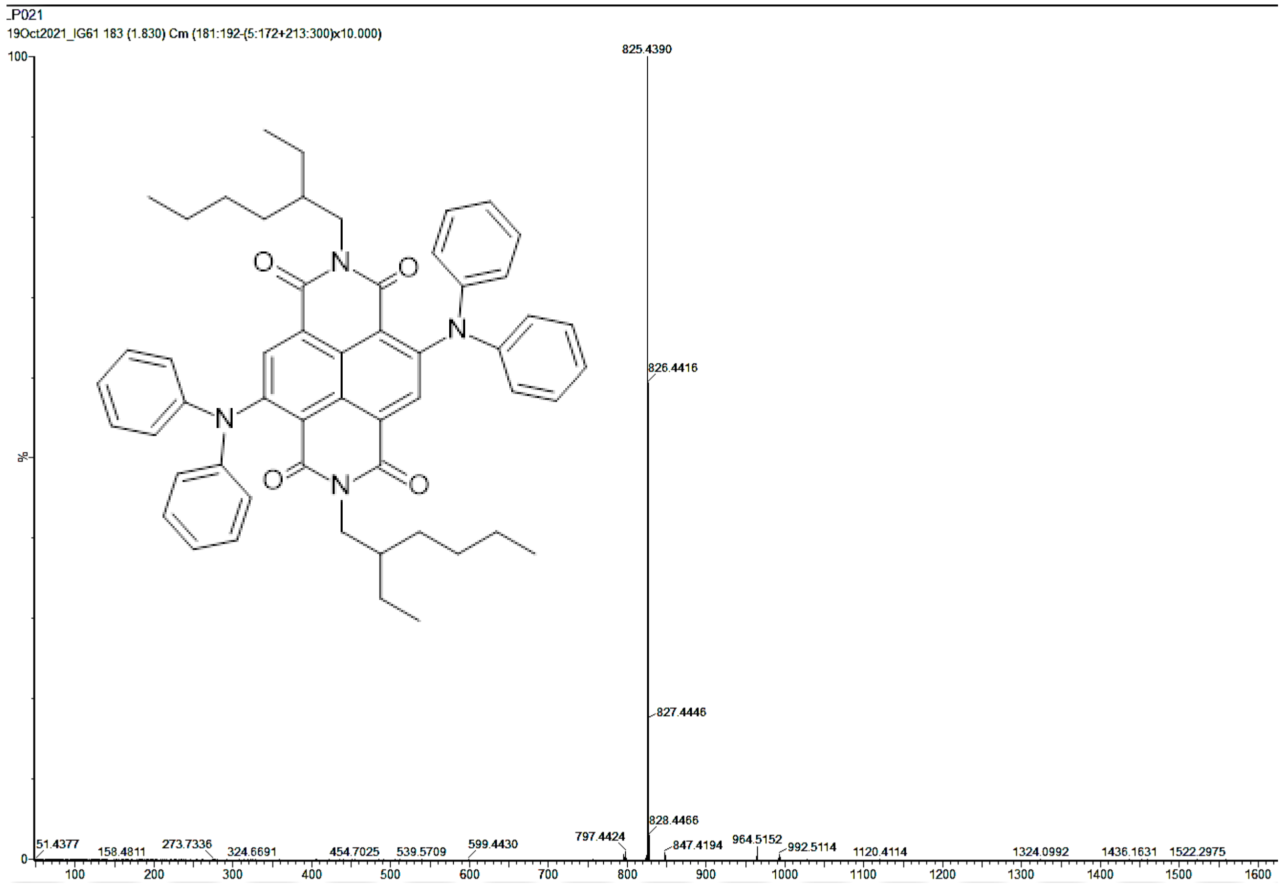
LP021
19Oct2021_IG61 183 (1.830) Cm (181:192-(5:172+213:300)x10.000)
825.4390
826.4416
827.4446
828.4466
847.4194
964.5152
992.5114
1120.4114
1324.0992
1436.1631
1522.2975
797.4424
599.4430
539.5709
454.7025
324.6691
273.7336
158.4811
51.4377
100
%
0
100
200
300
400
500
600
700
800
900
1000
1100
1200
1300
1400
1500
1600

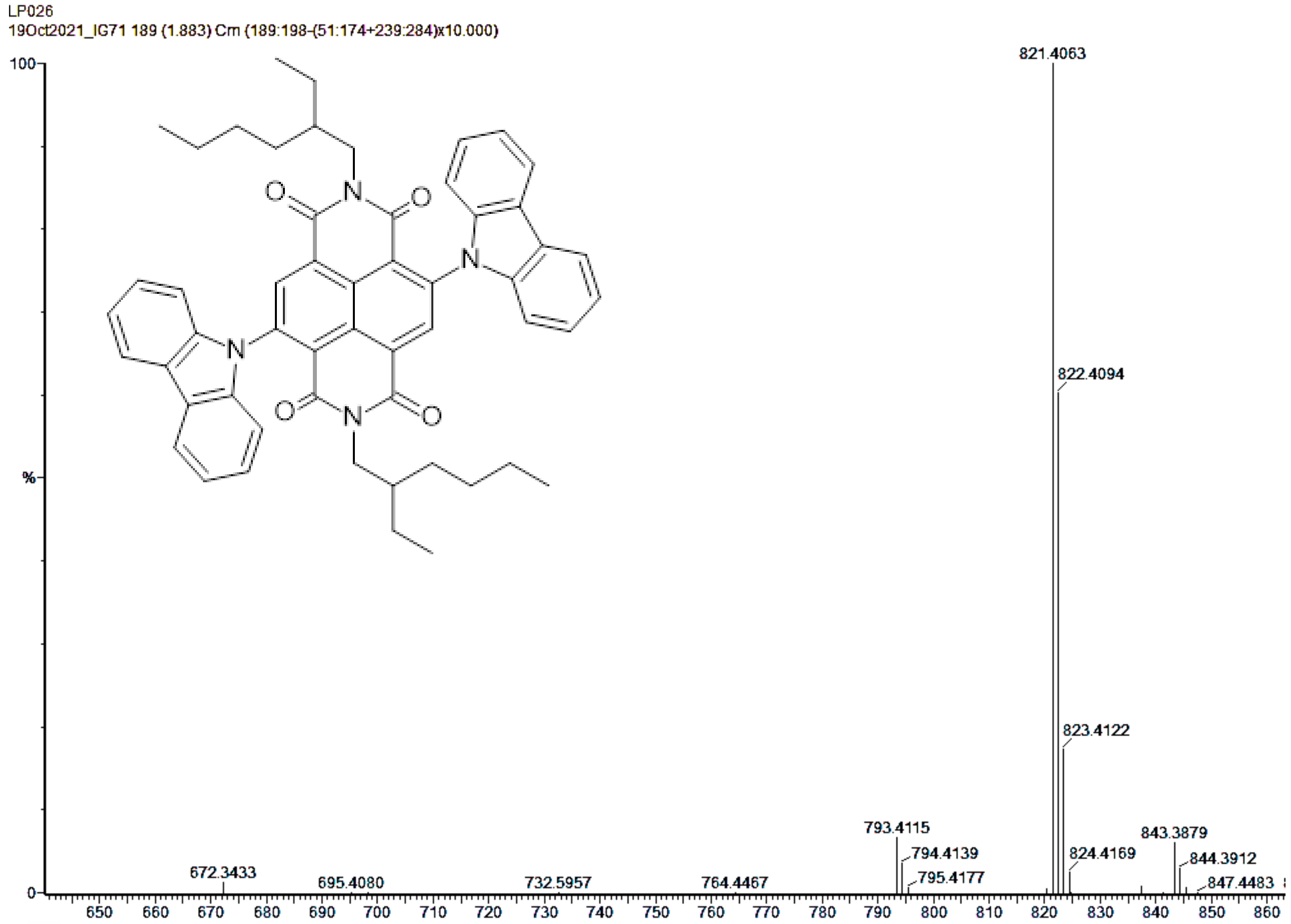
LP026
19Oct2021_IG71 189 (1.883) Cm (189:198-(51:174+239:284)x10.000)
100
%
0
821.4063
822.4094
823.4122
793.4115
794.4139
795.4177
824.4169
843.3879
844.3912
847.4483
672.3433
695.4080
732.5957
764.4467
650
660
670
680
690
700
710
720
730
740
750
760
770
780
790
800
810
820
830
840
850
860
N
O

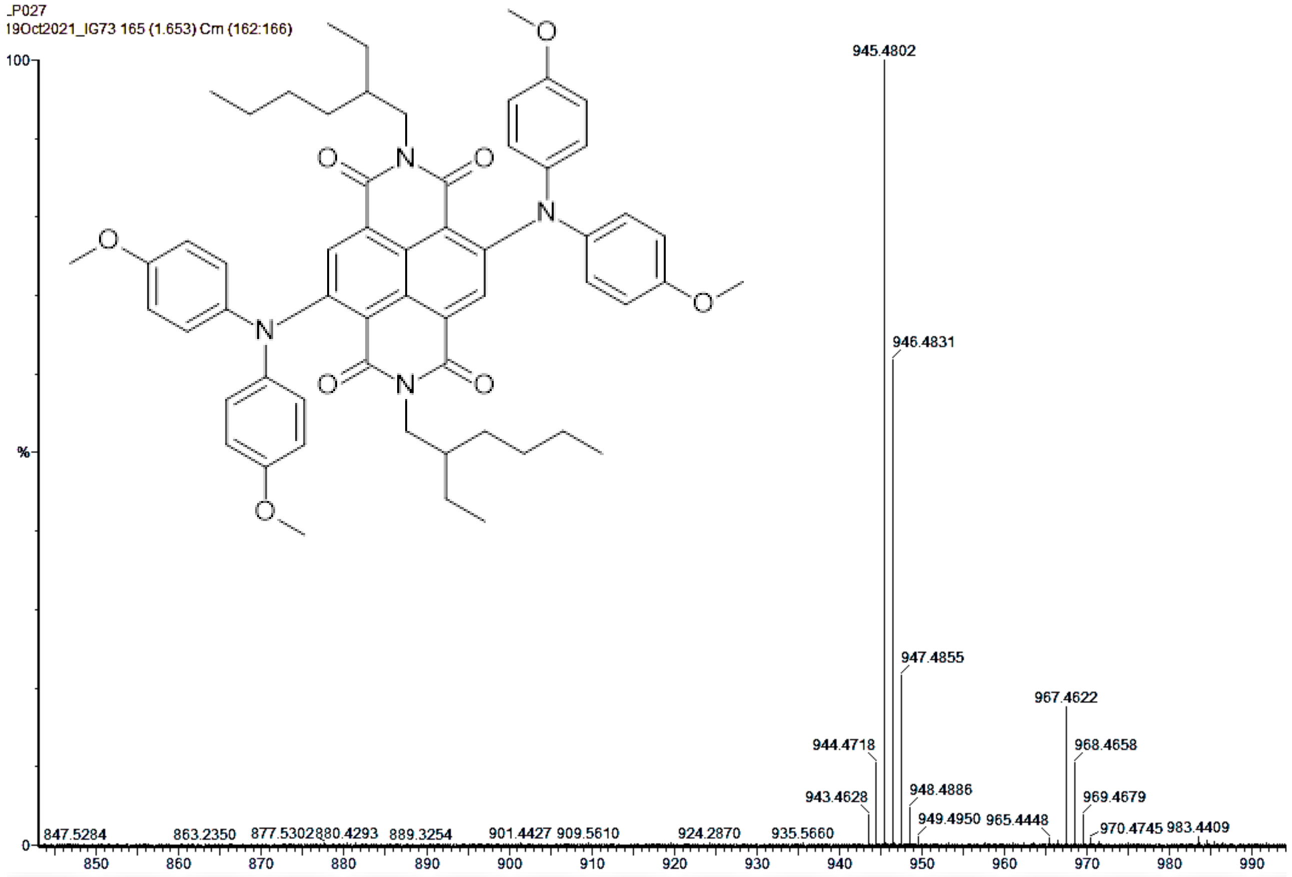
_P027
19Oct2021_IG73 165 (1.653) Cm (162:166)
100
%
0
945.4802
946.4831
947.4855
967.4622
944.4718
968.4658
943.4628
948.4886
969.4679
949.4950
965.4448
970.4745
983.4409
847.5284
863.2350
877.5302
880.4293
889.3254
901.4427
909.5610
924.2870
935.5660
850
860
870
880
890
900
910
920
930
940
950
960
970
980
990
N
O

Mass	Calc. Mass	mDa	PPM	DBE	Formula	i-FIT	i-FIT Norm	Fit Conf %	C	H	N	O
729.4368	729.4380	-1.2	-1.6	20.5	C46 H57 N4 O4	450.9	n/a	n/a	46	57	4	4

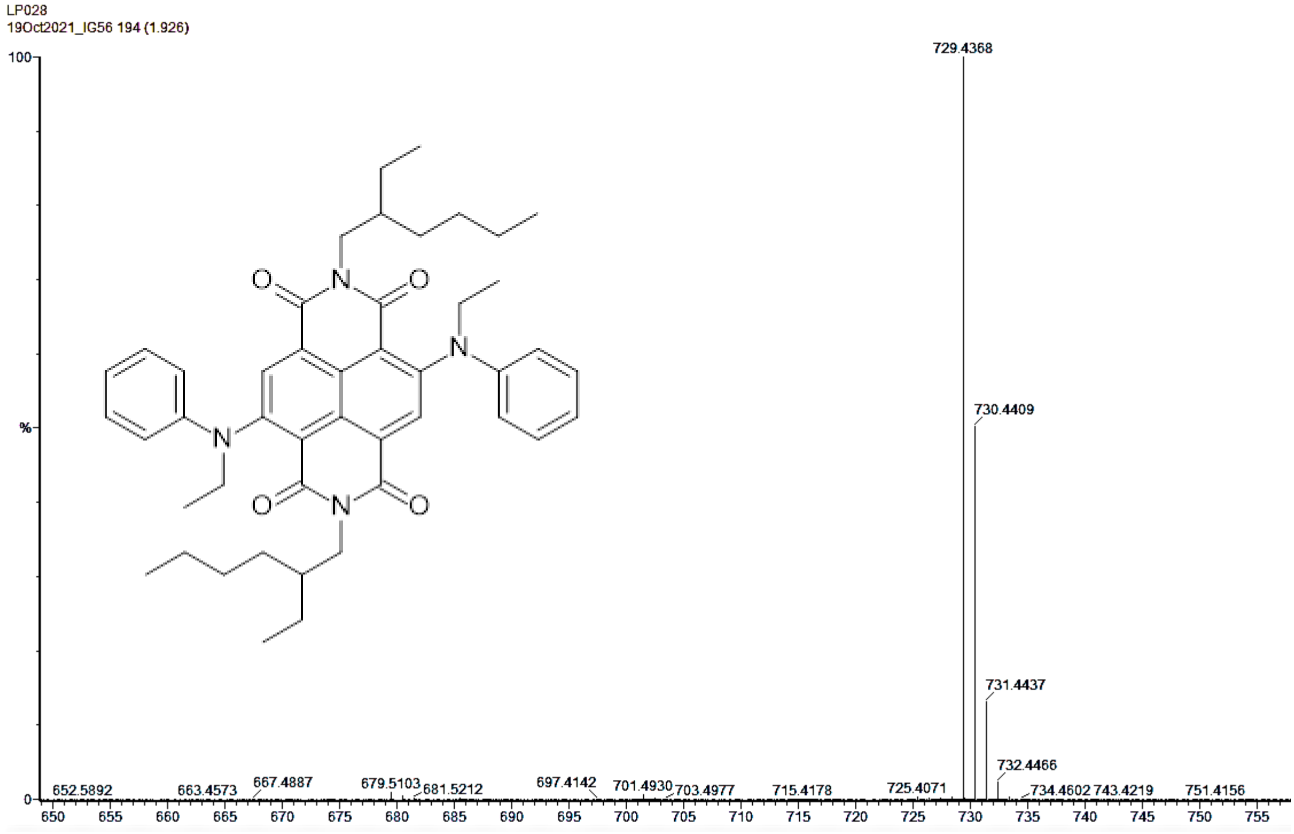

Mass	Calc. Mass	mDa	PPM	DBE	Formula	i-FIT	i-FIT Norm	Fit Conf %	C	H	N	O	S
1287.6521	1287.6490	3.1	2.4	31.5	C76 H95 N4 O10 S2	37.7	n/a	n/a	76	95	4	10	2

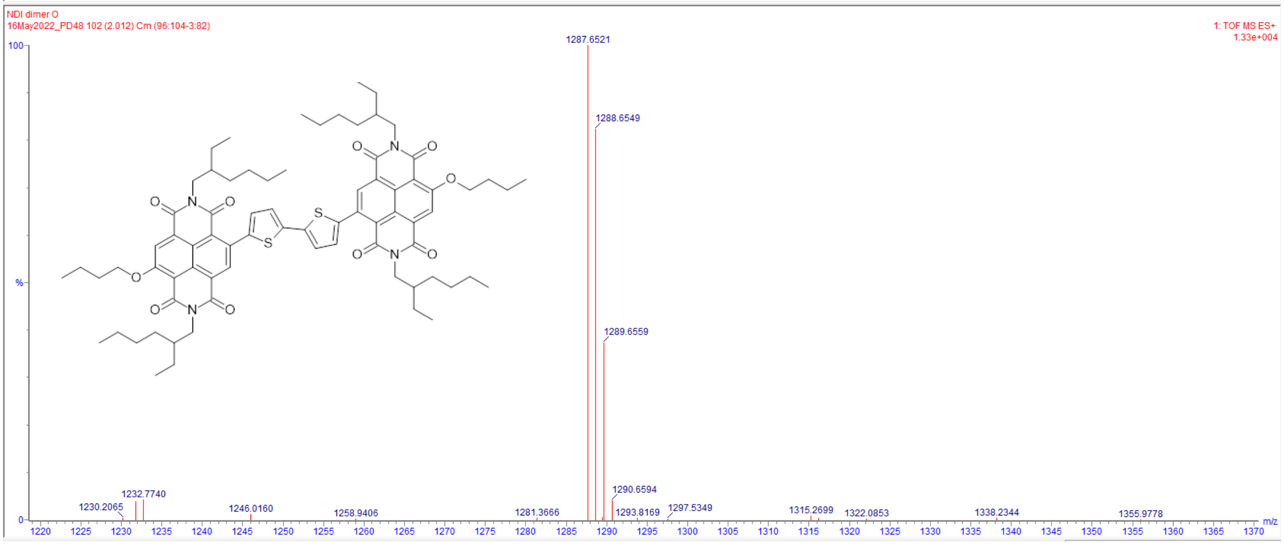

Mass	Calc. Mass	mDa	PPM	DBE	Formula	i-FIT	i-FIT Norm	Fit Conf %	C	H	N	O	S
1285.6862	1285.6809	5.3	4.1	31.5	C76 H97 N6 O8 S2	187.2	n/a	n/a	76	97	6	8	2

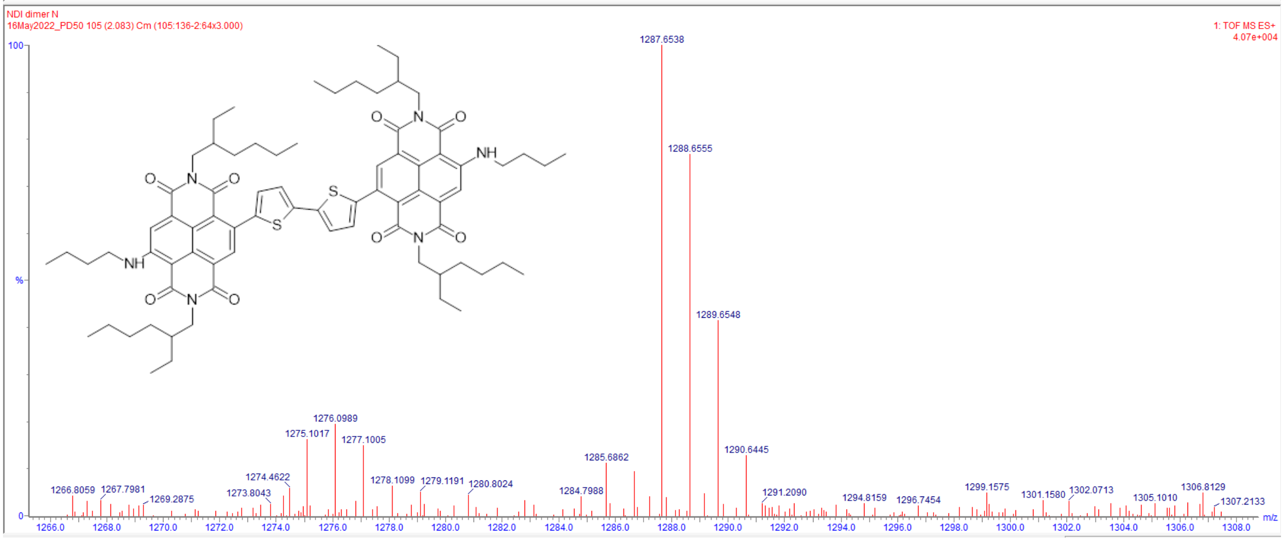

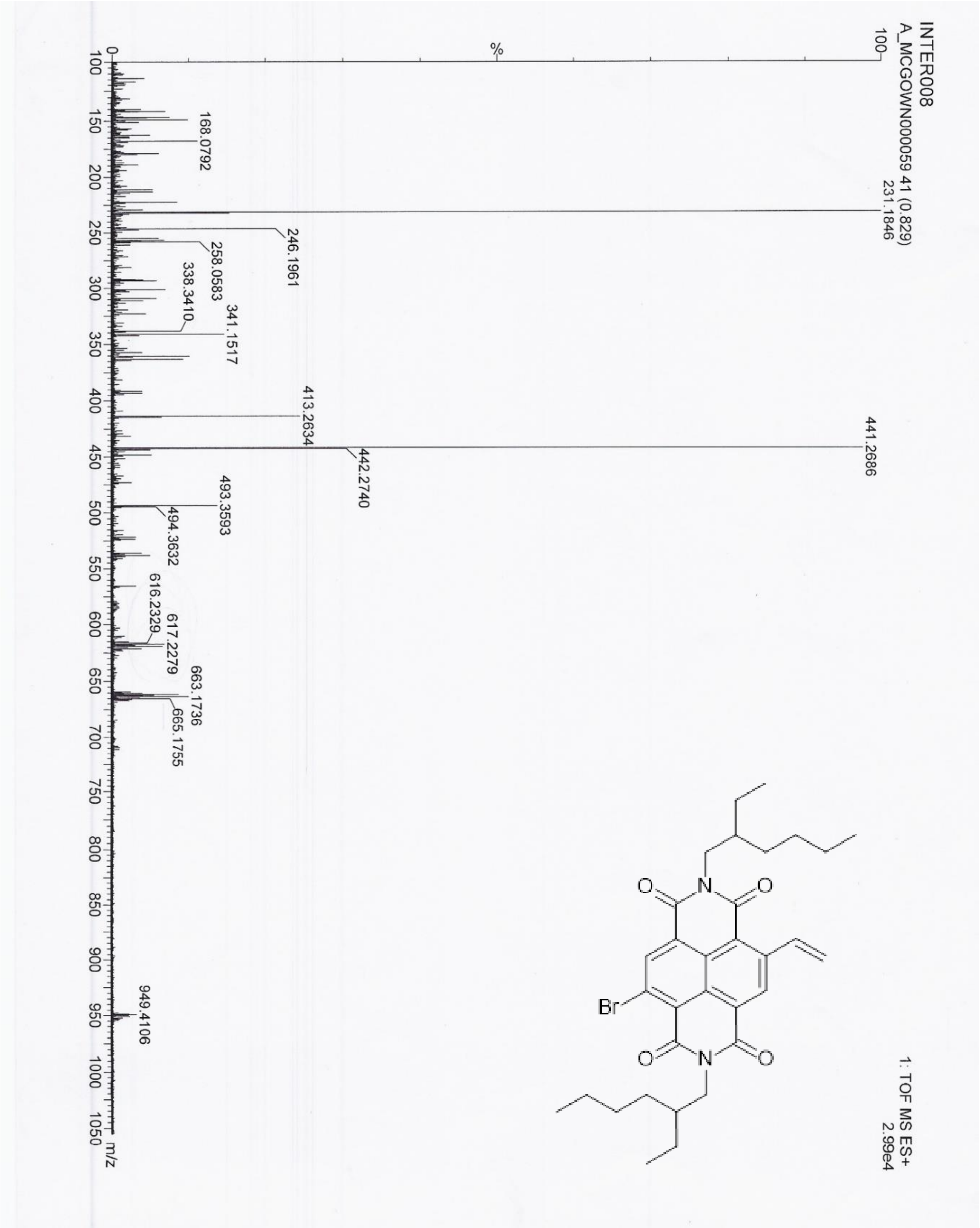
INTER008
A_MCGOWN000059 41 (0.829)
1: TOF MS ES+
2.99e4
100
%
0
231.1846
441.2686
442.2740
413.2634
246.1961
258.0583
168.0792
338.3410
341.1517
493.3593
494.3632
616.2329
617.2279
663.1736
665.1755
949.4106
m/z
Br
N
O

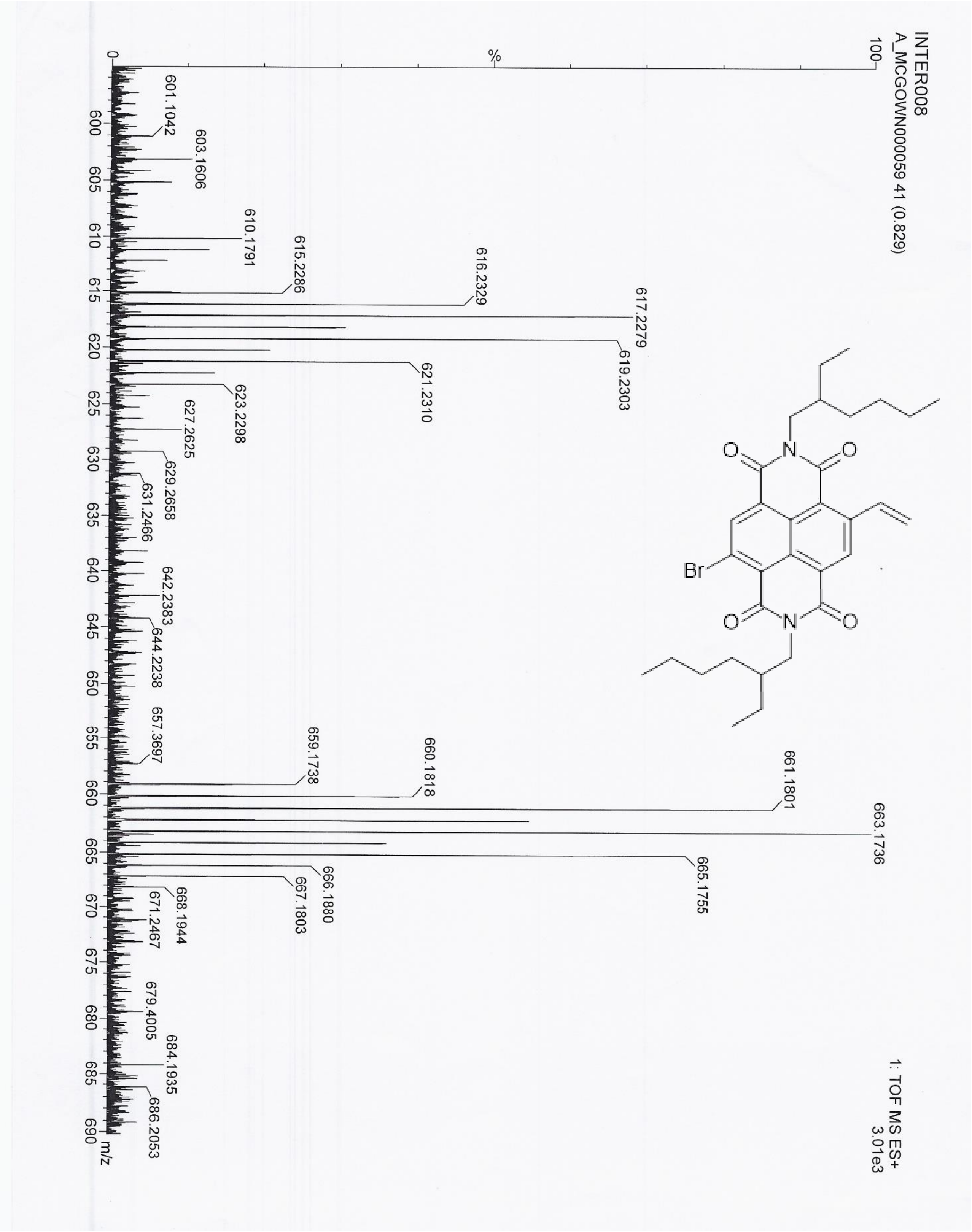

INTER008
A_MCGOWN000059 41 (0.829)
1: TOF MS ES+
3.01e3
100
%
0
Br
O
N
m/z
600
605
610
615
620
625
630
635
640
645
650
655
660
665
670
675
680
685
690
601.1042
603.1606
610.1791
615.2286
616.2329
617.2279
619.2303
621.2310
623.2298
627.2625
629.2658
631.2466
642.2383
644.2238
657.3697
659.1738
660.1818
661.1801
663.1736
665.1755
666.1880
667.1803
668.1944
671.2467
679.4005
684.1935
686.2053

S3.5 Crystal Data

2g

ncs | UK National Crystallography Service

www.ncs.ac.uk | info@ncs.ac.uk | 023 8059 6722

Submitted by: **John Spencer**

Solved by: **Graham J. Tizzard**

Sample ID: **DG140**

***R*₁=5.88**

Crystal Data and Experimental

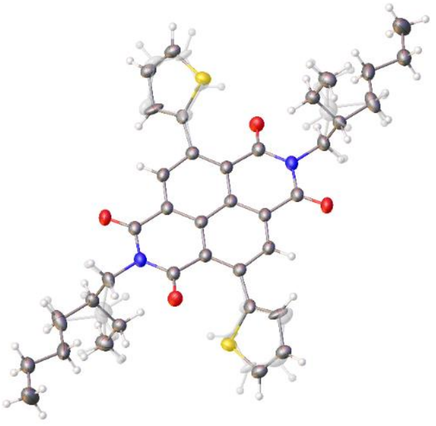

**Compound**	**2g**
Formula	$C_{38}H_{42}N_2O_4S_2$
Dcalc	1.342
μ/mm^{-1}	1.845
Formula Weight	654.85
Colour	red
Shape	rod-shaped
Size/mm^3	0.10×0.03×0.03
T/K	100(2)
Crystal System	monoclinic
Space Group	$P2_1/n$
a/Å	5.44570(10)
b/Å	17.5795(5)
c/Å	17.0505(4)
α/°	90
β/°	96.909(2)
γ/°	90
V/Å^3	1620.44(7)
Z	2
Z'	0.5
Wavelength/Å	1.54178
Radiation type	Cu K$_\alpha$
Θ_{min}/°	3.625
Θ_{max}/°	75.074
Measured Refl's.	14409
Indep't Refl's	3236
Refl's I≥2 σ(I)	2648
R_{int}	0.0534
Parameters	275
Restraints	78
Largest Peak	0.335
Deepest Hole	-0.482
GooF	1.060
wR_2 (all data)	0.1531
wR_2	0.1429
R_1 (all data)	0.0732
R_1	0.0588

Experimental. Single red rod-shaped crystals of **2g** were supplied. A suitable crystal 0.10×0.03×0.03 mm^3 was selected and mounted on a MITIGEN holder in perfluoroether oil on a Rigaku 007HF diffractometer equipped with Arc)Sec VHF Varimax confocal mirrors and a UG2 goniometer and HyPix 6000HE detector. The crystal was kept at a steady T = 100(2) K during data collection. The structure was solved with the ShelXT 2018/2 (Sheldrick, 2015) structure solution program using the using dual methods solution method and by using Olex2 1.5-alpha (Dolomanov et al., 2009) as the graphical interface. The model was refined with ShelXL 2018/3 (Sheldrick, 2015) using full matrix least squares minimisation on $\boldsymbol{F}^2$ minimisation.

Crystal Data. $C_{38}H_{42}N_2O_4S_2$, M_r = 654.85, monoclinic, $P2_1/n$ (No. 14), a = 5.44570(10) Å, b = 17.5795(5) Å, c = 17.0505(4) Å, β = 96.909(2)°, α = γ = 90°, V = 1620.44(7) Å^3, T = 100(2) K, Z = 2, Z' = 0.5, μ(Cu K$_\alpha$) = 1.845 mm^{-1}, 14409 reflections measured, 3236 unique (R_{int} = 0.0534) which were used in all calculations. The final wR_2 was 0.1531 (all data) and R_1 was 0.0588 (I≥2 σ(I)).

Structure Quality Indicators

**Reflections:**	d min (Cu\a) 2Θ=150.1° 0.80	I/σ(I) 27.2	Rint 5.34%	Full 135.4° 97% to 150.1° 99.7
**Refinement:**	Shift 0.000	Max Peak 0.3	Min Peak -0.5	GooF 1.060

A red rod-shaped crystal with dimensions 0.10×0.03×0.03 mm^3 was mounted on a MITIGEN holder in perfluoroether oil. X-ray diffraction data were collected using a Rigaku 007HF diffractometer equipped with Arc)Sec VHF Varimax confocal mirrors and a UG2 goniometer and HyPix 6000HE detector equipped with an Oxford Cryosystems low-temperature device, operating at T = 100(2) K.

Data were measured using profile data from ω-scans of 0.5 ° per frame for 2.0/8.0 s using Cu K$_\alpha$ radiation (Rotating anode, 40.0 kV, 30.0 mA). The total number of runs and images was based on the strategy calculation from the program CrysAlisPro 1.171.41.115a (Rigaku OD, 2021). The maximum resolution achieved was Θ = 75.074°.

Cell parameters were retrieved using the CrysAlisPro 1.171.41.115a (Rigaku OD, 2021) software and refined using CrysAlisPro 1.171.41.115a (Rigaku OD, 2021) on 5469 reflections, 38 % of the observed reflections. Data reduction was performed using the CrysAlisPro 1.171.41.115a (Rigaku OD, 2021) software which corrects for Lorentz polarisation. The final completeness is 99.70 % out to 75.074° in Θ.

A multi-scan absorption correction was performed using CrysAlisPro 1.171.41.115a (Rigaku Oxford Diffraction, 2021) Empirical absorption correction using spherical harmonics, implemented in SCALE3 ABSPACK scaling algorithm. The absorption coefficient μ of this material is 1.845 mm^{-1} at this wavelength (λ = 1.54178Å) and the minimum and maximum transmissions are 0.409 and 1.000.

The structure was solved in the space group $P2_1/n$ (# 14) by using dual methods using the ShelXT 2018/2 (Sheldrick, 2015) structure solution program and refined by full matrix least squares minimisation on $\boldsymbol{F^2}$ using ShelXL 2018/3 (Sheldrick, 2015). All non-hydrogen atoms were refined anisotropically. Hydrogen atom positions were calculated geometrically and refined using the riding model.

_*refine_special_details*: The thiophene is disordered over two sites (59:41). C9 and C10 are disordered over two positions (73:27). All equivalent pairs of atoms of the disorder components have 1,2 and 1,3 equal distance geometric restraints and thermal restraints applied.

The value of Z' is 0.5. This means that only half of the formula unit is present in the asymmetric unit, with the other half consisting of symmetry equivalent atoms.

Data Plots: Diffraction Data

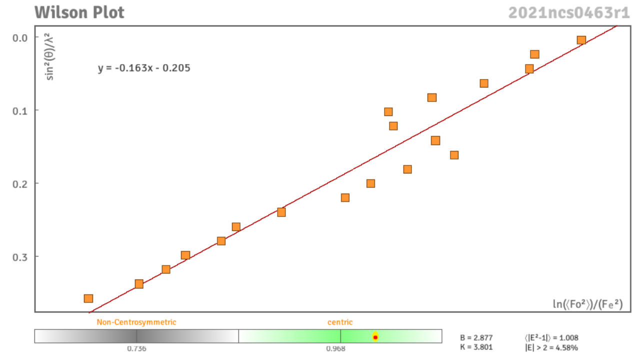

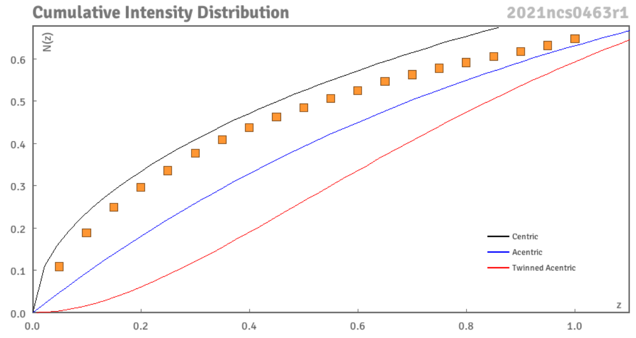

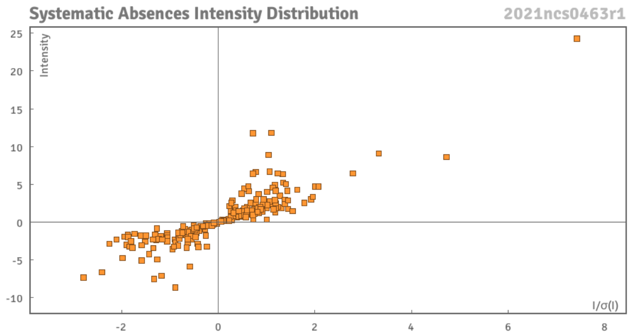

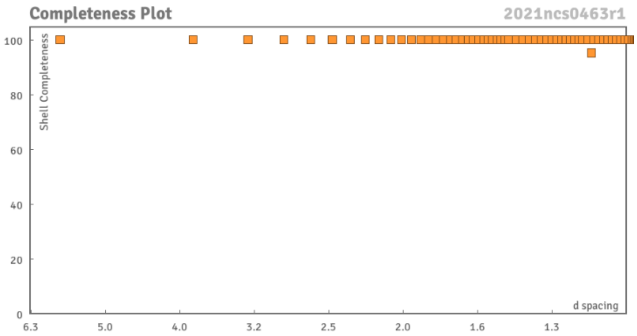

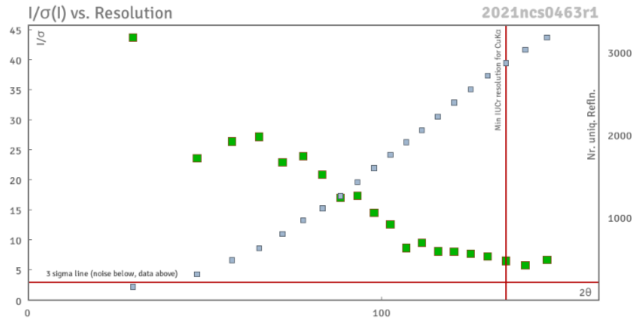

Data Plots: Refinement and Data

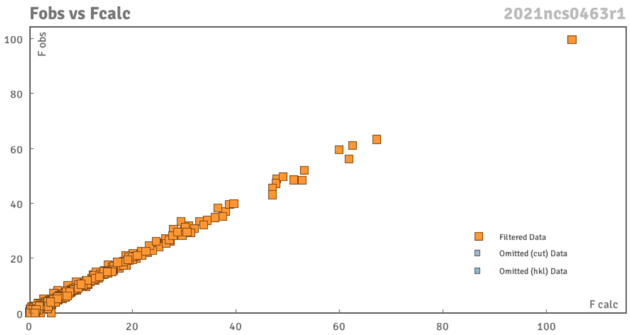

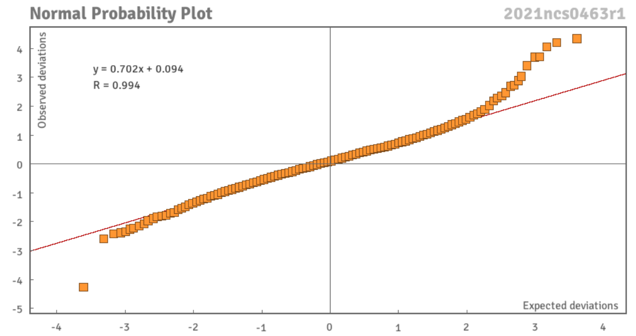

Reflection Statistics

Total reflections (after filtering)	14768	Unique reflections	3236
Completeness	0.97	Mean I/σ	16.22
hkl_{max} collected	(6, 19, 21)	hkl_{min} collected	(-6, -21, -21)
hkl_{max} used	(6, 21, 21)	hkl_{min} used	(-6, 0, 0)
Lim d_{max} collected	100.0	Lim d_{min} collected	0.77
d_{max} used	17.58	d_{min} used	0.8
Friedel pairs	1655	Friedel pairs merged	1
Inconsistent equivalents	7	R_{int}	0.0534
R_{sigma}	0.0367	Intensity transformed	0
Omitted reflections	0	Omitted by user (OMIT hkl)	0
Multiplicity	(3270, 2068, 1049, 536, 260, 93, 27, 3)	Maximum multiplicity	15
Removed systematic absences	359	Filtered off (Shel/OMIT)	0

Table 1: Fractional Atomic Coordinates (×10⁴) and Equivalent Isotropic Displacement Parameters (Å²×10³) for **2g**. U_{eq} is defined as 1/3 of the trace of the orthogonalised U_{ij}.

Atom	x	y	z	U_{eq}
S1A	5320(8)	3626(3)	2241.3(19)	34.9(7)
S1B	7884(16)	3425(6)	979(5)	40.4(11)
O1	4937(3)	5290.8(11)	2062.6(11)	40.0(5)
O2	-2094(4)	6678.8(10)	1528.7(11)	37.6(4)
N1	1342(4)	5943.8(11)	1825.5(12)	30.4(5)
C1	2992(5)	5377.5(14)	1645.4(14)	31.8(5)
C2	2303(5)	4919.5(13)	917.3(14)	28.5(5)
C3	3653(4)	4278.6(13)	739.3(14)	28.5(5)
C4	3117(5)	3959.0(14)	-24.5(14)	29.4(5)
C5	1223(5)	4217.0(13)	-565.2(14)	28.1(5)
C6	-287(4)	4825.0(13)	-373.5(13)	27.2(5)
C7	-765(5)	6173.4(14)	1338.6(14)	30.7(5)
C8	1908(5)	6347.0(15)	2588.1(14)	34.2(6)
C9A	261(8)	6059(2)	3194(2)	30.1(10)
C9B	1540(30)	5872(7)	3354(6)	44(3)
C10A	474(8)	5199(2)	3323(2)	32.9(10)
C10B	-990(20)	5482(7)	3238(6)	47(4)
C11	-1382(6)	4899.0(18)	3858.3(17)	44.4(7)
C12	1004(6)	6511.6(19)	3974.0(17)	50.9(8)
C13	3534(7)	6394.9(17)	4452.2(18)	52.1(8)
C14	3975(6)	6936.4(17)	5161.6(16)	44.4(7)
C15	6393(8)	6835(3)	5671(2)	70.7(12)
C16A	5650(30)	3869(10)	1264(10)	31(3)
C16B	5380(40)	3861(16)	1319(14)	26(4)
C17A	7932(16)	3076(5)	2325(6)	33.5(17)
C17B	8770(30)	3032(12)	1881(8)	42(3)
C18A	8990(20)	3086(8)	1644(6)	34.3(17)
C18B	7200(20)	3221(8)	2402(9)	33(3)
C19A	7600(40)	3536(14)	1020(12)	38(3)
C19B	5130(40)	3692(16)	2063(10)	30(3)

Table 2: Anisotropic Displacement Parameters (×10⁴) for **2g.** The anisotropic displacement factor exponent takes the form: $-2\pi^2[h^2a^{*2} \times U_{11}+ ... +2hka^* \times b^* \times U_{12}]$

Atom	U_{11}	U_{22}	U_{33}	U_{23}	U_{13}	U_{12}
S1A	39.7(14)	38.3(13)	26.3(11)	4.4(9)	2.1(10)	-0.5(10)
S1B	29.3(16)	36(3)	57.9(19)	6.0(14)	12.2(14)	7.3(13)
O1	41.6(10)	37.1(10)	39.0(10)	-6.8(8)	-4.8(8)	4.8(8)
O2	46.9(11)	31.1(10)	35.3(9)	-6.6(7)	7.1(8)	7.1(8)
N1	37.8(11)	25.2(10)	28.7(10)	-3.3(8)	6.2(8)	-1.4(9)
C1	38.2(13)	26.0(12)	31.5(12)	-0.1(10)	6.1(10)	0.2(11)
C2	34.5(12)	23.7(11)	28.0(11)	0.8(9)	6.8(9)	0.7(10)
C3	33.9(12)	23.0(11)	29.1(11)	1.8(9)	5.4(9)	-1.7(10)
C4	34.9(13)	23.3(12)	31.2(12)	0.3(9)	8.9(10)	2.2(10)
C5	36.3(13)	22.1(11)	27.0(11)	0.5(9)	8.3(9)	-0.5(10)
C6	32.2(12)	22.7(11)	27.6(11)	2.8(9)	6.9(9)	0.0(9)
C7	39.3(13)	24.1(12)	29.5(12)	-0.1(9)	8.1(10)	-1.2(10)
C8	44.7(15)	28.8(13)	29.1(12)	-7.1(10)	4.9(10)	-2.0(11)
C9A	28.0(19)	33(2)	28.1(18)	-3.8(14)	0.3(15)	0.1(17)
C9B	50(7)	52(8)	30(6)	-13(5)	1(5)	8(6)
C10A	37(2)	31(2)	29.5(17)	0.2(14)	-0.5(15)	-0.3(16)
C10B	60(8)	53(8)	28(5)	-1(5)	6(5)	1(6)
C11	51.2(17)	43.7(16)	37.7(15)	3.6(12)	2.7(12)	-8.3(14)

Atom	U_{11}	U_{22}	U_{33}	U_{23}	U_{13}	U_{12}
C12	70(2)	49.3(18)	37.3(15)	-14.4(13)	22.0(14)	-18.4(16)
C13	89(2)	31.8(15)	40.1(15)	1.4(12)	26.3(16)	12.8(15)
C14	59.1(18)	38.8(15)	36.7(14)	-3.1(12)	11.3(13)	5.0(14)
C15	76(2)	94(3)	40.7(17)	-5.6(18)	2.1(16)	37(2)
C16A	48(8)	17(5)	29(4)	-1(3)	12(4)	-4(4)
C16B	10(4)	32(8)	36(7)	3(5)	-1(5)	-1(3)
C17A	32(5)	28(5)	38(3)	5(3)	-3(3)	0(3)
C17B	34(6)	38(5)	52(9)	16(7)	-3(6)	12(4)
C18A	35(3)	29(3)	38(4)	6(3)	3(3)	0(2)
C18B	32(7)	21(5)	44(5)	10(4)	-9(4)	10(4)
C19A	31(6)	29(7)	52(6)	24(5)	0(4)	4(3)
C19B	24(4)	29(5)	33(7)	5(6)	-13(5)	16(3)

Table 3: Bond Lengths in Å for **2g.**

Atom	Atom	Length/Å
S1A	C16A	1.75(2)
S1A	C17A	1.712(8)
S1B	C16B	1.72(3)
S1B	C17B	1.702(12)
O1	C1	1.212(3)
O2	C7	1.215(3)
N1	C1	1.400(3)
N1	C7	1.392(3)
N1	C8	1.480(3)
C1	C2	1.489(3)
C2	C3	1.399(3)
C2	$C6^1$	1.422(3)
C3	C4	1.416(3)
C3	C16A	1.507(8)
C3	C16B	1.476(9)
C4	C5	1.375(3)
C5	C6	1.411(3)
C5	$C7^1$	1.481(3)
C6	$C6^1$	1.415(5)
C8	C9A	1.533(4)
C8	C9B	1.582(11)
C9A	C10A	1.529(6)
C9A	C12	1.561(4)
C9B	C10B	1.531(14)
C9B	C12	1.595(10)
C10A	C11	1.535(5)
C10B	C11	1.506(10)
C12	C13	1.528(5)
C13	C14	1.535(4)
C14	C15	1.498(5)
C16A	C19A	1.32(3)
C16B	C19B	1.33(3)
C17A	C18A	1.356(9)
C17B	C18B	1.349(12)
C18A	C19A	1.463(18)
C18B	C19B	1.459(17)

----
^{1}-x,1-y,-z

Table 4: Bond Angles in ° for **2g.**

Atom	Atom	Atom	Angle/°
C17A	S1A	C16A	92.1(7)
C17B	S1B	C16B	91.5(9)
C1	N1	C8	117.5(2)
C7	N1	C1	125.4(2)
C7	N1	C8	117.0(2)
O1	C1	N1	120.0(2)
O1	C1	C2	122.4(2)
N1	C1	C2	117.6(2)
C3	C2	C1	122.0(2)
C3	C2	$C6^1$	119.9(2)
$C6^1$	C2	C1	118.1(2)
C2	C3	C4	117.6(2)
C2	C3	C16A	128.1(8)
C2	C3	C16B	124.4(12)
C4	C3	C16A	114.3(8)
C4	C3	C16B	117.6(12)
C5	C4	C3	122.7(2)
C4	C5	C6	120.4(2)
C4	C5	$C7^1$	118.5(2)
C6	C5	$C7^1$	121.1(2)
C5	C6	$C2^1$	120.9(2)
C5	C6	$C6^1$	117.8(3)
$C6^1$	C6	$C2^1$	121.3(3)
O2	C7	N1	121.8(2)
O2	C7	$C5^1$	122.3(2)
N1	C7	$C5^1$	115.8(2)
N1	C8	C9A	110.9(2)
N1	C8	C9B	116.0(4)
C8	C9A	C12	107.0(3)
C10A	C9A	C8	112.5(3)
C10A	C9A	C12	111.9(3)
C8	C9B	C12	103.1(7)
C10B	C9B	C8	109.3(9)
C10B	C9B	C12	99.6(9)

Atom	Atom	Atom	Angle/°
C9A	C10A	C11	112.4(3)
C11	C10B	C9B	114.6(9)
C13	C12	C9A	120.7(3)
C13	C12	C9B	91.9(6)
C12	C13	C14	112.5(2)
C15	C14	C13	115.3(3)
C3	C16A	S1A	122.1(13)
C19A	C16A	S1A	111.8(9)
C19A	C16A	C3	125.1(16)
C3	C16B	S1B	117.5(17)
C19B	C16B	S1B	113.5(10)
C19B	C16B	C3	128(2)
C18A	C17A	S1A	110.8(9)
C18B	C17B	S1B	111.2(12)
C17A	C18A	C19A	113.2(12)
C17B	C18B	C19B	113.6(14)
C16A	C19A	C18A	112.0(16)
C16B	C19B	C18B	110.1(18)

----
^{1}-x,1-y,-z

Table 5: Torsion Angles in ° for **2g**.

Atom	Atom	Atom	Atom	Angle/°
S1A	C16A	C19A	C18A	-3(3)
S1A	C17A	C18A	C19A	-1.9(18)
S1B	C16B	C19B	C18B	4(3)
S1B	C17B	C18B	C19B	1(2)
O1	C1	C2	C3	10.9(4)
O1	C1	C2	C6^{1}	-165.7(2)
N1	C1	C2	C3	-171.5(2)
N1	C1	C2	C6^{1}	12.0(3)
N1	C8	C9A	C10A	56.3(4)
N1	C8	C9A	C12	179.7(3)
N1	C8	C9B	C10B	-48.5(11)
N1	C8	C9B	C12	-153.8(4)
C1	N1	C7	O2	-178.8(2)
C1	N1	C7	C5^{1}	-1.0(3)
C1	N1	C8	C9A	-103.3(3)
C1	N1	C8	C9B	-71.8(6)
C1	C2	C3	C4	-169.5(2)
C1	C2	C3	C16A	10.6(11)
C1	C2	C3	C16B	18.1(15)
C2	C3	C4	C5	-5.0(4)
C2	C3	C16A	S1A	46.9(18)
C2	C3	C16A	C19A	-146(2)
C2	C3	C16B	S1B	-149.1(13)
C2	C3	C16B	C19B	40(4)
C3	C4	C5	C6	0.1(4)
C3	C4	C5	C7^{1}	-178.0(2)
C3	C16A	C19A	C18A	-170.9(16)
C3	C16B	C19B	C18B	176(2)
C4	C3	C16A	S1A	-133.0(11)
C4	C3	C16A	C19A	34(3)
C4	C3	C16B	S1B	38(2)
C4	C3	C16B	C19B	-133(3)
C4	C5	C6	C2^{1}	-177.9(2)
C4	C5	C6	C6^{1}	2.7(4)
C6^{1}	C2	C3	C4	7.0(3)
C6^{1}	C2	C3	C16A	-172.9(11)
C6^{1}	C2	C3	C16B	-165.5(15)
C7	N1	C1	O1	170.2(2)
C7	N1	C1	C2	-7.5(4)
C7	N1	C8	C9A	78.1(3)
C7	N1	C8	C9B	109.7(6)
C7^{1}	C5	C6	C2^{1}	0.2(3)
C7^{1}	C5	C6	C6^{1}	-179.3(3)
C8	N1	C1	O1	-8.2(3)
C8	N1	C1	C2	174.1(2)

Atom	Atom	Atom	Atom	Angle/°
C8	N1	C7	O2	-0.4(3)
C8	N1	C7	C5[1]	177.4(2)
C8	C9A	C10A	C11	-173.6(3)
C8	C9A	C12	C13	-68.0(4)
C8	C9B	C10B	C11	169.3(7)
C8	C9B	C12	C13	-108.5(7)
C9A	C12	C13	C14	175.6(3)
C9B	C12	C13	C14	178.6(4)
C10A	C9A	C12	C13	55.8(4)
C10B	C9B	C12	C13	138.9(8)
C12	C9A	C10A	C11	65.8(4)
C12	C9B	C10B	C11	-83.1(11)
C12	C13	C14	C15	178.2(3)
C16A	S1A	C17A	C18A	0.4(11)
C16A	C3	C4	C5	174.9(10)
C16B	S1B	C17B	C18B	0.9(19)
C16B	C3	C4	C5	168.0(14)
C17A	S1A	C16A	C3	170.0(14)
C17A	S1A	C16A	C19A	1(2)
C17A	C18A	C19A	C16A	3(3)
C17B	S1B	C16B	C3	-175(2)
C17B	S1B	C16B	C19B	-3(3)
C17B	C18B	C19B	C16B	-4(3)

----
[1]-x,1-y,-z

Table 6: Hydrogen Fractional Atomic Coordinates (×10^4) and Equivalent Isotropic Displacement Parameters (Å^2×10^3) for **2g**. U_{eq} is defined as 1/3 of the trace of the orthogonalised U_{ij}.

Atom	x	y	z	U_{eq}
H4	4106.55	3549.16	-169.32	35
H8AA	1641.79	6899.7	2505.27	41
H8AB	3666.65	6266.28	2793.95	41
H8BC	846.5	6805.04	2577.33	41
H8BD	3646.53	6522.25	2633.42	41
H9A	-1497.9	6181.97	2996.49	36
H9B	2952.68	5526.34	3539.44	53
H10A	2172.78	5075.78	3564.13	39
H10B	189.89	4938.73	2805.52	39
H10C	-2287.59	5876.06	3234.33	56
H10D	-1195.88	5230.65	2714.87	56
H11A	-3068.42	5017.26	3621.21	67
H11B	-1069.66	5141.23	4378.42	67
H11C	-1194.58	4346.8	3917.03	67
H11D	-2802.52	4578.27	3668.71	67
H11E	-1701.05	5157.8	4345.03	67
H11F	99.93	4581.71	3964.69	67
H12A	859.2	7059.17	3840.54	61
H12B	-259.73	6401.47	4330.11	61
H12C	786.52	7025.21	3737.53	61
H12D	-394.41	6384.98	4271.8	61
H13A	4839.31	6476.14	4104.02	62
H13B	3663.48	5863.12	4643.94	62
H14A	3879.74	7466.02	4962.85	53
H14B	2621.13	6866.45	5493.72	53
H15A	7755.13	6934.33	5358.32	106
H15B	6516.14	6312.78	5872.82	106
H15C	6488.29	7191.9	6115.3	106
H17A	8557.08	2800.77	2785.94	40

Atom	x	y	z	U_{eq}
H17B	10190.81	2720.03	2001.67	51
H18A	10482.77	2826.39	1579.68	41
H18B	7410.05	3062.55	2938.54	40
H19A	8041.85	3583.05	499.66	45
H19B	3801.32	3854.42	2336.03	36

Table 7: Atomic Occupancies for all atoms that are not fully occupied in **2g**.

Atom	Occupancy
S1A	0.590(4)
S1B	0.410(4)
H8AA	0.736(9)
H8AB	0.736(9)
H8BC	0.264(9)
H8BD	0.264(9)
C9A	0.736(9)
H9A	0.736(9)
C9B	0.264(9)
H9B	0.264(9)
C10A	0.736(9)
H10A	0.736(9)
H10B	0.736(9)
C10B	0.264(9)
H10C	0.264(9)
H10D	0.264(9)
H11A	0.736(9)
H11B	0.736(9)
H11C	0.736(9)
H11D	0.264(9)
H11E	0.264(9)
H11F	0.264(9)
H12A	0.736(9)
H12B	0.736(9)
H12C	0.264(9)
H12D	0.264(9)
C16A	0.590(4)
C16B	0.410(4)
C17A	0.590(4)
H17A	0.590(4)
C17B	0.410(4)
H17B	0.410(4)
C18A	0.590(4)
H18A	0.590(4)
C18B	0.410(4)
H18B	0.410(4)
C19A	0.590(4)
H19A	0.590(4)
C19B	0.410(4)
H19B	0.410(4)

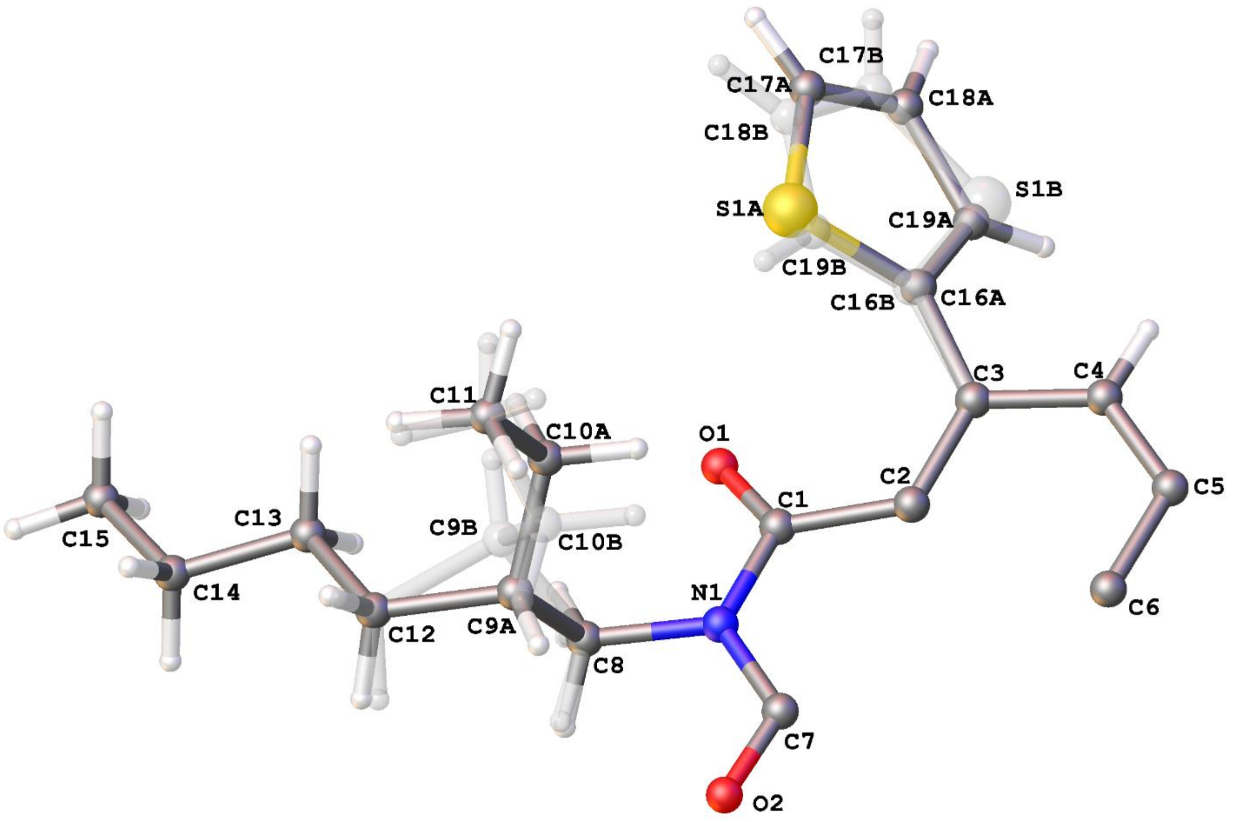

Fig S28 Asymmetric unit of **2g** showing a half molecule (the other half is generated by crystallographic inversion symmetry). Minor disorder components are shown 'ghosted' for clarity

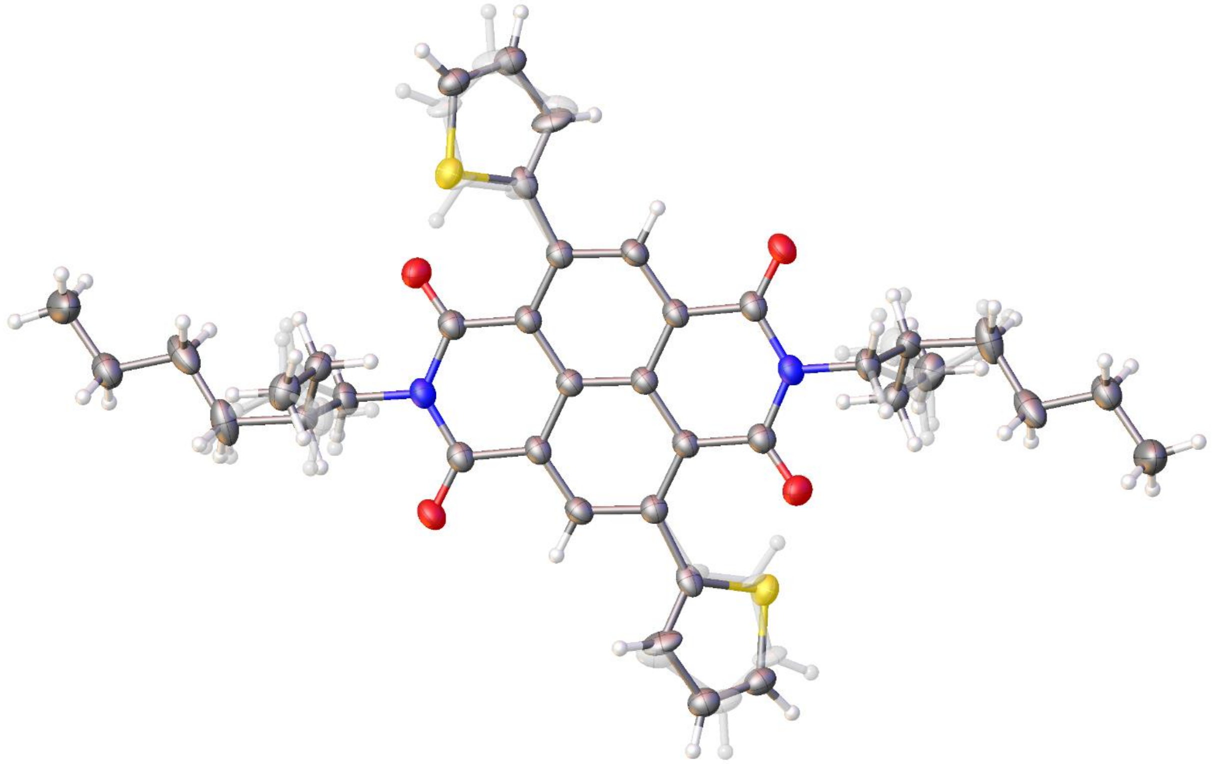

Fig S29 2g including crystallographic symmetry generated half. Minor disorder components are shown 'ghosted' for clarity. ADP ellipsoids are displayed at 50% probability.

4a

www.ncs.ac.uk | info@ncs.ac.uk | 023 8059 6722

Submitted by: **John Spencer**
Solved by: **Graham J. Tizzard**
Sample ID: **DG-NDI**

R_1=7.75

Crystal Data and Experimental

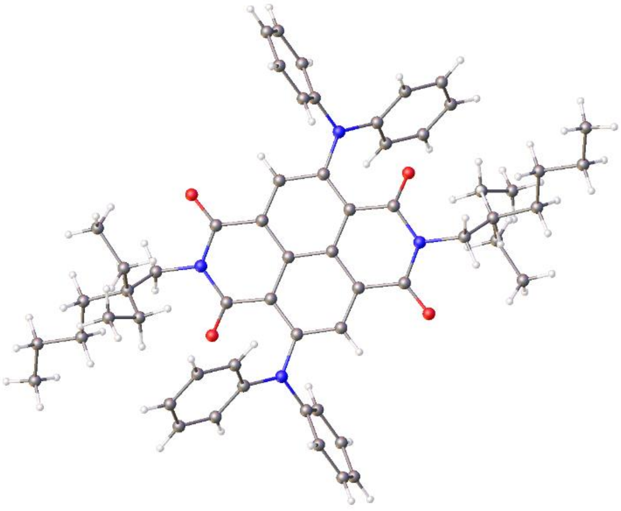

Experimental. Single blue lath-shaped crystals of **4a** were supplied. A suitable crystal with dimensions 0.20 × 0.04 × 0.01 mm^3 was selected and mounted on a MITIGEN holder in perfluoroether oil on a Rigaku 007HF diffractometer with HF Varimax confocal mirrors, an AFC11 goniometer and HyPix 6000HE detector diffractometer. The crystal was kept at a steady T = 100.00(11) K during data collection. The structure was solved with the **ShelXT** 2018/2 (Sheldrick, 2015) solution program using dual methods and by using **Olex2** 1.5 (Dolomanov et al., 2009) as the graphical interface. The model was refined with **ShelXL** 2018/3 (Sheldrick, 2015) using full matrix least squares minimisation on $\boldsymbol{F^2}$.

Crystal Data. $C_{54}H_{56}N_4O_4$, M_r = 825.02, triclinic, P-1 (No. 2), a = 11.5685(9) Å, b = 14.3736(9) Å, c = 15.5702(10) Å, α = 115.595(6)°, β = 99.722(7)°, γ = 97.130(6)°, V = 2244.2(3) Å^3, T = 100.00(11) K, Z = 2, Z' = 1, μ(Cu K$_\alpha$) = 0.606, 28811 reflections measured, 4997 unique (R_{int} = 0.0926) which were used in all calculations. The final wR_2 was 0.2433 (all data) and R_1 was 0.0775 (I≥2 σ(I)).

**Compound**	**4a**
Formula	$C_{54}H_{56}N_4O_4$
$D_{calc.}$/ g cm^{-3}	1.221
μ/mm^{-1}	0.606
Formula Weight	825.02
Colour	blue
Shape	lath-shaped
Size/mm^3	0.20×0.04×0.01
T/K	100.00(11)
Crystal System	triclinic
Space Group	P-1
a/Å	11.5685(9)
b/Å	14.3736(9)
c/Å	15.5702(10)
α/°	115.595(6)
β/°	99.722(7)
γ/°	97.130(6)
V/Å^3	2244.2(3)
Z	2
Z'	1
Wavelength/Å	1.54178
Radiation type	Cu K$_\alpha$
Θ_{min}/°	3.249
Θ_{max}/°	51.866
Measured Refl's.	28811
Indep't Refl's	4997
Refl's I≥2 σ(I)	3279
R_{int}	0.0926
Parameters	613
Restraints	490
Largest Peak	0.219
Deepest Hole	-0.208
GooF	1.018
wR_2 (all data)	0.2433
wR_2	0.2049
R_1 (all data)	0.1117
R_1	0.0775

Structure Quality Indicators

**Reflections:**	d min (Cu\a) 2Θ=103.7° 0.98	I/σ(I) 21.0	Rint 9.26%	Full 103.7° 100
**Refinement:**	Shift 0.000	Max Peak 0.2	Min Peak -0.2	GooF 1.018

A blue lath-shaped-shaped crystal with dimensions 0.20 × 0.04 × 0.01 mm^3 was mounted on a MITIGEN holder in perfluoroether oil. Data were collected using a Rigaku 007HF diffractometer with HF Varimax confocal mirrors, an AFC11 goniometer and HyPix 6000HE detector diffractometer operating at T = 100.00(11) K.

Data were measured using profile data from ω-scans with Cu K_α radiation. The diffraction pattern was indexed and the total number of runs and images was based on the strategy calculation from the program CrysAlisPro 1.171.41.120a (Rigaku OD, 2021). The maximum resolution that was achieved was Θ = 51.866° (0.98 Å).

The unit cell was refined using CrysAlisPro 1.171.41.120a (Rigaku OD, 2021) on 4803 reflections, 17% of the observed reflections.

Data reduction, scaling and absorption corrections were performed using CrysAlisPro 1.171.41.120a (Rigaku OD, 2021). The final completeness is 100.00 % out to 51.866° in Θ. A multi-scan absorption correction was performed using CrysAlisPro 1.171.41.120a (Rigaku Oxford Diffraction, 2021) with empirical absorption correction using spherical harmonics as implemented in the SCALE3 ABSPACK scaling algorithm. The absorption coefficient μ of this material is 0.606 mm^{-1} at this wavelength (λ = 1.54178Å) and the minimum and maximum transmissions are 0.647 and 1.000.

The structure was solved and the space group P-1 (# 2) determined by the ShelXT 2018/2 (Sheldrick, 2015) structure solution program using dual methods and refined by full matrix least squares minimisation on $\boldsymbol{F^2}$ using ShelXL 2018/3 (Sheldrick, 2015). All non-hydrogen atoms were refined anisotropically. Hydrogen atom positions were calculated geometrically and refined using the riding model.

_diffrn_special_details: The crystal was a very weak diffractor so data collection resolution was limited to 2theta = 103.732degs.

_refine_special_details: both ethyl branches modelled as disordered (ca. 75:25). Terminal C-atom of one hexyl ligand modelled as disordered (ca. 57:43). 1,2 and 1,3 equal distance restraints applied to all equivalent pairs of atoms. Thermal restraints have been applied throughout.

There are two 'halves' of two independent molecules (with the second 'halves' generated by crystallographic inversion centres in each instance) in the asymmetric unit, which is represented by the reported sum formula. In other words: Z is 2 and Z' is 1.

Data Plots: Diffraction Data

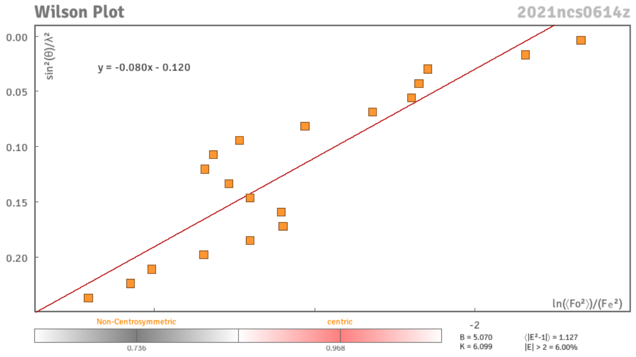

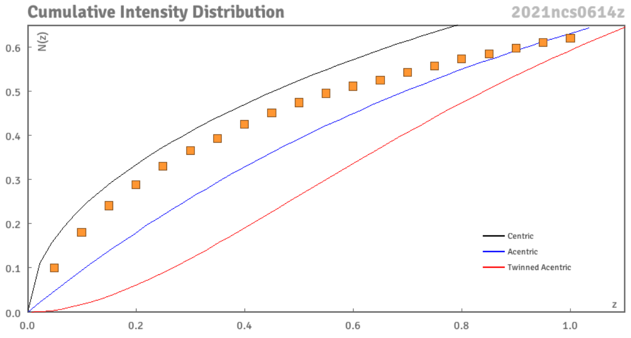

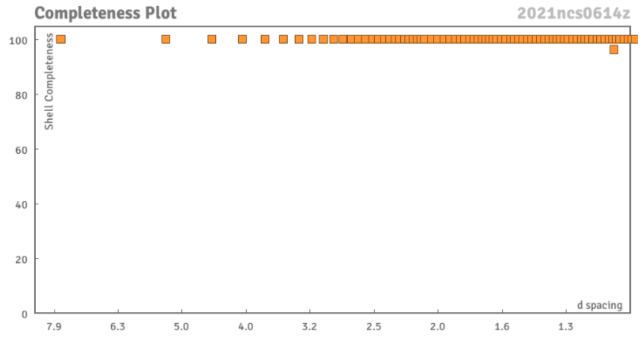

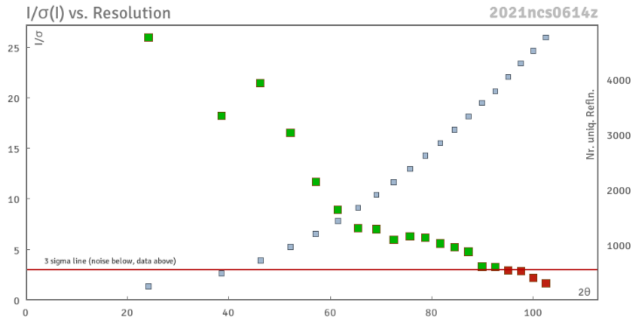

Data Plots: Refinement and Data

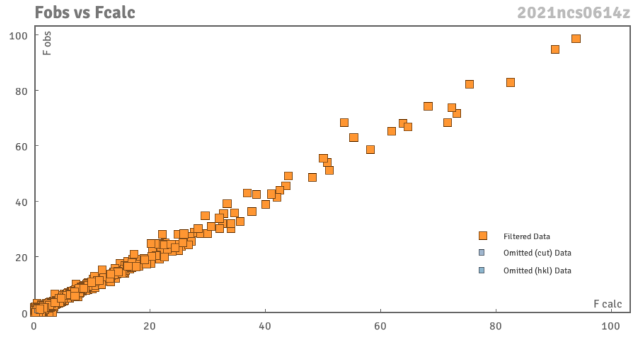

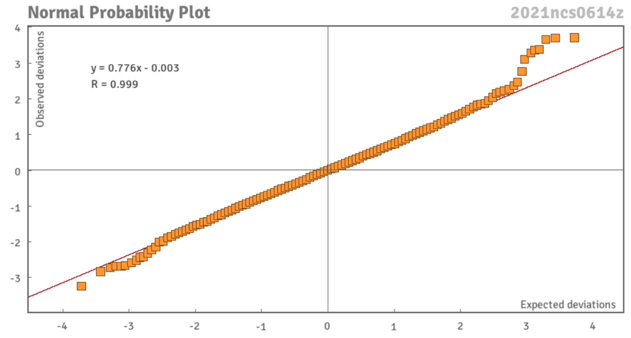

Reflection Statistics

Total reflections (after filtering)	28811	Unique reflections	4997
Completeness	1.0	Mean I/σ	8.35
hkl_{max} collected	(10, 14, 15)	hkl_{min} collected	(-11, -14, -15)
hkl_{max} used	(11, 13, 15)	hkl_{min} used	(-11, -14, 0)
Lim d_{max} collected	100.0	Lim d_{min} collected	0.77
d_{max} used	13.6	d_{min} used	0.98
Friedel pairs	3371	Friedel pairs merged	1
Inconsistent equivalents	4	R_{int}	0.0926
R_{sigma}	0.0477	Intensity transformed	0
Omitted reflections	0	Omitted by user (OMIT hkl)	0
Multiplicity	(1576, 1906, 1558, 987, 804, 672, 458, 227, 105, 46, 26, 3)	Maximum multiplicity	14
Removed systematic absences	0	Filtered off (Shel/OMIT)	0

Table 8: Fractional Atomic Coordinates (×10⁴) and Equivalent Isotropic Displacement Parameters (Å²×10³) for **4a**. U_{eq} is defined as 1/3 of the trace of the orthogonalised U_{ij}.

Atom	x	y	z	U_{eq}
O1	6396(3)	3292(3)	2614(2)	81.7(9)
O2	7551(3)	3393(3)	5583(2)	86.5(10)
N1	6915(3)	3290(3)	4074(3)	70.9(10)
N2	5735(4)	6121(4)	7877(3)	92.1(13)
C1	6206(4)	3558(4)	3418(3)	69.8(12)
C2	6914(4)	3659(4)	5058(3)	70.1(12)
C3	6135(4)	4397(4)	5434(3)	65.7(11)
C4	6216(4)	4894(4)	6426(3)	73.2(13)
C5	5532(4)	5629(4)	6852(3)	72.7(13)
C6	4691(4)	5832(3)	6228(3)	66.3(12)
C7	4646(4)	5364(3)	5208(3)	61.7(11)
C8	7779(4)	2645(4)	3694(4)	81.6(14)
C9	7237(6)	1480(5)	3069(6)	120(2)
C12	8205(8)	919(6)	2663(8)	168(3)
C13	8703(8)	1171(9)	1997(8)	197(5)
C14	9551(11)	531(12)	1500(9)	274(8)
C16	5955(5)	5495(6)	8362(4)	99.9(17)
C17	5049(6)	4695(6)	8228(4)	101.4(17)
C18	5245(6)	4106(6)	8720(4)	122(2)
C19	6344(7)	4302(9)	9335(5)	151(3)
C20	7249(7)	5084(10)	9453(6)	171(4)
C21	7061(5)	5688(8)	8986(4)	143(3)
C22	5871(5)	7223(6)	8457(4)	109.7(19)
C23	5490(6)	7575(7)	9339(5)	157(3)
C24	5680(10)	8657(10)	9907(7)	215(5)
C25	6174(10)	9386(9)	9621(9)	233(6)
C26	6528(7)	9019(7)	8761(7)	175(4)
C27	6388(5)	7937(6)	8180(5)	119(2)
C10A	6603(11)	952(7)	3484(9)	136(4)
C11A	5840(20)	-173(11)	2790(20)	170(8)
C15B	8950(20)	-680(20)	867(18)	236(13)
C10B	6044(17)	1001(17)	2650(20)	108(9)
C11B	5470(60)	-60(30)	2590(50)	160(30)
C15A	10185(18)	1010(20)	926(16)	350(17)
O51	1850(3)	2912(3)	3259(2)	83.2(10)
O52	2600(3)	4155(2)	6552(2)	78.5(9)
N51	2161(3)	3462(3)	4899(3)	68.4(10)
N52	781(3)	4868(4)	7502(3)	83.6(12)
C51	1601(4)	3452(4)	4033(4)	69.0(12)
C52	1923(4)	4058(4)	5812(3)	67.3(12)
C53	911(4)	4577(3)	5836(3)	63.6(11)
C54	415(4)	4682(3)	4987(3)	63.0(11)
C55	753(4)	4143(3)	4096(3)	63.1(11)
C56	480(4)	5046(4)	6677(3)	69.9(12)
C57	325(4)	4289(4)	3300(3)	68.7(12)
C58	3138(4)	2897(4)	4897(4)	79.6(13)
C59	2803(5)	1881(4)	4955(5)	97.8(17)
C62	3949(5)	1465(5)	5112(5)	114(2)
C63	4857(5)	2086(5)	6088(4)	100.9(17)
C64	5990(6)	1650(5)	6156(5)	111.9(19)
C65	6874(6)	2235(6)	7136(5)	133(2)
C66	960(5)	5720(6)	8462(4)	89.3(15)
C67	1811(5)	6629(6)	8773(4)	100.7(17)
C68	1987(6)	7458(6)	9706(4)	124(2)
C69	1316(7)	7369(8)	10331(5)	130(2)
C70	477(6)	6470(8)	10030(5)	122(2)
C71	271(5)	5620(6)	9092(4)	105.9(18)
C72	793(5)	3823(6)	7375(5)	95.2(16)

Atom	x	y	z	U_{eq}
C73	1679(5)	3685(6)	8019(5)	112(2)
C74	1675(7)	2671(8)	7900(7)	145(3)
C75	856(8)	1819(8)	7144(9)	174(4)
C76	-3(7)	1960(7)	6523(8)	169(4)
C77	-39(5)	2972(6)	6644(6)	121(2)
C60A	1788(7)	1048(6)	4190(6)	107(3)
C61A	2044(9)	446(7)	3203(7)	128(3)
C60B	1950(20)	1639(16)	5380(20)	119(8)
C61B	1530(20)	487(15)	5020(20)	134(10)

Table 9: Anisotropic Displacement Parameters (×10[4]) for **4a.** The anisotropic displacement factor exponent takes the form: $-2\pi^2[h^2a^{*2} \times U_{11} + ... + 2hka^* \times b^* \times U_{12}]$

Atom	U_{11}	U_{22}	U_{33}	U_{23}	U_{13}	U_{12}
O1	83(2)	97(2)	71(2)	34.4(18)	33.4(17)	37.0(18)
O2	82(2)	105(2)	89(2)	53(2)	20.6(17)	48.6(19)
N1	69(2)	80(2)	69(2)	31(2)	25.1(18)	34(2)
N2	82(3)	128(3)	54(2)	28(2)	10.1(19)	41(3)
C1	63(3)	82(3)	61(3)	26(2)	21(2)	26(2)
C2	62(3)	78(3)	72(3)	34(2)	16(2)	28(2)
C3	58(3)	80(3)	62(3)	32(2)	17(2)	24(2)
C4	65(3)	95(3)	61(3)	35(2)	12(2)	29(2)
C5	64(3)	94(3)	50(2)	24(2)	11(2)	25(2)
C6	61(3)	78(3)	56(2)	25(2)	15(2)	24(2)
C7	54(2)	74(3)	55(2)	26(2)	15.3(19)	21(2)
C8	82(3)	82(3)	94(3)	40(3)	38(3)	44(3)
C9	121(5)	81(4)	160(6)	42(4)	57(4)	47(3)
C12	151(7)	118(5)	200(9)	28(5)	55(6)	75(5)
C13	132(6)	221(10)	172(8)	11(7)	73(6)	79(6)
C14	170(10)	331(16)	185(10)	-23(11)	50(8)	130(11)
C16	75(3)	176(6)	58(3)	54(4)	21(3)	58(4)
C17	90(4)	162(5)	74(3)	63(4)	22(3)	63(4)
C18	113(4)	211(7)	100(4)	101(5)	45(3)	94(5)
C19	114(5)	314(11)	115(5)	152(7)	54(4)	114(6)
C20	93(5)	378(13)	122(5)	166(8)	49(4)	102(6)
C21	78(4)	295(9)	77(4)	100(5)	18(3)	61(5)
C22	82(4)	129(5)	64(3)	-2(3)	18(3)	21(4)
C23	127(5)	185(7)	78(4)	-12(4)	41(4)	17(5)
C24	201(10)	191(8)	113(6)	-54(6)	85(6)	-19(8)
C25	198(10)	158(8)	196(9)	-55(7)	116(8)	-25(7)
C26	135(6)	128(5)	167(7)	-23(5)	78(5)	0(5)
C27	86(4)	116(5)	97(4)	-4(4)	33(3)	12(4)
C10A	199(10)	84(6)	138(10)	51(5)	67(8)	42(6)
C11A	246(17)	87(8)	182(15)	55(9)	96(12)	22(8)
C15B	250(30)	289(18)	155(19)	62(17)	32(17)	187(19)
C10B	131(10)	80(13)	99(19)	26(12)	40(11)	14(10)
C11B	260(50)	90(20)	150(40)	30(20)	160(40)	30(20)
C15A	141(15)	540(40)	171(18)	-30(19)	79(13)	70(20)
O51	84(2)	109(2)	80(2)	52.6(19)	30.5(17)	53.4(19)
O52	69.0(19)	106(2)	79(2)	56.1(18)	12.3(16)	39.3(17)
N51	62(2)	88(3)	75(2)	50(2)	16.7(18)	36.9(19)
N52	74(2)	133(3)	84(3)	76(3)	27(2)	51(2)
C51	65(3)	85(3)	74(3)	47(3)	21(2)	30(2)
C52	58(3)	87(3)	73(3)	50(2)	10(2)	29(2)
C53	56(2)	80(3)	66(2)	43(2)	10(2)	29(2)
C54	53(2)	77(3)	67(2)	40(2)	9(2)	27(2)
C55	55(2)	80(3)	68(3)	43(2)	14(2)	29(2)
C56	58(3)	96(3)	71(3)	52(3)	11(2)	29(2)

Atom	U_{11}	U_{22}	U_{33}	U_{23}	U_{13}	U_{12}
C57	60(3)	94(3)	65(3)	46(2)	12(2)	32(2)
C58	70(3)	108(4)	91(3)	63(3)	27(2)	51(3)
C59	93(4)	84(3)	119(4)	49(3)	13(3)	41(3)
C62	109(4)	95(4)	136(5)	50(4)	16(3)	55(3)
C63	101(4)	108(4)	115(4)	64(3)	22(3)	54(3)
C64	110(4)	119(5)	130(5)	70(4)	28(3)	61(4)
C65	109(5)	165(6)	142(5)	85(5)	16(4)	60(4)
C66	67(3)	160(5)	74(3)	76(3)	18(3)	53(3)
C67	81(4)	160(5)	79(3)	69(4)	20(3)	35(3)
C68	115(5)	180(6)	76(4)	64(4)	14(3)	27(4)
C69	124(5)	201(7)	78(4)	71(5)	26(4)	53(5)
C70	108(5)	217(7)	80(4)	93(5)	36(4)	63(5)
C71	84(3)	188(6)	92(4)	97(4)	30(3)	56(4)
C72	71(3)	151(5)	128(4)	110(4)	36(3)	50(3)
C73	91(4)	182(6)	139(5)	127(5)	41(3)	67(4)
C74	101(5)	216(7)	225(8)	188(7)	45(5)	69(5)
C75	114(6)	194(8)	307(11)	204(8)	28(6)	45(5)
C76	106(5)	164(6)	295(10)	175(7)	7(5)	26(5)
C77	74(4)	147(5)	195(6)	134(5)	13(4)	24(3)
C60A	99(5)	99(5)	116(6)	49(5)	3(4)	35(4)
C61A	137(7)	94(6)	130(6)	39(5)	6(5)	35(5)
C60B	132(16)	103(13)	170(20)	82(16)	64(16)	75(13)
C61B	150(20)	94(13)	180(30)	68(16)	80(20)	63(14)

Table 10: Bond Lengths in Å for **4a**.

Atom	Atom	Length/Å
O1	C1	1.209(5)
O2	C2	1.222(5)
N1	C1	1.415(6)
N1	C2	1.389(6)
N1	C8	1.470(5)
N2	C5	1.400(6)
N2	C16	1.422(8)
N2	C22	1.415(8)
C1	C6[1]	1.466(6)
C2	C3	1.469(6)
C3	C4	1.372(6)
C3	C7[1]	1.415(6)
C4	C5	1.401(6)
C5	C6	1.404(6)
C6	C7	1.421(6)
C7	C7[1]	1.411(8)
C8	C9	1.507(8)
C9	C12	1.525(9)
C9	C10A	1.405(11)
C9	C10B	1.370(17)
C12	C13	1.417(14)
C13	C14	1.508(12)
C14	C15B	1.57(2)
C14	C15A	1.57(2)
C16	C17	1.373(9)
C16	C21	1.385(8)
C17	C18	1.380(8)
C18	C19	1.371(9)
C19	C20	1.363(13)
C20	C21	1.367(12)
C22	C23	1.412(9)
C22	C27	1.383(10)
C23	C24	1.382(13)
C24	C25	1.400(17)
C25	C26	1.368(13)
C26	C27	1.391(10)
C10A	C11A	1.543(16)
C10B	C11B	1.54(2)
O51	C51	1.225(5)
O52	C52	1.219(5)
N51	C51	1.387(5)
N51	C52	1.403(5)
N51	C58	1.471(5)
N52	C56	1.411(6)
N52	C66	1.424(7)
N52	C72	1.431(7)
C51	C55	1.466(6)
C52	C53	1.461(6)
C53	C54	1.427(6)
C53	C56	1.400(6)
C54	C54[2]	1.399(8)
C54	C55	1.417(6)
C55	C57	1.367(6)
C56	C57[2]	1.410(6)
C58	C59	1.509(8)
C59	C62	1.546(7)
C59	C60A	1.475(9)
C59	C60B	1.371(17)
C62	C63	1.506(8)
C63	C64	1.529(7)
C64	C65	1.494(9)
C66	C67	1.373(8)

Atom	Atom	Length/Å
C66	C71	1.405(8)
C67	C68	1.384(9)
C68	C69	1.380(10)
C69	C70	1.358(10)
C70	C71	1.397(9)
C72	C73	1.403(7)
C72	C77	1.361(9)
C73	C74	1.387(10)
C74	C75	1.369(12)
C75	C76	1.363(11)
C76	C77	1.391(9)
C60A	C61A	1.507(12)
C60B	C61B	1.48(2)

----

^{1}1-x,1-y,1-z; ^{2}-x,1-y,1-z

Table 11: Bond Angles in ° for **4a.**

Atom	Atom	Atom	Angle/°
C1	N1	C8	116.7(4)
C2	N1	C1	124.5(3)
C2	N1	C8	118.6(4)
C5	N2	C16	117.6(4)
C5	N2	C22	123.5(5)
C22	N2	C16	118.5(5)
O1	C1	N1	118.0(4)
O1	C1	C6^{1}	124.4(4)
N1	C1	C6^{1}	117.5(4)
O2	C2	N1	121.0(4)
O2	C2	C3	122.3(4)
N1	C2	C3	116.8(4)
C4	C3	C2	119.1(4)
C4	C3	C7^{1}	120.2(4)
C7^{1}	C3	C2	120.6(4)
C3	C4	C5	123.1(4)
N2	C5	C4	117.8(4)
N2	C5	C6	124.0(4)
C4	C5	C6	118.2(4)
C5	C6	C1^{1}	122.0(4)
C5	C6	C7	119.0(4)
C7	C6	C1^{1}	118.8(4)
C3^{1}	C7	C6	120.6(4)
C7^{1}	C7	C3^{1}	117.4(4)
C7^{1}	C7	C6	121.9(5)
N1	C8	C9	114.6(4)
C8	C9	C12	109.2(6)
C10A	C9	C8	117.8(7)
C10A	C9	C12	111.1(7)
C10B	C9	C8	127.4(11)
C10B	C9	C12	120.5(12)
C13	C12	C9	117.9(8)
C12	C13	C14	118.2(12)
C13	C14	C15B	112.7(14)
C13	C14	C15A	112.2(15)
C17	C16	N2	119.7(5)
C17	C16	C21	118.9(7)
C21	C16	N2	121.4(7)
C16	C17	C18	120.0(6)
C19	C18	C17	120.6(8)
C20	C19	C18	119.3(7)
C19	C20	C21	120.7(7)
C20	C21	C16	120.5(8)
C23	C22	N2	118.2(8)
C27	C22	N2	121.0(5)
C27	C22	C23	120.9(7)
C24	C23	C22	116.6(9)
C23	C24	C25	122.9(8)
C26	C25	C24	118.9(9)
C25	C26	C27	120.1(10)
C22	C27	C26	120.5(7)
C9	C10A	C11A	117.6(13)
C9	C10B	C11B	123(3)
C51	N51	C52	123.8(3)
C51	N51	C58	119.2(4)
C52	N51	C58	116.8(3)
C56	N52	C66	119.4(4)
C56	N52	C72	120.3(4)
C66	N52	C72	120.0(4)
O51	C51	N51	120.5(4)
O51	C51	C55	122.1(4)
N51	C51	C55	117.3(4)
O52	C52	N51	118.5(4)
O52	C52	C53	123.1(4)
N51	C52	C53	118.4(4)
C54	C53	C52	118.0(4)
C56	C53	C52	122.8(4)
C56	C53	C54	119.0(4)
C54^{2}	C54	C53	121.0(5)
C54^{2}	C54	C55	118.3(5)
C55	C54	C53	120.6(3)
C54	C55	C51	120.0(4)
C57	C55	C51	119.4(4)
C57	C55	C54	120.5(4)
C53	C56	N52	123.6(4)
C53	C56	C57^{2}	118.8(4)
C57^{2}	C56	N52	117.5(4)
C55	C57	C56^{2}	121.8(4)
N51	C58	C59	114.6(4)
C58	C59	C62	109.2(5)
C60A	C59	C58	118.5(5)
C60A	C59	C62	112.9(5)
C60B	C59	C58	127.0(9)
C60B	C59	C62	113.9(11)
C63	C62	C59	117.5(5)
C62	C63	C64	113.8(5)
C65	C64	C63	114.2(5)
C67	C66	N52	120.0(5)
C67	C66	C71	120.3(6)
C71	C66	N52	119.7(6)
C66	C67	C68	120.2(6)
C69	C68	C67	120.1(8)
C70	C69	C68	120.0(7)
C69	C70	C71	121.4(7)

Atom	Atom	Atom	Angle/˚
C70	C71	C66	118.0(7)
C73	C72	N52	118.9(6)
C77	C72	N52	120.9(4)
C77	C72	C73	120.2(6)
C74	C73	C72	118.6(7)
C75	C74	C73	120.5(6)
C76	C75	C74	120.4(8)
C75	C76	C77	120.0(9)
C72	C77	C76	120.1(6)
C59	C60A	C61A	115.3(7)
C59	C60B	C61B	113.0(17)

----

[1]1-x,1-y,1-z; [2]-x,1-y,1-z

Table 12: Torsion Angles in ˚ for **4a**.

Atom	Atom	Atom	Atom	Angle/˚
O2	C2	C3	C4	7.2(7)
O2	C2	C3	C7[1]	-176.0(4)
N1	C2	C3	C4	-171.5(4)
N1	C2	C3	C7[1]	5.3(6)
N1	C8	C9	C12	175.8(6)
N1	C8	C9	C10A	-56.4(9)
N1	C8	C9	C10B	15.3(18)
N2	C5	C6	C1[1]	-11.1(8)
N2	C5	C6	C7	173.8(5)
N2	C16	C17	C18	-177.9(5)
N2	C16	C21	C20	179.3(6)
N2	C22	C23	C24	177.5(8)
N2	C22	C27	C26	-179.4(6)
C1	N1	C2	O2	180.0(4)
C1	N1	C2	C3	-1.3(6)
C1	N1	C8	C9	-80.1(6)
C1[1]	C6	C7	C3[1]	8.8(6)
C1[1]	C6	C7	C7[1]	-170.0(5)
C2	N1	C1	O1	171.0(4)
C2	N1	C1	C6[1]	-7.4(6)
C2	N1	C8	C9	104.8(6)
C2	C3	C4	C5	178.3(4)
C3	C4	C5	N2	-177.0(5)
C3	C4	C5	C6	2.6(7)
C4	C5	C6	C1[1]	169.2(4)
C4	C5	C6	C7	-5.9(7)
C5	N2	C16	C17	-67.7(6)
C5	N2	C16	C21	113.6(6)
C5	N2	C22	C23	149.4(6)
C5	N2	C22	C27	-32.1(8)
C5	C6	C7	C3[1]	-175.9(4)
C5	C6	C7	C7[1]	5.3(8)
C7[1]	C3	C4	C5	1.5(7)
C8	N1	C1	O1	-3.8(6)
C8	N1	C1	C6[1]	177.8(4)
C8	N1	C2	O2	-5.4(7)
C8	N1	C2	C3	173.3(4)
C8	C9	C12	C13	-65.0(10)
C8	C9	C10A	C11A	167.9(15)
C8	C9	C10B	C11B	-141(3)
C9	C12	C13	C14	-172.9(8)
C12	C9	C10A	C11A	-65.1(18)
C12	C9	C10B	C11B	60(4)
C12	C13	C14	C15B	59.8(17)
C12	C13	C14	C15A	-170.7(12)
C16	N2	C5	C4	-38.7(7)
C16	N2	C5	C6	141.7(5)
C16	N2	C22	C23	-38.4(7)

Atom	Atom	Atom	Atom	Angle/°
C16	N2	C22	C27	140.2(6)
C16	C17	C18	C19	-0.9(9)
C17	C16	C21	C20	0.6(9)
C17	C18	C19	C20	-0.3(11)
C18	C19	C20	C21	1.7(13)
C19	C20	C21	C16	-1.9(12)
C21	C16	C17	C18	0.7(8)
C22	N2	C5	C4	133.7(5)
C22	N2	C5	C6	-46.0(7)
C22	N2	C16	C17	119.5(6)
C22	N2	C16	C21	-59.1(7)
C22	C23	C24	C25	2.7(17)
C23	C22	C27	C26	-0.9(10)
C23	C24	C25	C26	-2(2)
C24	C25	C26	C27	0.4(18)
C25	C26	C27	C22	1.2(13)
C27	C22	C23	C24	-1.0(11)
C10A	C9	C12	C13	163.5(9)
C10B	C9	C12	C13	97.1(18)
O51	C51	C55	C54	174.5(4)
O51	C51	C55	C57	-7.6(7)
O52	C52	C53	C54	161.0(4)
O52	C52	C53	C56	-13.7(7)
N51	C51	C55	C54	-8.1(6)
N51	C51	C55	C57	169.8(4)
N51	C52	C53	C54	-15.8(6)
N51	C52	C53	C56	169.5(4)
N51	C58	C59	C62	-171.2(4)
N51	C58	C59	C60A	57.7(7)
N51	C58	C59	C60B	-28.1(17)
N52	C66	C67	C68	-180.0(5)
N52	C66	C71	C70	179.7(4)
N52	C72	C73	C74	179.1(5)
N52	C72	C77	C76	179.1(7)
C51	N51	C52	O52	-167.6(4)
C51	N51	C52	C53	9.3(6)
C51	N51	C58	C59	-106.5(5)
C51	C55	C57	C56[2]	-179.2(4)
C52	N51	C51	O51	-179.9(4)
C52	N51	C51	C55	2.6(6)
C52	N51	C58	C59	78.9(5)
C52	C53	C54	C54[2]	-169.3(5)
C52	C53	C54	C55	10.6(6)
C52	C53	C56	N52	-14.6(7)
C52	C53	C56	C57[2]	167.1(4)
C53	C54	C55	C51	1.3(6)
C53	C54	C55	C57	-176.5(4)
C54	C53	C56	N52	170.7(4)
C54	C53	C56	C57[2]	-7.5(7)
C54[2]	C54	C55	C51	-178.8(5)
C54[2]	C54	C55	C57	3.4(8)
C54	C55	C57	C56[2]	-1.4(7)
C56	N52	C66	C67	-57.6(6)
C56	N52	C66	C71	123.2(5)
C56	N52	C72	C73	142.8(5)
C56	N52	C72	C77	-37.3(7)
C56	C53	C54	C54[2]	5.6(8)
C56	C53	C54	C55	-174.5(4)
C58	N51	C51	O51	5.8(7)
C58	N51	C51	C55	-171.6(4)
C58	N51	C52	O52	6.7(6)

Atom	Atom	Atom	Atom	Angle/°
C58	N51	C52	C53	-176.3(4)
C58	C59	C62	C63	69.1(7)
C58	C59	C60A	C61A	74.5(8)
C58	C59	C60B	C61B	159.3(16)
C59	C62	C63	C64	-176.8(6)
C62	C59	C60A	C61A	-54.9(9)
C62	C59	C60B	C61B	-59(2)
C62	C63	C64	C65	-177.6(6)
C66	N52	C56	C53	142.6(5)
C66	N52	C56	C57[2]	-39.1(6)
C66	N52	C72	C73	-43.3(7)
C66	N52	C72	C77	136.6(6)
C66	C67	C68	C69	0.7(9)
C67	C66	C71	C70	0.4(7)
C67	C68	C69	C70	-0.4(10)
C68	C69	C70	C71	0.1(10)
C69	C70	C71	C66	-0.1(9)
C71	C66	C67	C68	-0.7(8)
C72	N52	C56	C53	-43.4(6)
C72	N52	C56	C57[2]	134.8(5)
C72	N52	C66	C67	128.4(5)
C72	N52	C66	C71	-50.8(6)
C72	C73	C74	C75	2.9(11)
C73	C72	C77	C76	-1.0(10)
C73	C74	C75	C76	-3.1(14)
C74	C75	C76	C77	1.2(15)
C75	C76	C77	C72	0.9(13)
C77	C72	C73	C74	-0.8(9)
C60A	C59	C62	C63	-156.8(7)
C60B	C59	C62	C63	-79.3(14)

----

[1]1-x,1-y,1-z; [2]-x,1-y,1-z

Table 13: Hydrogen Fractional Atomic Coordinates (×10^4) and Equivalent Isotropic Displacement Parameters (Å^2×10^3) for **4a**. U_{eq} is defined as 1/3 of the trace of the orthogonalised U_{ij}.

Atom	x	y	z	U_{eq}
H4	6761.55	4731.72	6842.33	88
H8A	8413.57	2742.34	4257.33	98
H8B	8167.95	2910.82	3298.95	98
H9A	6642.09	1414.22	2488.75	144
H9B	7301.56	1307.74	3630.78	144
H12A	8871.48	1068.6	3226.79	201
H12B	7860.53	148.05	2328.9	201
H13A	9135.43	1923.07	2357.75	236
H13B	8028.29	1109.61	1477.31	236
H14A	10260.8	621.06	2007.36	329
H14B	9835.92	805.17	1071.24	329
H14C	9097.14	-200.04	1037.48	329
H14D	10170.19	495.84	2002.35	329
H17	4287.98	4546.08	7796.18	122
H18	4611.62	3559.49	8631.79	147
H19	6473.2	3897.23	9675	182
H20	8019.19	5210.11	9863.88	205
H21	7693.64	6244.3	9090.84	171
H23	5122.11	7092.73	9532.12	188
H24	5465.66	8917.74	10518.39	258
H25	6261.88	10123.2	10019.39	280
H26	6869.28	9502.99	8558.72	210

Atom	x	y	z	U_{eq}
H27	6649.02	7686.42	7589.42	143
H10A	6064.35	1394.92	3813.3	163
H10B	7191.8	911.78	4000.37	163
H11A	5320.21	-171.15	2221.86	255
H11B	5337.98	-402.46	3140.83	255
H11C	6368.89	-660.7	2560.34	255
H15A	8502.22	-916.23	1242.29	354
H15B	9578.31	-1079.44	704.38	354
H15C	8403.34	-793.75	260.15	354
H10C	5878.68	903.12	1970.22	130
H10D	5586.23	1521.09	3004.03	130
H11D	5551.01	-641.63	1991.42	240
H11E	4618.9	-87.05	2583.02	240
H11F	5883.12	-113.9	3166.38	240
H15D	10725.92	574.89	615.35	526
H15E	9576.36	1037.08	418.8	526
H15F	10649.22	1732.83	1383.5	526
H57	577.62	3931.25	2714.33	82
H58A	3823.22	3373.05	5462.51	96
H58B	3413.15	2735.64	4289.98	96
H59	2544.4	2093.27	5582.09	117
H59A	2422.24	1380.23	4246.29	117
H62A	3696.52	734.01	5011.85	136
H62B	4359.3	1423.3	4591.21	136
H63A	4473.41	2090.88	6612.35	121
H63B	5091.02	2826.93	6210.86	121
H64A	5751.41	899.53	6004.84	134
H64B	6390.03	1672.58	5649.09	134
H65A	7087.26	2986.31	7309.35	199
H65B	7599.72	1946.51	7107.55	199
H65C	6516.47	2154.59	7634.41	199
H67	2280.87	6689.36	8346.92	121
H68	2570.92	8088.85	9915.76	148
H69	1441.8	7937.68	10972.57	156
H70	20.11	6418.3	10466.18	146
H71	-316.7	4992.98	8885.99	127
H73	2269.19	4274.41	8525.34	135
H74	2244.93	2567.17	8347.99	174
H75	886.79	1125.56	7050.77	209
H76	-577.82	1365.51	6006.5	203
H77	-645.1	3069.72	6215.72	145
H60A	1104.71	1377.71	4102.94	129
H60B	1531.68	538.08	4428.34	129
H61A	1334.31	-110.1	2754.84	192
H61B	2727.9	123.9	3278.13	192
H61C	2235.49	930.99	2932.06	192
H60C	1247.6	1934.68	5252.87	143
H60D	2281.66	1979.41	6102.68	143
H61D	1162.81	149.61	4307.45	201
H61E	943.58	361.73	5357.95	201
H61F	2222.89	187.9	5136.68	201

Table 14: Atomic Occupancies for all atoms that are not fully occupied in **4a.**

Atom	Occupancy	Atom	Occupancy	Atom	Occupancy	Atom	Occupancy
H9A	0.754(15)	H14B	0.427(19)	C10A	0.754(15)	C11A	0.754(15)
H9B	0.246(15)	H14C	0.573(19)	H10A	0.754(15)	H11A	0.754(15)
H14A	0.427(19)	H14D	0.573(19)	H10B	0.754(15)	H11B	0.754(15)

Atom	Occupancy
H11C	0.754(15)
C15B	0.427(19)
H15A	0.427(19)
H15B	0.427(19)
H15C	0.427(19)
C10B	0.246(15)
H10C	0.246(15)
H10D	0.246(15)
C11B	0.246(15)
H11D	0.246(15)
H11E	0.246(15)
H11F	0.246(15)
C15A	0.573(19)
H15D	0.573(19)
H15E	0.573(19)
H15F	0.573(19)
H59	0.745(9)
H59A	0.255(9)
C60A	0.745(9)
H60A	0.745(9)
H60B	0.745(9)
C61A	0.745(9)
H61A	0.745(9)
H61B	0.745(9)
H61C	0.745(9)
C60B	0.255(9)
H60C	0.255(9)
H60D	0.255(9)
C61B	0.255(9)
H61D	0.255(9)
H61E	0.255(9)
H61F	0.255(9)

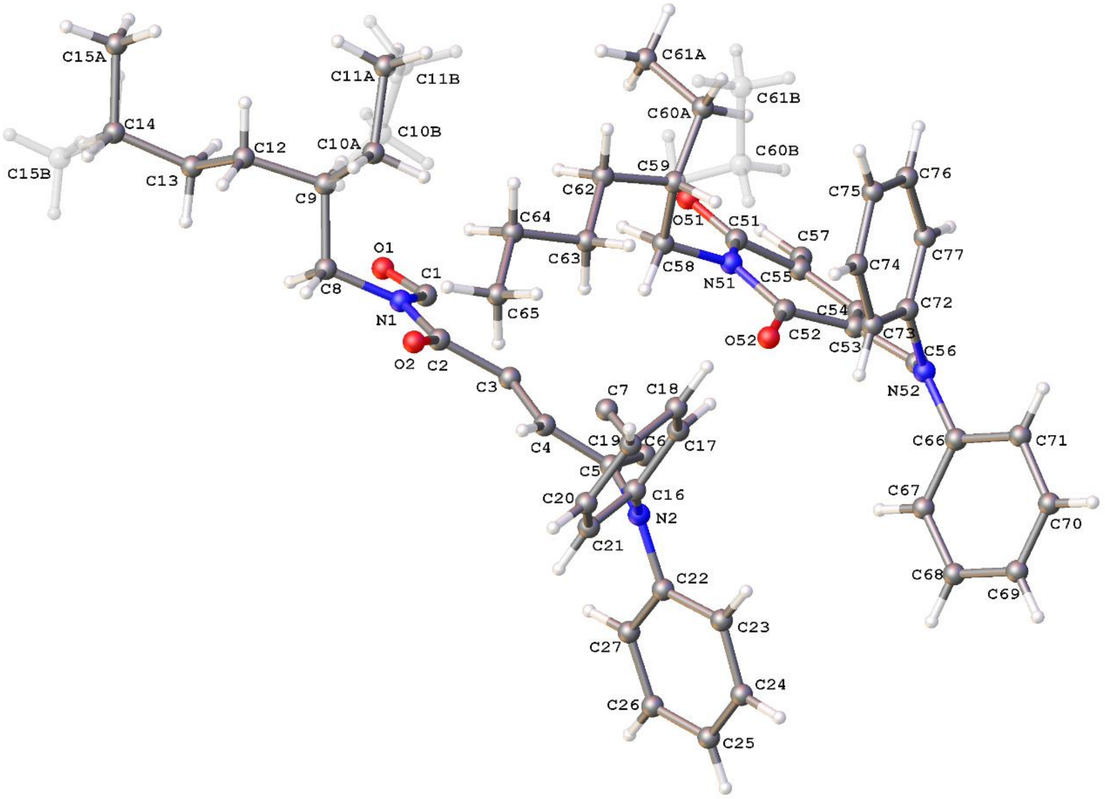

Fig S30 Asymmetric unit of **4a** showing two independent half molecules (the other halves are generated by crystallographic inversion symmetry). Minor disorder components are shown 'ghosted' for clarity

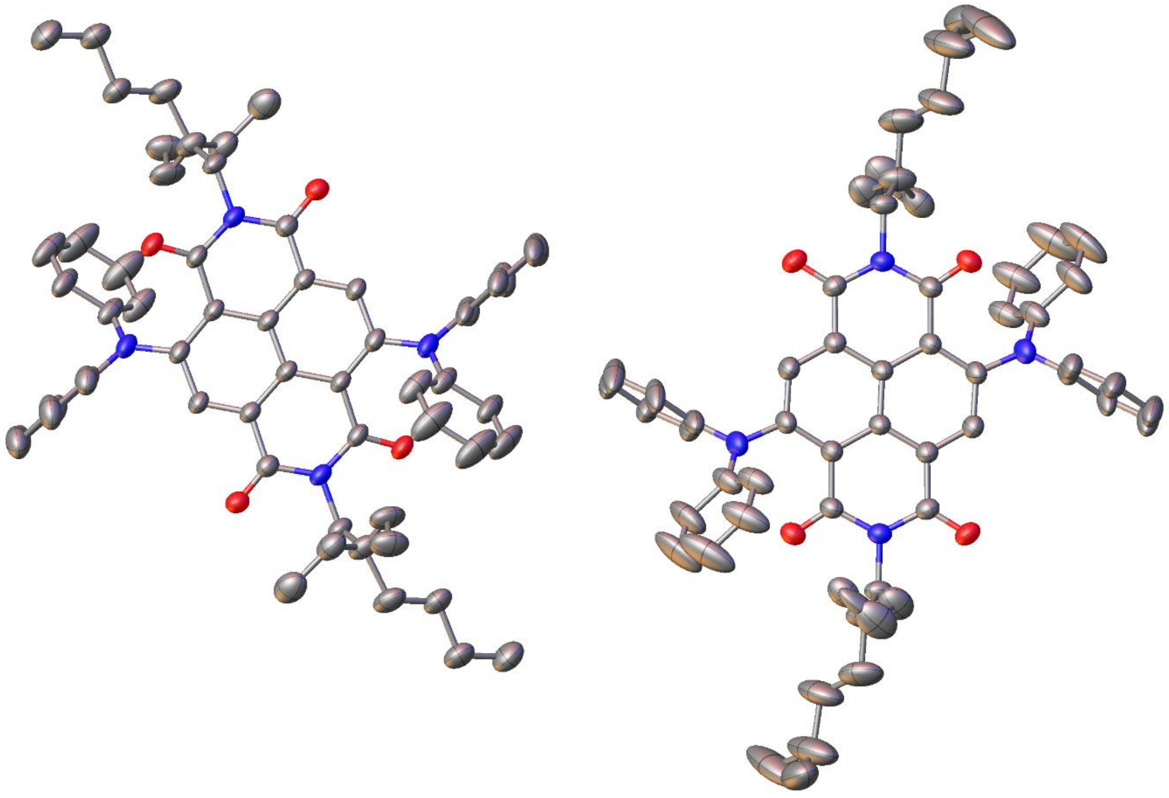

Fig S31 Both independent molecules of **4a** including crystallographic symmetry generated halves. Hydrogen atoms are omitted for clarity. ADP ellipsoids are displayed at 30% probability.

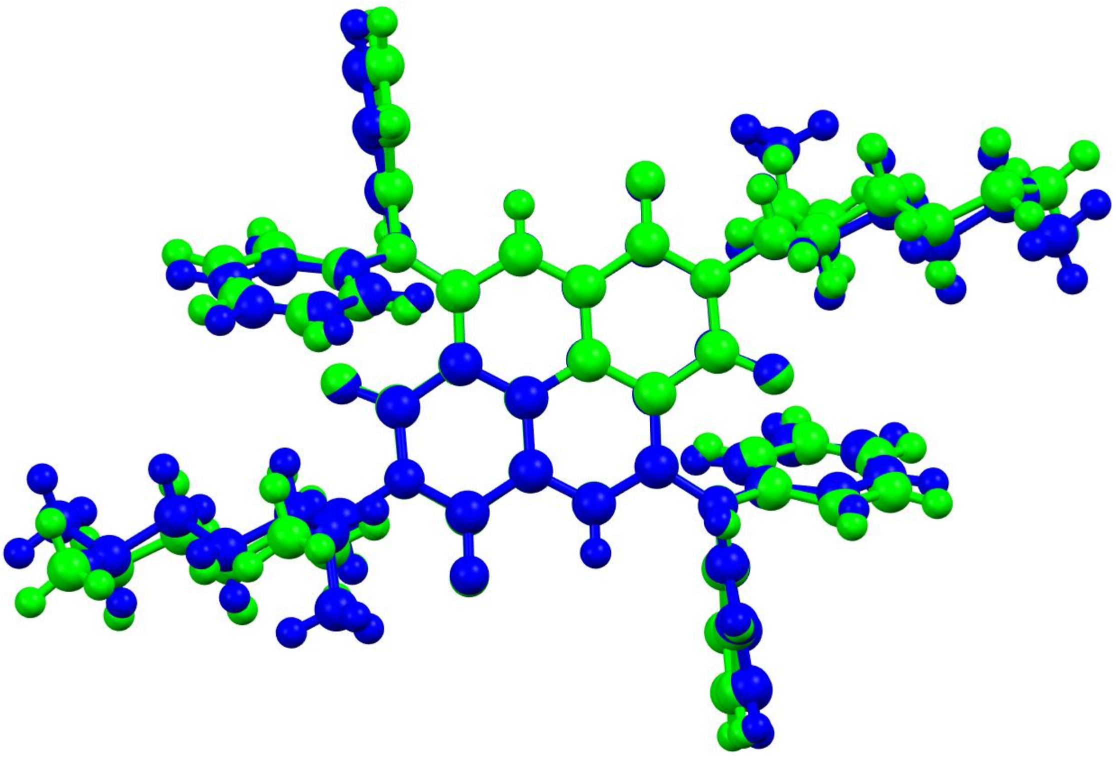

Fig S32 Molecular overlay of both independent molecules of **4a** including crystallographic symmetry generated halves (minor disorder components have been omitted) showing conformational differences.

S4.0 References

1 G. M. Sheldrick, *Acta Crystallogr. Sect. C Struct. Chem.*, **2015**, 71, 3–8.

2 O. V. Dolomanov, L. J. Bourhis, R. J. Gildea, J. A. K. Howard and H. Puschmann, *J. Appl. Crystallogr.*, **2009**, 42, 339–341.

3 G. M. Sheldrick, *Acta Crystallogr. Sect. A Found. Adv.*, **2015**, 71, 3–8.

4 M. Sasikumar, Y. V. Suseela and T. Govindaraju, *Asian J. Org. Chem.*, **2013**, 2, 779–785.

5 Y. V. Suseela, M. Sasikumar and T. Govindaraju, *Tetrahedron Lett.*, **2013**, 54, 6314–6318.

6 X. Guo and M. D. Watson, *Org. Lett.*, **2008**, 10, 5333–5336.

7 K. Chen, J. Zhao, X. Li and G. G. Gurzadyan, *J. Phys. Chem. A*, **2019**, 123, 2503–2516.

8 M. Hussain, A. M. El-Zohry, Y. Hou, A. Toffoletti, J. Zhao, A. Barbon and O. F. Mohammed, *J. Phys. Chem. B*, **2021**, 125, 10813–10831.

9 Z. Wu, C. Sun, S. Dong, X. F. Jiang, S. Wu, H. Wu, H. L. Yip, F. Huang and Y. Cao, *J. Am. Chem. Soc.*, **2016**, 138, 2004–2013.

10 K. Rundel, S. Maniam, K. Deshmukh, E. Gann, S. K. K. Prasad, J. M. Hodgkiss, S. J. Langford and C. R. McNeill, *J. Mater. Chem. A*, **2017**, 5, 12266–12277.
